# Atmospheric reaction of hydrazine plus hydroxyl radical

**DOI:** 10.1038/s41598-021-92563-8

**Published:** 2021-06-24

**Authors:** Hamed Douroudgari, Morteza Vahedpour, Fahime Khouini

**Affiliations:** Department of Chemistry, University of Zanjan, PO Box 38791-45371, Zanjan, Iran

**Keywords:** Computational chemistry, Astrochemistry

## Abstract

Understanding the mechanism of hydrazine oxidation reaction by OH radical along with the rate constants of all possible pathways leads to explain the fate of hydrazine in the atmosphere. In this article, the comprehensive mechanisms and kinetics of the hydrazine plus hydroxyl radical reaction have been investigated theoretically at different temperatures and pressures. To achieve the main goals, a series of high levels of quantum chemical calculations have been widely implemented in reliable channels of the H-abstraction, S_N_2, and addition/elimination reactions. The energy profile of all pathways accompanied by the molecular properties of the involved stationary points has been characterized at the MP2, M06-2X, and CCSD(T)/CBS levels. To estimate accurate barrier energies of the H-abstraction channels, large numbers of the CCSD (T) calculations in conjunction with various augmented basis sets have been implemented. The direct dynamic calculations have been carried out using the validated M06-2X/maug-cc-pVTZ level, and also by the CCSD(T) (energies) + MP2 (partition functions) level. The pressure-dependent rate constants of the barrierless pathways have been investigated by the strong collision approach. Therefore, the main behaviors of the N_2_H_4_ + OH reaction have been explored according to the influences of temperature and pressure on the computed rate coefficients within the well-behaved theoretical frameworks of the TST, VTST, and RRKM theories. It has been found that the H-abstraction mechanism (to form N_2_H_3_) is dominant relative to the S_N_2 reaction and OH-addition to the N center of N_2_H_4_ moiety (to form H_2_NOH + NH_2_). The computed high pressure limit rate constant of the main reaction pathway, k(298.15) = 7.31 × 10^–11^ cm^3^ molecule^−1^ s^−1^, has an excellent agreement with the experimental value (k (298.15) = (6.50 ± 1.3) × 10^–11^ cm^3^ molecule^−1^ s^−1^) recommended by Vaghjiani. Also, the atmospheric lifetime of hydrazine degradation by OH radicals has been demonstrated to be 32.80 to 1161.11 h at the altitudes of 0–50 km. Finally, the disagreement in the calculated rate constants between the previous theoretical study and experimental results has been rectified.

## Introduction

Hydrazine has three structural conformers: *s-cis* (C_2h_), *anti* (C_2v_), and *gauche* (C_2_). The gauche-conformer is the most stable, and the anti-conformer is more stable than the *cis*. In industry, hydrazine and its derivatives have many applications such as polymerization initiators, boiler water treatment, dyes^1–5^, and corrosion inhibitors for steel in contact with hot water^6–8^. Also, hydrazine plays an important role in the production of organic compounds^9^. The International Agency for Research on Cancer (IARC) and American Conference of Governmental Industrial Hygienists (ACGIH) take into account hydrazine as a carcinogenic substance with an unknown relevance for both animals and humans^10^. In unsymmetrical structures, the fuels containing hydrazine, methyl hydrazine, and dimethylhydrazine compounds are high-energy and have special applications such as fuels of rockets and spacecraft. All of them are crudely toxic^11–14^. Usually, some of the oxidizers are added to hydrazine fuels for high performance. The most common oxidizers are dinitrogen tetroxide (NTO) and inhibited red fuming nitric acid (IRFNA)^15–17^. Because decomposition of NTO and nitric acid can produce hydroxyl radicals and nitrogen dioxide, the reactions of hydrazine with these species are important in designing rockets^18^. Knowledge of hydrazine oxidation reactions has some advantages. The most important advantage is the development of the gelled hypergolic propellant (GHP)^19^. In the atmosphere, the complete decomposition of hydrazine with reactive species is probable^20^. A few numbers of experimental studies^21–24^ and only one theoretical investigation^25^ have been performed previously on the mechanisms and kinetics of the N_2_H_4_ + OH reaction in the second-order form. The obtained results have been shown that hydrogen abstraction via hydroxyl radical is the main reaction pathway at temperatures lower than 700 K.

1 $${\text{N}}_{{\text{2}}} {\text{H}}_{{\text{4}}} + {\text{ OH}} \to {\text{N}}_{{\text{2}}} {\text{H}}_{{\text{3}}} + {\text{ H}}_{{\text{2}}} {\text{O}}{\text{.}}$$

Also, the main reaction of hydrazine with O_3_ as other important atmospheric species is as follows:^12,26^

2 $${\text{N}}_{{\text{2}}} {\text{H}}_{{\text{4}}} + {\text{ O}}_{{\text{3}}} \to {\text{N}}_{{\text{2}}} {\text{H}}_{{\text{3}}} + {\text{ OH }} + {\text{ O}}_{{\text{2}}} .$$

Vaghjiani^21^ experimentally investigated the gas-phase rate constant of OH reaction with the hydrazine in the temperature range of 232–374 K and reported an expression as k = (1.25 ± 0.19) × 10^−11^ exp[(315 ± 55)/T] cm^3^ molecule^−1^ s^−1^. This expression has a weak temperature dependence. In another study, he reported another Arrhenius expression as k = (2.17 ± 0.39) × 10^−11^ exp[(160 ± 30)/T] cm^3^ molecule^−1^ s^−1^ at the 232–637 K temperature range. Also, he recommended that OH radicals can not be added to one center of diamine in low temperatures, and thus the reaction does not progress by rapid dissociation of the intermediate into products^22^. Harris et al.^23^ measured the rate constants of the title reaction by using photolysis-resonance fluorescence in the 298–424 K temperature range. They found a rate expression as k = 4.40 × 10^−11^ exp[(116 ± 176)/T] cm^3^ molecule^−1^ s^−1^. Hack et al.^24^ investigated the rate of hydrazine with hydroxyl radical, OH + N_2_H_4_ → products, in an isothermal flow reactor with helium flow as the carrier gas. The overall rate constant was k(298.15) = 2.20 × 10^–11^ cm^3^ molecule^−1^ s^−1^ at room temperature and pressure around 2 Torr. The reaction of N_2_H_4_ + OH was studied by Tang and et al*.*^25^ theoretically. The calculated rate expressions over 200–3000 K temperature range were k_1_ = 7.79 × 10^−18^ T^1.93^exp(− 1258.5/T) and k_2_ = 1.28 × 10^−18^ T^2.37^exp(− 1049.3/T) cm^3^ molecule^−1^ s^−1^ for H-abstraction reaction (generation of the N_2_H_3_ plus H_2_O products) and S_N_2 reaction (production of the NH_2_ plus H_2_NOH adducts), respectively. For the N_2_H_4_ reactant, the following results show that there is an inconsistency between the reported optimized geometry by Tang et al. and the original geometry of this reactant at the B3lyp/6-311g(d,p) level. On one hand, the B3LYP/6-311G(d,p) and the CCSD(T)/6-311 + + G(d,p)//B3LYP/6-311G(d,p) levels are weak computational levels to describe a potential energy surface especially in reactions including small compounds such as the N_2_H_4_ plus OH reaction^27–30^. On the other hand, the computed relative energies at this level were corrected and found that these levels have no accurate transition structures and energies. In addition, on the basis of our results, there are large discrepancies between the rate constants calculated at the CCSD(T)/6-311 +  + G(d,p)//B3LYP/6-311G(d,p) level and the abovementioned experimental results, so further calculations are needed to resolve the problem. Therefore, it is concluded that higher-level calculations are necessary to compute the saddle points structures and the potential energy surface of the title reaction. It will be discussed in more detail in the result and discussion section.

To the best of our knowledge, there are neither experimental studies at temperatures above 650 K and nor reliable theoretical data obtained by higher-level calculations. In the present work, the hydrazine plus hydroxyl radical reaction channels are investigated using both single reference and multi-reference methods to yield a more accurate potential energy surface, to calculate the exact rate constant for each channel at various temperatures, and to predict more precise thermodynamic parameters. Therefore, the potential energy surface is computed using higher-level methods such as the CASSCF-MP2 method (the multi-reference MØller–Plesset second-order perturbation theory), the CCSD(T) method (the coupled-cluster theory), and the MP2 method (the single reference MØller–Plesset second-order perturbation theory) in connection with various basis sets to obtain more accurate and complete treatment of the N_2_H_4_ + OH reaction pathways. Furthermore, the other main objectives of this study are fully and completely describing the mechanisms of the N_2_H_4_ + OH reaction comprehensively, and obtaining more accurate theoretical insight into the stationary points of computed PES using the topological theory of atoms in molecules (AIM) and NBO analysis. The rate constant calculations are carried out for all reaction pathways at the high-pressure limits by using transition state (TST) and variational transition state VTST theories, and at the low-pressure limit and falloff regime by the strong collision master equation Rice–Ramsperger–Kassel–Marcus (RRKM) theory.

## Computational methods

The UMP2^31^ and very popular density functional method, UB3LYP^32,33^, with the 6-311 +  + G (3df, 3pd), maug-cc-pVTZ^34^, aug-cc-pVTZ, and aug-cc-pVQZ^35^ basis sets and also some other basis sets were applied to geometry optimization of all stationary points in doublet state. Due to serious shortcomings of the most popular density functional method, B3LYP, in determining barrier heights and so kinetics of reactions, especially in reactions containing hydrogen shifts, we have used the UM06-2X^36,37^ method. This method is a highly parameterized meta hybrid density functional method and has reasonable results for specifying reaction kinetics.

The harmonic vibrational frequencies were computed by using the abovementioned methods and basis sets to determine the nature of all stationary points, including prereactive collision complexes (MCr), product complexes (MCp), and transition states (TS). In the whole paper, It was used the maTZ, aTZ, and aQZ for shorthand notations of the maug-cc-pVTZ^34^, aug-cc-pVTZ, and aug-cc-pVQZ^35^ basis sets, respectively. All of the structures concerning the minimum-energy points on the considered PES have only real frequencies, and all of the saddle point structures have just one imaginary frequency.

The zero-point vibrational energies were included in the calculated relative energies. Moreover, to evaluate the connectivity of all stationary points and also to compute the minimum-energy paths, we carried out the intrinsic reaction coordinate (IRC)^38,39^ calculations (MEP) for all saddle point structures using the MP2 and M06-2X methods.

**Figure 1:**
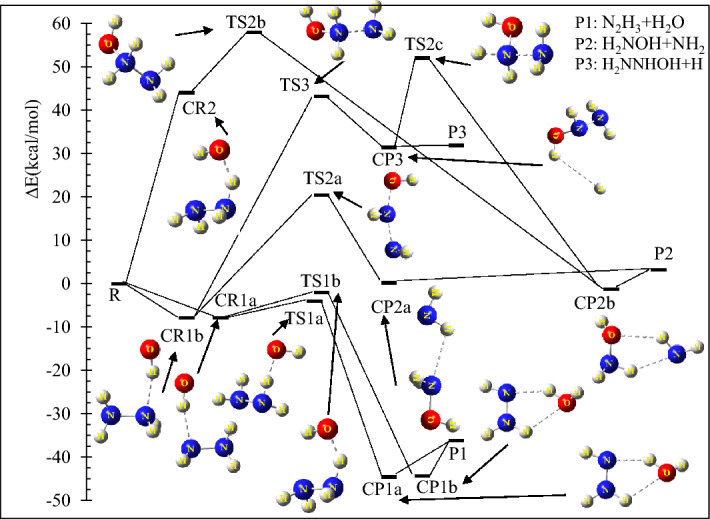
Potential energy profile of the N_2_H_4_ + OH reaction for reliable pathways at the CCSD(T)/CBS//MP2/aTZ level.

To estimate the barrier heights of the title reaction more precisely, a dual-level methodology was used similar to previous reactions of hydrazine with atomic oxygen simulated in the atmospheric conditions^40–42^. Thus, the CCS(T)/CBS//MP2/aTZ level was chosen. Because (a) (in the mentioned studies) it has been proved that the CCS(T)/CBS level has excellent results in energy prediction (b) the MP2 method along with a large basis set has better results than small basis sets^43^ for geometry optimization of all stationary points containing hydrogen bonds^44^ like the N_2_H_4_ + OH reaction. Therefore, we used the geometries obtained at the MP2/aTZ level for the CCSD(T)/CBS calculations. For constructing the CCSD(T)/CBS level, the results of the CCSD(T)/aQZ//UMP2/aTZ and CCSD(T)/aTZ//UMP2/aTZ levels were extrapolated to the complete basis set, CBS, limit using the suggested method by Halkier et al.^45^. To evaluate the effects of excited states on the potential energy surface, the multi-reference MØller–Plesset second-order perturbation method, MR-MP2^46,47^, in connection with the augmented correlation-consistent polarized quadruple-zeta, aug-cc-pVQZ, basis set were implemented on the structures obtained at the UMP2/aTZ and UM06-2X/aTZ levels. The T1 diagnostic^48,49^ values were calculated at CCSD(T)/Y level (Y = several basis sets of the Dunning and Pople types) for confirming the importance of higher-level calculations to the electronic structures of all species. If T1 diagnostic values are greater than 0.045 for both closed-shell and open-shell systems, higher-level calculations are needed for investigating systems^50–52^. The same value for the Largest amplitudes is 0.2, but our results are below 0.2 (0.045) except TS1b (see Supplementary Table S36). This is obvious for TS1b because we found the structure of TS1b through the scan option as mentioned in the “H_2_NOH + NH_2_ formation channels” section. Also, according to the proposed PES, the path containing TS1b has a very small contribution to hydrazine degradation due to having a transition state with a high energy barrier (see Fig. 1). And this path is not observed in experimental studies. This is only a theoretical pathway and does not affect the total rate of hydrazine degradation.

To explore deeper insight about the reaction mechanisms, the bonds taking part in reactions were analyzed based on the natural bond orbital, NBO, and the atoms in molecule, AIM, analyses at the UMP2/aTZ level. Also, the thermodynamic parameters of the predicted adducts were computed in the temperature range of 200–1200 K at the CCSD(T)/CBS (energy) + UMP2/aTZ (corrections), CCSD(T)/CBS (energy) + M06-2X/aQZ (corrections), M06-2X/aQZ and MP2/aTZ levels. The topological analyses of the wave functions were implemented by the AIM2000^53^ program. Gaussian 09 package program was executed for optimization and electronic structure calculations of all stationary points^54^. All images of molecular structures were created using the GaussView 5.0.8 software^55^.

### Rate constant calculations

All rate constants were calculated at the high-pressure limit using the transition state (TST) theory^56^ as follows:

3 $$k(T) = \kappa \frac{{k_{B} T}}{h}\left( {\frac{{Q_{{TS}}^{{ \ne ,IG}} }}{{Q_{{N_{2} H_{4} }}^{{IG}} Q_{{OH}}^{{IG}} }}} \right)\exp \left( { - \frac{{\Delta ^{ \ne } E^{{el}} }}{{k_{B} T}}} \right),$$

where $\kappa$ is the tunneling factor. $Q_{X}^{{IG}}$ is the partition function of *X* component that is assumed to be an ideal gas. $k_{B}$ and $T$ are the Boltzmann constant and the absolute temperature, respectively. $h$ is the Planck constant. $\Delta ^{ \ne } E^{{el}}$ is the difference of electronic energies between a transition state and the sum of reactants. Also, the variational effects on the reaction rate were implemented by the variational transition state theory (VTST)^57^. For all elementary bimolecular reactions, the high-pressure limit rate constants were computed by the Gpop program^58^. The rate constants at the low-pressure limit and the falloff regime were calculated using the strong collision master equation/Rice–Ramsperger–Kassel–Marcus (RRKM) theory by the Ssumes program^59^.

The main channels of the title reaction in this work are hydrogen shifts. Thereby, the exact quantum tunneling correction factor is necessary. The quantum tunneling correction factor was calculated at the UMP2/aTZ and M06-2X/maTZ levels. For the mentioned correction, the second-order correction of Shavitt^60^ was used as follows:

$$k_{i} (T) = 1 - \left( {\frac{1}{{24}}} \right)\left( {\frac{{h\upsilon _{{im}} c}}{{k_{B} T}}} \right)^{2} \left( {\frac{{1 + k_{B} T}}{{E_{0} }}} \right),$$

where ν_im_ is the imaginary frequency of a transition state, *c* is the speed of light, and E_0_ is the barrier height that may correct by the zero-point energy for the considering reaction.

As the authors of the UM06-2X^36,37^ method have recommended this method has excellent performance for main group thermochemistry, noncovalent interactions, and kinetics. Therefore, the rate constants of the N_2_H_4_ + OH reaction were computed at the M06-2X/maTZ level.

## Results and discussion

In the following sections, for the N_2_H_4_ + OH reaction, the potential energy surface, the rate constants of all reliable pathways at low, intermediate, and high pressures, and also thermodynamic data are discussed using different computational methods. Finally, the fate of hydrazine is studied in different heights of the atmosphere. Also, we use the same notation for stationary points as Tang et al. to simplify the PES following in both works.

### Potential energy surface

**Figure 2:**
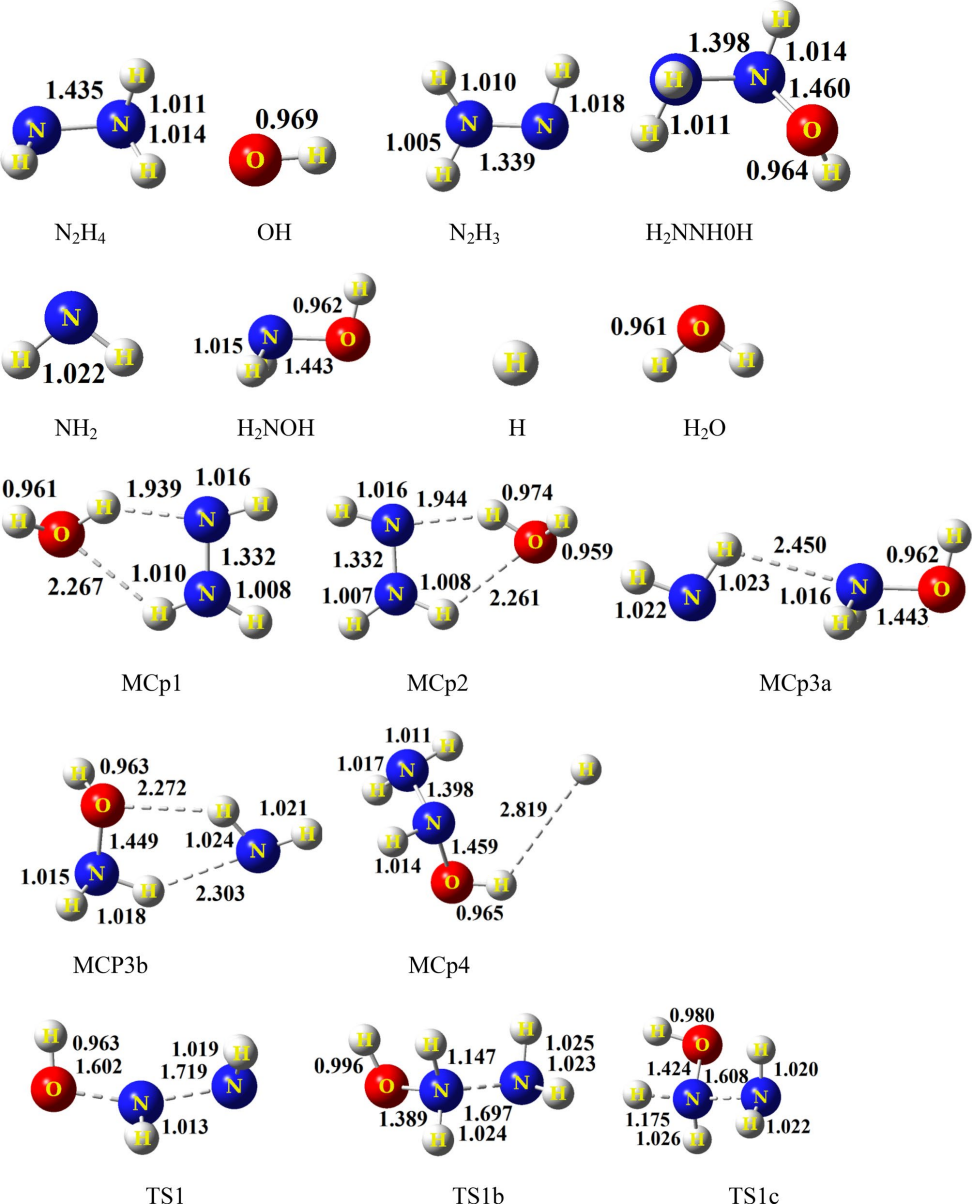

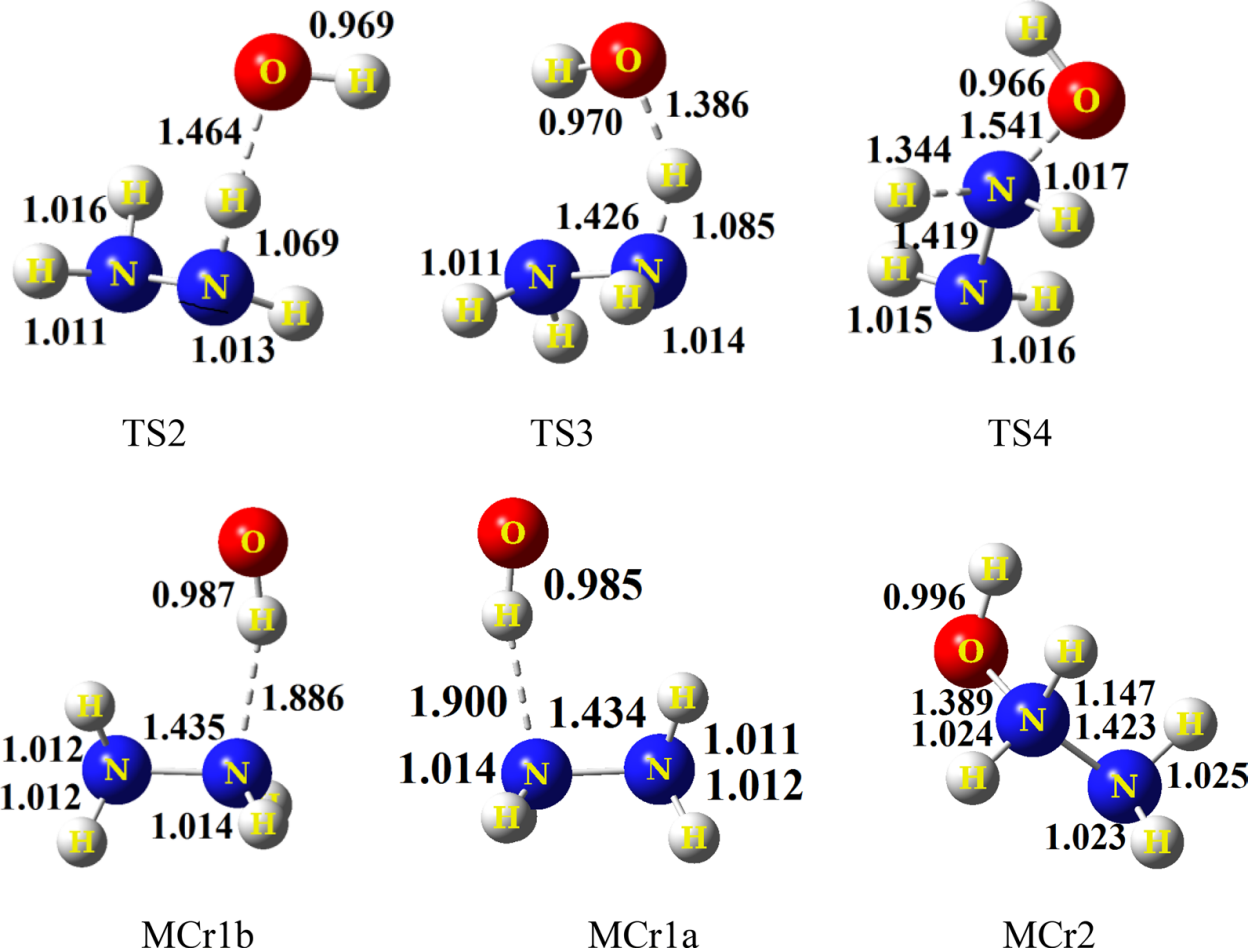
The optimized structural parameters of all species for reliable paths at the MP2/aTZ level.

The potential energy diagram of the N_2_H_4_ + OH reaction is depicted in Fig. 1. The relative energies of this figure are computed at the CCSD(T)/CBS level. The selected geometrical parameters of all species in the proposed pathways are shown in Fig. 2. Also, the selected bond lengths of key saddle points are listed in Table 1, Supplementary Tables S1, and S2 (see Supplementary information). The unscaled vibrational frequencies of stationary points are summarized in Supplementary Table S3. The energetic parameters of all stationary points computed at the CCSD(T)/CBS level are given in Table 2. Also, these parameters are calculated in several methods and various basis sets and collected in Supplementary Tables S4–S6 (see Supplementary information). The thermodynamic variables of all predicted adducts in the temperature range of 200–1200 K are calculated at both the UM06-2X/aQZ and UMP2/aTZ levels and collected in Table 4 and Supplementary Tables S33–S35. The temperature dependence of rate constants for all elementary reactions is calculated by TST and VTST theories. The obtained results are tabulated in and Supplementary Tables S7–S30 in additional information. The pressure-dependent rate constants for all H-abstraction channels are listed in Supplementary Table S31.

**Table 1:** Bond lengths (in Å) for TS2 and TS3.

Method	TS2		TS3	
	N–H	H–O	N–H	H–O
MP2/aTZ	1.069	1.464	1.087	1.386
MP2/maTZ	1.068	1.463	1.085	1.386
M06-2X/aTZ	1.031	1.742	1.047	1.580
M06-2X/maTZ	1.031	1.751	1.040	1.626
M06-HF/aTZ	1.045	1.562	1.064	1.466
M06-HF/maTZ	1.046	1.563	1.064	1.466
B3LYP/aTZ	1.034	1.700	1.023	1.809
B3LYP/maTZ	1.035	1.693	1.023	1.808

**Table 2:** The electronic energies (in Hartree), relative energies ($\Delta E$) (in kcal mol^−1^), forward barriers heights ($\Delta E_{f}^{ \ne }$) (in kcal mol^−1^), and reverse barrier heights ($\Delta E_{r}^{ \ne }$) (in kcal mol^−1^) of the reliable paths of N_2_H_4_ + OH reactions at the CCSD(T)/CBS level.

Species	$E_{{elec}}$	$\Delta E$	$\Delta E_{f}^{ \ne }$	$\Delta E_{r}^{ \ne }$
R (N_2_H_4_ + OH)	− 187.44049	0.00		
MCr1a	− 187.45305	− 7.88		
MCr2	− 187.37037	44.00		
TS1	− 187.40797	20.41	28.29	20.20
TS1b	− 187.34829	57.86	13.86	59.08
TS1c	− 187.35757	52.03	59.91	20.61
TS2	− 187.44540	− 3.08	4.80	41.37
TS3	− 187.44430	− 2.39	5.49	41.87
TS4	− 187.37169	43.17	51.05	11.75
MCp1	− 187.51133	− 44.45		
MCp2	− 187.51103	− 44.26		
MCp3a	− 187.44016	0.21		
MCp3b	− 187.44244	− 1.22		
MCp4	− 187.39042	31.42		
P1(N_2_H_3_ + H_2_O)	− 187.49810	− 36.15		
P2(NH_2_ + H_2_NOH)	− 187.43537	3.21		
P3(H_2_NNHOH + OH)	− 187.38978	31.82		

### Reaction entrance channels

Like many gas-phase reactions, the hydrazine reactions with hydroxyl radical begin with pre-reactive collision complexes. In this work, three pre-reaction complexes are predicted and named by MCr1a, MCr1b, and MCr2. The complex MCr1a is a mirror image of MCr1b. Therefore, the electronic structure of MCr1a is the same as MCr1b. Also, the structure of MCr1a (or MCr1b) including N_2_H_4_ and OH is associated with the formation of hydrogen-bond. So, they are more stable than MCr2. In MCr2, the hydroxyl radical moiety has a covalent bond with the lone pair electrons of the nitrogen atom of N_2_H_4_ moiety. This is a reason to increase the length of the H1-N2 bond in MCr2 in comparison with the corresponding bond in isolated hydrazine. The covalent interaction in MCr2 caused the HOMO $\alpha$ and $\beta$ orbitals (in unrestricted formalism) to have higher energy than the HOMO orbitals in MCr1a and MCr1b. In the MCr2 complex, the difference between the absolute energies of the LUMO $\alpha$ and $\beta$ orbitals is large. So, it is unstable complex in the doublet state. In MCr2, the covalent nature of the newly formed bond (O1–N2) with a length of 1.389 Å is confirmed by AIM analysis [*ρ* (r_bcp_) = 0.3454 e bohr^−3^ and ${\nabla }^{2}\rho$ (r_bcp_) = − 3.8432 e bohr^−5^]. Also, an NBO analysis confirms that the O1–N2 bond in MCr2 is a sigma (σ) type with covalent nature. Also, the contributions of the involved orbitals in σ (O1–N2) bond are as follows:

$$\left( {{\text{BD}}\left( {{\text{N2}} - {\text{O6}}} \right)} \right) = {\text{ }}0.{\text{6883 }}\left( {{\text{sp}}^{{{\text{3}}.{\text{51}}}} } \right){\text{ N2 }} + {\text{ }}0.{\text{7254 }}\left( {{\text{sp}}^{{{\text{4}}.{\text{44}}}} } \right){\text{ O6}}{\text{.}}$$

In MCr1a and MCr1b, the hydrazine fragment is a hydrogen bond acceptor and the OH fragment is a hydrogen bond donor. The AIM parameters of the N2…OH interaction in MCr1a are *ρ* (r_bcp_) = 0.0366 e bohr^−3^ and ${\nabla }^{2}\rho$ (_bcp_) = 0.0732 e bBohr^5^ that confirms the existence of a hydrogen bond. In this complex, the amounts of the standard enthalpy and Gibbs free energy at the CCSD(T)/CBS + MP2/aTZ level are − 6.36 and 1.31 kcal mol^−1^, respectively, in comparison with the original reactants at room temperature. For MCr2, the mentioned thermodynamic parameters are 43.65 and 53.44 kcal mol^−1^, respectively.

Firstly, in the following sections, the H abstraction (barrier-less) reaction channels will be discussed. Secondly, hydroxyl radical addition to the N site of hydrazine will be investigated. Finally, the S_N_2 reaction (OH replacement with a hydrogen atom of hydrazine) will be argued.

### N_2_H_3_ + H_2_O formation channels

The H abstraction channels start with MCr1a and MCr1b complexes. The relative energy of MCr1a is − 7.88 kcal mol^−1^ at the CCSD(T)/CBS level. The H abstraction reactions are summarized as follows:

1. N_2_H_4_ + OH → MCr1a → TS2 → MCp1 → P1(H_2_NNH + H_2_O) (Path 1)
2. N_2_H_4_ + OH → MCr1b → TS3 → MCp2 → P1(H_2_NNH + H_2_O) (Path 2)

These paths were studied by Tang and et al. at the CCSD(T)/6-311 +  + G(d,p)//B3LYP/6-311G(d,p) + ZPE level, and the reported relative energies for TS2 and TS3 were − 1.67 and 0.41 kcal mol^−1^, respectively.

We recomputed their suggested pathways at the same level and geometries. Our results show that the relative energies with including ZPE (and without including ZPE) for TS2 and TS3 are 0.38 (− 0.12) and 2.65 (1.58) kcal mol^−1^ (compared with − 1.67 and 0.43 kcal mol^−1^), respectively. The discrepancy between our results and Tang et al.'s work may be related to the optimized geometry of the N_2_H_4_ molecule at the B3LYP/6-311G(d,p) level. In Tang et al.'s study, the geometrical parameters of the N_2_H_4_ molecule were 1.487 Å for the N–N bond and 1.020 Å for the N–H bond, but our computed lengths are 1.435 Å and 1.018 Å for that bonds, respectively. For other species, the recomputed geometrical parameters at the mentioned level are the same as Tang et al. The T1 diagnostic values for some species calculated at the CCSD(T) method with different basis sets are in the range of 0.025–0.038. These values show that the higher-level calculations like the CCSD(T)/CBS//MP2/aTZ are important for a more accurate prediction of the energies and geometries of all stationary points. Therefore, the CCSD(T)/6-311 +  + G(d,p)//B3LYP/6-311G(d,p) level is inadequate due to having a small basis set to describe the mechanism and PES of the title reaction.

Based on the following results, for the H-abstraction saddle points, two different formalisms have different structures. In the DFT methods such as the B3LYP and M06-2X using several basis sets, the obtained structures are similar to the reactants, which are named early transition states. The ab initio methods such as the CASSCF and MP2 show that the structures of TS2 and TS3 are similar to neither reactants nor products. These transition states are called case transition states. For these reasons, we investigate the barrier heights and the rate constants using both formalisms. Using the ab initio methods, the MEP calculations show that TS2 and TS3 connect to MCr1a. The H abstraction reactions in paths 1 and 2 occur via TS2 and TS3, respectively. The IRC calculations verify that the formation of the complex products MCp1 and MCp2 is possible in the gas phase. The relative energies of MCp1 and MCp2 are − 44.45 and − 44.26 kcal mol^−1^ at the CCSD(T)/CBS level, respectively. The barrier energies (and relative energies) of TS2 and TS3 at the CCSD(T)/CBS level are 4.80 (− 3.08) and 5.49 (− 2.39) kcal mol^−1^. Also, the computed barrier energies and relative energies of TS2 and TS3 at different methods and basis sets are listed in Supplementary Table S5. The results show that the transition state structures and energies are sensitive to the applied methods and basis sets. It should be noted that the bond length and features of the OH and the shifting hydrogen of hydrazine in TS2 and TS3 are crucial. In MCr1a, the atoms H7 and O6 with a length of 2.76 Å have no interaction with each other. The corresponding bond length (H7–O6 = 1.464 Å) in TS2 is shorter than MCr1a. Also, the NBO outputs show that the mentioned bond in MCp1 and MCp2 has covalent nature as

$${\text{MCp1 }}\left( {\sigma {\text{ BD }}\left( {{\text{H7}} - {\text{O6}}} \right)} \right){\text{ }} = {\text{ }}0.{\text{8712 }}\left( {{\text{sp}}^{{{\text{2}}.{\text{72}}}} } \right){\text{ O6 }} + {\text{ }}0.{\text{49}}0{\text{9 }}\left( {{\text{s}}^{{0.{\text{99}}}} } \right){\text{ H7}}{\text{.}}$$

In MCp1, the N–N bond length is decreased compared with that bond in MCr1a. This change causes to make a bond with the $\pi$ character in nature and agrees with the following atomic hybridization:

$${\text{MCp1 }}({\text{BD }}\left( {{\text{N1}} - {\text{N2}}} \right)){\text{ }} = {\text{ }}0.{\text{67}}0{\text{2 }}\left( {{\text{sp}}^{{{\text{2}}.{\text{5}}0}} } \right){\text{ N }} + {\text{ }}0.{\text{7422 }}\left( {{\text{sp}}^{{{\text{1}}.{\text{83}}}} } \right){\text{ N2}}$$

(N1 occupies 44.91%, which involves 28.45% s orbital and 70.98% p orbital; N2 occupies 55.09%, which involves 35.19% s orbital and 64.44% p orbital.)

The electronic charge density of the ring critical point (RCP) for MCp1 and MCp2 is *ρ* (r_rcp_) = 0.0129, and 0.0128 e bohr^−3^, respectively. Also, the Laplacian of the charge density for that rings is ${\nabla }^{2}\rho$(r_rcp_) = 0.0706 and 0.0714 e bohr^−5^, respectively. The variation of the electronic charge density and its Laplacian confirms the hydrogen shift in path 1. Finally, the complex products, MCp1 and MCp2, by barrier-less processes release to the N_2_H_3_ and H_2_O adducts.

### H_2_NOH + NH_2_ formation channels

The H_2_NOH + NH_2_ generation pathways (S_N_2 and addition/elimination reactions) are summarized as follows:

3. N_2_H_4_ + OH → MCr1b → TS1 → MCp3a → P2(H_2_NOH + NH_2_) (Path 3)
4. N_2_H_4_ + OH → MCr2 → TS1b → MCp3b → P2(H_2_NOH + NH_2_) (Path 4)

Also, in the next section, we introduce another path for the production of P2 products. The third pathway begins with MCr1b pre-reactive complex, and the fourth pathway starts with MCr2. The substitution reaction between OH and NH_2_ fragments is an S_N_2 reaction similar to the organic reactions, occurring simultaneously. The NBO and MEP calculations confirm the S_N_2 substitution reaction. In this process, the hydroxyl radical is approaching the hydrazine molecule from behind, and the NH_2_ functional group is separating from the front side.

$${\text{TS1 }}\left( {{\text{BD }}\left( {{\text{O6}} - {\text{N2}}} \right)} \right){\text{ }} = ~0.{\text{6127 }}\left( {{\text{sp}}^{{{\text{12}}.{\text{35}}}} } \right){\text{ O6 }} + {\text{ }}0.{\text{79}}0{\text{3 }}\left( {{\text{sp}}^{{{\text{2}}.{\text{81}}}} } \right){\text{ N2}}{\text{.}}$$

This NBO analysis shows that the oxygen atom of hydroxyl moiety with sp^12.35^ hybrid orbital in TS1 has a weak covalent bond with non-bonding sp^2.81^ hybrid orbital of the atom N of hydrazine moiety. At the same time, the N–N bond is weaker than the corresponding bond in an isolated hydrazine molecule.

$${\text{TS1 }}\left( {{\text{BD }}\left( {{\text{N1}} - {\text{ N2}}} \right)} \right){\text{ }} = {\text{ }}0.{\text{4925 }}\left( {{\text{sp}}^{{{\text{13}}.{\text{32}}}} } \right){\text{ N1 }} + {\text{ }}0.{\text{87}}0{\text{3 }}\left( {{\text{s}}^{{{\text{2}}.{\text{27}}}} } \right){\text{ N2}}{\text{.}}$$

Tang et al. investigated also the substitution reaction between OH and NH_2_ moieties. They computed the electronic structure of this path at the CCSD(T)/6-311 +  + G(d,p)//B3LYP/6-311G(d,p) + ZPE level and showed that the OH radical group by surmounting a barrier height of 17.02 kcal mol^−1^ replaces to NH_2_ moiety. Our calculated relative energy at the CCSD(T)/6-311 +  + G(d,p)//B3LYP/6-311G(d,p) level with (and without) ZPE for that replacement is 21.52 (20.38) kcal mol^−1^. Also, Our computed barrier (and relative) energy for this substitution at the higher level, CCSD(T)/CBS, is 28.28 (20.41) kcal mol^−1^.

MCr2 is an energetic complex. So, it can be decomposed into several adducts. In this research, we investigate the only production of NH_2_OH + NH_2_ adducts. For this purpose, the relax scan option of Gaussian software was used. In MCr2, our calculated barrier energy for the N–N bond cleavage is 13.86 kcal mol^−1^ at the CCSD (T)/CBS level leading to produce the H_2_NOH + NH_2_ adducts by passing through TS1b.

### H_2_NNHOH + H formation channels

H_2_NNHOH + H production pathways (S_N_2 and addition/elimination reaction) are as follows:

5. N_2_H_4_ + OH → MCr1b → TS4 → MCp4 → P3(H_2_NNHOH + H) (Path 5)
6. N_2_H_4_ + OH → MCr1b → TS4 → MCp4 → TS1c → MCp3c → P2(H_2_NOH + NH_2_) (Path 6)

These pathways begin with MCr1b pre-reactive complex. The substitution reaction between H and OH is also the S_N_2 type. The replacement of hydroxyl radical with one hydrogen atom of hydrazine via TS4 happens by overcoming the barrier height of 51.05 kcal mol^−1^ at the CCSD(T)/CBS level. The IRC trajectory calculation confirms the production of MCp4 via TS4 through the S_N_2 process. The calculated high-pressure limit rate constant shows that the substitution reaction takes place in 700 cm^−1^. The imaginary frequency of TS4 is 2169 cm^−1^ at the UMP2x/aTZ level. The binding energy of the N–H bond in isolated hydrazine is 89.82 kcal mol^−1^ at the CCSD(T)/CBS level (and 88.41 kcal mol^−1^ at the UM06-2X/QZ level). So, the barrier energy of TS4 is lower than the binding energy. The binding energy of the N–H bond in hydrazine is the energy difference between two unstable radicals, N_2_H_3_ and H, and a stable hydrazine molecule. On one hand, when the hydrogen atom in TS4 leaves the nitrogen atom of the hydrazine fragment, the N_2_–H_3_ bond increases in comparison with the corresponding bond in isolated hydrazine. On the other hand, in the TS4 structure, the hydroxyl radical is approaching the nitrogen atom from behind and forms a weak covalent bond. So, the N–H covalent bond converts to weak covalent interaction. These interactions are the origin of stability for TS4 fragments compared to N_2_H_3_ and H free radicals. The NBO and AIM analyses show a weak covalent bond among the non-pair electrons of the atom N_2_ and hydroxyl radical. Also, the same interaction is observed between the atoms H_3_ and N_2_ in TS4 (similar to S_N_2 substitution reaction):

$${\text{TS4 }}\left( {{\text{BD }}\left( {{\text{N2 }} - {\text{ H3}}} \right)} \right){\text{ }} = {\text{ }}0.{\text{9142 }}\left( {{\text{sp}}^{{{\text{2}}.{\text{6}}0}} } \right){\text{ N2 }} + {\text{ }}0.{\text{4}}0{\text{54 }}\left( {{\text{s}}^{{0.{\text{99}}}} } \right){\text{ H3,}}$$

$${\text{TS4 }}\left( {{\text{BD }}\left( {{\text{N2 }} - {\text{ O6}}} \right)} \right){\text{ }} = {\text{ }}0.{\text{7428 }}\left( {{\text{sp}}^{{{\text{3}}.00}} } \right){\text{ N2 }} + {\text{ }}0.{\text{6695 }}\left( {{\text{sp}}^{{{\text{8}}.{\text{59}}}} } \right){\text{ O6}}{\text{.}}$$

An NBO analysis of TS4 shows that the sp^3.00^ hybrid orbital of the atom N2 has a weak covalent interaction with the sp^8.59^ hybrid orbital of the atom O6. The sp^3.00^ hybrid orbital of the atom N2 is varied to sp^2.60^. It shows that the p orbital contribution is decreased in the N–H bond during the S_N_2 process. The instability of MCp4 is related to the separated hydrogen atom.

The last path is another route for the production of the NH_2_OH + NH_2_ adducts. After MCp4 generation, MCP3b post reactive complex is created through TS1c by surmounting the barrier height of 59.91 kcal mol^−1^. This path is long, so kinetically it has less importance compared to the other pathways discussed above. In general, it should be pointed out that the paths 4–6 have a small contribution to hydrazine degradation due to having high energy barriers compared to the H abstraction reactions.

### Bond dissociation energy and the energy barrier of a saddle point

Calculating the bond dissociation energy (BDE) for the breaking bond and enthalpy of formation for the forming bond along with the distances of that bonds could help to understand the origin of the low or high value of the energy barrier (or relative energy) of a transition state. Different values for the BDE of the N–H and N–N bonds of hydrazine are listed in Table 3. The BDE of the N–H bond in isolated hydrazine is 82.64 kcal mol^−1^ at the CCSD(T)/CBS + MP2/aTZ level. Two different values have been reported for the BDE of the N–H bond in the literature. The first is around 76(± 2) kcal mol^−1^^61–63^ and the second is 87.5 kcal mol^−1^^64^. Our computed value is 6.64 kcal mol^−1^ higher than the prior value and is − 4.86 kcal mol^−1^ lower than the last. The differences may relate to the presence of atomic hydrogen that is very unstable and its control in homolytic bond dissociation is difficult experimentally. For the N–N bond of hydrazine, the experimental BDEs are in the range of 57.1–65.96 kcal mol^−1^^61,63,65–68^ (see Table 3). Also, the different theoretical methods have different predictions for the BDE of the N–N bond. The obtained values are in the range of 51.62–66.92 kcal mol^−1^^69–72^. Our used computational level is higher than all previous studies, the CCSD(T)/CBS + MP2/aTZ level. The obtained result (65.93 kcal mol^−1^) is close to the upper limit of the experimental results, but it is 1.29 kcal mol^−1^ lower than the upper limit of the theoretical results. The enthalpy of formation for N–O bond in H_2_NOH is − 64.03 kcal mol^−1^. In TS1, the N–N bond is breaking and the N–O bond is forming. So, the BDE is 1.91 kcal mol^−1^ higher than the absolute value of the enthalpy of formation. On the other hand, the N…N bond distance (1.719 Å) is about 0.12 Å higher than the N…O bond in TS1. Thus, a larger amount of BDE for the N…N bond accompanied by larger distances in TS1 (leading to weak interactions) is the origin of a high value for the energy barrier of TS1. TS1b involves just a bond breaking, the N…N bond, and no bond is forming, lacking enthalpy of formation and existing only one center to interaction. Therefore, it has a higher value than the others. In TS1c, the N…N bond is breaking and the N…H bond is forming, but the absolute value of enthalpy of formation of the N–H bond is higher than the BDE of the N–N bond. It is expected to have lower barrier energy. But, it should be noted that atomic hydrogen is seen in this transition state which has a weak van der Waals interaction with nitrogen atoms. So, hydrogen atom unstability is dominant. In TS4, the same argument is valid, but the leaving hydrogen atom is 0.08 Å closer than the attaching hydrogen atom of TS1c to the nitrogen atom, having stronger interaction. Therefore, TS4 has lower relative energy than TS1c.

**Table 3:** The computed BDEs of the N–N and N–H bonds through homolytic dissociation in the N_2_H_4_ molecule.

Bond	BDE
N–N	76.00 ± 5.00^61^
	76.00^62^
	76.00 ± 20^63^
	87.50^64^
	82.64 (this work-CCSD(T)/CBS//I)
	82.45 (this work-CCSD(T)/CBS//II)
	81.04 (this work-M06-2X/aQZ)
	83.26 (this work-MP2/aTZ)
N–N	57.10^67^
	58.00 ± 9.00^61^
	59.00 ± 3.00^63^
	60.00 ± 3.00^65^
	62.00^66^
	51.62- 64.29^69^^a^
	54.60 ± 5.30^71^^b^
	62.77^70^^c^
	65.96 ± 1.9^68^
	66.92^72^^d^
	65.93 (this work-CCSD(T)/CBS//I)
	65.64 (this work-CCSD(T)/CBS/II)
	67.97 (this work-M06-2X/aQZ)
	70.21 (this work-MP2/aTZ)

I: refers to the correction values at MP2/aTZ level.

II: refers to the correction values at M06-2X/aQZ level.

^a^Calculated by 7 methods.

^b^Calculated for 11 hydrazine moieties.

^c^The average value of eight computational methods.

^d^Computed by the G2MP2 method.

About the saddle points of barrier-less reactions, TS2 and TS3, it should be noted that as mentioned above, the N–H bond dissociation is 82.64 kcal mol^−1^ and $\Delta H_{f}^{0} ({\text{H}} - {\text{O}})$ = − 119.14 kcal mol^−1^. So, the H-shifts through TS2 and TS3 occur by barrierless processes. The difference between TS2 and TS3 energies is related to the OH orientation and the bond distances of the N…H bonds. In these saddle points, shifting hydrogen atom has a strong interaction with nitrogen atom and a hydrogen bond interaction with the oxygen of hydroxyl radical.

Overall, it can be concluded that if a bond has a high value for BDE than the $\Delta H_{f}^{0}$*,* and also a weak strength in a transition state the high value for barrier energy is expected, which will be higher if there is atomic hydrogen with weak interactions, and for barrierless reactions, it is expected that $\Delta H_{f}^{0}$ > BDE along with strong interactions.

### The high-pressure limit rate constant

The temperature dependence of rate constants for all elementary reactions is obtained via both variational transition state theory (VTST) and transition state theory (TST) for bimolecular reactions. Then, the rate constants are improved by Shavit tunneling correction. To achieve more reliable rate constants, higher-level calculations are performed such as the CCSD(T)/CBS//MP2/aTZ, CCSD(T)/aQZ//MP2/aTZ, CCSD(T)/aTZ//MP2/aTZ, and validated DFT-M06-2X/maTZ levels. Our calculated high-pressure limit rate constants in different levels in the temperature range of 230–3000 K are collected in Supplementary Tables S7–S30 (see Supplementary information). From a kinetic point of view, the rate constants for one-step elementary reactions are more important. So, in this section, we consider the production pathways of N_2_H_3_ + OH, NH_2_OH + NH_2_, and H_2_N_2_HOH + H products that need to pass just one transition state.

**Figure 3:**
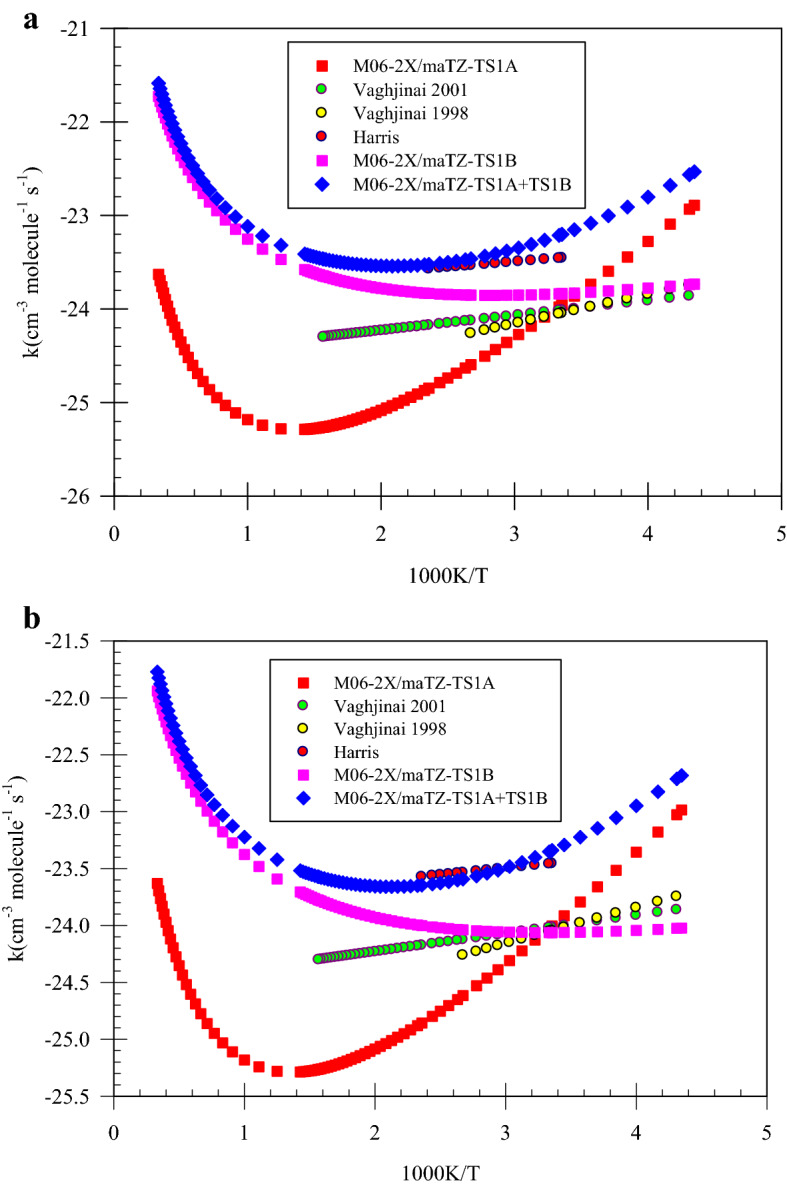
Representation of Arrhenius plot for P1 production pathways computed at the M06-2X/maTZ level : (**a**) TST theory  (**b**)   VTST theory

As explained above, the data used by Tang et al.^25^ for rate constant calculations are inconsistent with the results obtained using the applied methods. Thus, the rate constants of the maim channel pathways are calculated by the corrected energies. So, the rate constants for P1 and P2 generation pathways recalculated at the CCSD(T)/6-311 +  + G(d,p)//B3LYP/6-311G(d,p) level (energies are obtained at the CCSD(T)/6-311 +  + G(d,p)//B3LYP/6-311G(d,p) computational level and partition functions are taken at the B3LYP/6-311G(d,p) level). Theoretical rate constants obtained at this level using TST and VTST theories have large differences from observed rate constants experimentally by different groups, which are referred to below. Obtained rate constants at this level are collected in Supplementary Tables S9–S11. The Shavit tunneling factor is considered for correcting high-pressure limit rate constants. The obtained rate constants of P1 adducts through the pathways 1 and 2 by TST theory at the UM06-2X/maTZ level in temperatures of 298.15, 400, and 600 K are 8.38 × 10^–11^, 6.21 × 10^–11^, and 6.27 × 10^–11^ cm^−3^ molecule^−1^ s^−1^, respectively. The mentioned rates using VTST theory are 7.31 × 10^–11^, 5.49 × 10^–11^, and 5.62 × 10^–11^ cm^−3^ molecule^−1^ s^−1^, respectively. At a high-level ab initio computation, the CCSD(T)/aTZ//MP2/aTZ level, the rate constants of the considered adducts by using TST theory at the same temperatures are 2.11 × 10^–10^, 7.26 × 10^–11^, and 3.34 × 10^–11^ cm^−3^ molecule^−1^ s^−1^, respectively. Also, the obtained results using the VTST theory are 7.10 × 10^–11^, 3.72 × 10^–11^, and 2.52 × 10^–11^ cm^−3^ molecule^−1^ s^−1^, respectively. Our computed rate constants using TST and VTST theories at the UM06-2X/maTZ level have a good agreement with the experimentally reported rate constant by Vaghjiani^21^ ((6.50 ± 1.3) × 10^–11^ cm^3^ molecule^−1^ s^−1^), which has independent temperature behavior. Also, our results have a good agreement with Harris et al.^23^ results. Their reported rate constants using the photolysis-resonance fluorescence technique at temperatures of 298.15 and 400 K were 6.69 × 10^–11^ and 5.84 × 10^–11^ cm^3^ molecule^−1^ s^−1^, respectively. Our calculated rate constants at room temperature using TST and VTST theories at the CCSD(T)/aTZ//MP2/maTZ level are in excellent agreement with the experimental value (3.60 × 10^–11^ cm^3^ molecule^−1^ s^−1^) reported by Vaghjiani^22^. Also, a good agreement is observed for overall rate constants of title reaction at room temperature by Hack et al.^24^ (2.16 × 10^–11^ cm^3^ molecule^−1^ s^−1^). The graphs sketched for the obtained rate constants by experiment and theory are brought in Fig. 3a,b, respectively, for P1 adducts.

The rate constants of P2 adducts through the paths 3, 4, and 6 using TST theory at the UM06-2X/maTZ level in temperatures of 298.15, 400, 600, 1000, and 3000 K are 1.48 × 10^–29^, 2.30 × 10^–25^, 3.77 × 10^–21^, 1.45 × 10^–17^, and 2.60 × 10^–13^ cm^−3^ molecule^−1^ s^−1^, respectively. The rate constants for P3 adducts at the mentioned temperatures are 1.96 × 10^–43^, 8.72 × 10^–36^, 3.27 × 10^–28^, 6.57 × 10^–22^, and 8.38 × 10^–15^ cm^−3^ molecule^−1^ s^−1^, respectively. The rate constants for these adducts at several levels are computed and listed in Supplementary Tables S11, S16–S20, and S26–S29 in the Supplementary data. In summary, comparing the rate constant reported here with different experimental results shows that our used methods have adequately precise in describing the title reaction kinetics.

### The low-pressure limit rate constant and its behavior in the falloff regime

To investigate the pressure-dependent rate constant, the strong collision master equation Rice–Ramsperger–Kassel–Marcus (RRKM) theory is used. The rate constant of the title reaction in the low-pressure limit and its behavior in the falloff range is investigated in the 200–800 K temperature range. The chemical activation mechanism is implemented in the pressure-dependent rate constant as follows:

4 $$N_{2} H_{4} + {\text{ }}OH~\xrightarrow{{K(T)}}Cr,$$

5 $$Cr{\text{ }} + {\text{ }}M~\xrightarrow{{k_{1} }}Cr*{\text{ }} + {\text{ }}M,$$

6 $$Cr*{\text{ }} + {\text{ }}M~\xrightarrow{{k_{{ - 1}} }}Cr{\text{ }} + {\text{ }}M,$$

7 $$Cr*~\xrightarrow{{k_{2} }}N_{2} H_{3} + {\text{ }}H_{2} O,$$

where M is the third body. If we apply the steady-state approximation to the concentration of Cr*, the rate of conversion of Cr to final adducts is

8 $$k_{i} (T,p) = \frac{{k_{1} k_{2} [M]}}{{k_{{ - 1}} [M] + k_{2} }}.$$

In the high-pressure limit ([M] → ∞), k(T,p) is the first order and in the low-pressure limit (where [M] → 0) k(T,p) is the second order. Therefore, in the high and low-pressures, k(T,p) expressions in Eq. (8) can be written as follows:

9 $$k_{\infty } = \frac{{k_{1} k_{2} }}{{k_{{ - 1}} }}\quad \left( {\left[ {\text{M}} \right]{\text{ }} \to {\text{ }}\infty } \right),$$

and

10 $$k_{0} = k_{1} [M]\quad \left( {\left[ {\text{M}} \right]{\text{ }} \to {\text{ }}0} \right).$$

If we divide the numerator and the denominator of Eq. (8) into k_−1_ [M] and substitute Eqs. (9) and (10) in it, we have:

11 $$k_{i} (T,p) = \frac{{k_{\infty } }}{{1 + \frac{{k_{\infty } }}{{k_{0} }}}}.$$

Finally, for the calculation of pressure dependence of the rate constant, the following relation is used:

12 $$k(T,p) = \kappa K(T)k_{i} (T,p),$$

where $$\kappa$$ is tunneling correction, and *K*(*T*) is the temperature dependent equilibrium constant. Similar to temperature dependent rate constants, the Shavitt transmission coefficient was used for the correction of pressure-dependent rate constants. Because the nitrogen molecule is the most abundant species relative to other molecules in the atmosphere, it is used as the third body in the calculation of k(T,p). In the investigation of pressure effect on the temperature dependent rate constant by the master equation, the Lenard Jones parameters, σ (Å), and $${\raise0.7ex\hbox{$\varepsilon $} \!\mathord{\left/ {\vphantom {\varepsilon {k_{B} }}}\right.\kern-\nulldelimiterspace} \!\lower0.7ex\hbox{${k_{B} }$}}$$(K), and also the average amount of energy transferred per collision are important. The Lenard–Jones parameters of the reactants and bath gas are 4.230 Å and 250.000 K for N_2_H_4_, 2.750 Å, and 80.000 K for OH, and 3.798 Å and 71.400 K for N_2_^73^.

In the zero-pressure limit, P → 0, k(T,p)/[N_2_] is called the low-pressure limit rate constant k_0_(T). k_0_(T) is a termolecular rate constant with the units of cm^6^ molecule^−2^ s^−1^ and is also called pseudo-third order rate constant. Our calculated rate constants at the low-pressure limit in the temperature range of 200–800 K are listed in Supplementary Table S31. The results show that k_0_(T) is 7.87 × 10^–32^, 2.8 × 10^–32^, and 1.09 × 10^–32^ cm^−6^ molecule^−2^ s^−1^ at 298.15, 400 and 600 K, respectively. To our knowledge, the rate constant is not determined experimentally at the low-pressure limit for the generation of main reaction products.

**Figure 4:**
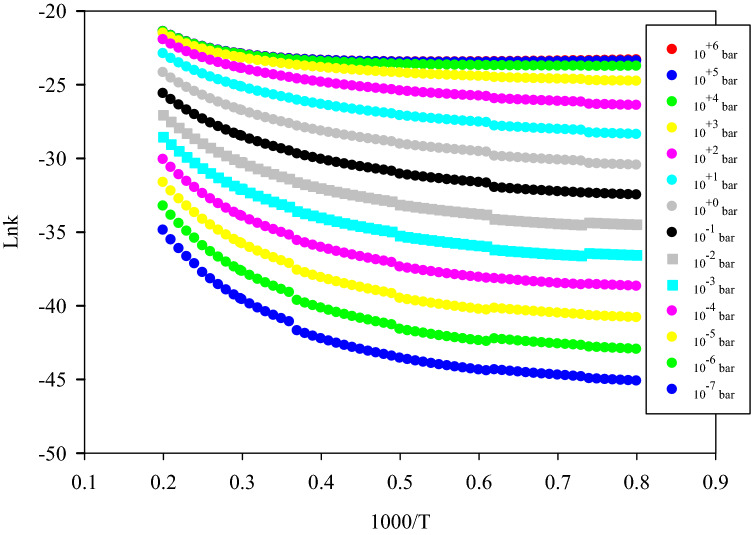
Representation of temperature and pressure-dependent rate constant of P1 production pathways (path 1 + path 2) of the N_2_H_4_ + OH reaction.

Our calculated rate constants at different pressures in the falloff regime in the temperature range of 200–800 K are depicted in Fig. 4 and collected in Supplementary Table S31. By using Eq. (11), we concluded that the ratio of k_∞_/k_0_ is significant in the calculation of k(T,p). The clear results relating to the effect of pressure on the main reaction pathways are seen when the pressure increases in the 200–800 K temperature range. In 298.15, 400, and 600 K, the ratio of k_∞_/k_0_ is 38.8, 196.0, and 513.0, respectively, which shows this ratio increases over the considered temperature range.

Reducing the calculated rate constants at 298.15 K into the rate constants at 1 bar, make better insight into the effect of pressure on the rate of reaction at room temperature. The reduced values of k(T,p)/k(T,1 bar) for p = 10^–4^, 0.01, 100, and 10^4^ bar are 8.71 × 10^–4^, 2.96 × 10^–2^, 17.2, and 43.8 at 298.15 K, and 3.72 × 10^–4^, 2.05 × 10^–2^, 27.3, and 110 at 400 K, respectively. Our data show that the rate constant increases with increasing pressure in the 200–800 K temperature range.

### Kinetic and thermodynamic viewpoints

Enthalpies and Gibbs free energies of all products calculated at the M06-2X/aQZ and MP2/aTZ levels in the temperature range of 200–1200 K are tabulated in Table 4 and Supplementary Table S31. The results show that among all the obtained products, P1 adducts are the most stable. Moreover, P2 adducts are more stable than P3. Based on the obtained results, the amount of stability of P1 products increases with the temperature that is going from − 37.36 in 200 K to − 41.45 kcal mol^−1^ in 1200 K.

**Table 4:** The thermodynamic parameters (kcal mol^−1^) of the P1–P3 adducts of the N_2_H_4_ + OH reaction on the doublet potential energy surface at the CCSD(T)/CBS (energy) + UMP2/aTZ (corrections) level.

T/K	${\Delta E}_{T}^{0}$	${\Delta H}_{T}^{0}$	${\Delta G}_{T}^{0}$	${T\Delta S}_{T}^{0}$	T/K	${\Delta E}_{T}^{0}$	${\Delta H}_{T}^{0}$	${\Delta G}_{T}^{0}$	${T\Delta S}_{T}^{0}$
** R → P1(N**_**2**_**H**_**3**_** + H**_**2**_**O)**									
200	− 36.58	− 36.58	− 37.36	0.78	500	− 36.48	− 36.48	− 38.62	2.14
250	− 36.54	− 36.54	− 37.56	1.02	550	− 36.49	− 36.49	− 38.83	2.35
300	− 36.51	− 36.51	− 37.77	1.26	600	− 36.50	− 36.50	− 39.05	2.55
350	− 36.49	− 36.49	− 37.98	1.49	700	− 36.55	− 36.55	− 39.47	2.92
400	− 36.48	− 36.48	− 38.19	1.71	900	− 36.68	− 36.68	− 40.29	3.61
450	− 36.47	− 36.47	− 38.40	1.93	1200	− 36.94	− 36.94	− 41.45	4.51
** R → P2(NH**_**2**_**OH + NH**_**2**_**)**									
200	1.82	1.82	0.90	0.92	500	1.91	1.91	− 0.57	2.48
250	1.86	1.86	0.67	1.20	550	1.90	1.90	− 0.81	2.72
300	1.89	1.89	0.42	1.47	600	1.89	1.89	− 1.06	2.95
350	1.91	1.91	0.18	1.73	700	1.68	1.69	− 1.61	3.29
400	1.92	1.92	− 0.07	1.99	900	1.74	1.74	− 2.51	4.25
450	1.92	1.92	− 0.32	2.24	1200	1.39	1.39	− 3.89	5.28
** R → P3(NH**_**2**_**NHOH + H)**									
200	28.85	28.85	30.68	− 1.83	500	29.35	29.35	33.24	− 3.89
250	28.88	28.88	31.13	− 2.26	550	29.49	29.49	33.63	− 4.14
300	28.93	28.93	31.58	− 2.65	600	29.63	29.63	34.00	− 4.36
350	27.97	29.01	32.01	− 3.00	700	29.71	29.71	34.63	− 4.93
400	29.11	29.11	32.43	− 3.32	900	30.37	30.37	35.96	− 5.59
450	29.22	29.22	32.85	− 3.62	1200	31.37	31.37	37.66	− 6.29

According to the origin of Gibbs free energy and Table 4 information, the stability of P1 is related to the entropy of reaction that increases with temperature. The variation of *TΔS°* for P1 in the temperature range 200–1200 K equals 0.78–4.51 kcal mol^−1^. The same behavior for the stability of P2 adducts is observed, but it is changed inversely for P3 adducts that is related to their electronic entropy. With increasing temperature from 200 to 1200 K, the amount of *TΔS°* for these species decreases from − 1.83 to − 6.29 kcal mol^−1^. Another factor for the instability of P3 adducts is the enthalpy function by total variation about + 2.52 kcal mol^−1^ that is related to the instability of the atom H. For P1 and P2 adducts, the variation of enthalpy is low in the mentioned temperature range.

In summary, with increasing temperature, the P1 and P2 generations are more favorable not only thermodynamically but also kinetically. The production of P3 adducts is favorable just kinetically at a temperature above 1000 K.

### The fate of hydrazine in the atmosphere

The lifetimes of hydrazine in the atmosphere at altitudes from 0 to 50 km (corresponding to pressures from 1013 to 0.801 mbar) are listed in Table 5, Supplementary Tables S7, and S8. The results show the H abstraction process is a key step for hydrazine degradation in the atmosphere. The computed rate constant decreases with increasing altitude from 2.83 × 10^–12^ to 3.52 × 10^–14^ cm^3^ molecule^−1^ s^−1^. Therefore, the calculated atmospheric lifetime of hydrazine degradation in the environment of hydroxyl radical varies from 32.80 to 1161.11 h.

**Table 5:** The rate constant of the N_2_H_4_ + OH reaction for Path 1 + Path 2, the concentration of hydroxyl radical in different altitudes, and lifetimes of N_2_H_4_ in the atmosphere in an ambient of atmospheric hydroxyl radical as functions of Height.

*H*/km	*T*/K	*P*/mbar	*k*^a^	[OH]/molecule cm^−3^	*τ*^b^/s	$\frac{{k}_{\infty }}{k}$
0	290.2	1013	2.83E−12	3.00E + 06	1.18E + 05	4.14E + 01
5	250.2	495.9	4.03E−12	1.00E + 06	2.48E + 05	4.46E + 01
10	215.6	242.8	7.17E−12	5.70E + 05	2.45E + 05	3.70E + 01
15	198.0	118.8	9.12E−12	4.20E + 05	2.61E + 05	7.28E + 01
20	208.0	58.18	3.80E−12	3.70E + 05	7.11E + 05	1.27E + 02
25	216.1	28.48	1.73E−12	6.60E + 05	8.78E + 05	1.91E + 02
30	221.5	13.94	8.66E−13	1.60E + 06	7.22E + 05	3.42E + 02
35	228.1	6.826	4.13E−13	3.70E + 06	6.54E + 05	6.28E + 02
40	240.5	3.341	1.60E−13	6.80E + 06	9.21E + 05	1.31E + 03
45	251.9	1.636	6.34E−14	8.50E + 06	1.86E + 06	2.78E + 03
50	253.7	0.801	3.52E−14	6.80E + 06	4.18E + 06	4.90E + 03

The altitude (H), pressure (P), the temperature (T), and the OH concentration ([OH]) in this Table are from reference^75^.

^a^k is the bimolecular rate constant at the mentioned temperature and pressure.

^b^τ = $\frac{1}{k[OH]}$ is the lifetime of N_2_H_4_ in the atmospheric concentration of OH.

The tropospheric half-life of hydrazine in the reaction with hydroxyl radical was reported ~ 3 h by Harris et al*.*^23^. By assuming roughly 1.1 × 10^6^ molecule cm^−3^ for OH concentration, Vaghjiani^22^ estimated that the fate of N_2_H_4_ is 6.6 h at an average ambient temperature of 279 K. A difference is observed between our calculated lifetime with the results obtained by Vaghjiani and Harris et al.experimentally. They have used the high-pressure limit rate constant instead of the pressure-dependent rate constant to calculate the mentioned half-life. Thus, If we use the obtained high-pressure limit rate constant by Hack^24^ (2 × 10^–11^ cm^3^ molecule^−1^ s^−1^) at 298 K with the OH concentration reported by Vaghjiani, the lifetime of N_2_H_4_ will be 12.6 h. Our computed overall high-pressure limit rate constant for H abstraction channels is 8.39 × 10^–11^ cm^3^ molecule^−1^ s^−1^ at room temperature. If we use this value with the mentioned concentration for OH, the lifetime of hydrazine is 3.01 h. Tuazon et al. have estimated an upper limit for the corresponding lifetime in the N_2_H_4_ + O_3_ reaction. Their reported rate coefficient at 298 K is 1.4 × 10^–16^ cm^3^ molecu1e^−1^ s^−1^. The O_3_ concentration used by Tuazon et al. was about 5 $\times$ 10^11^ molecule cm^−3^ or less. Therefore, the obtained lifetime for N_2_H_4_ degradation was about 2.6 h or longer^74^. As we know, the concentration of O_3_ in the ozone layer is larger than the other parts of the atmosphere, and O_3_ concentration is also less than the concentration of OH radicals in the urban area. Therefore, atmospheric hydroxyl radicals are an important factor for hydrazine degradation in urban areas.

## Conclusion

The kinetics and mechanisms of the N_2_H_4_ + OH reaction, including reliable paths, are investigated by accurate quantum chemical methods. The results obtained by single reference methods such as B3LYP, M06-2X, MP2, and CCSD(T) in conjunction with augmented triple zeta basis sets (6-311 + + g(3df,3pd) and aTZ) show that there are small differences among the computed relative energies of all stationary points in the mentioned methods. The relative energies of the multi-reference method (MR-MP2) are different compared with the single-reference methods, which correspond to select of a small active space for the N_2_H_4_ + OH case. Therefore, the sensitivity of the doublet PES of the title reaction for the applied methods and basis sets is negligible. The rate constant calculations show that the sum of the first and second paths rate constants have a negative temperature dependence behavior at low temperatures until 540 K at the M06-2X/maTZ level, and have a positive temperature dependence behavior in the range of 560–3000 K. Our computed high-pressure limit rate constants using two formalisms, TST and VTST theories, show the results of the DFT-M06-2X/maTZ level at low temperatures are roughly temperature independent, which have excellent agreement with the experimental results (see Fig. 3a,b). Also, the same results computed by the CCSD(T) method along with several basis sets (on the optimized structures of the MP2/aTZ level) at low temperatures depend on temperature. By applying pressure on the P1 generation pathways, we demonstrated that the reaction rate has a positive pressure dependence behavior. And, the variation of the rate constant with pressure is inversely related to temperature. Thermodynamic parameters of all suggested adducts are reported and discussed at the CCSD(T)/CBS (energy) + UMP2/aTZ (corrections), CCSD(T)/CBS (energy) + M06-2X/aQZ (corrections), M06-2X/aQZ and MP2/aTZ levels. Some of the predicted adducts can be produced in the atmosphere with a wave number greater than 700 cm^−1^.

## Supplementary Information

Supplementary Information.
