# Supplementary material for: Atmospheric reaction of hydrazine plus hydroxyl radical
Source: Sci Rep. 2021 Jun 24;11:13220. doi: 10.1038/s41598-021-92563-8 (PMC8225856; doi:10.1038/s41598-021-92563-8)
Supplement: Supplementary file 1 — Supplementary Information. [file 41598_2021_92563_MOESM1_ESM.docx]

**Atmospheric reaction of hydrazine plus hydroxyl radical**

Hamed Douroudgari^*^, Morteza Vahedpour^*^, Fahime Khouini

Department of Chemistry, University of Zanjan, PO Box 38791-45371, Zanjan, Iran

E-mail:  [douroudgari@znu.ac.ir](mailto:%20douroudgari@znu.ac.ir)

E-mail:  [vahed@znu.ac.ir](mailto:%20vahed@znu.ac.ir)

**Figure S1.** Atomic labels of all species.

**Table S1**. The selected important bond lengths (in Å) for TS1, TS1b and TS1c at different methods.

**Table S2**. The selected important bond lengths (in Å) for TS4 at different methods.

**Table S3**. Unscaled vibrational frequencies (in cm^-1^) of stationary points of reliable paths calculated at at different levels.

**Table S4**. The electronic ($E_{elec}$) and zero point (ZPE) energies (in Hartree) of all species at different levels.

**Table S5**. The relative energies (ΔΕ), forward barrier heights (${\Delta E}_{f}^{\neq}$), reverse barrier heights (${\Delta E}_{r}^{\neq}$), and reaction energies ($\Delta_{r}E$) of the N_2_H_4_ + OH reaction. (Units of all numbers are kcal mol^-1^)

**Table S6**. Recalculated electronic energies ($E_{elec}$) and zero point energies (ZPE) (in Hartree), and relative energies (ΔΕ), forward barrier heights (${\Delta E}_{f}^{\neq}$), and reverse barrier heights (${\Delta E}_{r}^{\neq}$) (in kcal mol^-1^) of the reaction of N_2_H_4_ + OH at the B3lyp/6-311G(d,p) and CCSD(T)/6-311++G(d,p)//B3lyp/6-311G(d,p) levels.

**Table S7**. The rate constant of the N_2_H_4_ + OH reaction for path1, the concentration of hydroxyl radical in different altitudes, and lifetimes of N_2_H_4_ in the atmosphere in an ambient of atmospheric hydroxyl radical as functions of height.

**Table S8**. The rate constant of the N_2_H_4_ + OH reaction for path 2, the concentration of hydroxyl radical in different altitudes, and lifetimes of N_2_H_4_ in the atmosphere in an ambient of atmospheric hydroxyl radical as functions of height.

**Table S9**. High pressure limit rate constants calculated at the CCSD(T)/6-311++G(d,p)//B3LYP/6-311G(d,p) levels for path1 and path 2.

**Table S10**. High pressure limit rate constants calculated at the CCSD(T)/6-311++G(d,p)//B3LYP/6-311G(d,p) levels for path 1 + path 2.

**Table S11**. High pressure limit rate constants calculated at the CCSD(T)/6-311++G(d,p)//B3LYP/6-311G(d,p) levels for path 3.

**Table S12**. The high-pressure limit rate constants of P1 generation pathways (path 1 + path 2) and Shavitt tunneling correction.

**Table S13**. High pressure limit rate constants calculated by TST theory at different levels for path 1.

**Table S14**. High pressure limit rate constants calculated by TST theory at different levels for path 2.

**Table S15**. High pressure limit rate constants calculated by TST theory at different levels for P1 adducts via path 1 + path 2.

**Table S16.** The high-pressure limit rate constants of P2 and P3 generation pathways and Shavit tunneling correction.

**Table S17**. High pressure limit rate constants calculated by TST theory at different levels for path 3.

**Table S18**. High pressure limit rate constants calculated by TST theory at different levels for path 4.

**Table S19**. High pressure limit rate constants calculated by TST theory at different levels for path 6.

**Table S20**. High pressure limit rate constants calculated by TST theory at different levels for P2 adducts via path 3 + path 4 + path 6.

**Table S21**. High pressure limit rate constants calculated by TST theory at different levels for path 5.

**Table S22**. High pressure limit rate constants calculated by VTST theory at the MP2/aTZ, M06-2X/maTZ, CCSD(T)/6-31++G(2df,pd)//MP2/aTZ, and CCSD(T)-full/6-311++G(df,2p)// MP2/aTZ levels for path 1.

**Table S23**. High pressure limit rate constants calculated by VTST theory at the CCSD(T)-full/6-311++G(2df,2p)//MP2/aTZ, CCSD(T)-full/6-311++G(3df,2p)//MP2/aTZ, CCSD(T)/TZ// MP2/aTZ, and CCSD(T)/aTZ//MP2/aTZ levels for path 1.

**Table S24**. High pressure limit rate constants calculated by VTST theory at the MP2/aTZ, M06-2X/maTZ, and CCSD(T)/6-31++G(2df, pd)//MP2/aTZ levels for path 2.

**Table S25**. High pressure limit rate constants calculated by VTST theory at the CCSD(T)-full/6-311++G(df, 2p)//MP2/aTZ, CCSD(T)-full/6-311++G(2df, 2p)//MP2/aTZ, CCSD(T)-full/6-311 ++G(3df, 2p)//MP2/aTZ, CCSD(T)/TZ//MP2/aTZ , and CCSD(T)/aTZ//MP2/aTZ levels for path 2.

**Table S26**. High pressure limit rate constants calculated by VTST theory at the MP2/aTZ, M06-2X/maTZ, and CCSD(T)/aTZ//MP2/aTZ levels for path 3.

**Table S27**. High pressure limit rate constants calculated by VTST theory at the MP2/aTZ, M06-2X/maTZ, and CCSD(T)/aTZ//MP2/aTZ levels for path 4.

**Table S28**. High pressure limit rate constants calculated by VTST theory at the MP2/aTZ, M06-2X/maTZ, and CCSD(T)/aTZ//MP2/aTZ levels for path 6.

**Table S29**. High pressure limit rate constants calculated by VTST theory at the MP2/aTZ, M06-2X/maTZ, and CCSD(T)/aTZ//MP2/aTZ levels for path 3 + path 4 + path 6.

**Table S30**. High pressure limit rate constants calculated by VTST theory at the MP2/aTZ, M06-2X/maTZ, and CCSD(T)/aTZ//MP2/aTZ levels for path 5.

**Table S31**. Pressure dependent rate constants calculated at the M06-2X/maTZ for P1 adducts via path 1 + path 2.

**Table S32**. Optimized Cartesian coordinates, X, Y, and Z (Å) other paths at different levels for N_2_H_4_ + OH reaction.

**Table S33.** The thermodynamic parameters (kcal/mol) of P1 – P3 adducts in the N_2_H_4_ + OH reaction at the MP2/aTZ Level.

**Table S34.** The thermodynamic parameters (kcal/mol) of P1-P3 adducts of N_2_H_4_+ OH reaction on the doublet potential energy surface at the UM06-2X/aug-cc-pVQZ level.

**Table S35.** The thermodynamic parameters (kcal/mol) of P1-P3 adducts of N_2_H_4_+ OH reaction on the doublet potential energy surface at the CCSD(T)/CBS (energy) + UMP2/aug-cc-pVTZ (corrections) level.

**Table S36.** T1 diagnostics and Largest amplitudes calculated at the CCSD(T)/aQZ and CCSD(T)/aTZ levels.


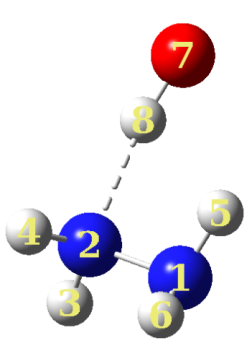

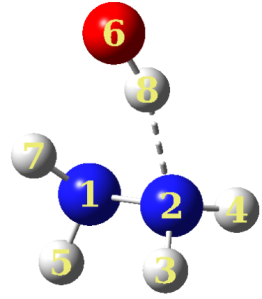

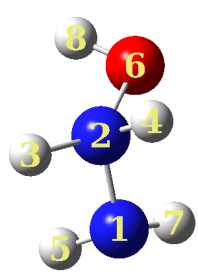

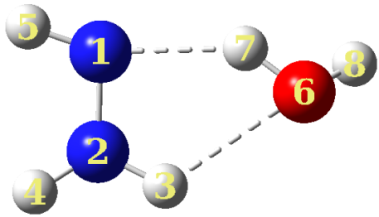


MCr1a MCr1b MCr2 MCp1


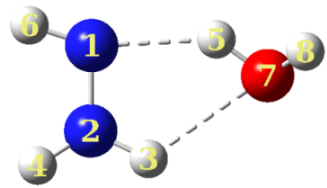

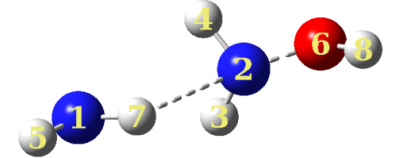

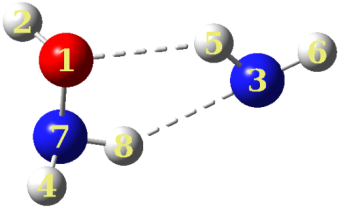

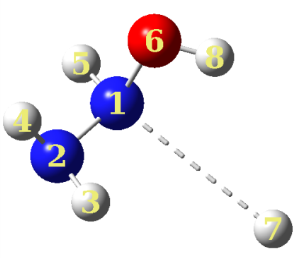


MCp2 MCp3a MCp3b MCp4


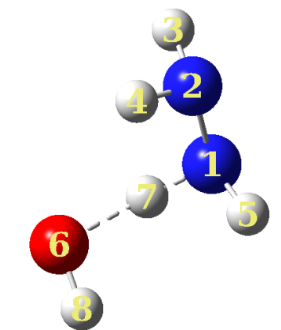

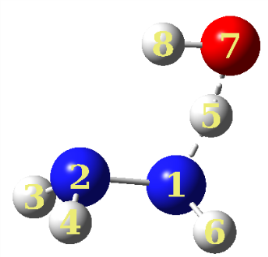

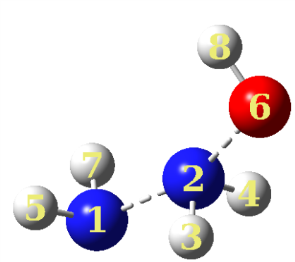

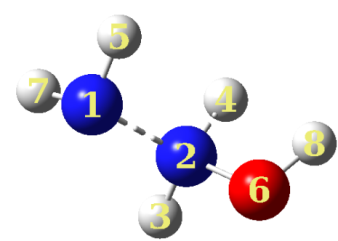


TS2 TS3 TS1 TS1b


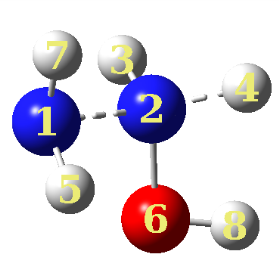

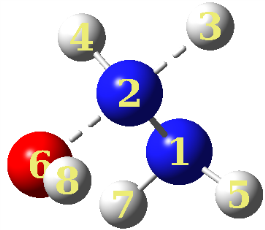


TS1c TS4

**Figure S1.** Atomic labels of all species.

|  | TS1 | | TS1b | TS1c | |
| --- | --- | --- | --- | --- | --- |
| Methods | N-N | N-O | N-N | N-N | N-H |
| MP2/aTZ | 1.719 | 1.602 | 1.697 | 1.608 | 1.175 |
| MP2/maTZ | 1.717 | 1.601 | - | 1.597 | 1.176 |
| MP2/6-311++g(3df,3pd) | 1.714 | 1.597 | 1.701 | 1.654 | 1.101 |
| M06-2X/aTZ | 1.805 | 1.661 | 1.710 | 1.629 | 1.266 |
| M06-2X /maTZ | 1.805 | 1.661 | 1.710 | 1.633 | 1.289 |
| M06-2X /6-311++g(3df,3pd) | 1.801 | 1.658 | 1.721 | 1.629 | 1.264 |
| M06-HF/aTZ | 1.780 | 1.643 | 1.712 | 1.652 | 1.099 |
| M06-HF/maTZ | 1.780 | 1.643 | 1.711 | 1.632 | 1.116 |
| M06-HF /6-311++g(3df,3pd) | 1.779 | 1.640 | 1.713 | 1.662 | 1.078 |
| B3LYP/aTZ | 1.854 | 1.690 | 1.669 | 1.603 | 1.165 |
| B3LYP/maTZ | 1.854 | 1.691 | - | 1.629 | 1.351 |
| B3LYP/6-311++g(3df,3pd) | 1.852 | 1.689 | 1.677 | 1.621 | 1.315 |

**Table S1**. The selected important bond lengths (in Å) for TS1, TS1b and TS1c at different methods.

| Methods | N-O | N-H |
| --- | --- | --- |
| MP2/aTZ | 1.541 | 1.344 |
| MP2/maTZ | 1.543 | 1.353 |
| MP2/6-311++g(3df,3pd) | 1.538 | 1.337 |
| M06-2X/aTZ | 1.583 | 1.480 |
| M06-2X /maTZ | 1.581 | 1.475 |
| M06-2X /6-311++g(3df,3pd) | 1.578 | 1.468 |
| M06-HF/aTZ | 1.563 | 1.430 |
| M06-HF/maTZ | 1.567 | 1.441 |
| M06-HF /6-311++g(3df,3pd) | 1.562 | 1.429 |
| B3LYP/aTZ | 1.599 | 1.571 |
| B3LYP/maTZ | 1.603 | 1.578 |
| B3LYP/6-311++g(3df,3pd) | 1.596 | 1.566 |

**Table S2**. The selected important bond lengths (in Å) for TS4 at different methods.

| Species | Methods | | Frequencies |
| --- | --- | --- | --- |
| N_2_H_4_ | | MP2/aTZ | 427,830,1001,1130,1304,1339,1675,1687,3505,3509,3617,3621 |
|  | | MP2/maTZ | 427,835,1003,1134,1309,1337,1677,1692,3518,3522,3632,3635 |
|  | | MP2/6-311++g(3df,3pd) | 431,842,1002,1143,1305,1343,1674,1685,3528,3532,3636,3639 |
|  | | M06-2X/aTZ | 447,832,982,1142,1306,1336,1675,1688,3499,3503,3594,3599 |
|  | | M06-2X/maTZ | 453,831,978,1143,1306,1335,1674,1689,3501,3505,3603,3607 |
|  | | M06-2X/6-11++g(3df,3pd) | 449,840,989,1150,1308,1341,1676,1689,3507,3512,3602,3607 |
|  | | M06-HF/aTZ | 493,867,993,1162,1316,1346,1681,1692,3570,3574,3661,3666 |
|  | | M06-HF/maTZ | 488,865,987,1161,1316,1343,1685,1699,3572,3576,3666,3671 |
|  | | M06-HF/6-11++g(3df,3pd) | 473,889,1024,1163,1320,1361,1699,1711,3607,3615,3701,3709 |
|  | | B3LYP/aTZ | 440,796,972,1108,1295,1323,1672,1685,3455,3463,3557,3563 |
|  | | B3LYP/maTZ | 443,793,967,1107,1294,1320,1672,1685,3458,3466,3563,3569 |
|  | | B3LYP/6-311++g(3df,3pd) | 440,802,976,1113,1295,1324,1671,1683,3458,3467,3558,3564 |
| OH | | MP2/aTZ | 3795 |
|  | | MP2/maTZ | 3812 |
|  | | MP2/6-311++g(3df,3pd) | 3853 |
|  | | M06-2X/aTZ | 3745 |
|  | | M06-2X/maTZ | 3753 |
|  | | M06-2X/6-11++g(3df,3pd) | 3770 |
|  | | M06-HF/aTZ | 3746 |
|  | | M06-HF/maTZ | 3755 |
|  | | M06-HF/6-11++g(3df,3pd) | 3786 |
|  | | B3LYP/aTZ | 3699 |
|  | | B3LYP/maTZ | 3704 |
|  | | B3LYP/6-311++g(3df,3pd) | 3714 |
| N_2_H_3_ | | MP2/aTZ | 522,736,1147,1291,1494,1672,3501,3553,3706 |
|  | | MP2/maTZ | 528,734,1147,1293,14981670,3509,3563,3718 |
|  | | MP2/6-311++g(3df,3pd) | 509,736,1148,1304,1492,1672,3534,3572,3723 |
|  | | M06-2X/aTZ | 517,708,1145,1285,1487,1660,3444,3523,3671 |
|  | | M06-2X/maTZ | 515,709,1144,1286,1488,1659,3446,3524,3675 |
|  | | M06-2X/6-11++g(3df,3pd) | 530,706,1147,1292,1487,1660,3453,3528,3675 |
|  | | M06-HF/aTZ | 547,738,1142,1318,1506,1680,3467,3584,3715 |
|  | | M06-HF/maTZ | 534,736,1142,1320,1504,1676,3465,3592,3724 |
|  | | M06-HF/6-11++g(3df,3pd) | 511,736,1146,1312,1502,1681,3507,3630,3755 |
|  | | B3LYP/aTZ | 545,696,1137,1230,1480,1658,3418,3483,3627 |
|  | | B3LYP/maTZ | 549,698,1137,1229,1481,1657,3419,3483,3630 |
|  | | B3LYP/6-311++g(3df,3pd) | 547,696,1137,1236,1479,1656,3425,3484,3629 |
| H_2_O | | MP2/aTZ | 1628,3822,3947 |
|  | | MP2/maTZ | 1643,3845,3968 |
|  | | MP2/6-311++g(3df,3pd) | 1625,3870,3989 |
|  | | M06-2X/aTZ | 1616,3871,3975 |
|  | | M06-2X/maTZ | 1616,3879,3984 |
|  | | M06-2X/6-11++g(3df,3pd) | 1620,3889,3990 |
|  | | M06-HF/aTZ | 1593,3890,3977 |
|  | | M06-HF/maTZ | 1559,3897,3987 |
|  | | M06-HF/6-11++g(3df,3pd) | 1586,3931,4018 |
|  | | B3LYP/aTZ | 1627,3796,3899 |
|  | | B3LYP/maTZ | 1627,3802,3905 |
|  | | B3LYP/6-311++g(3df,3pd) | 1627,3813,3912 |
| NH_2_ | | MP2/aTZ | 1541,3445,3550 |
|  | | MP2/maTZ | 1547,3449,3556 |
|  | | MP2/6-311++g(3df,3pd) | 1533,3469,3570 |
|  | | M06-2X/aTZ | 1526,3402,3494 |
|  | | M06-2X/maTZ | 1525,3401,3496 |
|  | | M06-2X/6-11++g(3df,3pd) | 1527,3412,3501 |
|  | | M06-HF/aTZ | 1549,3417,3504 |
|  | | M06-HF/maTZ | 1546,3420,3501 |
|  | | M06-HF/6-11++g(3df,3pd) | 1565,3454,3528 |
|  | | B3LYP/aTZ | 1531,3353,3442 |
|  | | B3LYP/maTZ | 1528,3355,3448 |
|  | | B3LYP/6-311++g(3df,3pd) | 1529,3360,3448 |
| ^2^H_2_NOH | | MP2/aTZ | 414,935,1153,1334,1397,1665,3486,3583,3838 |
|  | | MP2/maTZ | 425,943,1155,1335,1404,1671,3498,3595,3859 |
|  | | MP2/6-311++g(3df,3pd) | 421,954,1155,1342,1396,1666,3510,3601,3885 |
|  | | M06-2X/aTZ | 440,1003,1156,1350,1411,1675,3467,3549,3878 |
|  | | M06-2X/maTZ | 448,1005,1154,1350,1411,1676,3468,3553,3887 |
|  | | M06-2X/6-11++g(3df,3pd) | 461,1012,1162,1357,1420,1678,3477,3556,3905 |
|  | | M06-HF/aTZ | 460,1035,1156,1369,1407,1710,3535,3609,3957 |
|  | | M06-HF/maTZ | 473,1038,1154,1370,1405,1701,3537,3613,3968 |
|  | | M06-HF/6-11++g(3df,3pd) | 539,1049,1173,1397,1418,1724,3585,3655,4026 |
|  | | B3LYP/aTZ | 417,923,1142,1326,1391,1664,3442,3520,3807 |
|  | | B3LYP/maTZ | 424,923,1139,1325,1391,1664,3444,3525,3813 |
|  | | B3LYP/6-311++g(3df,3pd) | 416,932,1143,1329,1392,1663,3446,3521,3828 |
| H_2_NNHOH | | MP2/aTZ | 306,446,495,825,887,1065,1200,1269,1401,1517,1690,3474,3535,3612,3818 |
|  | | MP2/maTZ | 305,446,497,833,892,1066,1203,1274,1405,1520,1690,3484,3547,3624,3838 |
|  | | MP2/6-311++g(3df,3pd) | 313,449,501,846,902,1074,1209,1274,1404,1522,1687,3496,3570,3630,3863 |
|  | | M06-2X/aTZ | 343,460,512,877,932,1063,1210,1288,1407,1529,1687,3460,3508,3582,3873 |
|  | | M06-2X/maTZ | 346,462,512,877,932,1062,1211,1289,1407,1529,1688,3463,3511,3590,3880 |
|  | | M06-2X/6-11++g(3df,3pd) | 357,464,516,885,945,1069,1215,1292,1410,1532,1688,3467,3518,3588,3898 |
|  | | M06-HF/aTZ | 354,440,523,908,977,1079,1233,1295,1407,1541,1701,3531,3554,3653,3937 |
|  | | M06-HF/maTZ | 371,449,523,905,978,1082,1231,1296,1407,1541,1699,3535,3559,3660,3951 |
|  | | M06-HF/6-11++g(3df,3pd) | 368,442,527,907,976,1088,1231,1298,1414,1536,1704,3565,3601,3690,3996 |
|  | | B3LYP/aTZ | 318,454,491,784,848,1042,1179,1261,1386,1509,1688,3431,3500,3562,3801 |
|  | | B3LYP/maTZ | 321,456,491,784,848,1040,1179,1260,1386,1509,1688,3433,3503,3566,3807 |
|  | | B3LYP/6-311++g(3df,3pd) | 320,456,493,796,855,1046,1184,1264,1388,1510,1685,3434,3508,3564,3815 |
| MCr1a | | MP2/aTZ | 72,165,231,400,631,771,866,1036,1132,1308,1349,1672,1690,3438,3503,3510,3612,3614 |
|  | | MP2/maTZ | 69,162,224,402,623,758,869,1040,1136,1310,1349,1674,1693,3470,3512,3522,3621,3628 |
|  | | MP2/6-311++g(3df,3pd) | 73,166,228,415,652,785,874,1035,1143,1310,1351,1672,1688,3486,3524,3533,3628,3632 |
|  | | M06-2X/aTZ | 100,205,228,378,610,764,869,1018,1140,1307,1348,1671,1689,3379,3493,3503,3593,3597 |
|  | | M06-2X/maTZ | 96,205,228,380,609,757,868,1017,1140,1306,1346,1671,1689,3394,3495,3506,3600,3602 |
|  | | M06-2X/6-11++g(3df,3pd) | 108,204,231,380,619,765,876,1022,1146,1308,1352,1671,1691,3411,3503,3507,3597,3605 |
|  | | M06-HF/aTZ | 112,232,256,468,736,836,892,1020,1155,1303,1345,1679,1691,3248,3544,3568,3633,3655 |
|  | | M06-HF/maTZ | 109,220,243,456,709,818,886,1024,1155,1308,1347,1679,1697,3277,3552,3576,3640,3666 |
|  | | M06-HF/6-11++g(3df,3pd) | 130,219,332,566,1029,1126,1187,1272,1441,1581,1640,1679,1787,2294,3393,3535,3604,3655 |
|  | | B3LYP/aTZ | 53,160,223,404,613,759,840,1019,1110,1298,1333,1670,1686,3295,3453,3473,3550,3564 |
|  | | B3LYP/maTZ | 54,161,222,403,610,753,837,1017,1110,1296,1332,1670,1687,3308,3455,3475,3555,3569 |
|  | | B3LYP/6-311++g(3df,3pd) | 57,162,223,408,620,766,845,1019,1115,1298,1334,1669,1685,3323,3457,3477,3553,3566 |
| MCr2 | | MP2/aTZ | 137,361,505,874,968,1005,1043,1145,1263,1351,1491,1495,1536,1641,3163,3359,3382,3476 |
|  | | MP2/maTZ | - |
|  | | MP2/6-311++g(3df,3pd) | 145,364,509,885,980,1015,1054,1152,1266,1358,1488,1497,1533,1639,3204,3388,3406,3492 |
|  | | M06-2X/aTZ | 161,303,514,821,1002,1039,1074,1181,1288,1352,1478,1498,1582,1641,3174,3331,3364,3433 |
|  | | M06-2X/maTZ | 185,315,502,747,936,1008,1068,1106,1262,1335,1371,1501,1521,1644,3143,3350,3376,3462 |
|  | | M06-2X/6-11++g(3df,3pd) | 134,370,501,697,962,1020,1074,1120,1262,1347,1385,1503,1528,1637,3158,3368,3385,3448 |
|  | | M06-HF/aTZ | 212,343,518,945,1034,1083,1185,1335,1366,1402,1504,1522,1663,2295,3327,3428,3468,3526 |
|  | | M06-HF/maTZ | 207,400,514,944,1034,1082,1174,1321,1358,1387,1518,1528,1664,2185,3331,3408,3433,3517 |
|  | | M06-HF/6-11++g(3df,3pd) | 192,383,515,945,1038,1091,1192,1336,1364,1388,1508,1521,1662,2227,3335,3445,3526,3539 |
|  | | B3LYP/aTZ | 162,289,516,746,812,984,1095,1137,1231,1347,1413,1478,1546,1611,3131,3228,3313,3505 |
|  | | B3LYP/maTZ | - |
|  | | B3LYP/6-311++g(3df,3pd) | 165,301,518,728,802,992,1092,1112,1215,1306,1375,1467,1512,1601,3158,3231,3323,3520 |
| MCp1 | | MP2/aTZ | 141,164,223,237,370,513,694,757,1158,1331,1491,1645,1667,3527,3537,3604,3686,3907 |
|  | | MP2/maTZ | 146,171,225,241,378,530,686,763,1160,1334,1494,1656,1673,3536,3546,3625,3697,3927 |
|  | | MP2/6-311++g(3df,3pd) | 144,165,221,242,374,510,692,760,1159,1342,1490,1643,1667,3557,3561,3648,3705,3949 |
|  | | M06-2X/aTZ | 167,184,227,254,366,535,660,742,1163,1315,1484,1631,1656,3503,3509,3642,3671,3947 |
|  | | M06-2X/maTZ | 166,188,229,249,377,539,645,741,1163,1316,1484,1629,1656,3507,3511,3646,3680,3959 |
|  | | M06-2X/6-11++g(3df,3pd) | 180,185,228,264,374,546,662,741,1164,1321,1484,1637,1653,3511,3514,3647,3702,3969 |
|  | | M06-HF/aTZ | 186,200,238,266,437,545,753,794,1169,1353,1500,1633,1685,3520,3537,3595,3678,3966 |
|  | | M06-HF/maTZ | 158,185,235,241,426,528,742,774,1157,1355,1490,1629,1672,3527,3546,3592,3691,3977 |
|  | | M06-HF/6-11++g(3df,3pd) | 170,197,240,264,443,548,755,798,1175,1346,1497,1620,1678,3560,3566,3659,3714,4016 |
|  | | B3LYP/aTZ | 110,170,214,230,379,549,687,721,1151,1260,1476,1642,1655,3453,3483,3562,3613,3868 |
|  | | B3LYP/maTZ | 114,173,216,232,379,553,681,724,1151,1260,1476,1642,1655,3455,3483,3571,3615,3876 |
|  | | B3LYP/6-311++g(3df,3pd) | 111,169,214,236,377,549,696,722,1150,1267,1474,1641,1654,3463,3485,3581,3615,3883 |
| MCp2 | | MP2/aTZ | 144,165,216,225,386,473,678,755,1153,1332,1490,1646,1671,3527,3541,3601,3692,3907 |
|  | | MP2/maTZ | 144,177,213,226,389,476,671,759,1154,1336,1493,1655,1679,3536,3552,3626,3704,3927 |
|  | | MP2/6-311++g(3df,3pd) | 143,164,218,222,383,447,674,756,1153,1345,1489,1644,1671,3561,3565,3652,3713,3949 |
|  | | M06-2X/aTZ | 169,187,221,227,377,473,625,727,1156,1317,1480,1631,1660,3472,3510,3645,3662,3952 |
|  | | M06-2X/maTZ | 178,201,219,229,382,477,616,727,1156,1317,1480,1630,1661,3475,3511,3648,3669,3962 |
|  | | M06-2X/6-11++g(3df,3pd) | 174,219,227,258,365,481,651,731,1156,1322,1480,1634,1657,3486,3515,3652,3690,3971 |
|  | | M06-HF/aTZ | 179,239,275,301,401,510,735,758,1164,1356,1502,1626,1685,3492,3533,3578,3686,3969 |
|  | | M06-HF/maTZ | 159,234,242,269,426,500,694,743,1154,1357,1491,1628,1674,3504,3535,3575,3693,3976 |
|  | | M06-HF/6-11++g(3df,3pd) | 178,241,293,361,400,514,748,769,1171,1349,1499,1613,1681,3550,3571,3638,3733,4027 |
|  | | B3LYP/aTZ | 107,170,207,214,382,507,673,716,1146,1262,1474,1640,1661,3453,3488,3565,3619,3869 |
|  | | B3LYP/maTZ | 110,156,201,216,385,510,668,718,1146,1263,1474,1640,1662,3456,3488,3572,3622,3876 |
|  | | B3LYP/6-311++g(3df,3pd) | 108,177,213,217,381,508,681,719,1146,1269,1473,1639,1660,3463,3489,3583,3622,3883 |
| MCp3a | | MP2/aTZ | 45,56,124,124,220,242,418,943,1172,1326,1399,1535,1656,3435,3486,3545,3577,3841 |
|  | | MP2/maTZ | 39,51,118,124,215,232,427,953,1176,1328,1407,1543,1665,3445,3497,3556,3590,3861 |
|  | | MP2/6-311++g(3df,3pd) | 50,53,124,140,217,245,424,965,1174,1336,1400,1528,1657,3463,3511,3569,3596,3887 |
|  | | M06-2X/aTZ | 48,89,146,165,251,299,405,1016,1175,1340,1409,1524,1665,3406,3482,3499,3557,3889 |
|  | | M06-2X/maTZ | 52,93,149,168,259,293,416,1018,1175,1342,1410,1524,1667,3408,3485,3505,3565,3895 |
|  | | M06-2X/6-11++g(3df,3pd) | 32i,77,142,144,236,342,409,1024,1176,1340,1413,1520,1664,3415,3486,3511,3560,3913 |
|  | | M06-HF/aTZ | 308i,86,105,129,248,298,385,1048,1174,1354,1400,1561,1693,3429,3517,3541,3609,3938 |
|  | | M06-HF/maTZ | 307i,76,82,115,271,307,408,1048,1167,1347,1399,1566,1688,3429,3515,3549,3621,3939 |
|  | | M06-HF/6-11++g(3df,3pd) | 302i,33,54,135,237,383,460,1040,1154,1366,1396,1573,1704,3457,3528,3564,3631,3997 |
|  | | B3LYP/aTZ | 35,42,101,106,280,311,434,936,1154,1326,1397,1546,1664,3314,3423,3443,3518,3807 |
|  | | B3LYP/maTZ | 39i,39,95,106,271,304,437,936,1153,1325,1397,1543,1664,3318,3429,3445,3523,3813 |
|  | | B3LYP/6-311++g(3df,3pd) | 31,40,99,108,283,320,439,945,1154,1329,1399,1545,1664,3321,3428,3447,3521,3823 |
| MCp3b | | MP2/aTZ | 134,141,152,195,255,280,455,928,1174,1357,1390,1539,1677,3432,3472,3547,3571,3835 |
|  | | MP2/maTZ | 127,135,147,188,255,274,461,938,1179,1359,1399,1545,1684,3443,3483,3558,3581,3858 |
|  | | MP2/6-311++g(3df,3pd) | 135,145,150,198,261,283,460,949,1176,1365,1390,1532,1678,3459,3498,3570,3590,3884 |
|  | | M06-2X/aTZ | 162,173,201,247,294,318,482,998,1176,1379,1397,1530,1690,3395,3460,3498,3551,3885 |
|  | | M06-2X/maTZ | 161,173,206,250,286,320,489,999,1174,1378,1398,1526,1690,3397,3460,3500,3556,3892 |
|  | | M06-2X/6-11++g(3df,3pd) | 166,178,200,248,300,321,484,1008,1182,1384,1403,1529,1691,3407,3474,3514,3562,3909 |
|  | | M06-HF/aTZ | 65,147,154,230,276,310,531,1030,1176,1383,1398,1546,1705,3372,3463,3506,3602,3952 |
|  | | M06-HF/maTZ | 160i,143,165,235,301,318,522,1030,1171,1384,1395,1560,1709,3392,3479,3512,3606,3955 |
|  | | M06-HF/6-11++g(3df,3pd) | 569i,152,158,190,295,412,541,1041,1178,1388,1401,1558,1723,3393,3502,3543,3634,4002 |
|  | | B3LYP/aTZ | 106,111,132,180,239,269,447,916,1163,1348,1384,1526,1674,3355,3431,3452,3511,3811 |
|  | | B3LYP/maTZ | 107,113,135,180,241,269,453,916,1162,1348,1384,1524,1675,3357,3431,3456,3515,3818 |
|  | | B3LYP/6-311++g(3df,3pd) | 110,120,135,187,248,278,455,925,1164,1351,1386,1524,1674,3360,3437,3456,3516,3828 |
| MCP4 | | MP2/aTZ | 30,60,119,307,445,495,825,887,1064,1200,1269,1401,1516,1690,3474,3535,3610,3814 |
|  | | MP2/maTZ | 17,40,81,305,446,497,832,892,1065,1203,1274,1405,1520,1690,3484,3547,3623,3836 |
|  | | MP2/6-311++g(3df,3pd) | 34,54,115,313,449,501,846,903,1072,1209,1274,1404,1522,1687,3495,3570,3628,3860 |
|  | | M06-2X/aTZ | 164,188,311,356,456,513,873,933,1061,1208,1290,1408,1523,1686,3462,3510,3573,3855 |
|  | | M06-2X/maTZ | 143,179,255,333,460,513,874,934,1056,1209,1289,1405,1525,1686,3464,3514,3583,3863 |
|  | | M06-2X/6-11++g(3df,3pd) | 13,26,67,353,460,516,886,947,1075,1214,1300,1415,1536,1690,3484,3551,3603,3904 |
|  | | M06-HF/aTZ | - |
|  | | M06-HF/maTZ | - |
|  | | M06-HF/6-11++g(3df,3pd) | - |
|  | | B3LYP/aTZ | 148,176,228,346,449,494,804,854,1023,1179,1274,1391,1503,1683,3437,3505,3558,3766 |
|  | | B3LYP/maTZ | 122,146,195,340,451,494,799,851,1026,1179,1271,1390,1504,1684,3437,3507,3564,3779 |
|  | | B3LYP/6-311++g(3df,3pd) | 150,181,232,346,451,496,814,862,1027,1184,1275,1393,1504,1681,3439,3513,3560,3782 |
| TS2 | | MP2/aTZ | 1236i,115,146,250,513,740,782,915,1118,1253,1381,1573,1681,2015,3490,3567,3616,3797 |
|  | | MP2/maTZ | 1239i,115,142,246,516,750,780,915,1124,1258,1385,1578,1684,2002,3500,3580,3629,3814 |
|  | | MP2/6-311++g(3df,3pd) | 1173i,106,140,248,525,745,777,923,1131,1256,1383,1578,1680,2041,3513,3590,3635,3844 |
|  | | M06-2X/aTZ | 372i,141,178,217,515,571,761,926,1131,1254,1367,1639,1678,3111,3460,3581,3598,3788 |
|  | | M06-2X/maTZ | 355i,153,200,229,517,556,759,925,1134,1256,1367,1639,1678,3134,3462,3587,3604,3796 |
|  | | M06-2X/6-11++g(3df,3pd) | 401i,130,170,223,515,575,762,935,1139,1256,1372,1638,1679,3090,3464,3588,3605,3812 |
|  | | M06-HF/aTZ | 1186i,139,263,293,517,672,727,942,1146,1260,1361,1622,1698,2433,3551,3626,3649,3797 |
|  | | M06-HF/maTZ | 1213i,141,264,294,524,671,723,935,1146,1263,1365,1623,1695,2412,3551,3633,3656,3799 |
|  | | M06-HF/6-11++g(3df,3pd) | - |
|  | | B3LYP/aTZ | -545i,102,181,194,222,464,718,957,1152,1269,1306,1623,1685,3067,3476,3524,3574,3738 |
|  | | B3LYP/maTZ | 555i,105,187,197,225,468,717,954,1154,1267,1305,1623,1685,3058,3478,3528,3579,3744 |
|  | | B3LYP/6-311++g(3df,3pd) | 541i,102,181,195,218,464,717,952,1159,1268,1306,1621,1683,3074,3480,3528,3576,3758 |
| TS3 | | MP2/aTZ | 1889i,165,198,366,453,734,847,897,1121,1237,1401,1610,1668,1792,3522,3546,3625,3750 |
|  | | MP2/maTZ | 1874i,164,200,366,451,735,853,901,1127,1241,1406,1620,1673,1788,3533,3560,3638,3774 |
|  | | MP2/6-311++g(3df,3pd) | 1814i,164,203,361,452,735,846,897,1132,1244,1404,1614,1668,1801,3543,3571,3642,3802 |
|  | | M06-2X/aTZ | 696i,156,188,311,414,698,822,888,1133,1256,1403,1633,1680,2651,3525,3563,3614,3755 |
|  | | M06-2X/maTZ | 530i,103,149,225,482,604,773,796,1141,1279,1347,1629,1669,2822,3521,3548,3622,3774 |
|  | | M06-2X/6-11++g(3df,3pd) | 693i,159,187,316,413,703,828,895,1140,1261,1405,1634,1680,2642,3531,3569,3618,3778 |
|  | | M06-HF/aTZ | 1825i,149,191,384,521,713,807,876,1144,1247,1384,1638,1685,2018,3575,3587,3664,3753 |
|  | | M06-HF/maTZ | 1829i,181,261,385,539,715,823,906,1147,1239,1388,1631,1685,2018,3581,3597,3667,3763 |
|  | | M06-HF/6-11++g(3df,3pd) | 1858i,182,242,382,506,752,828,892,1154,1266,1390,1639,1694,2020,3612,3640,3693,3801 |
|  | | B3LYP/aTZ | 217i,145,175,271,397,556,721,867,1109,1287,1303,1651,1663,3312,3471,3513,3561,3728 |
|  | | B3LYP/maTZ | 220i,146,175,270,403,551,721,860,1110,1286,1303,1650,1663,3310,3474,3515,3568,3733 |
|  | | B3LYP/6-311++g(3df,3pd) | 226i,146,176,273,394,558,724,865,1116,1288,1303,1648,1661,3302,3475,3516,3565,3747 |
| TS1 | | MP2/aTZ | 1323i,126,302,333,514,682,945,987,1125,1366,1392,1521,1591,3472,3536,3578,3642,3851 |
|  | | MP2/maTZ | 1328i,156,304,334,517,680,944,987,1134,1364,1396,1524,1593,3482,3547,3590,3655,3872 |
|  | | MP2/6-311++g(3df,3pd) | 1335i,138,303,335,517,682,949,989,1130,1370,1396,1515,1587,3496,3556,3595,3657,3898 |
|  | | M06-2X/aTZ | 928i,225,269,289,459,522,910,925,1081,1271,1324,1529,1573,3431,3523,3531,3624,3885 |
|  | | M06-2X/maTZ | 930i,233,271,289,462,522,910,923,1082,1270,1324,1530,1572,3432,3528,3532,3628,3891 |
|  | | M06-2X/6-11++g(3df,3pd) | 942i,235,269,291,457,522,913,932,1091,1279,1331,1529,1576,3442,3530,3543,3632,3912 |
|  | | M06-HF/aTZ | 950i,294,301,347,484,562,944,953,1073,1305,1353,1550,1586,3471,3555,3581,3673,3927 |
|  | | M06-HF/maTZ | 966i,301,302,365,496,560,939,942,1078,1303,1351,1549,1591,3472,3556,3584,3672,3934 |
|  | | M06-HF/6-11++g(3df,3pd) | 978i,305,327,383,492,563,938,976,1097,1301,1353,1566,1608,3505,3585,3616,3701,3991 |
|  | | B3LYP/aTZ | 758i,149,255,266,433,478,857,890,1055,1207,1273,1526,1567,3415,3504,3507,3601,3811 |
|  | | B3LYP/maTZ | 759i,156,257,266,435,477,856,887,1055,1206,1272,1526,1566,3415,3508,3509,3604,3817 |
|  | | B3LYP/6-311++g(3df,3pd) | 768i,156,257,268,435,477,859,893,1058,1211,1276,1524,1566,3421,3508,3514,3606,3830 |
| TS1b | | MP2/aTZ | 6845i,226,280,465,589,804,876,1008,1145,1269,1418,1474,1535,1594,3322,3416,3434,3554 |
|  | | MP2/maTZ | - |
|  | | MP2/6-311++g(3df,3pd) | 5016i,194,285,526,626,789,836,993,1164,1303,1450,1516,1531,1615,3364,3447,3460,3566 |
|  | | M06-2X/aTZ | 2023i,219,285,473,664,890,923,1102,1161,1286,1359,1517,1540,1691,3312,3371,3407,3487 |
|  | | M06-2X/maTZ | 2082i,222,291,502,645,892,927,1093,1132,1283,1296,1462,1541,1557,3320,3359,3411,3518 |
|  | | M06-2X/6-11++g(3df,3pd) | 2094i,232,304,528,664,901,938,1094,1128,1268,1291,1482,1541,1572,3324,3383,3419,3514 |
|  | | M06-HF/aTZ | 1792i,210,342,399,732,954,1004,1152,1198,1295,1406,1511,1547,2095,3358,3440,3514,3553 |
|  | | M06-HF/maTZ | 1860i,94i,265,569,649,690,708,1072,1239,1365,1512,1550,1635,1952,3391,3450,3552,3587 |
|  | | M06-HF/6-11++g(3df,3pd) | 1533i,72i,258,479,619,712,726,1075,1239,1355,1515,1543,1651,2119,3390,3480,3567,3656 |
|  | | B3LYP/aTZ | 1846i,228,331,425,674,707,908,949,1128,1232,1264,1477,1500,1715,3174,3274,3362,3561 |
|  | | B3LYP/maTZ | - |
|  | | B3LYP/6-311++g(3df,3pd) | 1907i,222,320,439,663,677,899,943,1113,1232,1264,1473,1497,1630,3206,3292,3368,3572 |
| TS1c | | MP2/aTZ | 1439i,195,329,436,764,948,1094,1136,1169,1308,1382,1430,1561,1949,3387,3431,3501,3546 |
|  | | MP2/maTZ | 1373i,202,346,442,713,957,1086,1134,1161,1309,1382,1428,1561,1675,3398,3452,3518,3567 |
|  | | MP2/6-311++g(3df,3pd) | 2368i,214,347,413,681,964,993,1089,1189,1275,1403,1481,1543,2487,3400,3504,3610,3718 |
|  | | M06-2X/aTZ | 1358i,214,297,412,465,947,1023,1059,1112,1198,1350,1377,1439,1569,3430,3474,3571,3676 |
|  | | M06-2X/maTZ | 1412i,216,294,430,522,937,1018,1034,1092,1187,1347,1380,1435,1569,3438,3477,3578,3716 |
|  | | M06-2X/6-11++g(3df,3pd) | 1380i,209,295,408,466,952,1030,1064,1115,1201,1355,1381,1446,1570,3437,3482,3576,3702 |
|  | | M06-HF/aTZ | 2064i,209,361,426,593,1021,1028,1112,1187,1301,1428,1477,1550,2208,3389,3484,3589,3725 |
|  | | M06-HF/maTZ | 1693i,252,367,447,588,1020,1040,1102,1184,1307,1430,1462,1562,2107,3389,3496,3598,3705 |
|  | | M06-HF/6-11++g(3df,3pd) | 2420i,42,295,371,589,992,1044,1120,1193,1288,1399,1507,1568,2356,3385,3534,3622,3758 |
|  | | B3LYP/aTZ | 1028i,129,222,298,446,952,1014,1087,1176,1297,1342,1442,1459,1563,3300,3358,3419,3523 |
|  | | B3LYP/maTZ | 1222i,161,280,422,506,871,919,949,1063,1167,1294,1361,1432,1569,3424,3434,3528,3638 |
|  | | B3LYP/6-311++g(3df,3pd) | 1205i,157,295,383,436,887,961,967,1080,1178,1300,1360,1438,1567,3421,3434,3527,3600 |
| TS4 | | MP2/aTZ | 2164i,172,296,424,550,823,901,1157,1173,1178,1239,1407,1449,1668,3492,3518,3601,3815 |
|  | | MP2/maTZ | 2189i,200,301,451,615,823,907,1157,1171,1186,1243,1414,1451,1671,3507,3532,3617,3840 |
|  | | MP2/6-311++g(3df,3pd) | 2169i,168,299,425,551,836,915,1164,1175,1179,1244,1409,1451,1665,3515,3549,3618,3860 |
|  | | M06-2X/aTZ | 1487i,192,311,434,535,713,861,911,1132,1168,1209,1355,1456,1676,3490,3500,3586,3882 |
|  | | M06-2X/maTZ | 1500i,204,317,437,549,709,857,911,1133,1166,1210,1356,1455,1676,3493,3502,3593,3890 |
|  | | M06-2X/6-11++g(3df,3pd) | 1508i,189,312,437,541,720,867,922,1142,1176,1213,1359,1457,1675,3497,3510,3589,3908 |
|  | | M06-HF/aTZ | 1622i,217,317,451,540,770,949,961,1146,1187,1247,1387,1471,1695,3533,3538,3630,3932 |
|  | | M06-HF/maTZ | 1653i,256,327,457,565,765,948,957,1138,1189,1241,1383,1469,1691,3536,3543,3636,3941 |
|  | | M06-HF/6-11++g(3df,3pd) | 1666i,254,320,453,541,776,956,965,1134,1196,1265,1376,1483,1703,3578,3589,3666,3992 |
|  | | B3LYP/aTZ | 1140i,200,326,409,480,610,735,847,1101,1148,1184,1321,1458,1680,3476,3501,3572,3801 |
|  | | B3LYP/maTZ | 1146i,206,325,414,492,607,731,845,1099,1145,1184,1319,1457,1680,3479,3505,3577,3808 |
|  | | B3LYP/6-311++g(3df,3pd) | 1150i,200,327,411,483,616,740,854,1105,1153,1187,1324,1457,1677,3479,3509,3573,3818 |

**Table S3**. Unscaled vibrational frequencies (in cm^-1^) of stationary points of reliable paths calculated at at different levels.

| Species | Method | $E_{elec}$ | ZPE |
| --- | --- | --- | --- |
| N_2_H_4_ | MP2/aTZ | -111.67679456 | 0.053865 |
|  | MP2/maTZ | -111.66874082 | 0.054045 |
|  | MP2/6-311++g(3df,3pd) | -111.66973126 | 0.054129 |
|  | M06-2X/aTZ | -111.86322143 | 0.053771 |
|  | M06-2X/maTZ | -111.86226847 | 0.053823 |
|  | M06-2X/6-11++g(3df,3pd) | -111.85999447 | 0.053926 |
|  | M06-HF/aTZ | -111.88590731 | 0.054723 |
|  | M06-HF/maTZ | -111.88295198 | 0.054743 |
|  | M06-HF/6-11++g(3df,3pd) | -111.87715684 | 0.055297 |
|  | B3LYP/aTZ | -111.92148963 | 0.053146 |
|  | B3LYP/maTZ | -111.92057907 | 0.053168 |
|  | B3LYP/6-311++g(3df,3pd) | -111.91911748 | 0.053193 |
|  | CASSCF(6,6) – MP2/aQZ//MP2/aTZ | -111.7510115 | - |
|  | CCSD(T)/CBS//MP2/aTZ | -111.762193 | - |
|  | CCSD(T)/aQZ//MP2/aTZ | -111.7407136 | - |
|  | CCSD(T)/TZ//MP2/aTZ | -111.6985939 | - |
|  | CCSD(T)/aTZ//MP2/aTZ | -111.7112789 | - |
|  | CCSD(T)/maTZ//MP2/aTZ | -111.7029887 | - |
|  | CCSD(T)-full/aTZ//MP2/aTZ | -111.7457393 | - |
|  | CCSD(T)-full/maTZ//MP2/aTZ | -111.7323895 | - |
|  | CCSD(T)/DZ//MP2/aTZ | -111.5699538 | - |
|  | CCSD(T)/aDZ//MP2/aTZ | -111.6081187 | - |
|  | CCSD(T)/maDZ//MP2/aTZ | -111.5855419 | - |
|  | CCSD(T)-full/aDZ//MP2/aTZ | -111.6131857 | - |
|  | CCSD(T)-full/maDZ//MP2/aTZ | -111.5901723 | - |
|  | CCSD(T)/6-31++g(2df,pd) //MP2/aTZ | -111.6565439 | - |
|  | CCSDT-full/6-31++g(2df,2p) //MP2/aTZ | -111.6697695 | - |
|  | CCSD(T)-full-6-31++g(2df,3p) //MP2/aTZ | -111.6722576 | - |
|  | CCSD(T)-full-6-31++g(2df,pd) //MP2/aTZ | -111.6780422 | - |
|  | CCSD(T)-full-6-31++g(3df,2p) //MP2/aTZ | -111.6769323 | - |
|  | CCSD(T)-full-6-31++g(3df,3pd) //MP2/aTZ | -111.6945598 | - |
|  | CCSD(T)-full-6-31++g(3df,pd) //MP2/aTZ | -111.6882299 | - |
|  | CCSD(T)-full-6-31++g(df,2p) //MP2/aTZ | -111.6366439 | - |
|  | CCSD(T)-full-6-31++g(df,pd) //MP2/aTZ | -111.6424232 | - |
|  | CCSD(T)-full-6-311++g(2df,2p) //MP2/aTZ | -111.7342703 | - |
|  | CCSD(T)-full-6-311++g(3d2f,3pd) //MP2/aTZ | -111.7516088 | - |
|  | CCSD(T)-full-6-311++g(3df,2p) //MP2/aTZ | -111.7424687 | - |
|  | CCSD(T)-full-6-311++g(3df,3pd) //MP2/aTZ | -111.7565234 | - |
|  | CCSD(T)-full-6-311++g(df,2p) //MP2/aTZ | -111.7155198 | - |
|  | CCSD(T)/aTZ//M06-2X/maTZ | -111.7110586 | - |
|  | CCSD(T)-full/aTZ//M06-2X/maTZ | -111.7456146 | - |
|  | CCSD(T)-full-6-31++g(3df,3pd)//M06-2X/maTZ | -111.6942843 | - |
|  | CCSD(T)-full-6-311++g(3df,3pd)//M06-2X/maTZ | -111.7514906 | - |
|  | CCSD(T)-full-6-311++g(3d2f,3pd)//M06-2X/maTZ | -111.7564053 | - |
| OH | MP2/aTZ | -75.62633729 | 0.008645 |
|  | MP2/maTZ | -75.62208295 | 0.008685 |
|  | MP2/6-311++g(3df,3pd) | -75.62080774 | 0.008777 |
|  | M06-2X/aTZ | -75.73378897 | 0.008532 |
|  | M06-2X/maTZ | -75.73336497 | 0.008549 |
|  | M06-2X/6-11++g(3df,3pd) | -75.73033617 | 0.008589 |
|  | M06-HF/aTZ | -75.74217647 | 0.008534 |
|  | M06-HF/maTZ | -75.74128171 | 0.008554 |
|  | M06-HF/6-11++g(3df,3pd) | -75.73309285 | 0.008625 |
|  | B3LYP/aTZ | -75.76859918 | 0.008427 |
|  | B3LYP/maTZ | -75.76815499 | 0.008439 |
|  | B3LYP/6-311++g(3df,3pd) | -75.76624485 | 0.008461 |
|  | CASSCF(3,3) – MP2/aQZ//MP2/aTZ | -75.6701273 | - |
|  | CCSD(T)/CBS//MP2/aTZ | -75.67830055 | - |
|  | CCSD(T)/aQZ//MP2/aTZ | -75.6644937 | - |
|  | CCSD(T)/TZ//MP2/aTZ | -75.6377212 | - |
|  | CCSD(T)/aTZ//MP2/aTZ | -75.6455732 | - |
|  | CCSD(T)/maTZ//MP2/aTZ | -75.6408966 | - |
|  | CCSD(T)-full/aTZ//MP2/aTZ | -75.6590872 | - |
|  | CCSD(T)-full/maTZ//MP2/aTZ | -75.6534076 | - |
|  | CCSD(T)/DZ//MP2/aTZ | -75.5592629 | - |
|  | CCSD(T)/aDZ//MP2/aTZ | -75.5839882 | - |
|  | CCSD(T)/maDZ//MP2/aTZ | -75.5713562 | - |
|  | CCSD(T)-full/aDZ//MP2/aTZ | -75.5859632 | - |
|  | CCSD(T)-full/maDZ//MP2/aTZ | -75.5732638 | - |
|  | CCSD(T)/6-31++g(2df,pd) //MP2/aTZ | -75.6060746 | - |
|  | CCSDT-full/6-31++g(2df,2p) //MP2/aTZ | -75.610272 | - |
|  | CCSD(T)-full-6-31++g(2df,3p) //MP2/aTZ | -75.6106968 | - |
|  | CCSD(T)-full-6-31++g(2df,pd) //MP2/aTZ | -75 .6131826 | - |
|  | CCSD(T)-full-6-31++g(3df,2p) //MP2/aTZ | -75.6149538 | - |
|  | CCSD(T)-full-6-31++g(3df,3pd) //MP2/aTZ | -75.6202986 | - |
|  | CCSD(T)-full-6-31++g(3df,pd) //MP2/aTZ | -75.6187814 | - |
|  | CCSD(T)-full-6-31++g(df,2p) //MP2/aTZ | -75.5806627 | - |
|  | CCSD(T)-full-6-31++g(df,pd) //MP2/aTZ | -75.5824843 | - |
|  | CCSD(T)-full-6-311++g(2df,2p) //MP2/aTZ | -75.6545717 | - |
|  | CCSD(T)-full-6-311++g(3d2f,3pd) //MP2/aTZ | -75.6664161 | - |
|  | CCSD(T)-full-6-311++g(3df,2p) //MP2/aTZ | -75.6596305 | - |
|  | CCSD(T)-full-6-311++g(3df,3pd) //MP2/aTZ | -75.662965 | - |
|  | CCSD(T)-full-6-311++g(df,2p) //MP2/aTZ | -75.63836 | - |
|  | CCSD(T)/aTZ//M06-2X/maTZ | -75.6455845 | - |
|  | CCSD(T)-full/aTZ//M06-2X/maTZ | -75.659084 | - |
|  | CCSD(T)-full-6-31++g(3df,3pd)//M06-2X/maTZ | -75.6202784 | - |
|  | CCSD(T)-full-6-311++g(3df,3pd)//M06-2X/maTZ | -75.6629584 | - |
|  | CCSD(T)-full-6-311++g(3d2f,3pd)//M06-2X/maTZ | -75.6664082 | - |
| N_2_H_3_ | MP2/aTZ | -111.03284355 | 0.040145 |
|  | MP2/maTZ | -111.02550111 | 0.040245 |
|  | MP2/6-311++g(3df,3pd) | -111.02577158 | 0.040302 |
|  | M06-2X/aTZ | -111.22392029 | 0.039731 |
|  | M06-2X/maTZ | -111.22301415 | 0.039747 |
|  | M06-2X/6-11++g(3df,3pd) | -111.22014976 | 0.039815 |
|  | M06-HF/aTZ | -111.24317373 | 0.040322 |
|  | M06-HF/maTZ | -111.24055738 | 0.040303 |
|  | M06-HF/6-11++g(3df,3pd) | -111.23255454 | 0.040506 |
|  | B3LYP/aTZ | -111.28293246 | 0.039352 |
|  | B3LYP/maTZ | -111.28210878 | 0.039371 |
|  | B3LYP/6-311++g(3df,3pd) | -111.28028758 | 0.039389 |
|  | CASSCF(5,5) – MP2/aQZ//MP2/aTZ | -111.1247587 | - |
|  | CCSD(T)/CBS//MP2/aTZ | -111.119013 | - |
|  | CCSD(T)/aQZ//MP2/aTZ | -111.0982728 | - |
|  | CCSD(T)/TZ//MP2/aTZ | -111.0576218 | - |
|  | CCSD(T)/aTZ//MP2/aTZ | -111.0698511 | - |
|  | CCSD(T)/maTZ//MP2/aTZ | -111.0620602 | - |
|  | CCSD(T)-full/aTZ//MP2/aTZ | -111.1032736 | - |
|  | CCSD(T)-full/maTZ//MP2/aTZ | -111.090805 | - |
|  | CCSD(T)/DZ//MP2/aTZ | -110.9367656 | - |
|  | CCSD(T)/aDZ//MP2/aTZ | -110.9722379 | - |
|  | CCSD(T)/maDZ//MP2/aTZ | -110.9518838 | - |
|  | CCSD(T)-full/aDZ//MP2/aTZ | -110.9770455 | - |
|  | CCSD(T)-full/maDZ//MP2/aTZ | -110.9563253 | - |
|  | CCSD(T)/6-31++g(2df,pd) //MP2/aTZ | -111.002608 | - |
|  | CCSDT-full/6-31++g(2df,2p) //MP2/aTZ | -111.0312289 | - |
|  | CCSD(T)-full-6-31++g(2df,3p) //MP2/aTZ | -111.0327762 | - |
|  | CCSD(T)-full-6-31++g(2df,pd) //MP2/aTZ | -111.0373037 | - |
|  | CCSD(T)-full-6-31++g(3df,2p) //MP2/aTZ | -111.0379931 | - |
|  | CCSD(T)-full-6-31++g(3df,3pd) //MP2/aTZ | -111.0507332 | - |
|  | CCSD(T)-full-6-31++g(3df,pd) //MP2/aTZ | -111.0460751 | - |
|  | CCSD(T)-full-6-31++g(df,2p) //MP2/aTZ | -110.9975113 | - |
|  | CCSD(T)-full-6-31++g(df,pd) //MP2/aTZ | -111.0016462 | - |
|  | CCSD(T)-full-6-311++g(2df,2p) //MP2/aTZ | -111.0944654 | - |
|  | CCSD(T)-full-6-311++g(3d2f,3pd) //MP2/aTZ | -111.1144743 | - |
|  | CCSD(T)-full-6-311++g(3df,2p) //MP2/aTZ | -111.1030133 | - |
|  | CCSD(T)-full-6-311++g(3df,3pd) //MP2/aTZ | -111.1097409 | - |
|  | CCSD(T)-full-6-311++g(df,2p) //MP2/aTZ | -111.074431 | - |
|  | CCSD(T)/aTZ//M06-2X/maTZ | -111.0698765 | - |
|  | CCSD(T)-full/aTZ//M06-2X/maTZ | -111.1032631 | - |
|  | CCSD(T)-full-6-31++g(3df,3pd)//M06-2X/maTZ | -111.0507142 | - |
|  | CCSD(T)-full-6-311++g(3df,3pd)//M06-2X/maTZ | -111.1097518 | - |
|  | CCSD(T)-full-6-311++g(3d2f,3pd)//M06-2X/maTZ | -111.1144787 | - |
| H_2_O | MP2/aTZ | -76.32899232 | 0.021409 |
|  | MP2/maTZ | -76.32387205 | 0.021541 |
|  | MP2/6-311++g(3df,3pd) | -76.32428624 | 0.021606 |
|  | M06-2X/aTZ | -76.43009225 | 0.021556 |
|  | M06-2X/maTZ | -76.42947713 | 0.021593 |
|  | M06-2X/6-11++g(3df,3pd) | -76.42701160 | 0.021638 |
|  | M06-HF/aTZ | -76.44035772 | 0.021553 |
|  | M06-HF/maTZ | -76.43888141 | 0.021597 |
|  | M06-HF/6-11++g(3df,3pd) | -76.43222726 | 0.021726 |
|  | B3LYP/aTZ | -76.46619656 | 0.021237 |
|  | B3LYP/maTZ | -76.46554007 | 0.021262 |
|  | B3LYP/6-311++g(3df,3pd) | -76.46451155 | 0.021305 |
|  | CASSCF(4,4) – MP2/aQZ//MP2/aTZ | -76.38000027 |  |
|  | CCSD(T)/CBS//MP2/aTZ | -76.37908226 | - |
|  | CCSD(T)/aQZ//MP2/aTZ | -76.3635755 | - |
|  | CCSD(T)/TZ//MP2/aTZ | -76.3321996 | - |
|  | CCSD(T)/aTZ//MP2/aTZ | -76.3423255 | - |
|  | CCSD(T)/maTZ//MP2/aTZ | -76.3370064 | - |
|  | CCSD(T)-full/aTZ//MP2/aTZ | -76.3575186 | - |
|  | CCSD(T)-full/maTZ//MP2/aTZ | -76.3506781 | - |
|  | CCSD(T)/DZ//MP2/aTZ | -76.2411565 | - |
|  | CCSD(T)/aDZ//MP2/aTZ | -76.273855 | - |
|  | CCSD(T)/maDZ//MP2/aTZ | -76.2574664 | - |
|  | CCSD(T)-full/aDZ//MP2/aTZ | -76.2761123 | - |
|  | CCSD(T)-full/maDZ//MP2/aTZ | -76.2596156 | - |
|  | CCSD(T)/6-31++g(2df,pd) //MP2/aTZ | -76.3015924 | - |
|  | CCSDT-full/6-31++g(2df,2p) //MP2/aTZ | -76.3036546 | - |
|  | CCSD(T)-full-6-31++g(2df,3p) //MP2/aTZ | -76.3047018 | - |
|  | CCSD(T)-full-6-31++g(2df,pd) //MP2/aTZ | -76.309653 | - |
|  | CCSD(T)-full-6-31++g(3df,2p) //MP2/aTZ | -76.3092677 | - |
|  | CCSD(T)-full-6-31++g(3df,3pd) //MP2/aTZ | -76.3198894 | - |
|  | CCSD(T)-full-6-31++g(3df,pd) //MP2/aTZ | -76.3167586 | - |
|  | CCSD(T)-full-6-31++g(df,2p) //MP2/aTZ | -76.2724739 | - |
|  | CCSD(T)-full-6-31++g(df,pd) //MP2/aTZ | -76.2758581 | - |
|  | CCSD(T)-full-6-311++g(2df,2p) //MP2/aTZ | -76.3498609 | - |
|  | CCSD(T)-full-6-311++g(3d2f,3pd) //MP2/aTZ | -76.3646002 | - |
|  | CCSD(T)-full-6-311++g(3df,2p) //MP2/aTZ | -76.355133 | - |
|  | CCSD(T)-full-6-311++g(3df,3pd) //MP2/aTZ | -76.3613843 | - |
|  | CCSD(T)-full-6-311++g(df,2p) //MP2/aTZ | -76.3357875 | - |
|  | CCSD(T)/aTZ//M06-2X/maTZ | -76.3422922 | - |
|  | CCSD(T)-full/aTZ//M06-2X/maTZ | -76.3575098 | - |
|  | CCSD(T)-full-6-31++g(3df,3pd)//M06-2X/maTZ | -76.3198742 | - |
|  | CCSD(T)-full-6-311++g(3df,3pd)//M06-2X/maTZ | -76.3613722 | - |
|  | CCSD(T)-full-6-311++g(3d2f,3pd)//M06-2X/maTZ | -76.3646 | - |
| NH_2_ | MP2/aTZ | -55.77663602 | 0.019445 |
|  | MP2/maTZ | -55.77299147 | 0.019484 |
|  | MP2/6-311++g(3df,3pd) | -55.77289958 | 0.019529 |
|  | M06-2X/aTZ | -55.87191893 | 0.019188 |
|  | M06-2X/maTZ | -55.87128624 | 0.019185 |
|  | M06-2X/6-11++g(3df,3pd) | -55.86977191 | 0.019226 |
|  | M06-HF/aTZ | -55.87815459 | 0.019297 |
|  | M06-HF/maTZ | -55.87678447 | 0.019289 |
|  | M06-HF/6-11++g(3df,3pd) | -55.87245244 | 0.019471 |
|  | B3LYP/aTZ | -55.90659194 | 0.018969 |
|  | B3LYP/maTZ | -55.90595459 | 0.018979 |
|  | B3LYP/6-311++g(3df,3pd) | -55.90512907 | 0.018992 |
|  | CASSCF(3,3) – MP2/aQZ//MP2/aTZ | -55.83538904 |  |
|  | CCSD(T)/CBS//MP2/aTZ | -55.82274568 | - |
|  | CCSD(T)/aQZ//MP2/aTZ | -55.8130683 | - |
|  | CCSD(T)/aTZ//MP2/aTZ | -55.7998067 | - |
|  | CCSD(T)-full/aTZ//MP2/aTZ | -55.8153641 | - |
|  | CCSD(T)-full-6-31++g(3df,3pd) //MP2/aTZ | -55.7905727 | - |
|  | CCSD(T)-full-6-311++g(3df,3pd) //MP2/aTZ | -55.8190105 | - |
|  | CCSD(T)-full-6-311++g(3d2f,3pd) //MP2/aTZ | -55.8210555 | - |
|  | CCSD(T)/aTZ//M06-2X/maTZ | -55.7998369 | - |
|  | CCSD(T)-full/aTZ//M06-2X/maTZ | -55.8153636 | - |
|  | CCSD(T)-full-6-31++g(3df,3pd)//M06-2X/maTZ | -55.7905544 | - |
|  | CCSD(T)-full-6-311++g(3df,3pd)//M06-2X/maTZ | -55.819029 | - |
|  | CCSD(T)-full-6-311++g(3d2f,3pd)//M06-2X/maTZ | -55.8210694 | - |
| H_2_NOH | MP2/aTZ | -131.52244983 | 0.040563 |
|  | MP2/maTZ | -131.51349151 | 0.040748 |
|  | MP2/6-311++g(3df,3pd) | -131.51302349 | 0.040849 |
|  | M06-2X/aTZ | -131.71874904 | 0.040846 |
|  | M06-2X/maTZ | -131.71784456 | 0.040896 |
|  | M06-2X/6-11++g(3df,3pd) | -131.71418810 | 0.041071 |
|  | M06-HF/aTZ | -131.74026428 | 0.041547 |
|  | M06-HF/maTZ | -131.73757339 | 0.041595 |
|  | M06-HF/6-11++g(3df,3pd) | -131.72789034 | 0.042297 |
|  | B3LYP/aTZ | -131.77943376 | 0.040168 |
|  | B3LYP/maTZ | -131.77859493 | 0.040205 |
|  | B3LYP/6-311++g(3df,3pd) | -131.77620867 | 0.040253 |
|  | CASSCF(9,9) – MP2/aQZ//MP2/aTZ |  |  |
|  | CCSD(T)/CBS//MP2/aTZ | -131.6126286 | - |
|  | CCSD(T)/aQZ//MP2/aTZ | -131.5872898 | - |
|  | CCSD(T)/aTZ//MP2/aTZ | -131.5525663 | - |
|  | CCSD(T)-full/aTZ//MP2/aTZ | -131.5845174 | - |
|  | CCSD(T)-full-6-31++g(3df,3pd) //MP2/aTZ | -131.5202375 | - |
|  | CCSD(T)-full-6-311++g(3df,3pd) //MP2/aTZ | -131.5901394 | - |
|  | CCSD(T)-full-6-311++g(3d2f,3pd) //MP2/aTZ | -131.5965687 | - |
|  | CCSD(T)/aTZ//M06-2X/maTZ | -131.5522425 | - |
|  | CCSD(T)-full/aTZ//M06-2X/maTZ | -131.5843329 | - |
|  | CCSD(T)-full-6-31++g(3df,3pd)//M06-2X/maTZ | -131.5200771 | - |
|  | CCSD(T)-full-6-311++g(3df,3pd)//M06-2X/maTZ | -131.59006 | - |
|  | CCSD(T)-full-6-311++g(3d2f,3pd)//M06-2X/maTZ | -131.5964356 | - |
| H_2_NNHOH | MP2/aTZ | -186.76167100 | 0.058185 |
|  | MP2/maTZ | -186.74866242 | 0.058375 |
|  | MP2/6-311++g(3df,3pd) | -186.74893183 | 0.058638 |
|  | M06-2X/aTZ | -187.04993738 | 0.058621 |
|  | M06-2X/maTZ | -187.04864242 | 0.058683 |
|  | M06-2X/6-11++g(3df,3pd) | -187.04412061 | 0.058880 |
|  | M06-HF/aTZ | -187.08290510 | 0.059533 |
|  | M06-HF/maTZ | -187.07875116 | 0.059659 |
|  | M06-HF/6-11++g(3df,3pd) | -187.06644346 | 0.060018 |
|  | B3LYP/aTZ | -187.13343564 | 0.057531 |
|  | B3LYP/maTZ | -187.13231994 | 0.057570 |
|  | B3LYP/6-311++g(3df,3pd) | -187.12924734 | 0.057677 |
|  | CASSCF(9,9) – MP2/aQZ//MP2/aTZ |  |  |
|  | CCSD(T)/CBS//MP2/aTZ | -186.889743 | - |
|  | CCSD(T)/aQZ//MP2/aTZ | -186.8540856 | - |
|  | CCSD(T)/aTZ//MP2/aTZ | -186.8052239 | - |
|  | CCSD(T)-full/aTZ//MP2/aTZ | -186.8548504 | - |
|  | CCSD(T)-full-6-31++g(3df,3pd) //MP2/aTZ | -186.7647206 | - |
|  | CCSD(T)-full-6-311++g(3df,3pd) //MP2/aTZ | -186.8631629 | - |
|  | CCSD(T)-full-6-311++g(3d2f,3pd) //MP2/aTZ | -186.8723218 | - |
|  | CCSD(T)/aTZ//M06-2X/maTZ | -186.8047453 | - |
|  | CCSD(T)-full/aTZ//M06-2X/maTZ | **-**186.8545879 | - |
|  | CCSD(T)-full-6-31++g(3df,3pd)//M06-2X/maTZ | -186.7644352 | - |
|  | CCSD(T)-full-6-311++g(3df,3pd)//M06-2X/maTZ | -186.8630272 | - |
|  | CCSD(T)-full-6-311++g(3d2f,3pd)//M06-2X/maTZ | -186.8721279 | - |
| H | MP2/aTZ | -0.49982118 | - |
|  | MP2/maTZ | -0.49980981 | - |
|  | MP2/6-311++g(3df,3pd) | -0.49981792 | - |
|  | M06-2X/aTZ | -0.49820646 | - |
|  | M06-2X/maTZ | -0.49813477 | - |
|  | M06-2X/6-11++g(3df,3pd) | -0.49819484 | - |
|  | M06-HF/aTZ | -0.49659997 | - |
|  | M06-HF/maTZ | -0.49617260 | - |
|  | M06-HF/6-11++g(3df,3pd) | -0.49672603 | - |
|  | B3LYP/aTZ | -0.50225968 | - |
|  | B3LYP/maTZ | -0.50215634 | - |
|  | B3LYP/6-311++g(3df,3pd) | -0.50225698 | - |
|  | CASSCF(1,3) – MP2/aQZ//MP2/aTZ | -0.49994832 |  |
|  | CCSD(T)/CBS//MP2/aTZ | -0.500041049 | - |
|  | CCSD(T)/aQZ | -0.4999483 | - |
|  | CCSD(T)/aTZ | -0.4998212 | - |
|  | CCSD(T)-full/aTZ | -0.4998212 | - |
|  | CCSD(T)-full-6-31++g(3df,3pd | -0.4992657 | - |
|  | CCSD(T)-full-6-311++g(3df,3pd) | -0.4998179 | - |
|  | CCSD(T)-full-6-311++g(3d2f,3pd) | -0.4998179 | - |
| MCr1a | MP2/aTZ | -187.31631057 | 0.066064 |
|  | MP2/maTZ | -187.30330421 | 0.066206 |
|  | MP2/6-311++g(3df,3pd) | -187.30382522 | 0.066508 |
|  | M06-2X/aTZ | -187.61050152 | 0.065820 |
|  | M06-2X/maTZ | -187.60915965 | 0.065856 |
|  | M06-2X/6-11++g(3df,3pd) | -187.60416582 | 0.066058 |
|  | M06-HF/aTZ | -187.64219363 | 0.066919 |
|  | M06-HF/maTZ | -187.63814711 | 0.066893 |
|  | M06-HF/6-311++g(3df,3pd) | - | - |
|  | B3LYP/aTZ | -187.70206147 | 0.064938 |
|  | B3LYP/maTZ | -187.70086214 | 0.064956 |
|  | B3LYP/6-311++g(3df,3pd) | -187.69770127 | 0.065098 |
|  | CASSCF(9,9) – MP2/aQZ//MP2/aTZ | -187.45788008 | - |
|  | CCSD(T)/CBS//MP2/aTZ | -187.4530462 | - |
|  | CCSD(T)/aQZ//MP2/aTZ | -187.417989 | - |
|  | CCSD(T)/TZ//MP2/aTZ | -187.3503123 | - |
|  | CCSD(T)/aTZ//MP2/aTZ | -187.3699477 | - |
|  | CCSD(T)/maTZ//MP2/aTZ | -187.3561473 | - |
|  | CCSD(T)-full/aTZ//MP2/aTZ | -187.4192979 | - |
|  | CCSD(T)-full/maTZ//MP2/aTZ | -187.3985696 | - |
|  | CCSD(T)/DZ//MP2/aTZ | -187.1451042 | - |
|  | CCSD(T)/aDZ//MP2/aTZ | -187.2054442 | - |
|  | CCSD(T)/maDZ//MP2/aTZ | -187.1690268 | - |
|  | CCSD(T)-full/aDZ//MP2/aTZ | -187.2126719 | - |
|  | CCSD(T)-full/maDZ//MP2/aTZ | -187.1756256 | - |
|  | CCSD(T)/6-31++g(2df,pd) //MP2/aTZ | -187.2758707 | - |
|  | CCSDT-full/6-31++g(2df,2p) //MP2/aTZ | -187.293665 | - |
|  | CCSD(T)-full-6-31++g(2df,3p) //MP2/aTZ | -187.2970985 | - |
|  | CCSD(T)-full-6-31++g(2df,pd) //MP2/aTZ | -187.3051492 | - |
|  | CCSD(T)-full-6-31++g(3df,2p) //MP2/aTZ | -187.3056662 | - |
|  | CCSD(T)-full-6-31++g(3df,3pd) //MP2/aTZ | -187.3288283 | - |
|  | CCSD(T)-full-6-31++g(3df,pd) //MP2/aTZ | -187.3204169 | - |
|  | CCSD(T)-full-6-31++g(df,2p) //MP2/aTZ | -187.2309143 | - |
|  | CCSD(T)-full-6-31++g(df,pd) //MP2/aTZ | -187.2393933 | - |
|  | CCSD(T)-full-6-311++g(2df,2p) //MP2/aTZ | -187.4016931 | - |
|  | CCSD(T)-full-6-311++g(3d2f,3pd) //MP2/aTZ | -187.4365351 | - |
|  | CCSD(T)-full-6-311++g(3df,2p) //MP2/aTZ | -187.4151089 | - |
|  | CCSD(T)-full-6-311++g(3df,3pd) //MP2/aTZ | -187.4280608 | - |
|  | CCSD(T)-full-6-311++g(df,2p) //MP2/aTZ | -187.3663817 | - |
|  | CCSD(T)/aTZ//M06-2X/maTZ | -187.3696868 | - |
|  | CCSD(T)-full/aTZ//M06-2X/maTZ | -187.4190466 | - |
|  | CCSD(T)-full-6-31++g(3df,3pd)//M06-2X/maTZ | -187.3284766 | - |
|  | CCSD(T)-full-6-311++g(3df,3pd)//M06-2X/maTZ | -187.4278029 | - |
|  | CCSD(T)-full-6-311++g(3d2f,3pd)//M06-2X/maTZ | -187.4362863 | - |
| MCr2 | MP2/aTZ | -187.23811830 | 0.064234 |
|  | MP2/maTZ | - | - |
|  | MP2/6-311++g(3df,3pd) | -187.22539468 | 0.064645 |
|  | M06-2X/aTZ | -187.52837015 | 0.064327 |
|  | M06-2X/maTZ | -187.52278723 | 0.063406 |
|  | M06-2X/6-11++g(3df,3pd) | -187.52233930 | 0.063559 |
|  | M06-HF/aTZ | -187.57028120 | 0.068704 |
|  | M06-HF/maTZ | -187.56007116 | 0.068358 |
|  | M06-HF/6-11++g(3df,3pd) | -187.55392222 | 0.068816 |
|  | B3LYP/aTZ | -187.62153044 | 0.062753 |
|  | B3LYP/maTZ | - | - |
|  | B3LYP/6-311++g(3df,3pd) | -187.61693159 | 0.062464 |
|  | CASSCF(9,9) – MP2/aQZ//MP2/aTZ | -187.36888861 | - |
|  | CCSD(T)/CBS//MP2/aTZ | -187.3703734 | - |
|  | CCSD(T)/aQZ//MP2/aTZ | -187.3346478 | - |
|  | CCSD(T)/aTZ//MP2/aTZ | -187.2856905 | - |
|  | CCSD(T)-full/aTZ//MP2/aTZ | -187.3369515 | - |
|  | CCSD(T)-full-6-31++g(3df,3pd) //MP2/aTZ | -187.2463318 | - |
|  | CCSD(T)-full-6-311++g(3df,3pd) //MP2/aTZ | -187.3440271 | - |
|  | CCSD(T)-full-6-311++g(3d2f,3pd) //MP2/aTZ | -187.3526908 | - |
|  | CCSD(T)/aTZ//M06-2X/maTZ | -187.2849706 | - |
|  | CCSD(T)-full/aTZ//M06-2X/maTZ | -187.3363907 | - |
|  | CCSD(T)-full-6-31++g(3df,3pd)//M06-2X/maTZ | -187.2457277 | - |
|  | CCSD(T)-full-6-311++g(3df,3pd)//M06-2X/maTZ | -187.3435424 | - |
|  | CCSD(T)-full-6-311++g(3d2f,3pd)//M06-2X/maTZ | -187.3521492 | - |
| MCp1 | MP2/aTZ | -187.37585762 | 0.065278 |
|  | MP2/maTZ | -187.36298811 | 0.065577 |
|  | MP2/6-311++g(3df,3pd) | -187.36406609 | 0.065677 |
|  | M06-2X/aTZ | -187.66824053 | 0.065282 |
|  | M06-2X/maTZ | -187.66694487 | 0.065347 |
|  | M06-2X/6-11++g(3df,3pd) | -187.66167866 | 0.065568 |
|  | M06-HF/aTZ | -187.69821375 | 0.066192 |
|  | M06-HF/maTZ | -187.69402897 | 0.065896 |
|  | M06-HF/6-11++g(3df,3pd) | -187.68001674 | 0.066631 |
|  | B3LYP/aTZ | -187.76049530 | 0.064296 |
|  | B3LYP/maTZ | -187.75936338 | 0.064372 |
|  | B3LYP/6-311++g(3df,3pd) | -187.75646353 | 0.064440 |
|  | CASSCF(9,9) – MP2/aQZ//MP2/aTZ | -187.49747637 |  |
|  | CCSD(T)/CBS//MP2/aTZ | -187.511328 | - |
|  | CCSD(T)/aQZ//MP2/aTZ | -187.4751178 | - |
|  | CCSD(T)/TZ//MP2/aTZ | -187.4044007 | - |
|  | CCSD(T)/aTZ//MP2/aTZ | -187.4254971 | - |
|  | CCSD(T)/maTZ//MP2/aTZ | -187.4118975 | - |
|  | CCSD(T)-full/aTZ//MP2/aTZ | -187.4748431 | - |
|  | CCSD(T)-full/maTZ//MP2/aTZ | -187.4548941 | - |
|  | CCSD(T)/DZ//MP2/aTZ | -187.1939199 | - |
|  | CCSD(T)/aDZ//MP2/aTZ | -187.2591822 | - |
|  | CCSD(T)/maDZ//MP2/aTZ | -187.2216969 | - |
|  | CCSD(T)-full/aDZ//MP2/aTZ | -187.2663889 | - |
|  | CCSD(T)-full/maDZ//MP2/aTZ | -187.2283508 | - |
|  | CCSD(T)/6-31++g(2df,pd) //MP2/aTZ | -187.3316862 | - |
|  | CCSDT-full/6-31++g(2df,2p) //MP2/aTZ | -187.3487688 | - |
|  | CCSD(T)-full-6-31++g(2df,3p) //MP2/aTZ | -187.3519787 | - |
|  | CCSD(T)-full-6-31++g(2df,pd) //MP2/aTZ | -187.361184 | - |
|  | CCSD(T)-full-6-31++g(3df,2p) //MP2/aTZ | -187.3606323 | - |
|  | CCSD(T)-full-6-31++g(3df,3pd) //MP2/aTZ | -187.3842641 | - |
|  | CCSD(T)-full-6-31++g(3df,pd) //MP2/aTZ | -187.3760457 | - |
|  | CCSD(T)-full-6-31++g(df,2p) //MP2/aTZ | -187.2839591 | - |
|  | CCSD(T)-full-6-31++g(df,pd) //MP2/aTZ | -187.2922149 | - |
|  | CCSD(T)-full-6-311++g(2df,2p) //MP2/aTZ | -187.4578221 | - |
|  | CCSD(T)-full-6-311++g(3d2f,3pd) //MP2/aTZ | -187.4929226 | - |
|  | CCSD(T)-full-6-311++g(3df,2p) //MP2/aTZ | -187.4715251 | - |
|  | CCSD(T)-full-6-311++g(3df,3pd) //MP2/aTZ | -187.4848035 | - |
|  | CCSD(T)-full-6-311++g(df,2p) //MP2/aTZ | -187.4231849 | - |
|  | CCSD(T)/aTZ//M06-2X/maTZ | -187.4255272 | - |
|  | CCSD(T)-full/aTZ//M06-2X/maTZ | -187.4748251 | - |
|  | CCSD(T)-full-6-31++g(3df,3pd)//M06-2X/maTZ | -187.3843164 | - |
|  | CCSD(T)-full-6-311++g(3df,3pd)//M06-2X/maTZ | -187.4848068 | - |
|  | CCSD(T)-full-6-311++g(3d2f,3pd)//M06-2X/maTZ | -187.4929347 | - |
| MCp2 | MP2/aTZ | -187.37562825 | 0.065165 |
|  | MP2/maTZ | -187.36272680 | 0.065421 |
|  | MP2/6-311++g(3df,3pd) | -187.36383315 | 0.065498 |
|  | M06-2X/aTZ | -187.66796179 | 0.064925 |
|  | M06-2X/maTZ | -187.66665290 | 0.065007 |
|  | M06-2X/6-11++g(3df,3pd) | -187.66137818 | 0.065314 |
|  | M06-HF/aTZ | -187.69792459 | 0.066039 |
|  | M06-HF/maTZ | -187.69375518 | 0.065732 |
|  | M06-HF/6-11++g(3df,3pd) | -187.67972242 | 0.066826 |
|  | B3LYP/aTZ | -187.76020024 | 0.064141 |
|  | B3LYP /maTZ | -187.75904353 | 0.064160 |
|  | B3LYP /6-311++g(3df,3pd) | -187.75615972 | 0.064317 |
|  | CASSCF(9,9) – MP2/aQZ//MP2/aTZ | -187.50641533 | - |
|  | CCSD(T)/CBS//MP2/aTZ | -187.511029 | - |
|  | CCSD(T)/aQZ//MP2/aTZ | -187.4748132 | - |
|  | CCSD(T)/TZ//MP2/aTZ | -187.4040086 | - |
|  | CCSD(T)/aTZ//MP2/aTZ | -187.4251842 | - |
|  | CCSD(T)/maTZ//MP2/aTZ | -187.411563 | - |
|  | CCSD(T)-full/aTZ//MP2/aTZ | -187.4745359 | - |
|  | CCSD(T)-full/maTZ//MP2/aTZ | -187.4545723 | - |
|  | CCSD(T)/DZ//MP2/aTZ | -187.1933621 | - |
|  | CCSD(T)/aDZ//MP2/aTZ | -187.2588751 | - |
|  | CCSD(T)/maDZ//MP2/aTZ | -187.2212737 | - |
|  | CCSD(T)-full/aDZ//MP2/aTZ | -187.266082 | - |
|  | CCSD(T)-full/maDZ//MP2/aTZ | -187.2279277 | - |
|  | CCSD(T)/6-31++g(2df,pd) //MP2/aTZ | -187.331312 | - |
|  | CCSDT-full/6-31++g(2df,2p) //MP2/aTZ | -187.3484459 | - |
|  | CCSD(T)-full-6-31++g(2df,3p) //MP2/aTZ | -187.3516491 | - |
|  | CCSD(T)-full-6-31++g(2df,pd) //MP2/aTZ | -187.3608457 | - |
|  | CCSD(T)-full-6-31++g(3df,2p) //MP2/aTZ | -187.3602738 | - |
|  | CCSD(T)-full-6-31++g(3df,3pd) //MP2/aTZ | -187.3838757 | - |
|  | CCSD(T)-full-6-31++g(3df,pd) //MP2/aTZ | -187.3756729 | - |
|  | CCSD(T)-full-6-31++g(df,2p) //MP2/aTZ | -187.2835817 | - |
|  | CCSD(T)-full-6-31++g(df,pd) //MP2/aTZ | -187.2918026 | - |
|  | CCSD(T)-full-6-311++g(2df,2p) //MP2/aTZ | -187.4575121 | - |
|  | CCSD(T)-full-6-311++g(3d2f,3pd) //MP2/aTZ | -187.4926017 | - |
|  | CCSD(T)-full-6-311++g(3df,2p) //MP2/aTZ | -187.4712252 | - |
|  | CCSD(T)-full-6-311++g(3df,3pd) //MP2/aTZ | -187.4844994 | - |
|  | CCSD(T)-full-6-311++g(df,2p) //MP2/aTZ | -187.4228162 | - |
|  | CCSD(T)/aTZ//M06-2X/maTZ | -187.4251707 | - |
|  | CCSD(T)-full/aTZ//M06-2X/maTZ | -187.4744826 | - |
|  | CCSD(T)-full-6-31++g(3df,3pd)//M06-2X/maTZ | -187.3838571 | - |
|  | CCSD(T)-full-6-311++g(3df,3pd)//M06-2X/maTZ | -187.4844796 | - |
|  | CCSD(T)-full-6-311++g(3d2f,3pd)//M06-2X/maTZ | -187.49259 | - |
| MCp3a | MP2/aTZ | -187.30398199 | 0.061837 |
|  | MP2/maTZ | -187.29109557 | 0.062029 |
|  | MP2/6-311++g(3df,3pd) | -187.29089275 | 0.062280 |
|  | M06-2X/aTZ | -187.59584575 | 0.062343 |
|  | M06-2X/maTZ | -187.59443617 | 0.062479 |
|  | M06-2X/6-11++g(3df,3pd) | -187.58937379 | 0.062355 |
|  | M06-HF/aTZ | -187.62294600 | 0.062684 |
|  | M06-HF/maTZ | -187.61899793 | 0.062712 |
|  | M06-HF/6-11++g(3df,3pd) | -187.60539787 | 0.063128 |
|  | B3LYP/aTZ | -187.68964187 | 0.061132 |
|  | B3LYP/maTZ | -187.68827841 | 0.061049 |
|  | B3LYP/6-311++g(3df,3pd) | -187.68512852 | 0.061278 |
|  | CASSCF(9,9) – MP2/aQZ//MP2/aTZ |  |  |
|  | CCSD(T)/CBS//MP2/aTZ | -187.4401569 | - |
|  | CCSD(T)/aQZ//MP2/aTZ | -187.4051857 | - |
|  | CCSD(T)/aTZ//MP2/aTZ | -187.3572622 | - |
|  | CCSD(T)-full/aTZ//MP2/aTZ | -187.4052554 | - |
|  | CCSD(T)-full-6-31++g(3df,3pd) //MP2/aTZ | -187.3165764 | - |
|  | CCSD(T)-full-6-311++g(3df,3pd) //MP2/aTZ | -187.4142498 | - |
|  | CCSD(T)-full-6-311++g(3d2f,3pd) //MP2/aTZ | -187.4227853 | - |
|  | CCSD(T)/aTZ//M06-2X/maTZ | -187.3568757 | - |
|  | CCSD(T)-full/aTZ//M06-2X/maTZ | -187.4050132 | - |
|  | CCSD(T)-full-6-31++g(3df,3pd)//M06-2X/maTZ | -187.3163769 | - |
|  | CCSD(T)-full-6-311++g(3df,3pd)//M06-2X/maTZ | -187.4141195 | - |
|  | CCSD(T)-full-6-311++g(3d2f,3pd)//M06-2X/maTZ | -187.422618 | - |
| MCp3b | MP2/aTZ | -187.30649897 | 0.062726 |
|  | MP2/maTZ | -187.29348131 | 0.062908 |
|  | MP2/6-311++g(3df,3pd) | -187.29342011 | 0.063157 |
|  | M06-2X/aTZ | -187.59818117 | 0.063411 |
|  | M06-2X/maTZ | -187.59688141 | 0.063461 |
|  | M06-2X/6-11++g(3df,3pd) | -187.59185100 | 0.063692 |
|  | M06-HF/aTZ | -187.62544094 | 0.063463 |
|  | M06-HF/maTZ | -187.62147007 | 0.063511 |
|  | M06-HF/6-11++g(3df,3pd) | -187.60833886 | 0.064043 |
|  | B3LYP/aTZ | -187.69107520 | 0.061638 |
|  | B3LYP/maTZ | -187.68992825 | 0.061702 |
|  | B3LYP/6-311++g(3df,3pd) | -187.68668434 | 0.061864 |
|  | CASSCF(9,9) – MP2/aQZ//MP2/aTZ | -187.44389009 |  |
|  | CCSD(T)/CBS//MP2/aTZ | -187.4424354 | - |
|  | CCSD(T)/aQZ//MP2/aTZ | -187.4075493 | - |
|  | CCSD(T)/aTZ//MP2/aTZ | -187.359742 | - |
|  | CCSD(T)-full/aTZ//MP2/aTZ | -187.4078929 | - |
|  | CCSD(T)-full-6-31++g(3df,3pd) //MP2/aTZ | -187.3186802 | - |
|  | CCSD(T)-full-6-311++g(3df,3pd) //MP2/aTZ | -187.4168302 | - |
|  | CCSD(T)-full-6-311++g(3d2f,3pd) //MP2/aTZ | -187.4253821 | - |
|  | CCSD(T)/aTZ//M06-2X/maTZ | -187.3593861 | - |
|  | CCSD(T)-full/aTZ//M06-2X/maTZ | -187.4076578 | - |
|  | CCSD(T)-full-6-31++g(3df,3pd)//M06-2X/maTZ | -187.3184362 | - |
|  | CCSD(T)-full-6-311++g(3df,3pd)//M06-2X/maTZ | -187.4167029 | - |
|  | CCSD(T)-full-6-311++g(3d2f,3pd)//M06-2X/maTZ | -187.4252139 | - |
| MCP4 | MP2/aTZ | -187.26194243 | 0.058644 |
|  | MP2/maTZ | -187.24866754 | 0.058680 |
|  | MP2/6-311++g(3df,3pd) | -187.24914792 | 0.059083 |
|  | M06-2X/aTZ | -187.54838178 | 0.060082 |
|  | M06-2X/maTZ | -187.54661807 | 0.059881 |
|  | M06-2X/6-11++g(3df,3pd) | -187.54235729 | 0.059332 |
|  | M06-HF/aTZ | - | - |
|  | M06-HF/maTZ | - | - |
|  | M06-HF/6-11++g(3df,3pd) | - | - |
|  | B3LYP/aTZ | -187.63651688 | 0.058818 |
|  | B3LYP/maTZ | -187.63511186 | 0.058642 |
|  | B3LYP/6-311++g(3df,3pd) | -187.63236973 | 0.058980 |
|  | CCSD(T)/CBS//MP2/aTZ | -187.3904206 | - |
|  | CCSD(T)/aQZ//MP2/aTZ | -187.354691 | - |
|  | CASSCF(9,9) – MP2/aQZ//MP2/aTZ | -187.39907201 |  |
|  | CCSD(T)/aTZ//MP2/aTZ | -187.305728 | - |
|  | CCSD(T)-full/aTZ//MP2/aTZ | -187.3554496 | - |
|  | CCSD(T)-full-6-31++g(3df,3pd) //MP2/aTZ | -187.2646761 | - |
|  | CCSD(T)-full-6-311++g(3df,3pd) //MP2/aTZ | -187.3636121 | - |
|  | CCSD(T)-full-6-311++g(3d2f,3pd) //MP2/aTZ | -187.3727459 | - |
|  | CCSD(T)/aTZ//M06-2X/maTZ | -187.3048571 | - |
|  | CCSD(T)-full/aTZ//M06-2X/maTZ | -187.3548152 | - |
|  | CCSD(T)-full-6-31++g(3df,3pd)//M06-2X/maTZ | -187.264004 | - |
|  | CCSD(T)-full-6-311++g(3df,3pd)//M06-2X/maTZ | -187.3629812 | - |
|  | CCSD(T)-full-6-311++g(3d2f,3pd)//M06-2X/maTZ | -187.3720783 | - |
| TS2 | MP2/aTZ | -187.29881847 | 0.061405 |
|  | MP2/maTZ | -187.28569665 | 0.061549 |
|  | MP2/6-311++g(3df,3pd) | -187.28632439 | 0.061776 |
|  | M06-2X/aTZ | -187.60222642 | 0.063597 |
|  | M06-2X/maTZ | -187.60073821 | 0.063783 |
|  | M06-2X/6-11++g(3df,3pd) | -187.59582994 | 0.063681 |
|  | M06-HF/aTZ | -187.62550171 | 0.063098 |
|  | M06-HF/maTZ | -187.62103377 | 0.063094 |
|  | M06-HF/6-11++g(3df,3pd) | - | - |
|  | B3LYP/aTZ | -187.69651512 | 0.062085 |
|  | B3LYP/maTZ | -187.69538400 | 0.062133 |
|  | B3LYP/6-311++g(3df,3pd) | -187.69202476 | 0.062154 |
|  | CASSCF(9,9) – MP2/aQZ//MP2/aTZ | -187.41846649 |  |
|  | CCSD(T)/CBS//MP2/aTZ | -187.445397 | - |
|  | CCSD(T)/aQZ//MP2/aTZ | -187.4100991 | - |
|  | CCSD(T)/TZ//MP2/aTZ | -187.3401126 | - |
|  | CCSD(T)/aTZ//MP2/aTZ | -187.3617275 | - |
|  | CCSD(T)/maTZ//MP2/aTZ | -187.3475416 | - |
|  | CCSD(T)-full/aTZ//MP2/aTZ | -187.4109249 | - |
|  | CCSD(T)-full/maTZ//MP2/aTZ | -187.3900243 | - |
|  | CCSD(T)/DZ//MP2/aTZ | -187.1317438 | - |
|  | CCSD(T)/aDZ//MP2/aTZ | -187.1969612 | - |
|  | CCSD(T)/maDZ//MP2/aTZ | -187.1585287 | - |
|  | CCSD(T)-full/aDZ//MP2/aTZ | -187.204205 | - |
|  | CCSD(T)-full/maDZ//MP2/aTZ | -187.1651152 | - |
|  | CCSD(T)/6-31++g(2df,pd) //MP2/aTZ | -187.2670132 | - |
|  | CCSDT-full/6-31++g(2df,2p) //MP2/aTZ | -187.2843442 | - |
|  | CCSD(T)-full-6-31++g(2df,3p) //MP2/aTZ | -187.2881454 | - |
|  | CCSD(T)-full-6-31++g(2df,pd) //MP2/aTZ | -187.2961922 | - |
|  | CCSD(T)-full-6-31++g(3df,2p) //MP2/aTZ | -187.297094 | - |
|  | CCSD(T)-full-6-31++g(3df,3pd) //MP2/aTZ | -187.3210661 | - |
|  | CCSD(T)-full-6-31++g(3df,pd) //MP2/aTZ | -187.3124719 | - |
|  | CCSD(T)-full-6-31++g(df,2p) //MP2/aTZ | -187.2207325 | - |
|  | CCSD(T)-full-6-31++g(df,pd) //MP2/aTZ | -187.2291141 | - |
|  | CCSD(T)-full-6-311++g(2df,2p) //MP2/aTZ | -187.3924348 | - |
|  | CCSD(T)-full-6-311++g(3d2f,3pd) //MP2/aTZ | -187.4281681 | - |
|  | CCSD(T)-full-6-311++g(3df,2p) //MP2/aTZ | -187.4060438 | - |
|  | CCSD(T)-full-6-311++g(3df,3pd) //MP2/aTZ | -187.4197074 | - |
|  | CCSD(T)-full-6-311++g(df,2p) //MP2/aTZ | -187.3581079 | - |
|  | CCSD(T)/aTZ//M06-2X/maTZ | -187.3602201 | - |
|  | CCSD(T)-full/aTZ//M06-2X/maTZ | -187.4094412 | - |
|  | CCSD(T)-full-6-31++g(3df,3pd)//M06-2X/maTZ | -187.3191429 | - |
|  | CCSD(T)-full-6-311++g(3df,3pd)//M06-2X/maTZ | -187.4180679 | - |
|  | CCSD(T)-full-6-311++g(3d2f,3pd)//M06-2X/maTZ | -187.4267434 | - |
| TS3 | MP2/aTZ | -187.29827608 | 0.061358 |
|  | MP2/maTZ | -187.28534072 | 0.061577 |
|  | MP2/6-311++g(3df,3pd) | -187.28579920 | 0.061695 |
|  | M06-2X/aTZ | -187.60158149 | 0.063081 |
|  | M06-2X/maTZ | -187.59586849 | 0.062615 |
|  | M06-2X/6-11++g(3df,3pd) | -187.59510183 | 0.063238 |
|  | M06-HF/aTZ | -187.62584106 | 0.062275 |
|  | M06-HF/maTZ | -187.62177639 | 0.062708 |
|  | M06-HF/6-11++g(3df,3pd) | -187.60843770 | 0.063093 |
|  | B3LYP/aTZ | -187.69839085 | 0.063179 |
|  | B3LYP/maTZ | -187.69703721 | 0.063190 |
|  | B3LYP/6-311++g(3df,3pd) | -187.69390061 | 0.063235 |
|  | CASSCF(9,9) – MP2/aQZ//MP2/aTZ | -187.41665115 |  |
|  | CCSD(T)/CBS//MP2/aTZ | -187.4442964 | - |
|  | CCSD(T)/aQZ//MP2/aTZ | -187.4089645 | - |
|  | CCSD(T)/TZ//MP2/aTZ | -187.3393691 | - |
|  | CCSD(T)/aTZ//MP2/aTZ | -187.3605467 | - |
|  | CCSD(T)/maTZ//MP2/aTZ | -187.3465933 | - |
|  | CCSD(T)-full/aTZ//MP2/aTZ | -187.4096834 | - |
|  | CCSD(T)-full/maTZ//MP2/aTZ | -187.3890834 | - |
|  | CCSD(T)/DZ//MP2/aTZ | -187.1312219 | - |
|  | CCSD(T)/aDZ//MP2/aTZ | -187.1956511 | - |
|  | CCSD(T)/maDZ//MP2/aTZ | -187.1575494 | - |
|  | CCSD(T)-full/aDZ//MP2/aTZ | -187.2029063 | - |
|  | CCSD(T)-full/maDZ//MP2/aTZ | -187.1641269 | - |
|  | CCSD(T)/6-31++g(2df,pd) //MP2/aTZ | -187.266144 | - |
|  | CCSDT-full/6-31++g(2df,2p) //MP2/aTZ | -187.2831509 | - |
|  | CCSD(T)-full-6-31++g(2df,3p) //MP2/aTZ | -187.2870035 | - |
|  | CCSD(T)-full-6-31++g(2df,pd) //MP2/aTZ | -187.2953558 | - |
|  | CCSD(T)-full-6-31++g(3df,2p) //MP2/aTZ | -187.2956638 | - |
|  | CCSD(T)-full-6-31++g(3df,3pd) //MP2/aTZ | -187.3200246 | - |
|  | CCSD(T)-full-6-31++g(3df,pd) //MP2/aTZ | -187.3114025 | - |
|  | CCSD(T)-full-6-31++g(df,2p) //MP2/aTZ | -187.2197075 | - |
|  | CCSD(T)-full-6-31++g(df,pd) //MP2/aTZ | -187.2282179 | - |
|  | CCSD(T)-full-6-311++g(2df,2p) //MP2/aTZ | -187.3912625 | - |
|  | CCSD(T)-full-6-311++g(3d2f,3pd) //MP2/aTZ | -187.4269636 | - |
|  | CCSD(T)-full-6-311++g(3df,2p) //MP2/aTZ | -187.4048579 | - |
|  | CCSD(T)-full-6-311++g(3df,3pd) //MP2/aTZ | -187.4186061 | - |
|  | CCSD(T)-full-6-311++g(df,2p) //MP2/aTZ | -187.3572122 | - |
|  | CCSD(T)/aTZ//M06-2X/maTZ | -187.355444 | - |
|  | CCSD(T)-full/aTZ//M06-2X/maTZ | -187.4045714 | - |
|  | CCSD(T)-full-6-31++g(3df,3pd)//M06-2X/maTZ | -187.314378 | - |
|  | CCSD(T)-full-6-311++g(3df,3pd)//M06-2X/maTZ | -187.4133814 | - |
|  | CCSD(T)-full-6-311++g(3d2f,3pd)//M06-2X/maTZ | -187.4219233 | - |
| TS1 | MP2/aTZ | -187.26224225 | 0.065979 |
|  | MP2/maTZ | -187.24824562 | 0.066250 |
|  | MP2/6-311++g(3df,3pd) | -187.24734782 | 0.066329 |
|  | M06-2X/aTZ | -187.56343119 | 0.064633 |
|  | M06-2X/maTZ | -187.56194737 | 0.064697 |
|  | M06-2X/6-11++g(3df,3pd) | -187.55620146 | 0.064896 |
|  | M06-HF/aTZ | -187.58834823 | 0.065972 |
|  | M06-HF/maTZ | -187.58344934 | 0.066057 |
|  | M06-HF/6-11++g(3df,3pd) | -187.57033869 | 0.066764 |
|  | B3LYP/aTZ | -187.66868863 | 0.063319 |
|  | B3LYP/maTZ | -187.66746062 | 0.063363 |
|  | B3LYP/6-311++g(3df,3pd) | -187.66358863 | 0.063465 |
|  | CASSCF(9,9) – MP2/aQZ//MP2/aTZ | -187.41134884 |  |
|  | CCSD(T)/CBS//MP2/aTZ | -187.4079726 | - |
|  | CCSD(T)/aQZ//MP2/aTZ | -187.372944 | - |
|  | CCSD(T)/aTZ//MP2/aTZ | -187.324943 | - |
|  | CCSD(T)-full/aTZ//MP2/aTZ | -187.373696 | - |
|  | CCSD(T)-full-6-31++g(3df,3pd) //MP2/aTZ | -187.2840157 | - |
|  | CCSD(T)-full-6-311++g(3df,3pd) //MP2/aTZ | -187.3801439 | - |
|  | CCSD(T)-full-6-311++g(3d2f,3pd) //MP2/aTZ | -187.3904279 | - |
|  | CCSD(T)/aTZ//M06-2X/maTZ | -187.3295799 | - |
|  | CCSD(T)-full/aTZ//M06-2X/maTZ | -187.3779008 | - |
|  | CCSD(T)-full-6-31++g(3df,3pd)//M06-2X/maTZ | -187.2882936 | - |
|  | CCSD(T)-full-6-311++g(3df,3pd)//M06-2X/maTZ | -187.3841195 | - |
|  | CCSD(T)-full-6-311++g(3d2f,3pd)//M06-2X/maTZ | -187.3945264 | - |
| TS1b | MP2/aTZ | -187.20843327 | 0.060160 |
|  | MP2/maTZ | - | - |
|  | MP2/6-311++g(3df,3pd) | -187.19436348 | 0.060744 |
|  | M06-2X/aTZ | -187.50556339 | 0.060803 |
|  | M06-2X/maTZ | -187.50022850 | 0.060263 |
|  | M06-2X/6-11++g(3df,3pd) | -187.49699915 | 0.060561 |
|  | M06-HF/aTZ | -187.53728139 | 0.063127 |
|  | M06-HF/maTZ | -187.52868528 | 0.061938 |
|  | M06-HF/6-11++g(3df,3pd) | -187.52007460 | 0.062386 |
|  | B3LYP/aTZ | -187.60187483 | 0.059026 |
|  | B3LYP/maTZ | - | - |
|  | B3LYP/6-311++g(3df,3pd) | -187.59613467 | 0.058797 |
|  | CASSCF(9,9) – MP2/aQZ//MP2/aTZ |  |  |
|  | CCSD(T)/CBS//MP2/aTZ | -187.3482886 | - |
|  | CCSD(T)/aQZ//MP2/aTZ | -187.3132118 | - |
|  | CCSD(T)/aTZ//MP2/aTZ | -187.265144 | - |
|  | CCSD(T)-full/aTZ//MP2/aTZ | -187.3147242 | - |
|  | CCSD(T)-full-6-31++g(3df,3pd) //MP2/aTZ | -187.2240239 | - |
|  | CCSD(T)-full-6-311++g(3df,3pd) //MP2/aTZ | -187.3214681 | - |
|  | CCSD(T)-full-6-311++g(3d2f,3pd) //MP2/aTZ | -187.3306289 | - |
|  | CCSD(T)/aTZ//M06-2X/maTZ | -187.2645737 | - |
|  | CCSD(T)-full/aTZ//M06-2X/maTZ | -187.3141624 | - |
|  | CCSD(T)-full-6-31++g(3df,3pd)//M06-2X/maTZ | -187.223494 | - |
|  | CCSD(T)-full-6-311++g(3df,3pd)//M06-2X/maTZ | -187.321003 | - |
|  | CCSD(T)-full-6-311++g(3d2f,3pd)//M06-2X/maTZ | -187.3301366 | - |
| TS1c | MP2/aTZ | -187.22483569 | 0.062799 |
|  | MP2/maTZ | -187.20941852 | 0.062265 |
|  | MP2/6-311++g(3df,3pd) | -187.21257997 | 0.064498 |
|  | M06-2X/aTZ | -187.51376777 | 0.060625 |
|  | M06-2X/maTZ | -187.51118724 | 0.060760 |
|  | M06-2X/6-11++g(3df,3pd) | -187.50737586 | 0.060803 |
|  | M06-HF/aTZ | -187.54442829 | 0.063986 |
|  | M06-HF/maTZ | -187.53659125 | 0.063912 |
|  | M06-HF/6-11++g(3df,3pd) | -187.52741980 | 0.063935 |
|  | B3LYP/aTZ | -187.61105084 | 0.059294 |
|  | B3LYP/maTZ | -187.60913766 | 0.059276 |
|  | B3LYP/6-311++g(3df,3pd) | -187.60678072 | 0.059210 |
|  | CASSCF(9,9) – MP2/aQZ//MP2/aTZ | -187.35082411 |  |
|  | CCSD(T)/CBS//MP2/aTZ | -187.3575697 | - |
|  | CCSD(T)/aQZ//MP2/aTZ | -187.3223664 | - |
|  | CCSD(T)/aTZ//MP2/aTZ | -187.274125 | - |
|  | CCSD(T)-full/aTZ//MP2/aTZ | -187.3236673 | - |
|  | CCSD(T)-full-6-31++g(3df,3pd) //MP2/aTZ | -187.232972 | - |
|  | CCSD(T)-full-6-311++g(3df,3pd) //MP2/aTZ | -187.3400638 | - |
|  | CCSD(T)-full-6-311++g(3d2f,3pd) //MP2/aTZ | -187.3400638 | - |
|  | CCSD(T)/aTZ//M06-2X/maTZ | -187.2744861 | - |
|  | CCSD(T)-full/aTZ//M06-2X/maTZ | -187.3237783 | - |
|  | CCSD(T)-full-6-31++g(3df,3pd)//M06-2X/maTZ | -187.2329275 | - |
|  | CCSD(T)-full-6-311++g(3df,3pd)//M06-2X/maTZ | -187.3309883 | - |
|  | CCSD(T)-full-6-311++g(3d2f,3pd)//M06-2X/maTZ | -187.3404149 | - |
| TS4 | MP2/aTZ | -187.23418041 | 0.061199 |
|  | MP2/maTZ | -187.21962295 | 0.061702 |
|  | MP2/6-311++g(3df,3pd) | -187.22063417 | 0.061563 |
|  | M06-2X/aTZ | -187.52859670 | 0.060174 |
|  | M06-2X/maTZ | -187.52649299 | 0.060282 |
|  | M06-2X/6-11++g(3df,3pd) | -187.52205166 | 0.060400 |
|  | M06-HF/aTZ | -187.55667409 | 0.061447 |
|  | M06-HF/maTZ | -187.55104343 | 0.061603 |
|  | M06-HF/6-11++g(3df,3pd) | -187.53958979 | 0.062074 |
|  | B3LYP/aTZ | -187.62697898 | 0.058886 |
|  | B3LYP/maTZ | -187.62525182 | 0.058944 |
|  | B3LYP/6-311++g(3df,3pd) | -187.62245562 | 0.059037 |
|  | CASSCF(9,9) – MP2/aQZ//MP2/aTZ | -187.35928327 |  |
|  | CCSD(T)/CBS//MP2/aTZ | -187.3716868 | - |
|  | CCSD(T)/aQZ//MP2/aTZ | -187.3361501 | - |
|  | CCSD(T)/aTZ//MP2/aTZ | -187.287452 | - |
|  | CCSD(T)-full/aTZ//MP2/aTZ | -187.3374275 | - |
|  | CCSD(T)-full-6-31++g(3df,3pd) //MP2/aTZ | -187.2464213 | - |
|  | CCSD(T)-full-6-311++g(3df,3pd) //MP2/aTZ | -187.3444643 | - |
|  | CCSD(T)-full-6-311++g(3d2f,3pd) //MP2/aTZ | -187.3539621 | - |
|  | CCSD(T)/aTZ//M06-2X/maTZ | -187.2892845 | - |
|  | CCSD(T)-full/aTZ//M06-2X/maTZ | -187.3390115 | - |
|  | CCSD(T)-full-6-31++g(3df,3pd)//M06-2X/maTZ | -187.2479216 | - |
|  | CCSD(T)-full-6-311++g(3df,3pd)//M06-2X/maTZ | -187.3459806 | - |
|  | CCSD(T)-full-6-311++g(3d2f,3pd)//M06-2X/maTZ | -187.3556016 | - |

**Table S4**. The electronic ($E_{elec}$) and zero point (ZPE) energies (in Hartree) of all species at different levels.

| Method | ΔΕ | ${\Delta E}_{f}^{\neq}$ | ${\Delta E}_{r}^{\neq}$ | $\Delta_{r}E$ |
| --- | --- | --- | --- | --- |
|  | TS2 | TS2 | TS2 | P1 |
| MP2/aTZ | 2.01 | 8.05 | 45.91 | -37.44 |
| MP2/maTZ | 2.48 | 8.13 | 45.97 | -37.33 |
| MP2/6-311++g(3df,3pd) | 1.94 | 8.01 | 46.34 | -37.97 |
| M06-2X/aTZ | -2.46 | 3.80 | 40.37 | -36.41 |
| M06-2X/maTZ | -2.32 | 3.98 | 40.56 | -36.33 |
| M06-2X/6-11++g(3df,3pd) | -2.72 | 3.74 | 40.14 | -36.33 |
| M06-HF/aTZ | 1.52 | 8.08 | 43.69 | -35.66 |
| M06-HF/maTZ | 1.88 | 8.35 | 44.05 | -35.52 |
| M06-HF/6-11++g(3df,3pd) | - | - | - | -35.28 |
| B3LYP/aTZ | -3.71 | 1.69 | 38.76 | -37.67 |
| B3LYP/maTZ | -3.84 | 1.67 | 38.74 | -37.58 |
| B3LYP/6-311++g(3df,3pd) | -3.87 | 1.71 | 39.00 | -37.90 |
| CASSCF(9,9) – MP2/aQZ//MP2/aTZ | 1.68 | 24.73 | 49.58 | -52.47 |
| CCSD(T)/CBS//MP2/aTZ | -3.08 | 4.80 | 41.37 | -36.15 |
| CCSD(T)/aQZ//MP2/aTZ | -3.07 | 4.95 | 40.80 | -35.54 |
| CCSD(T)/TZ//MP2/aTZ | -2.38 | 6.40 | 40.34 | -33.58 |
| CCSD(T)/aTZ//MP2/aTZ | -3.06 | 5.16 | 40.02 | -34.72 |
| CCSD(T)/maTZ//MP2/aTZ | -2.29 | 5.40 | 40.38 | -34.63 |
| CCSD(T)-full/aTZ//MP2/aTZ | -3.83 | 5.25 | 40.11 | -35.12 |
| CCSD(T)-full/maTZ//MP2/aTZ | -2.65 | 5.36 | 40.71 | -34.94 |
| CCSD(T)/DZ//MP2/aTZ | -1.59 | 8.38 | 39.02 | -30.56 |
| CCSD(T)/aDZ//MP2/aTZ | -3.05 | 5.32 | 39.04 | -33.88 |
| CCSD(T)/maDZ//MP2/aTZ | -1.02 | 6.59 | 39.64 | -32.91 |
| CCSD(T)-full/aDZ//MP2/aTZ | -3.17 | 5.31 | 39.02 | -33.89 |
| CCSD(T)-full/maDZ//MP2/aTZ | -1.05 | 6.60 | 39.68 | -32.95 |
| CCSD(T)/6-31++g(2df,pd) //MP2/aTZ | -2.76 | 5.56 | 40.58 | -26.09 |
| CCSDT-full/6-31++g(2df,2p) //MP2/aTZ | -2.70 | 5.85 | 40.43 | -34.41 |
| CCSD(T)-full-6-31++g(2df,3p) //MP2/aTZ | -3.26 | 5.62 | 40.06 | -34.21 |
| CCSD(T)-full-6-31++g(2df,pd) //MP2/aTZ | -3.12 | 5.62 | 40.78 | -34.97 |
| CCSD(T)-full-6-31++g(3df,2p) //MP2/aTZ | -3.27 | 5.38 | 39.87 | -34.75 |
| CCSD(T)-full-6-31++g(3df,3pd) //MP2/aTZ | -3.90 | 4.87 | 39.66 | -34.99 |
| CCSD(T)-full-6-31++g(3df,pd) //MP2/aTZ | -3.43 | 4.99 | 39.89 | -35.03 |
| CCSD(T)-full-6-31++g(df,2p) //MP2/aTZ | -2.15 | 6.39 | 39.68 | -33.06 |
| CCSD(T)-full-6-31++g(df,pd) //MP2/aTZ | -2.64 | 6.45 | 39.60 | -33.01 |
| CCSD(T)-full-6-311++g(2df,2p) //MP2/aTZ | -2.25 | 5.81 | 41.03 | -34.82 |
| CCSD(T)-full-6-311++g(3d2f,3pd) //MP2/aTZ | -6.36 | 5.25 | 40.63 | -38.31 |
| CCSD(T)-full-6-311++g(3df,2p) //MP2/aTZ | -2.48 | 5.69 | 41.09 | -35.17 |
| CCSD(T)-full-6-311++g(3df,3pd) //MP2/aTZ | -0.14 | 5.24 | 40.85 | -32.40 |
| CCSD(T)-full-6-311++g(df,2p) //MP2/aTZ | -2.65 | 5.19 | 40.84 | -35.35 |
| CCSD(T)/aTZ//M06-2X/maTZ | -2.24 | 5.94 | 40.98 | -34.84 |
| CCSD(T)-full/aTZ//M06-2X/maTZ | -2.98 | 6.03 | 41.03 | -35.19 |
| CCSD(T)-full-6-31++g(3df,3pd)//M06-2X/maTZ | -2.87 | 5.86 | 40.90 | -35.16 |
| CCSD(T)-full-6-311++g(3df,3pd)//M06-2X/maTZ | -2.27 | 6.11 | 41.88 | -35.56 |
| CCSD(T)-full-6-311++g(3d2f,3pd)//M06-2X/maTZ | -2.47 | 5.99 | 41.54 | -35.31 |
|  | TS3 | TS3 | TS3 | - |
| MP2/aTZ | 2.32 | 8.36 | 46.15 | - |
| MP2/maTZ | 2.72 | 8.37 | 46.15 | - |
| MP2/6-311++g(3df,3pd) | 2.21 | 8.29 | 46.58 | - |
| M06-2X/aTZ | -2.38 | 3.88 | 40.50 | - |
| M06-2X/maTZ | 0.00 | 6.31 | 42.92 | - |
| M06-2X/6-11++g(3df,3pd) | -2.54 | 3.92 | 40.29 | - |
| M06-HF/aTZ | 0.79 | 7.35 | 42.87 | - |
| M06-HF/maTZ | 1.17 | 7.65 | 43.27 | - |
| M06-HF/6-11++g(3df,3pd) | - |  |  | - |
| B3LYP/aTZ | -4.20 | 1.20 | 38.18 | - |
| B3LYP/maTZ | -4.22 | 1.29 | 38.30 | - |
| B3LYP/6-311++g(3df,3pd) | -4.37 | 1.22 | 38.39 | - |
| CASSCF(9,9) – MP2/aQZ//MP2/aTZ | 2.82 | 25.87 | 56.33 | - |
| CCSD(T)/CBS//MP2/aTZ | -2.39 | 5.49 | 41.88 | - |
| CCSD(T)/aQZ//MP2/aTZ | -2.36 | 5.66 | 41.32 | - |
| CCSD(T)/TZ//MP2/aTZ | -1.92 | 6.87 | 40.56 | - |
| CCSD(T)/aTZ//MP2/aTZ | -2.32 | 5.90 | 40.56 | - |
| CCSD(T)/maTZ//MP2/aTZ | -1.70 | 6.00 | 40.77 | - |
| CCSD(T)-full/aTZ//MP2/aTZ | -3.05 | 6.03 | 40.70 | - |
| CCSD(T)-full/maTZ//MP2/aTZ | -2.06 | 5.95 | 41.09 | - |
| CCSD(T)/DZ//MP2/aTZ | -1.26 | 8.71 | 38.99 | - |
| CCSD(T)/aDZ//MP2/aTZ | -2.22 | 6.15 | 39.67 | - |
| CCSD(T)/maDZ//MP2/aTZ | -0.41 | 7.20 | 39.99 | - |
| CCSD(T)-full/aDZ//MP2/aTZ | -2.36 | 6.13 | 39.64 | - |
| CCSD(T)-full/maDZ//MP2/aTZ | -0.43 | 7.22 | 40.04 | - |
| CCSD(T)/6-31++g(2df,pd) //MP2/aTZ | -2.21 | 6.10 | 40.89 | - |
| CCSDT-full/6-31++g(2df,2p) //MP2/aTZ | -1.95 | 6.60 | 40.97 | - |
| CCSD(T)-full-6-31++g(2df,3p) //MP2/aTZ | -2.54 | 6.33 | 40.57 | - |
| CCSD(T)-full-6-31++g(2df,pd) //MP2/aTZ | -2.59 | 6.15 | 41.10 | - |
| CCSD(T)-full-6-31++g(3df,2p) //MP2/aTZ | -2.37 | 6.28 | 40.54 | - |
| CCSD(T)-full-6-31++g(3df,3pd) //MP2/aTZ | -3.24 | 5.52 | 40.07 | - |
| CCSD(T)-full-6-31++g(3df,pd) //MP2/aTZ | -2.76 | 5.66 | 40.33 | - |
| CCSD(T)-full-6-31++g(df,2p) //MP2/aTZ | -1.51 | 7.03 | 40.08 | - |
| CCSD(T)-full-6-31++g(df,pd) //MP2/aTZ | -2.08 | 7.01 | 39.90 | - |
| CCSD(T)-full-6-311++g(2df,2p) //MP2/aTZ | -1.52 | 6.55 | 41.57 | - |
| CCSD(T)-full-6-311++g(3d2f,3pd) //MP2/aTZ | -5.61 | 6.01 | 41.19 | - |
| CCSD(T)-full-6-311++g(3df,2p) //MP2/aTZ | -1.73 | 6.43 | 41.65 | - |
| CCSD(T)-full-6-311++g(3df,3pd) //MP2/aTZ | 0.55 | 5.93 | 41.35 | - |
| CCSD(T)-full-6-311++g(df,2p) //MP2/aTZ | -2.09 | 5.75 | 41.17 | - |
| CCSD(T)/aTZ//M06-2X/maTZ | 0.75 | 8.94 | 43.75 | - |
| CCSD(T)-full/aTZ//M06-2X/maTZ | 0.08 | 9.08 | 43.87 | - |
| CCSD(T)-full-6-31++g(3df,3pd)//M06-2X/maTZ | 0.12 | 8.85 | 43.60 | - |
| CCSD(T)-full-6-311++g(3df,3pd)//M06-2X/maTZ | 0.67 | 9.05 | 44.61 | - |
| CCSD(T)-full-6-311++g(3d2f,3pd)//M06-2X/maTZ | 0.56 | 9.01 | 44.34 | - |
|  | TS1 | TS1 | TS1 | P2 |
| MP2/aTZ | 27.84 | 33.88 | 28.79 | 0.97 |
| MP2/maTZ | 28.93 | 34.58 | 29.54 | 1.16 |
| MP2/6-311++g(3df,3pd) | 29.25 | 35.33 | 29.87 | 1.31 |
| M06-2X/aTZ | 22.53 | 28.79 | 21.78 | 2.56 |
| M06-2X/maTZ | 22.60 | 28.90 | 21.78 | 2.64 |
| M06-2X/6-11++g(3df,3pd) | 22.91 | 29.37 | 22.41 | 2.61 |
| M06-HF/aTZ | 26.64 | 33.19 | 23.77 | 4.55 |
| M06-HF/maTZ | 27.32 | 33.80 | 24.41 | 4.68 |
| M06-HF/6-11++g(3df,3pd) | 26.83 |  | 24.28 | 4.87 |
| B3LYP/aTZ | 14.52 | 19.93 | 14.52 | 1.02 |
| B3LYP/maTZ | 14.45 | 19.96 | 14.52 | 1.11 |
| B3LYP/6-311++g(3df,3pd) | 14.80 | 20.38 | 14.89 | 1.01 |
| CASSCF(9,9) – MP2/aQZ//MP2/aTZ | 6.14 | 29.20 |  |  |
| CCSD(T)/CBS//MP2/aTZ | 20.41 | 28.28 | 20.20 | 3.21 |
| CCSD(T)/aQZ//MP2/aTZ | 20.25 | 28.27 | 20.23 | 3.04 |
| CCSD(T)/aTZ//MP2/aTZ | 20.02 | 28.24 | 20.28 | 2.81 |
| CCSD(T)-full/aTZ//MP2/aTZ | 19.53 | 28.62 | 19.80 | 3.10 |
| CCSD(T)-full-6-31++g(3df,3pd) //MP2/aTZ | 19.35 | 28.12 | 20.43 | 2.54 |
| CCSD(T)-full-6-311++g(3df,3pd) //MP2/aTZ | 24.69 | 30.07 | 21.40 | 6.49 |
| CCSD(T)-full-6-311++g(3d2f,3pd) //MP2/aTZ | 17.32 | 28.93 | 20.30 | 0.25 |
| CCSD(T)/aTZ//MP2/aTZ | 16.98 | 25.17 | 17.13 | 2.86 |
| CCSD(T)-full/aTZ//MP2/aTZ | 16.82 | 25.82 | 17.01 | 3.14 |
| CCSD(T)-full-6-31++g(3df,3pd)//M06-2X/maTZ | 16.48 | 25.22 | 17.62 | 2.47 |
| CCSD(T)-full-6-311++g(3df,3pd)//M06-2X/maTZ | 19.03 | 27.41 | 18.83 | 3.36 |
| CCSD(T)-full-6-311++g(3d2f,3pd)//M06-2X/maTZ | 17.75 | 26.20 | 17.63 | 3.33 |
|  | TS1b | TS1b | TS1b | - |
| MP2/aTZ | 57.95 | 16.07 | 59.93 | - |
| MP2/maTZ |  |  |  | - |
| MP2/6-311++g(3df,3pd) | 58.99 | 17.02 | 60.64 | - |
| M06-2X/aTZ | 56.44 | 12.10 | 56.48 | - |
| M06-2X/maTZ | 58.54 | 12.18 | 58.64 | - |
| M06-2X/6-11++g(3df,3pd) | 57.34 | 14.02 | 57.56 | - |
| M06-HF/aTZ | 56.90 | 17.21 | 55.11 | - |
| M06-HF/maTZ | 59.10 | 15.67 | 57.24 | - |
| M06-HF/6-11++g(3df,3pd) | 55.62 | 17.20 | 54.35 | - |
| B3LYP/aTZ | 53.76 | 10.00 | 54.34 | - |
| B3LYP/maTZ |  |  |  | - |
| B3LYP/6-311++g(3df,3pd) | 54.20 | 10.75 | 54.90 | - |
| CASSCF(9,9) – MP2/aQZ//MP2/aTZ |  |  |  | - |
| CCSD(T)/CBS//MP2/aTZ | 57.86 | 13.86 | 59.08 | - |
| CCSD(T)/aQZ//MP2/aTZ | 57.73 | 13.45 | 59.20 | - |
| CCSD(T)/aTZ//MP2/aTZ | 57.55 | 12.89 | 59.36 | - |
| CCSD(T)-full/aTZ//MP2/aTZ | 56.54 | 13.95 | 58.46 | - |
| CCSD(T)-full-6-31++g(3df,3pd) //MP2/aTZ | 57.00 | 14.00 | 59.40 | - |
| CCSD(T)-full-6-311++g(3df,3pd) //MP2/aTZ | 61.51 | 14.16 | 59.84 | - |
| CCSD(T)-full-6-311++g(3d2f,3pd) //MP2/aTZ | 54.84 | 13.84 | 59.46 | - |
| CCSD(T)/aTZ//MP2/aTZ | 57.77 | 12.80 | 59.50 | - |
| CCSD(T)-full/aTZ//MP2/aTZ | 56.81 | 13.95 | 58.67 | - |
| CCSD(T)-full-6-31++g(3df,3pd)//M06-2X/maTZ | 57.15 | 13.95 | 59.58 | - |
| CCSD(T)-full-6-311++g(3df,3pd)//M06-2X/maTZ | 58.64 | 14.14 | 60.05 | - |
| CCSD(T)-full-6-311++g(3d2f,3pd)//M06-2X/maTZ | 58.16 | 13.81 | 59.66 | - |
|  | TS1c | TS1c | TS1c | - |
| MP2/aTZ | 49.31 | 55.35 | 51.29 | - |
| MP2/maTZ | 50.79 | 56.44 | 52.35 | - |
| MP2/6-311++g(3df,3pd) | 49.92 | 56.00 | 51.57 | - |
| M06-2X/aTZ | 51.18 | 57.44 | 51.22 | - |
| M06-2X/maTZ | 51.98 | 58.28 | 52.08 | - |
| M06-2X/6-11++g(3df,3pd) | 50.98 | 57.44 | 51.20 | - |
| M06-HF/aTZ | 52.95 | 59.51 | 51.16 | - |
| M06-HF/maTZ | 55.38 | 61.86 | 53.51 | - |
| M06-HF/6-11++g(3df,3pd) | 51.98 |  | 50.71 | - |
| B3LYP/aTZ | 48.17 | 53.57 | 48.75 | - |
| B3LYP/maTZ | 48.48 | 53.99 | 49.17 | - |
| B3LYP/6-311++g(3df,3pd) | 47.78 | 53.36 | 48.47 | - |
| CASSCF(9,9) – MP2/aQZ//MP2/aTZ | 44.12 | 67.18 |  | - |
| CCSD(T)/CBS//MP2/aTZ | 52.04 | 59.91 | 53.25 | - |
| CCSD(T)/aQZ//MP2/aTZ | 51.98 | 60.00 | 53.45 | - |
| CCSD(T)/aTZ//MP2/aTZ | 51.91 | 60.13 | 53.73 | - |
| CCSD(T)-full/aTZ//MP2/aTZ | 50.93 | 60.01 | 52.85 | - |
| CCSD(T)-full-6-31++g(3df,3pd) //MP2/aTZ | 51.38 | 60.15 | 53.78 | - |
| CCSD(T)-full-6-311++g(3df,3pd) //MP2/aTZ | 49.84 | 55.22 | 48.17 | - |
| CCSD(T)-full-6-311++g(3d2f,3pd) //MP2/aTZ | 48.92 | 60.54 | 53.54 | - |
| CCSD(T)/aTZ//MP2/aTZ | 51.55 | 59.74 | 53.28 | - |
| CCSD(T)-full/aTZ//MP2/aTZ | 50.78 | 59.78 | 52.64 | - |
| CCSD(T)-full-6-31++g(3df,3pd)//M06-2X/maTZ | 51.23 | 59.96 | 53.66 | - |
| CCSD(T)-full-6-311++g(3df,3pd)//M06-2X/maTZ | 52.37 | 60.75 | 53.79 | - |
| CCSD(T)-full-6-311++g(3d2f,3pd)//M06-2X/maTZ | 51.71 | 60.16 | 53.21 | - |
|  | TS4 | TS4 | TS4 | P3 |
| MP2/aTZ | 42.45 | 48.48 | 19.02 | 23.42 |
| MP2/maTZ | 44.03 | 49.68 | 20.12 | 23.84 |
| MP2/6-311++g(3df,3pd) | 43.02 | 49.10 | 19.45 | 23.54 |
| M06-2X/aTZ | 41.59 | 47.85 | 12.47 | 28.35 |
| M06-2X/maTZ | 42.07 | 48.38 | 12.88 | 28.34 |
| M06-2X/6-11++g(3df,3pd) | 41.52 | 47.98 | 13.41 | 27.85 |
| M06-HF/aTZ | 43.67 | 50.23 | - | 28.15 |
| M06-HF/maTZ | 44.86 | 51.34 | - | 28.66 |
| M06-HF/6-11++g(3df,3pd) | 43.18 | - | - | 27.09 |
| B3LYP/aTZ | 37.92 | 43.32 | 6.03 | 31.60 |
| B3LYP/maTZ | 38.16 | 43.67 | 6.38 | 31.51 |
| B3LYP/6-311++g(3df,3pd) | 37.83 | 43.41 | 6.26 | 31.30 |
| CASSCF(9,9) – MP2/aQZ//MP2/aTZ | 38.81 | 61.87 | 24.97 |  |
| CCSD(T)/CBS//MP2/aTZ | 43.18 | 51.05 | 11.76 | 31.82 |
| CCSD(T)/aQZ//MP2/aTZ | 43.33 | 51.35 | 11.63 | 32.11 |
| CCSD(T)/aTZ//MP2/aTZ | 43.55 | 51.77 | 11.47 | 32.51 |
| CCSD(T)-full/aTZ//MP2/aTZ | 42.29 | 51.37 | 11.31 | 31.47 |
| CCSD(T)-full-6-31++g(3df,3pd) //MP2/aTZ | 42.94 | 51.71 | 11.46 | 31.92 |
| CCSD(T)-full-6-311++g(3df,3pd) //MP2/aTZ | 47.08 | 52.46 | 12.02 | 35.46 |
| CCSD(T)-full-6-311++g(3d2f,3pd) //MP2/aTZ | 40.20 | 51.82 | 11.79 | 28.79 |
| CCSD(T)/aTZ//MP2/aTZ | 42.27 | 50.45 | 9.77 | 32.68 |
| CCSD(T)-full/aTZ//MP2/aTZ | 41.22 | 50.22 | 9.92 | 31.56 |
| CCSD(T)-full-6-31++g(3df,3pd)//M06-2X/maTZ | 41.82 | 50.55 | 10.09 | 31.92 |
| CCSD(T)-full-6-311++g(3df,3pd)//M06-2X/maTZ | 42.96 | 51.34 | 10.67 | 32.38 |
| CCSD(T)-full-6-311++g(3d2f,3pd)//M06-2X/maTZ | 42.18 | 50.63 | 10.34 | 31.92 |

**Table S5**. The relative energies (ΔΕ), forward barrier heights (${\Delta E}_{f}^{\neq}$), reverse barrier heights (${\Delta E}_{r}^{\neq}$), and reaction energies ($\Delta_{r}E$) of the N_2_H_4_ + OH reaction. (Units of all numbers are kcal mol^-1^)

| Species | method | $E_{elec}$ | ZPE | ΔΕ + ZPE | ΔΕ |
| --- | --- | --- | --- | --- | --- |
| N_2_H_4_ | B3lyp/6-311g(d,p) | -111.9015329 | 0.053273 |  |  |
|  | CCSD(T)/6-311++g(d,p) | -111.6269886 |  |  |  |
| OH | B3lyp/6-311g(d,p) | -75.75452736 | 0.00844 |  |  |
|  | CCSD(T)/6-311++g(d,p) | -75.5964607 |  |  |  |
| R (N_2_H_4_+OH) | B3lyp/6-311g(d,p) | -187.6560603 | 0.061713 | 0.00 | 0.00 |
|  | CCSD(T)/6-311++g(d,p) | -187.2234493 |  | 0.00 | 0.00 |
| MCr | B3lyp/6-311g(d,p) | -187.6735771 | 0.065735 | -8.47 | -10.99 |
|  | CCSD(T)/6-311++g(d,p) | -187.2355139 |  | -5.05 | -7.57 |
| TS1 | B3lyp/6-311g(d,p) | -187.6384119 | 0.06353 | 12.21 | 11.07 |
|  | CCSD(T)/6-311++g(d,p) | -187.1909782 |  | 21.52 | 20.38 |
| TS2 | B3lyp/6-311g(d,p) | -187.6653373 | 0.062505 | -5.32 | -5.82 |
|  | CCSD(T)/6-311++g(d,p) | -187.2236376 |  | 0.38 | -0.12 |
| TS3 | B3lyp/6-311g(d,p) | -187.6670334 | 0.063425 | -5.81 | -6.89 |
|  | CCSD(T)/6-311++g(d,p) | -187.2209322 |  | 2.65 | 1.58 |
| MCp1 | B3lyp/6-311g(d,p) | -187.7293128 | 0.065135 | -43.82 | -45.97 |
|  | CCSD(T)/6-311++g(d,p) | -187.2889992 |  | -38.99 | -41.13 |
| MCp2 | B3lyp/6-311g(d,p) | -187.7288404 | 0.065033 | -43.59 | -45.67 |
|  | CCSD(T)/6-311++g(d,p) | -187.2886241 |  | -38.81 | -40.90 |
| MCp3a | B3lyp/6-311g(d,p) | -187.6579113 | 0.06117 | -1.50 | -1.16 |
|  | CCSD(T)/6-311++g(d,p) | -187.2213499 |  | 0.98 | 1.32 |
| P1(N_2_H_3_+H_2_O) | B3lyp/6-311g(d,p) | -187.7115705 | 0.060746 | -35.44 | -34.83 |
|  | CCSD(T)/6-311++g(d,p) | -187.2765674 |  | -33.94 | -33.33 |
| P2(NH_2_+H_2_NOH) | B3lyp/6-311g(d,p) | -187.6518307 | 0.059166 | 1.06 | 2.65 |
|  | CCSD(T)/6-311++g(d,p) | -187.2167379 |  | 2.61 | 4.21 |

**Table S6**. Calculated electronic energies ($E_{elec}$) and zero point energies (ZPE) (in Hartree), and relative energies (ΔΕ) (in kcal mol^-1^) of the reaction of N_2_H_4_ + OH at the B3lyp/6-311G(d,p) and CCSD(T)/6-311++G(d,p)//B3lyp/6-311G(d,p) levels.

| T/K | H/km | P/mbar | k^a^ | [OH]/molecule cm^-3^ | τ^b^ | ν^c^ |
| --- | --- | --- | --- | --- | --- | --- |
| 290.2 | 0 | 1013 | 6.25E-13 | 3.00E+06 | 5.33E+05 | 86.85 |
| 250.2 | 5 | 495.9 | 1.07E-12 | 1.00E+06 | 9.32E+05 | 97.03 |
| 215.6 | 10 | 242.8 | 2.44E-12 | 5.70E+05 | 7.20E+05 | 97.36 |
| 198.0 | 15 | 118.8 | 3.45E-12 | 4.20E+05 | 6.91E+05 | 119.01 |
| 208.0 | 20 | 58.18 | 1.20E-12 | 3.70E+05 | 2.26E+06 | 247.98 |
| 216.1 | 25 | 28.48 | 4.66E-13 | 6.60E+05 | 3.25E+06 | 501.88 |
| 221.5 | 30 | 13.94 | 2.08E-13 | 1.60E+06 | 3.00E+06 | 970.71 |
| 228.1 | 35 | 6.826 | 8.82E-14 | 3.70E+06 | 3.06E+06 | 1935.14 |
| 240.5 | 40 | 3.341 | 2.96E-14 | 6.80E+06 | 4.98E+06 | 4333.27 |
| 251.9 | 45 | 1.636 | 9.99E-15 | 8.50E+06 | 1.18E+07 | 10150.10 |
| 253.7 | 50 | 0.801 | 5.16E-15 | 6.80E+06 | 2.85E+07 | 18974.56 |

The mentioned altitude (H), pressure (P), the temperature (T), and the OH concentration ([OH]) in this Table are from reference 75.

^a^k is the bimolecular rate constant at the mentioned temperature and pressure.

^b^τ = $\frac{1}{k[OH]}$ is the lifetime of N_2_H_4_ in the atmospheric concentration of OH.

^c^ν is the ratio of $\frac{k_{\infty}}{k}$

**Table S7**. The rate constant of the N_2_H_4_ + OH reaction for path1, the concentration of hydroxyl radical in different altitudes, and lifetimes of N_2_H_4_ in the atmosphere in an ambient of atmospheric hydroxyl radical as functions of height.

| T/K | H/km | P/mbar | k^a^ | [OH]/molecule cm^-3^ | τ^b^ | ν^c^ |
| --- | --- | --- | --- | --- | --- | --- |
| 290.2 | 0 | 1013 | 2.20E-12 | 3.00E+06 | 1.51E+05 | 2.85E+01 |
| 250.2 | 5 | 495.9 | 2.96E-12 | 1.00E+06 | 3.38E+05 | 2.56E+01 |
| 215.6 | 10 | 242.8 | 4.73E-12 | 5.70E+05 | 3.71E+05 | 5.97E+00 |
| 198.0 | 15 | 118.8 | 5.67E-12 | 4.20E+05 | 4.20E+05 | 4.47E+01 |
| 208.0 | 20 | 58.18 | 2.60E-12 | 3.70E+05 | 1.04E+06 | 7.17E+01 |
| 216.1 | 25 | 28.48 | 1.26E-12 | 6.60E+05 | 1.20E+06 | 7.60E+01 |
| 221.5 | 30 | 13.94 | 6.58E-13 | 1.60E+06 | 9.50E+05 | 1.43E+02 |
| 228.1 | 35 | 6.826 | 3.25E-13 | 3.70E+06 | 8.32E+05 | 2.73E+02 |
| 240.5 | 40 | 3.341 | 1.30E-13 | 6.80E+06 | 1.13E+06 | 6.22E+02 |
| 251.9 | 45 | 1.636 | 5.34E-14 | 8.50E+06 | 2.20E+06 | 1.40E+03 |
| 253.7 | 50 | 0.801 | 3.00E-14 | 6.80E+06 | 4.91E+06 | 2.48E+03 |

The mentioned altitude (H), pressure (P), the temperature (T), and the OH concentration ([OH]) in this table are from reference 75.

^a^k is the bimolecular rate constant at mentioned temperature and pressure.

^b^τ = $\frac{1}{k[OH]}$ is the lifetime of N_2_H_4_ in the atmospheric concentration of OH.

^c^ν is the ratio of $\frac{k_{\infty}}{k}$.

**Table S8**. The rate constant of the N_2_H_4_ + OH reaction for path 2, the concentration of hydroxyl radical in different altitudes, and lifetimes of N_2_H_4_ in the atmosphere in an ambient of atmospheric hydroxyl radical as functions of height.

|  | CCSD(T)/6-311++G(d,p)//B3LYP/6-311G(d,p) | CCSD(T)/6-311++G(d,p)//B3LYP/6-311G(d,p) | CCSD(T)/6-311++G(d,p)//B3LYP/6-311G(d,p) | | CCSD(T)/6-311++G(d,p)//B3LYP/6-311G(d,p) | | |
| --- | --- | --- | --- | --- | --- | --- | --- |
| T/K | k_TST_Path1 | k_VTST_Path 1 | k^Eckart^ _TST_ Path 2 | k^Shavit^ _TST_ Path 2 | | k^Eckart^ _VTST_ Path 2 | k^Shavit^ _VTST_ Path 2 |
| 230 | 1.73E-12 | 5.94E-16 | 2.28E-14 | 2.33E-14 | | 2.27E-14 | 2.32E-14 |
| 232 | 1.73E-12 | 6.29E-16 | 2.35E-14 | 2.40E-14 | | 2.34E-14 | 2.39E-14 |
| 240 | 1.76E-12 | 7.85E-16 | 2.64E-14 | 2.69E-14 | | 2.63E-14 | 2.69E-14 |
| 250 | 1.79E-12 | 1.02E-15 | 3.02E-14 | 3.08E-14 | | 3.02E-14 | 3.08E-14 |
| 260 | 1.83E-12 | 1.29E-15 | 3.44E-14 | 3.50E-14 | | 3.43E-14 | 3.50E-14 |
| 270 | 1.87E-12 | 1.62E-15 | 3.88E-14 | 3.96E-14 | | 3.88E-14 | 3.95E-14 |
| 280 | 1.92E-12 | 2.00E-15 | 4.36E-14 | 4.44E-14 | | 4.35E-14 | 4.43E-14 |
| 290 | 1.97E-12 | 2.45E-15 | 4.86E-14 | 4.95E-14 | | 4.86E-14 | 4.95E-14 |
| 298 | 2.01E-12 | 2.84E-15 | 5.29E-14 | 5.39E-14 | | 5.29E-14 | 5.38E-14 |
| 298.15 | 2.01E-12 | 2.85E-15 | 5.30E-14 | 5.40E-14 | | 5.29E-14 | 5.39E-14 |
| 300 | 2.02E-12 | 2.95E-15 | 5.40E-14 | 5.50E-14 | | 5.40E-14 | 5.49E-14 |
| 310 | 2.07E-12 | 3.52E-15 | 5.98E-14 | 6.08E-14 | | 5.97E-14 | 6.07E-14 |
| 320 | 2.13E-12 | 4.17E-15 | 6.58E-14 | 6.69E-14 | | 6.57E-14 | 6.68E-14 |
| 330 | 2.19E-12 | 4.89E-15 | 7.22E-14 | 7.33E-14 | | 7.21E-14 | 7.32E-14 |
| 340 | 2.25E-12 | 5.69E-15 | 7.89E-14 | 8.01E-14 | | 7.88E-14 | 8.00E-14 |
| 350 | 2.32E-12 | 6.58E-15 | 8.59E-14 | 8.72E-14 | | 8.58E-14 | 8.71E-14 |
| 360 | 2.39E-12 | 7.56E-15 | 9.33E-14 | 9.47E-14 | | 9.32E-14 | 9.46E-14 |
| 374 | 2.49E-12 | 9.09E-15 | 1.04E-13 | 1.06E-13 | | 1.04E-13 | 1.06E-13 |
| 380 | 2.53E-12 | 9.80E-15 | 1.09E-13 | 1.11E-13 | | 1.09E-13 | 1.11E-13 |
| 390 | 2.61E-12 | 1.11E-14 | 1.18E-13 | 1.19E-13 | | 1.17E-13 | 1.19E-13 |
| 400 | 2.68E-12 | 1.24E-14 | 1.26E-13 | 1.28E-13 | | 1.26E-13 | 1.28E-13 |
| 410 | 2.76E-12 | 1.39E-14 | 1.36E-13 | 1.37E-13 | | 1.35E-13 | 1.37E-13 |
| 424 | 2.88E-12 | 1.62E-14 | 1.49E-13 | 1.51E-13 | | 1.49E-13 | 1.51E-13 |
| 430 | 2.93E-12 | 1.73E-14 | 1.55E-13 | 1.57E-13 | | 1.55E-13 | 1.57E-13 |
| 440 | 3.02E-12 | 1.91E-14 | 1.65E-13 | 1.68E-13 | | 1.65E-13 | 1.67E-13 |
| 450 | 3.11E-12 | 2.11E-14 | 1.76E-13 | 1.78E-13 | | 1.76E-13 | 1.78E-13 |
| 460 | 3.20E-12 | 2.32E-14 | 1.87E-13 | 1.90E-13 | | 1.87E-13 | 1.89E-13 |
| 470 | 3.29E-12 | 2.54E-14 | 1.99E-13 | 2.01E-13 | | 1.99E-13 | 2.01E-13 |
| 480 | 3.39E-12 | 2.78E-14 | 2.11E-13 | 2.13E-13 | | 2.10E-13 | 2.13E-13 |
| 490 | 3.49E-12 | 3.03E-14 | 2.23E-13 | 2.26E-13 | | 2.23E-13 | 2.25E-13 |
| 500 | 3.59E-12 | 3.29E-14 | 2.36E-13 | 2.38E-13 | | 2.35E-13 | 2.38E-13 |
| 510 | 3.69E-12 | 3.57E-14 | 2.49E-13 | 2.52E-13 | | 2.49E-13 | 2.51E-13 |
| 520 | 3.80E-12 | 3.87E-14 | 2.62E-13 | 2.65E-13 | | 2.62E-13 | 2.65E-13 |
| 530 | 3.91E-12 | 4.18E-14 | 2.76E-13 | 2.79E-13 | | 2.76E-13 | 2.79E-13 |
| 540 | 4.02E-12 | 4.51E-14 | 2.91E-13 | 2.94E-13 | | 2.91E-13 | 2.94E-13 |
| 550 | 4.13E-12 | 4.85E-14 | 3.06E-13 | 3.09E-13 | | 3.05E-13 | 3.09E-13 |
| 560 | 4.25E-12 | 5.21E-14 | 3.21E-13 | 3.24E-13 | | 3.21E-13 | 3.24E-13 |
| 570 | 4.36E-12 | 5.59E-14 | 3.37E-13 | 3.40E-13 | | 3.36E-13 | 3.40E-13 |
| 580 | 4.48E-12 | 5.99E-14 | 3.53E-13 | 3.56E-13 | | 3.53E-13 | 3.56E-13 |
| 590 | 4.61E-12 | 6.40E-14 | 3.70E-13 | 3.73E-13 | | 3.69E-13 | 3.73E-13 |
| 600 | 4.73E-12 | 6.83E-14 | 3.87E-13 | 3.90E-13 | | 3.86E-13 | 3.90E-13 |
| 610 | 4.86E-12 | 7.28E-14 | 4.04E-13 | 4.08E-13 | | 4.04E-13 | 4.08E-13 |
| 620 | 4.99E-12 | 7.76E-14 | 4.22E-13 | 4.26E-13 | | 4.22E-13 | 4.26E-13 |
| 630 | 5.12E-12 | 8.25E-14 | 4.41E-13 | 4.45E-13 | | 4.40E-13 | 4.44E-13 |
| 637 | 5.22E-12 | 8.60E-14 | 4.54E-13 | 4.58E-13 | | 4.54E-13 | 4.57E-13 |
| 640 | 5.26E-12 | 8.76E-14 | 4.60E-13 | 4.64E-13 | | 4.59E-13 | 4.63E-13 |
| 650 | 5.39E-12 | 9.29E-14 | 4.79E-13 | 4.83E-13 | | 4.79E-13 | 4.83E-13 |
| 660 | 5.53E-12 | 9.84E-14 | 4.99E-13 | 5.03E-13 | | 4.99E-13 | 5.03E-13 |
| 670 | 5.68E-12 | 1.04E-13 | 5.19E-13 | 5.24E-13 | | 5.19E-13 | 5.23E-13 |
| 680 | 5.82E-12 | 1.10E-13 | 5.40E-13 | 5.45E-13 | | 5.40E-13 | 5.44E-13 |
| 690 | 5.97E-12 | 1.16E-13 | 5.62E-13 | 5.66E-13 | | 5.61E-13 | 5.66E-13 |
| 700 | 6.12E-12 | 1.23E-13 | 5.83E-13 | 5.88E-13 | | 5.83E-13 | 5.88E-13 |
| 800 | 7.76E-12 | 2.00E-13 | 8.31E-13 | 8.36E-13 | | 8.30E-13 | 8.36E-13 |
| 900 | 9.67E-12 | 3.05E-13 | 1.13E-12 | 1.14E-12 | | 1.13E-12 | 1.14E-12 |
| 1000 | 1.19E-11 | 4.41E-13 | 1.49E-12 | 1.50E-12 | | 1.49E-12 | 1.50E-12 |
| 1100 | 1.44E-11 | 6.12E-13 | 1.91E-12 | 1.92E-12 | | 1.91E-12 | 1.92E-12 |
| 1200 | 1.72E-11 | 8.23E-13 | 2.40E-12 | 2.41E-12 | | 2.40E-12 | 2.41E-12 |
| 1300 | 2.03E-11 | 1.08E-12 | 2.95E-12 | 2.96E-12 | | 2.95E-12 | 2.96E-12 |
| 1400 | 2.38E-11 | 1.38E-12 | 3.58E-12 | 3.59E-12 | | 3.58E-12 | 3.59E-12 |
| 1500 | 2.76E-11 | 1.73E-12 | 4.28E-12 | 4.29E-12 | | 4.28E-12 | 4.29E-12 |
| 1600 | 3.18E-11 | 2.14E-12 | 5.05E-12 | 5.07E-12 | | 5.05E-12 | 5.07E-12 |
| 1700 | 3.64E-11 | 2.60E-12 | 5.91E-12 | 5.93E-12 | | 5.91E-12 | 5.93E-12 |
| 1800 | 4.13E-11 | 3.13E-12 | 6.85E-12 | 6.87E-12 | | 6.85E-12 | 6.87E-12 |
| 1900 | 4.66E-11 | 3.72E-12 | 7.88E-12 | 7.90E-12 | | 7.88E-12 | 7.90E-12 |
| 2000 | 5.24E-11 | 4.38E-12 | 8.99E-12 | 9.02E-12 | | 8.99E-12 | 9.02E-12 |
| 2100 | 5.85E-11 | 5.10E-12 | 1.02E-11 | 1.02E-11 | | 1.02E-11 | 1.02E-11 |
| 2200 | 6.51E-11 | 5.90E-12 | 1.15E-11 | 1.15E-11 | | 1.15E-11 | 1.15E-11 |
| 2300 | 7.22E-11 | 6.78E-12 | 1.29E-11 | 1.29E-11 | | 1.29E-11 | 1.29E-11 |
| 2400 | 7.96E-11 | 7.74E-12 | 1.44E-11 | 1.44E-11 | | 1.44E-11 | 1.44E-11 |
| 2500 | 8.75E-11 | 8.77E-12 | 1.60E-11 | 1.60E-11 | | 1.59E-11 | 1.59E-11 |
| 2600 | 9.59E-11 | 9.90E-12 | 1.77E-11 | 1.77E-11 | | 1.73E-11 | 1.74E-11 |
| 2700 | 1.05E-10 | 1.11E-11 | 1.95E-11 | 1.95E-11 | | 1.89E-11 | 1.89E-11 |
| 2800 | 1.14E-10 | 1.24E-11 | 2.14E-11 | 2.14E-11 | | 2.05E-11 | 2.06E-11 |
| 2900 | 1.24E-10 | 1.38E-11 | 2.34E-11 | 2.34E-11 | | 2.22E-11 | 2.23E-11 |
| 3000 | 1.34E-10 | 1.53E-11 | 2.55E-11 | 2.55E-11 | | 2.40E-11 | 2.41E-11 |

**Table S9**. High pressure limit rate constants calculated at the CCSD(T)/6-311++G(d,p)//B3LYP/6-311G(d,p) levels for path1 and path 2.

|  | CCSD(T)/6-311++G(d,p)//B3LYP/6-311G(d,p) | | CCSD(T)/6-311++G(d,p)//B3LYP/6-311G(d,p) | |
| --- | --- | --- | --- | --- |
| T/K | k^Eckart^ _TST_ Path 1+ Path 2 | k^Shavit^ _TST_ Path 1+ Path 2 | k^Eckart^ _VTST_ Path 1+ Path 2 | k^Shavit^ _VTST_ Path 1+ Path 2 |
| 230 | 1.75E-12 | 1.75E-12 | 2.33E-14 | 2.38E-14 |
| 232 | 1.75E-12 | 1.75E-12 | 2.40E-14 | 2.45E-14 |
| 240 | 1.79E-12 | 1.79E-12 | 2.71E-14 | 2.77E-14 |
| 250 | 1.82E-12 | 1.82E-12 | 3.12E-14 | 3.18E-14 |
| 260 | 1.86E-12 | 1.87E-12 | 3.56E-14 | 3.63E-14 |
| 270 | 1.91E-12 | 1.91E-12 | 4.04E-14 | 4.11E-14 |
| 280 | 1.96E-12 | 1.96E-12 | 4.55E-14 | 4.63E-14 |
| 290 | 2.02E-12 | 2.02E-12 | 5.11E-14 | 5.20E-14 |
| 298 | 2.06E-12 | 2.06E-12 | 5.57E-14 | 5.66E-14 |
| 298.15 | 2.06E-12 | 2.06E-12 | 5.58E-14 | 5.68E-14 |
| 300 | 2.07E-12 | 2.08E-12 | 5.70E-14 | 5.79E-14 |
| 310 | 2.13E-12 | 2.13E-12 | 6.32E-14 | 6.42E-14 |
| 320 | 2.20E-12 | 2.20E-12 | 6.99E-14 | 7.10E-14 |
| 330 | 2.26E-12 | 2.26E-12 | 7.70E-14 | 7.81E-14 |
| 340 | 2.33E-12 | 2.33E-12 | 8.45E-14 | 8.57E-14 |
| 350 | 2.41E-12 | 2.41E-12 | 9.24E-14 | 9.37E-14 |
| 360 | 2.48E-12 | 2.48E-12 | 1.01E-13 | 1.02E-13 |
| 374 | 2.59E-12 | 2.60E-12 | 1.13E-13 | 1.15E-13 |
| 380 | 2.64E-12 | 2.64E-12 | 1.19E-13 | 1.21E-13 |
| 390 | 2.73E-12 | 2.73E-12 | 1.28E-13 | 1.30E-13 |
| 400 | 2.81E-12 | 2.81E-12 | 1.38E-13 | 1.40E-13 |
| 410 | 2.90E-12 | 2.90E-12 | 1.49E-13 | 1.51E-13 |
| 424 | 3.03E-12 | 3.03E-12 | 1.65E-13 | 1.67E-13 |
| 430 | 3.09E-12 | 3.09E-12 | 1.72E-13 | 1.74E-13 |
| 440 | 3.19E-12 | 3.19E-12 | 1.84E-13 | 1.86E-13 |
| 450 | 3.29E-12 | 3.29E-12 | 1.97E-13 | 1.99E-13 |
| 460 | 3.39E-12 | 3.39E-12 | 2.10E-13 | 2.12E-13 |
| 470 | 3.49E-12 | 3.49E-12 | 2.24E-13 | 2.26E-13 |
| 480 | 3.60E-12 | 3.60E-12 | 2.38E-13 | 2.41E-13 |
| 490 | 3.71E-12 | 3.72E-12 | 2.53E-13 | 2.55E-13 |
| 500 | 3.83E-12 | 3.83E-12 | 2.68E-13 | 2.71E-13 |
| 510 | 3.94E-12 | 3.94E-12 | 2.85E-13 | 2.87E-13 |
| 520 | 4.06E-12 | 4.07E-12 | 3.01E-13 | 3.04E-13 |
| 530 | 4.19E-12 | 4.19E-12 | 3.18E-13 | 3.21E-13 |
| 540 | 4.31E-12 | 4.31E-12 | 3.36E-13 | 3.39E-13 |
| 550 | 4.44E-12 | 4.44E-12 | 3.54E-13 | 3.58E-13 |
| 560 | 4.57E-12 | 4.57E-12 | 3.73E-13 | 3.76E-13 |
| 570 | 4.70E-12 | 4.70E-12 | 3.92E-13 | 3.96E-13 |
| 580 | 4.83E-12 | 4.84E-12 | 4.13E-13 | 4.16E-13 |
| 590 | 4.98E-12 | 4.98E-12 | 4.33E-13 | 4.37E-13 |
| 600 | 5.12E-12 | 5.12E-12 | 4.54E-13 | 4.58E-13 |
| 610 | 5.26E-12 | 5.27E-12 | 4.77E-13 | 4.81E-13 |
| 620 | 5.41E-12 | 5.42E-12 | 5.00E-13 | 5.04E-13 |
| 630 | 5.56E-12 | 5.57E-12 | 5.23E-13 | 5.27E-13 |
| 637 | 5.67E-12 | 5.68E-12 | 5.40E-13 | 5.43E-13 |
| 640 | 5.72E-12 | 5.72E-12 | 5.47E-13 | 5.51E-13 |
| 650 | 5.87E-12 | 5.87E-12 | 5.72E-13 | 5.76E-13 |
| 660 | 6.03E-12 | 6.03E-12 | 5.97E-13 | 6.01E-13 |
| 670 | 6.20E-12 | 6.20E-12 | 6.23E-13 | 6.27E-13 |
| 680 | 6.36E-12 | 6.37E-12 | 6.50E-13 | 6.54E-13 |
| 690 | 6.53E-12 | 6.54E-12 | 6.77E-13 | 6.82E-13 |
| 700 | 6.70E-12 | 6.71E-12 | 7.06E-13 | 7.11E-13 |
| 800 | 8.59E-12 | 8.60E-12 | 1.03E-12 | 1.04E-12 |
| 900 | 1.08E-11 | 1.08E-11 | 1.44E-12 | 1.45E-12 |
| 1000 | 1.34E-11 | 1.34E-11 | 1.93E-12 | 1.94E-12 |
| 1100 | 1.63E-11 | 1.63E-11 | 2.52E-12 | 2.53E-12 |
| 1200 | 1.96E-11 | 1.96E-11 | 3.22E-12 | 3.23E-12 |
| 1300 | 2.33E-11 | 2.33E-11 | 4.03E-12 | 4.04E-12 |
| 1400 | 2.74E-11 | 2.74E-11 | 4.96E-12 | 4.97E-12 |
| 1500 | 3.19E-11 | 3.19E-11 | 6.01E-12 | 6.02E-12 |
| 1600 | 3.69E-11 | 3.69E-11 | 7.19E-12 | 7.21E-12 |
| 1700 | 4.23E-11 | 4.23E-11 | 8.51E-12 | 8.53E-12 |
| 1800 | 4.82E-11 | 4.82E-11 | 9.98E-12 | 1.00E-11 |
| 1900 | 5.45E-11 | 5.45E-11 | 1.16E-11 | 1.16E-11 |
| 2000 | 6.14E-11 | 6.14E-11 | 1.34E-11 | 1.34E-11 |
| 2100 | 6.87E-11 | 6.87E-11 | 1.53E-11 | 1.53E-11 |
| 2200 | 7.66E-11 | 7.66E-11 | 1.74E-11 | 1.74E-11 |
| 2300 | 8.51E-11 | 8.51E-11 | 1.97E-11 | 1.97E-11 |
| 2400 | 9.40E-11 | 9.40E-11 | 2.21E-11 | 2.21E-11 |
| 2500 | 1.04E-10 | 1.04E-10 | 2.47E-11 | 2.47E-11 |
| 2600 | 1.14E-10 | 1.14E-10 | 2.72E-11 | 2.73E-11 |
| 2700 | 1.25E-10 | 1.25E-10 | 3.00E-11 | 3.00E-11 |
| 2800 | 1.35E-10 | 1.35E-10 | 3.29E-11 | 3.30E-11 |
| 2900 | 1.47E-10 | 1.47E-10 | 3.60E-11 | 3.61E-11 |
| 3000 | 1.60E-10 | 1.60E-10 | 3.93E-11 | 3.94E-11 |

**Table S10**. High pressure limit rate constants calculated at the CCSD(T)/6-311++G(d,p)//B3LYP/6-311G(d,p) levels for path 1 + path 2.

|  | CCSD(T)/6-311++G(d,p)//B3LYP/6-311G(d,p) | | CCSD(T)/6-311++G(d,p)//B3LYP/6-311G(d,p) | |
| --- | --- | --- | --- | --- |
| T/K | k^Eckart^ _TST_ Path 3 | k^Shavit^ _TST_ Path 3 | k^Eckart^ _VTST_ Path 3 | k^Shavit^ _VTST_ Path 3 |
| 230 | 5.98E-32 | 3.69E-32 | 5.98E-32 | 3.69E-32 |
| 232 | 8.58E-32 | 5.38E-32 | 8.58E-32 | 5.38E-32 |
| 240 | 3.43E-31 | 2.28E-31 | 3.43E-31 | 2.28E-31 |
| 250 | 1.73E-30 | 1.22E-30 | 1.73E-30 | 1.22E-30 |
| 260 | 7.79E-30 | 5.77E-30 | 7.79E-30 | 5.77E-30 |
| 270 | 3.16E-29 | 2.44E-29 | 3.16E-29 | 2.44E-29 |
| 280 | 1.17E-28 | 9.31E-29 | 1.17E-28 | 9.31E-29 |
| 290 | 3.96E-28 | 3.25E-28 | 3.96E-28 | 3.25E-28 |
| 298 | 9.96E-28 | 8.33E-28 | 9.96E-28 | 8.33E-28 |
| 298.15 | 1.01E-27 | 8.48E-28 | 1.01E-27 | 8.48E-28 |
| 300 | 1.24E-27 | 1.05E-27 | 1.24E-27 | 1.05E-27 |
| 310 | 3.65E-27 | 3.13E-27 | 3.65E-27 | 3.13E-27 |
| 320 | 1.00E-26 | 8.77E-27 | 1.00E-26 | 8.77E-27 |
| 330 | 2.61E-26 | 2.31E-26 | 2.61E-26 | 2.31E-26 |
| 340 | 6.43E-26 | 5.77E-26 | 6.43E-26 | 5.77E-26 |
| 350 | 1.51E-25 | 1.37E-25 | 1.51E-25 | 1.37E-25 |
| 360 | 3.39E-25 | 3.11E-25 | 3.39E-25 | 3.11E-25 |
| 374 | 9.82E-25 | 9.11E-25 | 9.82E-25 | 9.11E-25 |
| 380 | 1.51E-24 | 1.41E-24 | 1.51E-24 | 1.41E-24 |
| 390 | 3.03E-24 | 2.84E-24 | 3.03E-24 | 2.84E-24 |
| 400 | 5.88E-24 | 5.54E-24 | 5.88E-24 | 5.54E-24 |
| 410 | 1.10E-23 | 1.05E-23 | 1.10E-23 | 1.05E-23 |
| 424 | 2.55E-23 | 2.43E-23 | 2.55E-23 | 2.43E-23 |
| 430 | 3.60E-23 | 3.44E-23 | 3.60E-23 | 3.44E-23 |
| 440 | 6.25E-23 | 6.00E-23 | 6.25E-23 | 6.00E-23 |
| 450 | 1.06E-22 | 1.02E-22 | 1.06E-22 | 1.02E-22 |
| 460 | 1.76E-22 | 1.70E-22 | 1.76E-22 | 1.70E-22 |
| 470 | 2.87E-22 | 2.78E-22 | 2.87E-22 | 2.78E-22 |
| 480 | 4.58E-22 | 4.45E-22 | 4.58E-22 | 4.45E-22 |
| 490 | 7.19E-22 | 7.00E-22 | 7.19E-22 | 7.00E-22 |
| 500 | 1.11E-21 | 1.08E-21 | 1.11E-21 | 1.08E-21 |
| 510 | 1.69E-21 | 1.65E-21 | 1.69E-21 | 1.65E-21 |
| 520 | 2.52E-21 | 2.47E-21 | 2.52E-21 | 2.47E-21 |
| 530 | 3.72E-21 | 3.65E-21 | 3.72E-21 | 3.65E-21 |
| 540 | 5.42E-21 | 5.32E-21 | 5.42E-21 | 5.32E-21 |
| 550 | 7.79E-21 | 7.65E-21 | 7.79E-21 | 7.65E-21 |
| 560 | 1.11E-20 | 1.09E-20 | 1.11E-20 | 1.09E-20 |
| 570 | 1.55E-20 | 1.53E-20 | 1.55E-20 | 1.53E-20 |
| 580 | 2.16E-20 | 2.13E-20 | 2.16E-20 | 2.13E-20 |
| 590 | 2.97E-20 | 2.93E-20 | 2.97E-20 | 2.93E-20 |
| 600 | 4.04E-20 | 3.99E-20 | 4.04E-20 | 3.99E-20 |
| 610 | 5.45E-20 | 5.38E-20 | 5.45E-20 | 5.38E-20 |
| 620 | 7.28E-20 | 7.20E-20 | 7.28E-20 | 7.20E-20 |
| 630 | 9.65E-20 | 9.55E-20 | 9.65E-20 | 9.55E-20 |
| 637 | 1.17E-19 | 1.16E-19 | 1.17E-19 | 1.16E-19 |
| 640 | 1.27E-19 | 1.26E-19 | 1.27E-19 | 1.26E-19 |
| 650 | 1.66E-19 | 1.64E-19 | 1.66E-19 | 1.64E-19 |
| 660 | 2.14E-19 | 2.13E-19 | 2.14E-19 | 2.13E-19 |
| 670 | 2.76E-19 | 2.74E-19 | 2.76E-19 | 2.74E-19 |
| 680 | 3.52E-19 | 3.50E-19 | 3.52E-19 | 3.50E-19 |
| 690 | 4.47E-19 | 4.44E-19 | 4.47E-19 | 4.44E-19 |
| 700 | 5.64E-19 | 5.60E-19 | 5.64E-19 | 5.60E-19 |
| 800 | 4.28E-18 | 4.27E-18 | 4.28E-18 | 4.27E-18 |
| 900 | 2.16E-17 | 2.15E-17 | 2.16E-17 | 2.15E-17 |
| 1000 | 8.09E-17 | 8.07E-17 | 8.09E-17 | 8.07E-17 |
| 1100 | 2.45E-16 | 2.44E-16 | 2.45E-16 | 2.44E-16 |
| 1200 | 6.28E-16 | 6.27E-16 | 6.28E-16 | 6.27E-16 |
| 1300 | 1.42E-15 | 1.42E-15 | 1.42E-15 | 1.42E-15 |
| 1400 | 2.89E-15 | 2.89E-15 | 2.89E-15 | 2.89E-15 |
| 1500 | 5.42E-15 | 5.42E-15 | 5.42E-15 | 5.42E-15 |
| 1600 | 9.51E-15 | 9.50E-15 | 9.51E-15 | 9.50E-15 |
| 1700 | 1.58E-14 | 1.57E-14 | 1.58E-14 | 1.57E-14 |
| 1800 | 2.49E-14 | 2.49E-14 | 2.49E-14 | 2.49E-14 |
| 1900 | 3.77E-14 | 3.77E-14 | 3.77E-14 | 3.77E-14 |
| 2000 | 5.53E-14 | 5.53E-14 | 5.53E-14 | 5.53E-14 |
| 2100 | 7.86E-14 | 7.85E-14 | 7.86E-14 | 7.85E-14 |
| 2200 | 1.09E-13 | 1.09E-13 | 1.09E-13 | 1.09E-13 |
| 2300 | 1.47E-13 | 1.47E-13 | 1.47E-13 | 1.47E-13 |
| 2400 | 1.95E-13 | 1.95E-13 | 1.95E-13 | 1.95E-13 |
| 2500 | 2.53E-13 | 2.53E-13 | 2.53E-13 | 2.53E-13 |
| 2600 | 3.24E-13 | 3.24E-13 | 3.24E-13 | 3.24E-13 |
| 2700 | 4.09E-13 | 4.08E-13 | 4.09E-13 | 4.08E-13 |
| 2800 | 5.08E-13 | 5.08E-13 | 5.08E-13 | 5.08E-13 |
| 2900 | 6.25E-13 | 6.25E-13 | 6.25E-13 | 6.25E-13 |
| 3000 | 7.60E-13 | 7.60E-13 | 7.60E-13 | 7.60E-13 |

**Table S11**. High pressure limit rate constants calculated at the CCSD(T)/6-311++G(d,p)//B3LYP/6-311G(d,p) levels for path 3.

| T/K | k^TSTa^ | k^CVTa^ | k^TSTb^ | k^CVTb^ | Sh-Corr^c^ | Vaghjinai 2001^d^ | Vaghjinai 1998^e^ | Harris^f^ | Hack |
| --- | --- | --- | --- | --- | --- | --- | --- | --- | --- |
| 230 | 1.64E-10 | 1.41E-10 | 8.51E-10 | 1.74E-10 | 43.09 |  |  |  |  |
| 232 | 1.59E-10 | 1.37E-10 | 8.06E-10 | 1.68E-10 | 42.72 | 4.32E-11 | 4.86E-11 |  |  |
| 240 | 1.41E-10 | 1.22E-10 | 6.55E-10 | 1.46E-10 | 41.32 | 4.23E-11 | 4.64E-11 |  |  |
| 250 | 1.25E-10 | 1.08E-10 | 5.16E-10 | 1.26E-10 | 39.69 | 4.12E-11 | 4.41E-11 |  |  |
| 260 | 1.12E-10 | 9.72E-11 | 4.15E-10 | 1.09E-10 | 38.19 | 4.02E-11 | 4.20E-11 |  |  |
| 270 | 1.02E-10 | 8.87E-11 | 3.40E-10 | 9.60E-11 | 36.80 | 3.92E-11 | 4.01E-11 |  |  |
| 280 | 9.44E-11 | 8.20E-11 | 2.83E-10 | 8.55E-11 | 35.51 | 3.84E-11 | 3.85E-11 |  |  |
| 290 | 8.80E-11 | 7.66E-11 | 2.40E-10 | 7.69E-11 | 34.31 | 3.77E-11 | 3.70E-11 |  |  |
| 298 | 8.39E-11 | 7.31E-11 | 2.11E-10 | 7.11E-11 | 33.40 | 3.71E-11 | 3.60E-11 | 6.49E-11 | 2.00E-11 |
| 298.15 | 8.38E-11 | 7.30E-11 | 2.11E-10 | 7.10E-11 | 33.39 | 3.71E-11 | 3.60E-11 | 6.49E-11 |  |
| 300 | 8.29E-11 | 7.22E-11 | 2.05E-10 | 6.98E-11 | 33.19 | 3.70E-11 | 3.57E-11 | 6.48E-11 |  |
| 310 | 7.88E-11 | 6.87E-11 | 1.78E-10 | 6.38E-11 | 32.14 | 3.64E-11 | 3.45E-11 | 6.40E-11 |  |
| 320 | 7.53E-11 | 6.57E-11 | 1.56E-10 | 5.88E-11 | 31.16 | 3.58E-11 | 3.35E-11 | 6.32E-11 |  |
| 330 | 7.25E-11 | 6.33E-11 | 1.38E-10 | 5.46E-11 | 30.24 | 3.52E-11 | 3.25E-11 | 6.25E-11 |  |
| 340 | 7.01E-11 | 6.13E-11 | 1.23E-10 | 5.09E-11 | 29.37 | 3.47E-11 | 3.16E-11 | 6.19E-11 |  |
| 350 | 6.81E-11 | 5.97E-11 | 1.11E-10 | 4.78E-11 | 28.56 | 3.43E-11 | 3.07E-11 | 6.13E-11 |  |
| 360 | 6.64E-11 | 5.82E-11 | 1.01E-10 | 4.51E-11 | 27.78 | 3.38E-11 | 3.00E-11 | 6.07E-11 |  |
| 374 | 6.46E-11 | 5.67E-11 | 8.89E-11 | 4.19E-11 | 26.78 | 3.33E-11 | 2.90E-11 | 6.00E-11 |  |
| 380 | 6.39E-11 | 5.62E-11 | 8.46E-11 | 4.07E-11 | 26.37 | 3.31E-11 |  | 5.97E-11 |  |
| 390 | 6.29E-11 | 5.54E-11 | 7.82E-11 | 3.88E-11 | 25.71 | 3.27E-11 |  | 5.92E-11 |  |
| 400 | 6.21E-11 | 5.48E-11 | 7.26E-11 | 3.72E-11 | 25.09 | 3.24E-11 |  | 5.88E-11 |  |
| 410 | 6.15E-11 | 5.43E-11 | 6.78E-11 | 3.58E-11 | 24.50 | 3.21E-11 |  | 5.84E-11 |  |
| 424 | 6.08E-11 | 5.37E-11 | 6.21E-11 | 3.41E-11 | 23.72 | 3.16E-11 |  | 5.78E-11 |  |
| 430 | 6.05E-11 | 5.36E-11 | 5.99E-11 | 3.34E-11 | 23.40 | 3.15E-11 |  |  |  |
| 440 | 6.02E-11 | 5.33E-11 | 5.67E-11 | 3.24E-11 | 22.89 | 3.12E-11 |  |  |  |
| 450 | 6.00E-11 | 5.32E-11 | 5.38E-11 | 3.15E-11 | 22.40 | 3.10E-11 |  |  |  |
| 460 | 5.98E-11 | 5.31E-11 | 5.12E-11 | 3.07E-11 | 21.93 | 3.07E-11 |  |  |  |
| 470 | 5.97E-11 | 5.30E-11 | 4.9E-11 | 3.00E-11 | 21.48 | 3.05E-11 |  |  |  |
| 480 | 5.97E-11 | 5.31E-11 | 4.69E-11 | 2.94E-11 | 21.05 | 3.03E-11 |  |  |  |
| 490 | 5.97E-11 | 5.32E-11 | 4.51E-11 | 2.88E-11 | 20.64 | 3.01E-11 |  |  |  |
| 500 | 5.98E-11 | 5.33E-11 | 4.34E-11 | 2.82E-11 | 20.25 | 2.99E-11 |  |  |  |
| 510 | 5.99E-11 | 5.35E-11 | 4.19E-11 | 2.78E-11 | 19.87 | 2.97E-11 |  |  |  |
| 520 | 6.01E-11 | 5.36E-11 | 4.06E-11 | 2.73E-11 | 19.50 | 2.95E-11 |  |  |  |
| 530 | 6.03E-11 | 5.38E-11 | 3.93E-11 | 2.70E-11 | 19.15 | 2.93E-11 |  |  |  |
| 540 | 6.06E-11 | 5.41E-11 | 3.82E-11 | 2.66E-11 | 18.81 | 2.92E-11 |  |  |  |
| 550 | 6.08E-11 | 5.44E-11 | 3.72E-11 | 2.63E-11 | 18.49 | 2.90E-11 |  |  |  |
| 560 | 6.12E-11 | 5.47E-11 | 3.63E-11 | 2.60E-11 | 18.18 | 2.89E-11 |  |  |  |
| 570 | 6.15E-11 | 5.50E-11 | 3.55E-11 | 2.58E-11 | 17.87 | 2.87E-11 |  |  |  |
| 580 | 6.19E-11 | 5.54E-11 | 3.47E-11 | 2.55E-11 | 17.58 | 2.86E-11 |  |  |  |
| 590 | 6.22E-11 | 5.58E-11 | 3.40E-11 | 2.53E-11 | 17.30 | 2.85E-11 |  |  |  |
| 600 | 6.27E-11 | 5.62E-11 | 3.34E-11 | 2.52E-11 | 17.03 | 2.83E-11 |  |  |  |
| 610 | 6.31E-11 | 5.66E-11 | 3.28E-11 | 2.50E-11 | 16.76 | 2.82E-11 |  |  |  |
| 620 | 6.36E-11 | 5.71E-11 | 3.23E-11 | 2.49E-11 | 16.51 | 2.81E-11 |  |  |  |
| 630 | 6.40E-11 | 5.75E-11 | 3.18E-11 | 2.47E-11 | 16.26 | 2.80E-11 |  |  |  |
| 637 | 6.44E-11 | 5.79E-11 | 3.15E-11 | 2.46E-11 | 16.09 | 2.79E-11 |  |  |  |
| 640 | 6.45E-11 | 5.80E-11 | 3.13E-11 | 2.46E-11 | 16.02 |  |  |  |  |
| 650 | 6.50E-11 | 5.85E-11 | 3.09E-11 | 2.45E-11 | 15.79 |  |  |  |  |
| 660 | 6.56E-11 | 5.89E-11 | 3.06E-11 | 2.44E-11 | 15.56 |  |  |  |  |
| 670 | 6.61E-11 | 5.95E-11 | 3.02E-11 | 2.43E-11 | 15.35 |  |  |  |  |
| 680 | 6.67E-11 | 6.00E-11 | 2.99E-11 | 2.42E-11 | 15.13 |  |  |  |  |
| 690 | 6.73E-11 | 6.05E-11 | 2.96E-11 | 2.42E-11 | 14.93 |  |  |  |  |
| 700 | 6.79E-11 | 6.11E-11 | 2.94E-11 | 2.41E-11 | 14.73 |  |  |  |  |
| 800 | 7.46E-11 | 6.73E-11 | 2.80E-11 | 2.44E-11 | 13.01 |  |  |  |  |
| 900 | 8.25E-11 | 7.43E-11 | 2.81E-11 | 2.54E-11 | 11.67 |  |  |  |  |
| 1000 | 9.13E-11 | 8.21E-11 | 2.90E-11 | 2.71E-11 | 10.60 |  |  |  |  |
| 1100 | 1.01E-10 | 9.04E-11 | 3.06E-11 | 2.91E-11 | 9.72 |  |  |  |  |
| 1200 | 1.11E-10 | 9.95E-11 | 3.27E-11 | 3.16E-11 | 9.00 |  |  |  |  |
| 1300 | 1.23E-10 | 1.09E-10 | 3.52E-11 | 3.43E-11 | 8.38 |  |  |  |  |
| 1400 | 1.34E-10 | 1.19E-10 | 3.81E-11 | 3.74E-11 | 7.85 |  |  |  |  |
| 1500 | 1.47E-10 | 1.29E-10 | 4.13E-11 | 4.08E-11 | 7.39 |  |  |  |  |
| 1600 | 1.61E-10 | 1.41E-10 | 4.49E-11 | 4.45E-11 | 6.99 |  |  |  |  |
| 1700 | 1.75E-10 | 1.53E-10 | 4.89E-11 | 4.85E-11 | 6.64 |  |  |  |  |
| 1800 | 1.89E-10 | 1.64E-10 | 5.32E-11 | 5.28E-11 | 6.33 |  |  |  |  |
| 1900 | 2.05E-10 | 1.77E-10 | 5.78E-11 | 5.74E-11 | 6.05 |  |  |  |  |
| 2000 | 2.21E-10 | 1.91E-10 | 6.27E-11 | 6.23E-11 | 5.79 |  |  |  |  |
| 2100 | 2.38E-10 | 2.05E-10 | 6.80E-11 | 6.75E-11 | 5.57 |  |  |  |  |
| 2200 | 2.55E-10 | 2.19E-10 | 7.36E-11 | 7.29E-11 | 5.36 |  |  |  |  |
| 2300 | 2.74E-10 | 2.34E-10 | 7.96E-11 | 7.87E-11 | 5.17 |  |  |  |  |
| 2400 | 2.93E-10 | 2.49E-10 | 8.59E-11 | 8.48E-11 | 4.99 |  |  |  |  |
| 2500 | 3.12E-10 | 2.65E-10 | 9.26E-11 | 9.12E-11 | 4.83 |  |  |  |  |
| 2600 | 3.33E-10 | 2.81E-10 | 9.96E-11 | 9.79E-11 | 4.69 |  |  |  |  |
| 2700 | 3.54E-10 | 2.98E-10 | 1.07E-10 | 1.05E-10 | 4.55 |  |  |  |  |
| 2800 | 3.75E-10 | 3.15E-10 | 1.15E-10 | 1.12E-10 | 4.42 |  |  |  |  |
| 2900 | 3.98E-10 | 3.32E-10 | 1.23E-10 | 1.20E-10 | 4.30 |  |  |  |  |
| 3000 | 4.21E-10 | 3.51E-10 | 1.31E-10 | 1.28E-10 | 4.19 |  |  |  |  |

All of the rate constants are in cm^3^molecule^-1^ s^-1^.

^a^calculated rate constant of P1 pathways at the M06-2X/maTZ with Shavit correction.

^b^calculated rate constant of P1 pathways at the CCSD(T)/aTZ//MP2/aTZ with Shavit correction.

^c^Shavit tunneling correction computed at the M06-2X/maTZ.

^d^obtained by the expression of k=(2.17±0.39) × 10^-11^­exp[(160±30)/T] cm^3^ molecule^-1^ s^-1^

^e^obtained by the expression of k=(1.25±0.19) × 10^-11^exp[(315±55)/T] cm^3^ molecule^-1^ s^-1^

^f^obtained by the expression of k=4.40 × 10^-11^­­­exp[(116±176)/T] cm^3^ molecule^-1^ s^-1^

**Table S12.** The high-pressure limit rate constants of P1 generation pathways (path 1 + path 2) and Shavitt tunneling correction.

|  | CCSD(T)/CBS//MP2/aTZ | CCSD(T)/aQZ//MP2/aTZ | CCSD(T)/aTZ// MP2/aTZ | MP2/aTZ | | M06-2X/aTZ |
| --- | --- | --- | --- | --- | --- | --- |
| T/K | k | k | k | k^Eckart^ | k^Shavit^ | k |
| 230 | 7.97E-10 | 7.84E-10 | 7.66E-10 | 5.35E-14 | 4.70E-14 | 1.14E-10 |
| 232 | 7.54E-10 | 7.41E-10 | 7.25E-10 | 5.44E-14 | 4.84E-14 | 1.10E-10 |
| 240 | 6.09E-10 | 5.99E-10 | 5.87E-10 | 5.83E-14 | 5.39E-14 | 9.34E-11 |
| 250 | 4.76E-10 | 4.69E-10 | 4.60E-10 | 6.33E-14 | 6.13E-14 | 7.77E-11 |
| 260 | 3.81E-10 | 3.75E-10 | 3.68E-10 | 6.88E-14 | 6.91E-14 | 6.58E-11 |
| 270 | 3.10E-10 | 3.06E-10 | 3.00E-10 | 7.45E-14 | 7.73E-14 | 5.65E-11 |
| 280 | 2.57E-10 | 2.54E-10 | 2.49E-10 | 8.06E-14 | 8.59E-14 | 4.92E-11 |
| 290 | 2.16E-10 | 2.13E-10 | 2.10E-10 | 8.70E-14 | 9.49E-14 | 4.33E-11 |
| 298 | 1.90E-10 | 1.88E-10 | 1.84E-10 | 9.23E-14 | 1.02E-13 | 3.94E-11 |
| 298.15 | 1.90E-10 | 1.87E-10 | 1.84E-10 | 9.25E-14 | 1.03E-13 | 3.94E-11 |
| 300 | 1.84E-10 | 1.82E-10 | 1.79E-10 | 9.37E-14 | 1.04E-13 | 3.85E-11 |
| 310 | 1.59E-10 | 1.57E-10 | 1.54E-10 | 1.01E-13 | 1.14E-13 | 3.46E-11 |
| 320 | 1.39E-10 | 1.37E-10 | 1.35E-10 | 1.08E-13 | 1.25E-13 | 3.14E-11 |
| 330 | 1.22E-10 | 1.21E-10 | 1.19E-10 | 1.16E-13 | 1.35E-13 | 2.87E-11 |
| 340 | 1.09E-10 | 1.07E-10 | 1.06E-10 | 1.24E-13 | 1.46E-13 | 2.64E-11 |
| 350 | 9.74E-11 | 9.64E-11 | 9.50E-11 | 1.33E-13 | 1.58E-13 | 2.45E-11 |
| 360 | 8.80E-11 | 8.71E-11 | 8.59E-11 | 1.42E-13 | 1.70E-13 | 2.28E-11 |
| 374 | 7.73E-11 | 7.65E-11 | 7.54E-11 | 1.55E-13 | 1.87E-13 | 2.08E-11 |
| 380 | 7.33E-11 | 7.26E-11 | 7.16E-11 | 1.61E-13 | 1.95E-13 | 2.01E-11 |
| 390 | 6.76E-11 | 6.69E-11 | 6.60E-11 | 1.72E-13 | 2.09E-13 | 1.90E-11 |
| 400 | 6.26E-11 | 6.20E-11 | 6.12E-11 | 1.82E-13 | 2.22E-13 | 1.80E-11 |
| 410 | 5.82E-11 | 5.77E-11 | 5.70E-11 | 1.93E-13 | 2.37E-13 | 1.72E-11 |
| 424 | 5.31E-11 | 5.26E-11 | 5.20E-11 | 2.10E-13 | 2.57E-13 | 1.62E-11 |
| 430 | 5.12E-11 | 5.07E-11 | 5.01E-11 | 2.17E-13 | 2.67E-13 | 1.58E-11 |
| 440 | 4.82E-11 | 4.78E-11 | 4.73E-11 | 2.30E-13 | 2.82E-13 | 1.52E-11 |
| 450 | 4.57E-11 | 4.53E-11 | 4.48E-11 | 2.43E-13 | 2.99E-13 | 1.47E-11 |
| 460 | 4.34E-11 | 4.30E-11 | 4.25E-11 | 2.56E-13 | 3.15E-13 | 1.42E-11 |
| 470 | 4.13E-11 | 4.10E-11 | 4.06E-11 | 2.70E-13 | 3.32E-13 | 1.38E-11 |
| 480 | 3.95E-11 | 3.92E-11 | 3.88E-11 | 2.84E-13 | 3.50E-13 | 1.34E-11 |
| 490 | 3.79E-11 | 3.76E-11 | 3.72E-11 | 2.99E-13 | 3.68E-13 | 1.31E-11 |
| 500 | 3.64E-11 | 3.61E-11 | 3.57E-11 | 3.15E-13 | 3.87E-13 | 1.28E-11 |
| 510 | 3.51E-11 | 3.48E-11 | 3.45E-11 | 3.31E-13 | 4.07E-13 | 1.25E-11 |
| 520 | 3.39E-11 | 3.36E-11 | 3.33E-11 | 3.47E-13 | 4.27E-13 | 1.23E-11 |
| 530 | 3.28E-11 | 3.25E-11 | 3.22E-11 | 3.64E-13 | 4.47E-13 | 1.20E-11 |
| 540 | 3.18E-11 | 3.16E-11 | 3.13E-11 | 3.82E-13 | 4.68E-13 | 1.18E-11 |
| 550 | 3.09E-11 | 3.07E-11 | 3.04E-11 | 4.00E-13 | 4.90E-13 | 1.16E-11 |
| 560 | 3.01E-11 | 2.99E-11 | 2.96E-11 | 4.19E-13 | 5.12E-13 | 1.15E-11 |
| 570 | 2.93E-11 | 2.91E-11 | 2.89E-11 | 4.38E-13 | 5.35E-13 | 1.13E-11 |
| 580 | 2.86E-11 | 2.84E-11 | 2.82E-11 | 4.58E-13 | 5.58E-13 | 1.12E-11 |
| 590 | 2.80E-11 | 2.78E-11 | 2.76E-11 | 4.78E-13 | 5.82E-13 | 1.11E-11 |
| 600 | 2.74E-11 | 2.73E-11 | 2.70E-11 | 4.99E-13 | 6.07E-13 | 1.10E-11 |
| 610 | 2.69E-11 | 2.67E-11 | 2.65E-11 | 5.21E-13 | 6.33E-13 | 1.09E-11 |
| 620 | 2.64E-11 | 2.63E-11 | 2.60E-11 | 5.43E-13 | 6.59E-13 | 1.08E-11 |
| 630 | 2.60E-11 | 2.58E-11 | 2.56E-11 | 5.66E-13 | 6.85E-13 | 1.07E-11 |
| 637 | 2.57E-11 | 2.55E-11 | 2.53E-11 | 5.83E-13 | 7.04E-13 | 1.07E-11 |
| 640 | 2.56E-11 | 2.54E-11 | 2.52E-11 | 5.90E-13 | 7.13E-13 | 1.07E-11 |
| 650 | 2.52E-11 | 2.51E-11 | 2.49E-11 | 6.14E-13 | 7.41E-13 | 1.06E-11 |
| 660 | 2.49E-11 | 2.47E-11 | 2.45E-11 | 6.39E-13 | 7.69E-13 | 1.05E-11 |
| 670 | 2.46E-11 | 2.44E-11 | 2.42E-11 | 6.64E-13 | 7.99E-13 | 1.05E-11 |
| 680 | 2.43E-11 | 2.42E-11 | 2.40E-11 | 6.91E-13 | 8.29E-13 | 1.05E-11 |
| 690 | 2.40E-11 | 2.39E-11 | 2.37E-11 | 7.18E-13 | 8.60E-13 | 1.04E-11 |
| 700 | 2.38E-11 | 2.37E-11 | 2.35E-11 | 7.45E-13 | 8.91E-13 | 1.04E-11 |
| 800 | 2.24E-11 | 2.23E-11 | 2.22E-11 | 1.06E-12 | 1.25E-12 | 1.05E-11 |
| 900 | 2.23E-11 | 2.22E-11 | 2.20E-11 | 1.46E-12 | 1.69E-12 | 1.09E-11 |
| 1000 | 2.28E-11 | 2.27E-11 | 2.26E-11 | 1.94E-12 | 2.22E-12 | 1.16E-11 |
| 1100 | 2.39E-11 | 2.38E-11 | 2.37E-11 | 2.52E-12 | 2.86E-12 | 1.24E-11 |
| 1200 | 2.54E-11 | 2.53E-11 | 2.52E-11 | 3.21E-12 | 3.60E-12 | 1.35E-11 |
| 1300 | 2.72E-11 | 2.71E-11 | 2.70E-11 | 4.01E-12 | 4.46E-12 | 1.46E-11 |
| 1400 | 2.93E-11 | 2.92E-11 | 2.91E-11 | 4.93E-12 | 5.45E-12 | 1.59E-11 |
| 1500 | 3.17E-11 | 3.16E-11 | 3.15E-11 | 5.99E-12 | 6.58E-12 | 1.74E-11 |
| 1600 | 3.43E-11 | 3.43E-11 | 3.41E-11 | 7.18E-12 | 7.84E-12 | 1.90E-11 |
| 1700 | 3.72E-11 | 3.72E-11 | 3.70E-11 | 8.52E-12 | 9.26E-12 | 2.07E-11 |
| 1800 | 4.04E-11 | 4.03E-11 | 4.02E-11 | 1.00E-11 | 1.08E-11 | 2.25E-11 |
| 1900 | 4.38E-11 | 4.37E-11 | 4.36E-11 | 1.17E-11 | 1.26E-11 | 2.44E-11 |
| 2000 | 4.74E-11 | 4.73E-11 | 4.72E-11 | 1.35E-11 | 1.45E-11 | 2.65E-11 |
| 2100 | 5.13E-11 | 5.12E-11 | 5.11E-11 | 1.55E-11 | 1.65E-11 | 2.87E-11 |
| 2200 | 5.54E-11 | 5.53E-11 | 5.52E-11 | 1.76E-11 | 1.88E-11 | 3.10E-11 |
| 2300 | 5.98E-11 | 5.97E-11 | 5.96E-11 | 2.00E-11 | 2.12E-11 | 3.35E-11 |
| 2400 | 6.45E-11 | 6.43E-11 | 6.42E-11 | 2.25E-11 | 2.39E-11 | 3.61E-11 |
| 2500 | 6.93E-11 | 6.92E-11 | 6.91E-11 | 2.52E-11 | 2.67E-11 | 3.88E-11 |
| 2600 | 7.45E-11 | 7.44E-11 | 7.42E-11 | 2.81E-11 | 2.97E-11 | 4.17E-11 |
| 2700 | 7.99E-11 | 7.98E-11 | 7.96E-11 | 3.13E-11 | 3.30E-11 | 4.47E-11 |
| 2800 | 8.56E-11 | 8.55E-11 | 8.53E-11 | 3.46E-11 | 3.64E-11 | 4.79E-11 |
| 2900 | 9.15E-11 | 9.14E-11 | 9.12E-11 | 3.82E-11 | 4.01E-11 | 5.12E-11 |
| 3000 | 9.77E-11 | 9.76E-11 | 9.74E-11 | 4.20E-11 | 4.40E-11 | 5.46E-11 |

**Table S13**. High pressure limit rate constants calculated by TST theory at different levels for path 1.

|  | CCSD(T)/CBS//MP2/aTZ | CCSD(T)/aQ Z//MP2/aTZ | CCSD(T)/aTZ//MP2/aTZ | MP2/aTZ | | M06-2X/maTZ | |
| --- | --- | --- | --- | --- | --- | --- | --- |
| T/K | k | k | k | k^Eckart^ | k^Shavit^ | k^Eckart^ | k^Shavit^ |
| 230 | 9.81E-11 | 9.22E-11 | 8.46E-11 | 4.11E-14 | 2.61E-14 | 4.77E-13 | 4.92E-11 |
| 232 | 9.37E-11 | 8.81E-11 | 8.09E-11 | 4.15E-14 | 2.69E-14 | 4.80E-13 | 4.90E-11 |
| 240 | 7.87E-11 | 7.41E-11 | 6.83E-11 | 4.31E-14 | 3.00E-14 | 4.94E-13 | 4.81E-11 |
| 250 | 6.44E-11 | 6.08E-11 | 5.62E-11 | 4.53E-14 | 3.42E-14 | 5.11E-13 | 4.72E-11 |
| 260 | 5.36E-11 | 5.07E-11 | 4.70E-11 | 4.76E-14 | 3.86E-14 | 5.29E-13 | 4.64E-11 |
| 270 | 4.54E-11 | 4.30E-11 | 4.00E-11 | 5.00E-14 | 4.32E-14 | 5.48E-13 | 4.57E-11 |
| 280 | 3.89E-11 | 3.70E-11 | 3.44E-11 | 5.25E-14 | 4.80E-14 | 5.68E-13 | 4.52E-11 |
| 290 | 3.38E-11 | 3.22E-11 | 3.00E-11 | 5.52E-14 | 5.30E-14 | 5.89E-13 | 4.47E-11 |
| 298 | 3.04E-11 | 2.90E-11 | 2.71E-11 | 5.74E-14 | 5.71E-14 | 6.06E-13 | 4.45E-11 |
| 298.15 | 3.04E-11 | 2.89E-11 | 2.71E-11 | 5.75E-14 | 5.72E-14 | 6.07E-13 | 4.44E-11 |
| 300 | 2.97E-11 | 2.83E-11 | 2.65E-11 | 5.80E-14 | 5.81E-14 | 6.11E-13 | 4.44E-11 |
| 310 | 2.63E-11 | 2.51E-11 | 2.36E-11 | 6.09E-14 | 6.35E-14 | 6.34E-13 | 4.41E-11 |
| 320 | 2.36E-11 | 2.25E-11 | 2.12E-11 | 6.40E-14 | 6.90E-14 | 6.57E-13 | 4.39E-11 |
| 330 | 2.13E-11 | 2.04E-11 | 1.92E-11 | 6.72E-14 | 7.47E-14 | 6.82E-13 | 4.38E-11 |
| 340 | 1.94E-11 | 1.86E-11 | 1.75E-11 | 7.05E-14 | 8.05E-14 | 7.07E-13 | 4.37E-11 |
| 350 | 1.77E-11 | 1.70E-11 | 1.61E-11 | 7.40E-14 | 8.65E-14 | 7.34E-13 | 4.36E-11 |
| 360 | 1.64E-11 | 1.57E-11 | 1.49E-11 | 7.77E-14 | 9.27E-14 | 7.61E-13 | 4.37E-11 |
| 374 | 1.47E-11 | 1.42E-11 | 1.35E-11 | 8.30E-14 | 1.02E-13 | 8.01E-13 | 4.37E-11 |
| 380 | 1.41E-11 | 1.36E-11 | 1.29E-11 | 8.54E-14 | 1.06E-13 | 8.18E-13 | 4.38E-11 |
| 390 | 1.32E-11 | 1.28E-11 | 1.21E-11 | 8.94E-14 | 1.12E-13 | 8.48E-13 | 4.39E-11 |
| 400 | 1.25E-11 | 1.20E-11 | 1.14E-11 | 9.37E-14 | 1.19E-13 | 8.79E-13 | 4.41E-11 |
| 410 | 1.18E-11 | 1.14E-11 | 1.08E-11 | 9.81E-14 | 1.26E-13 | 9.11E-13 | 4.43E-11 |
| 424 | 1.10E-11 | 1.06E-11 | 1.01E-11 | 1.04E-13 | 1.36E-13 | 9.58E-13 | 4.46E-11 |
| 430 | 1.06E-11 | 1.03E-11 | 9.84E-12 | 1.07E-13 | 1.41E-13 | 9.78E-13 | 4.47E-11 |
| 440 | 1.02E-11 | 9.85E-12 | 9.42E-12 | 1.12E-13 | 1.48E-13 | 1.01E-12 | 4.50E-11 |
| 450 | 9.75E-12 | 9.45E-12 | 9.04E-12 | 1.17E-13 | 1.56E-13 | 1.05E-12 | 4.53E-11 |
| 460 | 9.38E-12 | 9.09E-12 | 8.70E-12 | 1.23E-13 | 1.64E-13 | 1.09E-12 | 4.56E-11 |
| 470 | 9.04E-12 | 8.76E-12 | 8.40E-12 | 1.28E-13 | 1.72E-13 | 1.12E-12 | 4.59E-11 |
| 480 | 8.73E-12 | 8.47E-12 | 8.13E-12 | 1.34E-13 | 1.80E-13 | 1.16E-12 | 4.63E-11 |
| 490 | 8.46E-12 | 8.21E-12 | 7.89E-12 | 1.39E-13 | 1.89E-13 | 1.20E-12 | 4.67E-11 |
| 500 | 8.21E-12 | 7.98E-12 | 7.67E-12 | 1.45E-13 | 1.98E-13 | 1.24E-12 | 4.70E-11 |
| 510 | 7.99E-12 | 7.77E-12 | 7.47E-12 | 1.51E-13 | 2.07E-13 | 1.29E-12 | 4.74E-11 |
| 520 | 7.79E-12 | 7.57E-12 | 7.29E-12 | 1.58E-13 | 2.16E-13 | 1.33E-12 | 4.79E-11 |
| 530 | 7.60E-12 | 7.40E-12 | 7.13E-12 | 1.64E-13 | 2.25E-13 | 1.37E-12 | 4.83E-11 |
| 540 | 7.44E-12 | 7.24E-12 | 6.98E-12 | 1.71E-13 | 2.34E-13 | 1.42E-12 | 4.87E-11 |
| 550 | 7.29E-12 | 7.10E-12 | 6.85E-12 | 1.78E-13 | 2.44E-13 | 1.46E-12 | 4.92E-11 |
| 560 | 7.15E-12 | 6.97E-12 | 6.73E-12 | 1.85E-13 | 2.54E-13 | 1.51E-12 | 4.97E-11 |
| 570 | 7.03E-12 | 6.85E-12 | 6.62E-12 | 1.92E-13 | 2.64E-13 | 1.56E-12 | 5.02E-11 |
| 580 | 6.91E-12 | 6.74E-12 | 6.52E-12 | 2.00E-13 | 2.75E-13 | 1.61E-12 | 5.07E-11 |
| 590 | 6.81E-12 | 6.65E-12 | 6.43E-12 | 2.08E-13 | 2.86E-13 | 1.66E-12 | 5.12E-11 |
| 600 | 6.72E-12 | 6.56E-12 | 6.35E-12 | 2.16E-13 | 2.96E-13 | 1.71E-12 | 5.17E-11 |
| 610 | 6.64E-12 | 6.48E-12 | 6.27E-12 | 2.24E-13 | 3.08E-13 | 1.77E-12 | 5.22E-11 |
| 620 | 6.56E-12 | 6.41E-12 | 6.21E-12 | 2.32E-13 | 3.19E-13 | 1.82E-12 | 5.28E-11 |
| 630 | 6.49E-12 | 6.35E-12 | 6.15E-12 | 2.41E-13 | 3.31E-13 | 1.87E-12 | 5.33E-11 |
| 637 | 6.45E-12 | 6.30E-12 | 6.11E-12 | 2.47E-13 | 3.39E-13 | 1.91E-12 | 5.37E-11 |
| 640 | 6.43E-12 | 6.29E-12 | 6.10E-12 | 2.50E-13 | 3.42E-13 | 1.93E-12 | 5.39E-11 |
| 650 | 6.38E-12 | 6.24E-12 | 6.05E-12 | 2.59E-13 | 3.55E-13 | 1.99E-12 | 5.45E-11 |
| 660 | 6.33E-12 | 6.19E-12 | 6.01E-12 | 2.68E-13 | 3.67E-13 | 2.05E-12 | 5.50E-11 |
| 670 | 6.29E-12 | 6.15E-12 | 5.97E-12 | 2.78E-13 | 3.80E-13 | 2.11E-12 | 5.56E-11 |
| 680 | 6.25E-12 | 6.12E-12 | 5.94E-12 | 2.88E-13 | 3.93E-13 | 2.17E-12 | 5.62E-11 |
| 690 | 6.21E-12 | 6.09E-12 | 5.91E-12 | 2.98E-13 | 4.06E-13 | 2.23E-12 | 5.68E-11 |
| 700 | 6.19E-12 | 6.06E-12 | 5.89E-12 | 3.08E-13 | 4.19E-13 | 2.30E-12 | 5.75E-11 |
| 800 | 6.10E-12 | 5.99E-12 | 5.84E-12 | 4.27E-13 | 5.70E-13 | 3.01E-12 | 6.41E-11 |
| 900 | 6.27E-12 | 6.17E-12 | 6.04E-12 | 5.75E-13 | 7.53E-13 | 3.85E-12 | 7.15E-11 |
| 1000 | 6.62E-12 | 6.52E-12 | 6.40E-12 | 7.55E-13 | 9.71E-13 | 4.85E-12 | 7.97E-11 |
| 1100 | 7.10E-12 | 7.01E-12 | 6.88E-12 | 9.72E-13 | 1.23E-12 | 6.00E-12 | 8.85E-11 |
| 1200 | 7.69E-12 | 7.60E-12 | 7.48E-12 | 1.23E-12 | 1.53E-12 | 7.32E-12 | 9.79E-11 |
| 1300 | 8.39E-12 | 8.30E-12 | 8.17E-12 | 1.53E-12 | 1.88E-12 | 8.82E-12 | 1.08E-10 |
| 1400 | 9.18E-12 | 9.09E-12 | 8.96E-12 | 1.87E-12 | 2.27E-12 | 1.05E-11 | 1.19E-10 |
| 1500 | 1.01E-11 | 9.97E-12 | 9.83E-12 | 2.27E-12 | 2.72E-12 | 1.24E-11 | 1.30E-10 |
| 1600 | 1.10E-11 | 1.09E-11 | 1.08E-11 | 2.71E-12 | 3.22E-12 | 1.45E-11 | 1.42E-10 |
| 1700 | 1.21E-11 | 1.20E-11 | 1.19E-11 | 3.22E-12 | 3.78E-12 | 1.68E-11 | 1.54E-10 |
| 1800 | 1.32E-11 | 1.31E-11 | 1.30E-11 | 3.78E-12 | 4.40E-12 | 1.93E-11 | 1.67E-10 |
| 1900 | 1.45E-11 | 1.44E-11 | 1.42E-11 | 4.40E-12 | 5.09E-12 | 2.21E-11 | 1.80E-10 |
| 2000 | 1.58E-11 | 1.57E-11 | 1.55E-11 | 5.08E-12 | 5.84E-12 | 2.51E-11 | 1.95E-10 |
| 2100 | 1.72E-11 | 1.71E-11 | 1.69E-11 | 5.83E-12 | 6.66E-12 | 2.84E-11 | 2.09E-10 |
| 2200 | 1.87E-11 | 1.86E-11 | 1.84E-11 | 6.64E-12 | 7.54E-12 | 3.19E-11 | 2.24E-10 |
| 2300 | 2.03E-11 | 2.02E-11 | 2.00E-11 | 7.53E-12 | 8.51E-12 | 3.57E-11 | 2.40E-10 |
| 2400 | 2.20E-11 | 2.19E-11 | 2.17E-11 | 8.49E-12 | 9.54E-12 | 3.97E-11 | 2.57E-10 |
| 2500 | 2.38E-11 | 2.37E-11 | 2.35E-11 | 9.52E-12 | 1.07E-11 | 4.40E-11 | 2.73E-10 |
| 2600 | 2.57E-11 | 2.56E-11 | 2.54E-11 | 1.06E-11 | 1.19E-11 | 4.86E-11 | 2.91E-10 |
| 2700 | 2.77E-11 | 2.76E-11 | 2.74E-11 | 1.18E-11 | 1.31E-11 | 5.35E-11 | 3.09E-10 |
| 2800 | 2.98E-11 | 2.97E-11 | 2.94E-11 | 1.31E-11 | 1.45E-11 | 5.87E-11 | 3.27E-10 |
| 2900 | 3.20E-11 | 3.18E-11 | 3.16E-11 | 1.44E-11 | 1.59E-11 | 6.42E-11 | 3.47E-10 |
| 3000 | 3.43E-11 | 3.41E-11 | 3.39E-11 | 1.59E-11 | 1.75E-11 | 7.00E-11 | 3.66E-10 |

**Table S14**. High pressure limit rate constants calculated by TST theory at different levels for path 2.

|  | CCSD(T)/CBS//MP2/aTZ | CCSD(T)/aQZ//MP2/aTZ | CCSD(T)/aTZ//MP2/aTZ | MP2/aTZ | | M06-2X/maTZ | |
| --- | --- | --- | --- | --- | --- | --- | --- |
| T/K | k | k | k | k^Eckart^ | k^Shavit^ | k^Eckart^ | k^Shavit^ |
| 230 | 8.95E-10 | 8.76E-10 | 8.51E-10 | 9.46E-14 | 7.31E-14 | 1.15E-10 | 1.64E-10 |
| 232 | 8.47E-10 | 8.29E-10 | 8.06E-10 | 9.59E-14 | 7.52E-14 | 1.10E-10 | 1.59E-10 |
| 240 | 6.88E-10 | 6.73E-10 | 6.55E-10 | 1.01E-13 | 8.40E-14 | 9.39E-11 | 1.41E-10 |
| 250 | 5.41E-10 | 5.3E-10 | 5.16E-10 | 1.09E-13 | 9.55E-14 | 7.82E-11 | 1.25E-10 |
| 260 | 4.34E-10 | 4.26E-10 | 4.15E-10 | 1.16E-13 | 1.08E-13 | 6.63E-11 | 1.12E-10 |
| 270 | 3.56E-10 | 3.49E-10 | 3.4E-10 | 1.24E-13 | 1.20E-13 | 5.70E-11 | 1.02E-10 |
| 280 | 2.96E-10 | 2.90E-10 | 2.83E-10 | 1.33E-13 | 1.34E-13 | 4.97E-11 | 9.44E-11 |
| 290 | 2.50E-10 | 2.45E-10 | 2.4E-10 | 1.42E-13 | 1.48E-13 | 4.39E-11 | 8.80E-11 |
| 298 | 2.20E-10 | 2.17E-10 | 2.11E-10 | 1.50E-13 | 1.60E-13 | 4.00E-11 | 8.39E-11 |
| 298.15 | 2.20E-10 | 2.16E-10 | 2.11E-10 | 1.50E-13 | 1.60E-13 | 4.00E-11 | 8.38E-11 |
| 300 | 2.14E-10 | 2.1E-10 | 2.05E-10 | 1.52E-13 | 1.63E-13 | 3.92E-11 | 8.29E-11 |
| 310 | 1.85E-10 | 1.82E-10 | 1.78E-10 | 1.62E-13 | 1.78E-13 | 3.53E-11 | 7.88E-11 |
| 320 | 1.62E-10 | 1.60E-10 | 1.56E-10 | 1.72E-13 | 1.94E-13 | 3.21E-11 | 7.53E-11 |
| 330 | 1.43E-10 | 1.41E-10 | 1.38E-10 | 1.83E-13 | 2.10E-13 | 2.94E-11 | 7.25E-11 |
| 340 | 1.28E-10 | 1.26E-10 | 1.23E-10 | 1.95E-13 | 2.27E-13 | 2.71E-11 | 7.01E-11 |
| 350 | 1.15E-10 | 1.13E-10 | 1.11E-10 | 2.07E-13 | 2.44E-13 | 2.52E-11 | 6.81E-11 |
| 360 | 1.04E-10 | 1.03E-10 | 1.01E-10 | 2.20E-13 | 2.63E-13 | 2.35E-11 | 6.64E-11 |
| 374 | 9.20E-11 | 9.07E-11 | 8.89E-11 | 2.38E-13 | 2.89E-13 | 2.16E-11 | 6.46E-11 |
| 380 | 8.75E-11 | 8.62E-11 | 8.46E-11 | 2.47E-13 | 3.01E-13 | 2.09E-11 | 6.39E-11 |
| 390 | 8.08E-11 | 7.91E-11 | 7.82E-11 | 2.61E-13 | 3.21E-13 | 1.99E-11 | 6.29E-11 |
| 400 | 7.50E-11 | 7.40E-11 | 7.26E-11 | 2.76E-13 | 3.42E-13 | 1.89E-11 | 6.21E-11 |
| 410 | 7.00E-11 | 6.91E-11 | 6.78E-11 | 2.92E-13 | 3.63E-13 | 1.81E-11 | 6.15E-11 |
| 424 | 6.41E-11 | 6.32E-11 | 6.21E-11 | 3.14E-13 | 3.94E-13 | 1.71E-11 | 6.08E-11 |
| 430 | 6.18E-11 | 6.10E-11 | 5.99E-11 | 3.24E-13 | 4.07E-13 | 1.68E-11 | 6.05E-11 |
| 440 | 5.84E-11 | 5.77E-11 | 5.67E-11 | 3.42E-13 | 4.31E-13 | 1.62E-11 | 6.02E-11 |
| 450 | 5.54E-11 | 5.47E-11 | 5.38E-11 | 3.60E-13 | 4.55E-13 | 1.57E-11 | 6.00E-11 |
| 460 | 5.28E-11 | 5.21E-11 | 5.12E-11 | 3.78E-13 | 4.79E-13 | 1.53E-11 | 5.98E-11 |
| 470 | 5.04E-11 | 4.98E-11 | 4.9E-11 | 3.98E-13 | 5.05E-13 | 1.49E-11 | 5.97E-11 |
| 480 | 4.82E-11 | 4.77E-11 | 4.69E-11 | 4.18E-13 | 5.31E-13 | 1.46E-11 | 5.97E-11 |
| 490 | 4.63E-11 | 4.58E-11 | 4.51E-11 | 4.39E-13 | 5.57E-13 | 1.43E-11 | 5.97E-11 |
| 500 | 4.46E-11 | 4.41E-11 | 4.34E-11 | 4.60E-13 | 5.85E-13 | 1.40E-11 | 5.98E-11 |
| 510 | 4.31E-11 | 4.26E-11 | 4.19E-11 | 4.82E-13 | 6.13E-13 | 1.38E-11 | 5.99E-11 |
| 520 | 4.16E-11 | 4.12E-11 | 4.06E-11 | 5.05E-13 | 6.42E-13 | 1.36E-11 | 6.01E-11 |
| 530 | 4.04E-11 | 3.99E-11 | 3.93E-11 | 5.28E-13 | 6.72E-13 | 1.34E-11 | 6.03E-11 |
| 540 | 3.92E-11 | 3.88E-11 | 3.82E-11 | 5.53E-13 | 7.03E-13 | 1.32E-11 | 6.06E-11 |
| 550 | 3.82E-11 | 3.78E-11 | 3.72E-11 | 5.78E-13 | 7.34E-13 | 1.31E-11 | 6.08E-11 |
| 560 | 3.72E-11 | 3.68E-11 | 3.63E-11 | 6.04E-13 | 7.66E-13 | 1.3E-11 | 6.12E-11 |
| 570 | 3.63E-11 | 3.60E-11 | 3.55E-11 | 6.30E-13 | 7.99E-13 | 1.29E-11 | 6.15E-11 |
| 580 | 3.55E-11 | 3.52E-11 | 3.47E-11 | 6.58E-13 | 8.33E-13 | 1.28E-11 | 6.19E-11 |
| 590 | 3.48E-11 | 3.45E-11 | 3.4E-11 | 6.86E-13 | 8.68E-13 | 1.27E-11 | 6.22E-11 |
| 600 | 3.41E-11 | 3.38E-11 | 3.34E-11 | 7.15E-13 | 9.04E-13 | 1.27E-11 | 6.27E-11 |
| 610 | 3.35E-11 | 3.32E-11 | 3.28E-11 | 7.45E-13 | 9.40E-13 | 1.26E-11 | 6.31E-11 |
| 620 | 3.30E-11 | 3.27E-11 | 3.23E-11 | 7.76E-13 | 9.77E-13 | 1.26E-11 | 6.36E-11 |
| 630 | 3.25E-11 | 3.22E-11 | 3.18E-11 | 8.07E-13 | 1.02E-12 | 1.26E-11 | 6.40E-11 |
| 637 | 3.22E-11 | 3.19E-11 | 3.15E-11 | 8.30E-13 | 1.04E-12 | 1.26E-11 | 6.44E-11 |
| 640 | 3.20E-11 | 3.17E-11 | 3.13E-11 | 8.40E-13 | 1.05E-12 | 1.26E-11 | 6.45E-11 |
| 650 | 3.16E-11 | 3.13E-11 | 3.09E-11 | 8.73E-13 | 1.10E-12 | 1.26E-11 | 6.50E-11 |
| 660 | 3.12E-11 | 3.09E-11 | 3.06E-11 | 9.07E-13 | 1.14E-12 | 1.26E-11 | 6.56E-11 |
| 670 | 3.09E-11 | 3.06E-11 | 3.02E-11 | 9.43E-13 | 1.18E-12 | 1.26E-11 | 6.61E-11 |
| 680 | 3.05E-11 | 3.03E-11 | 2.99E-11 | 9.79E-13 | 1.22E-12 | 1.26E-11 | 6.67E-11 |
| 690 | 3.02E-11 | 3.00E-11 | 2.96E-11 | 1.02E-12 | 1.27E-12 | 1.27E-11 | 6.73E-11 |
| 700 | 3.00E-11 | 2.97E-11 | 2.94E-11 | 1.05E-12 | 1.31E-12 | 1.27E-11 | 6.79E-11 |
| 800 | 2.85E-11 | 2.83E-11 | 2.8E-11 | 1.49E-12 | 1.82E-12 | 1.35E-11 | 7.46E-11 |
| 900 | 2.85E-11 | 2.83E-11 | 2.81E-11 | 2.03E-12 | 2.44E-12 | 1.48E-11 | 8.25E-11 |
| 1000 | 2.94E-11 | 2.93E-11 | 2.9E-11 | 2.70E-12 | 3.19E-12 | 1.64E-11 | 9.13E-11 |
| 1100 | 3.10E-11 | 3.08E-11 | 3.06E-11 | 3.49E-12 | 4.09E-12 | 1.84E-11 | 1.01E-10 |
| 1200 | 3.31E-11 | 3.29E-11 | 3.27E-11 | 4.44E-12 | 5.13E-12 | 2.08E-11 | 1.11E-10 |
| 1300 | 3.56E-11 | 3.54E-11 | 3.52E-11 | 5.54E-12 | 6.34E-12 | 2.35E-11 | 1.23E-10 |
| 1400 | 3.85E-11 | 3.83E-11 | 3.81E-11 | 6.81E-12 | 7.73E-12 | 2.65E-11 | 1.34E-10 |
| 1500 | 4.18E-11 | 4.16E-11 | 4.13E-11 | 8.26E-12 | 9.30E-12 | 2.98E-11 | 1.47E-10 |
| 1600 | 4.54E-11 | 4.52E-11 | 4.49E-11 | 9.89E-12 | 1.11E-11 | 3.35E-11 | 1.61E-10 |
| 1700 | 4.93E-11 | 4.91E-11 | 4.89E-11 | 1.17E-11 | 1.30E-11 | 3.75E-11 | 1.75E-10 |
| 1800 | 5.36E-11 | 5.34E-11 | 5.32E-11 | 1.38E-11 | 1.52E-11 | 4.18E-11 | 1.89E-10 |
| 1900 | 5.83E-11 | 5.81E-11 | 5.78E-11 | 1.60E-11 | 1.76E-11 | 4.65E-11 | 2.05E-10 |
| 2000 | 6.32E-11 | 6.30E-11 | 6.27E-11 | 1.85E-11 | 2.03E-11 | 5.16E-11 | 2.21E-10 |
| 2100 | 6.85E-11 | 6.83E-11 | 6.8E-11 | 2.13E-11 | 2.32E-11 | 5.71E-11 | 2.38E-10 |
| 2200 | 7.42E-11 | 7.39E-11 | 7.36E-11 | 2.43E-11 | 2.63E-11 | 6.29E-11 | 2.55E-10 |
| 2300 | 8.01E-11 | 7.99E-11 | 7.96E-11 | 2.75E-11 | 2.97E-11 | 6.92E-11 | 2.74E-10 |
| 2400 | 8.65E-11 | 8.63E-11 | 8.59E-11 | 3.10E-11 | 3.34E-11 | 7.58E-11 | 2.93E-10 |
| 2500 | 9.32E-11 | 9.29E-11 | 9.26E-11 | 3.47E-11 | 3.74E-11 | 8.29E-11 | 3.12E-10 |
| 2600 | 1.00E-10 | 1.00E-10 | 9.96E-11 | 3.88E-11 | 4.16E-11 | 9.03E-11 | 3.33E-10 |
| 2700 | 1.08E-10 | 1.07E-10 | 1.07E-10 | 4.31E-11 | 4.61E-11 | 9.82E-11 | 3.54E-10 |
| 2800 | 1.15E-10 | 1.15E-10 | 1.15E-10 | 4.77E-11 | 5.09E-11 | 1.07E-10 | 3.75E-10 |
| 2900 | 1.24E-10 | 1.23E-10 | 1.23E-10 | 5.26E-11 | 5.61E-11 | 1.15E-10 | 3.98E-10 |
| 3000 | 1.32E-10 | 1.32E-10 | 1.31E-10 | 5.79E-11 | 6.15E-11 | 1.25E-10 | 4.21E-10 |

**Table S15**. High pressure limit rate constants calculated by TST theory at different levels for P1 adducts via path 1 + path 2.

| T/K | k^TSTa^ | k^CVTa^ | k^TSTb^ | k^CVTb^ |
| --- | --- | --- | --- | --- |
| 230 | 2.29E-34 | 2.58E-34 | 2.70E-52 | 1.09E-52 |
| 232 | 3.47E-34 | 3.90E-34 | 5.82E-52 | 2.38E-52 |
| 240 | 1.71E-33 | 1.92E-33 | 1.11E-50 | 4.68E-51 |
| 250 | 1.09E-32 | 1.22E-32 | 3.40E-49 | 1.48E-49 |
| 260 | 6.07E-32 | 6.73E-32 | 7.99E-48 | 3.60E-48 |
| 270 | 2.97E-31 | 3.28E-31 | 1.49E-46 | 6.90E-47 |
| 280 | 1.30E-30 | 1.43E-30 | 2.25E-45 | 1.07E-45 |
| 290 | 5.16E-30 | 5.66E-30 | 2.83E-44 | 1.38E-44 |
| 298 | 1.45E-29 | 1.59E-29 | 1.90E-43 | 9.44E-44 |
| 298.15 | 1.48E-29 | 1.62E-29 | 1.96E-43 | 9.77E-44 |
| 300 | 1.87E-29 | 2.04E-29 | 3.00E-43 | 1.50E-43 |
| 310 | 6.24E-29 | 6.81E-29 | 2.74E-42 | 1.40E-42 |
| 320 | 1.94E-28 | 2.11E-28 | 2.18E-41 | 1.14E-41 |
| 330 | 5.63E-28 | 6.11E-28 | 1.53E-40 | 8.14E-41 |
| 340 | 1.54E-27 | 1.67E-27 | 9.61E-40 | 5.20E-40 |
| 350 | 3.98E-27 | 4.3E-27 | 5.43E-39 | 2.99E-39 |
| 360 | 9.77E-27 | 1.05E-26 | 2.79E-38 | 1.56E-38 |
| 374 | 3.18E-26 | 3.42E-26 | 2.39E-37 | 1.36E-37 |
| 380 | 5.14E-26 | 5.52E-26 | 5.73E-37 | 3.29E-37 |
| 390 | 1.11E-25 | 1.19E-25 | 2.31E-36 | 1.35E-36 |
| 400 | 2.3E-25 | 2.47E-25 | 8.72E-36 | 5.14E-36 |
| 410 | 4.63E-25 | 4.94E-25 | 3.09E-35 | 1.84E-35 |
| 424 | 1.17E-24 | 1.24E-24 | 1.64E-34 | 9.94E-35 |
| 430 | 1.70E-24 | 1.81E-24 | 3.25E-34 | 1.98E-34 |
| 440 | 3.13E-24 | 3.32E-24 | 9.76E-34 | 6.01E-34 |
| 450 | 5.6E-24 | 5.94E-24 | 2.79E-33 | 1.73E-33 |
| 460 | 9.78E-24 | 1.04E-23 | 7.64E-33 | 4.79E-33 |
| 470 | 1.67E-23 | 1.77E-23 | 2.00E-32 | 1.27E-32 |
| 480 | 2.8E-23 | 2.96E-23 | 5.06E-32 | 3.23E-32 |
| 490 | 4.59E-23 | 4.85E-23 | 1.23E-31 | 7.91E-32 |
| 500 | 7.39E-23 | 7.80E-23 | 2.89E-31 | 1.87E-31 |
| 510 | 1.17E-22 | 1.23E-22 | 6.59E-31 | 4.29E-31 |
| 520 | 1.82E-22 | 1.91E-22 | 1.45E-30 | 9.54E-31 |
| 530 | 2.78E-22 | 2.93E-22 | 3.12E-30 | 2.06E-30 |
| 540 | 4.2E-22 | 4.41E-22 | 6.50E-30 | 4.32E-30 |
| 550 | 6.24E-22 | 6.56E-22 | 1.32E-29 | 8.84E-30 |
| 560 | 9.16E-22 | 9.62E-22 | 2.62E-29 | 1.77E-29 |
| 570 | 1.33E-21 | 1.39E-21 | 5.09E-29 | 3.44E-29 |
| 580 | 1.90E-21 | 1.99E-21 | 9.65E-29 | 6.56E-29 |
| 590 | 2.69E-21 | 2.82E-21 | 1.79E-28 | 1.23E-28 |
| 600 | 3.77E-21 | 3.95E-21 | 3.27E-28 | 2.24E-28 |
| 610 | 5.23E-21 | 5.46E-21 | 5.84E-28 | 4.03E-28 |
| 620 | 7.18E-21 | 7.50E-21 | 1.02E-27 | 7.10E-28 |
| 630 | 9.76E-21 | 1.02E-20 | 1.77E-27 | 1.23E-27 |
| 637 | 1.20E-20 | 1.26E-20 | 2.57E-27 | 1.79E-27 |
| 640 | 1.31E-20 | 1.37E-20 | 3.00E-27 | 2.10E-27 |
| 650 | 1.76E-20 | 1.83E-20 | 5.02E-27 | 3.52E-27 |
| 660 | 2.33E-20 | 2.43E-20 | 8.27E-27 | 5.82E-27 |
| 670 | 3.06E-20 | 3.19E-20 | 1.34E-26 | 9.49E-27 |
| 680 | 4.00E-20 | 4.16E-20 | 2.15E-26 | 1.53E-26 |
| 690 | 5.18E-20 | 5.39E-20 | 3.40E-26 | 2.42E-26 |
| 700 | 6.67E-20 | 6.93E-20 | 5.31E-26 | 3.80E-26 |
| 800 | 6.03E-19 | 6.24E-19 | 2.56E-24 | 1.88E-24 |
| 900 | 3.47E-18 | 3.58E-18 | 5.47E-23 | 4.10E-23 |
| 1000 | 1.45E-17 | 1.49E-17 | 6.57E-22 | 5.00E-22 |
| 1100 | 4.79E-17 | 4.91E-17 | 5.17E-21 | 3.98E-21 |
| 1200 | 1.32E-16 | 1.35E-16 | 2.96E-20 | 2.30E-20 |
| 1300 | 3.17E-16 | 3.24E-16 | 1.32E-19 | 1.03E-19 |
| 1400 | 6.81E-16 | 6.95E-16 | 4.85E-19 | 3.81E-19 |
| 1500 | 1.34E-15 | 1.36E-15 | 1.52E-18 | 1.20E-18 |
| 1600 | 2.44E-15 | 2.48E-15 | 4.18E-18 | 3.31E-18 |
| 1700 | 4.19E-15 | 4.26E-15 | 1.03E-17 | 8.21E-18 |
| 1800 | 6.84E-15 | 6.94E-15 | 2.33E-17 | 1.86E-17 |
| 1900 | 1.07E-14 | 1.08E-14 | 4.87E-17 | 3.88E-17 |
| 2000 | 1.60E-14 | 1.62E-14 | 9.53E-17 | 7.61E-17 |
| 2100 | 2.33E-14 | 2.36E-14 | 1.76E-16 | 1.41E-16 |
| 2200 | 3.29E-14 | 3.33E-14 | 3.09E-16 | 2.48E-16 |
| 2300 | 4.54E-14 | 4.59E-14 | 5.21E-16 | 4.17E-16 |
| 2400 | 6.12E-14 | 6.19E-14 | 8.44E-16 | 6.76E-16 |
| 2500 | 8.09E-14 | 8.17E-14 | 1.32E-15 | 1.06E-15 |
| 2600 | 1.05E-13 | 1.06E-13 | 2.01E-15 | 1.61E-15 |
| 2700 | 1.34E-13 | 1.35E-13 | 2.97E-15 | 2.38E-15 |
| 2800 | 1.69E-13 | 1.71E-13 | 4.29E-15 | 3.44E-15 |
| 2900 | 2.11E-13 | 2.13E-13 | 6.05E-15 | 4.85E-15 |
| 3000 | 2.60E-13 | 2.62E-13 | 8.38E-15 | 6.71E-15 |

All of the rate constants are in cm^3^molecule^-1^ s^-1^.

^a^calculated rate constant of P2 pathways at the M06-2X/maTZ with Shavit correction.

^b^calculated rate constant of P3 pathways at the M06-2X/maTZ with Shavit correction.

**Table S16.** The high-pressure limit rate constants of P2 and P3 generation pathways and Shavit tunneling correction.

|  | CCSD(T)/ aTZ//MP2/aTZ | | MP2/aTZ | |  | M06-2X/maTZ | |  | |
| --- | --- | --- | --- | --- | --- | --- | --- | --- | --- |
| T/K | k^Eckart^ | k^Shavit^ | k^Eckart^ | k^Shavit^ | | k^Eckart^ | k^Shavit^ | |  |
| 230 | 8.37E-30 | 1.35E-31 | 1.04E-36 | 5.08E-39 | | 6.83E-34 | 2.29E-34 | |  |
| 232 | 1.04E-29 | 1.95E-31 | 1.41E-36 | 8.46E-39 | | 9.94E-34 | 3.47E-34 | |  |
| 240 | 2.48E-29 | 7.84E-31 | 4.73E-36 | 5.99E-38 | | 4.26E-33 | 1.71E-33 | |  |
| 250 | 7.21E-29 | 3.95E-30 | 2.13E-35 | 5.81E-37 | | 2.36E-32 | 1.09E-32 | |  |
| 260 | 2.06E-28 | 1.76E-29 | 9.38E-35 | 4.74E-36 | | 1.17E-31 | 6.07E-32 | |  |
| 270 | 5.71E-28 | 7.01E-29 | 4.02E-34 | 3.31E-35 | | 5.21E-31 | 2.97E-31 | |  |
| 280 | 1.54E-27 | 2.54E-28 | 1.65E-33 | 2.01E-34 | | 2.12E-30 | 1.30E-30 | |  |
| 290 | 3.99E-27 | 8.41E-28 | 6.50E-33 | 1.08E-33 | | 7.88E-30 | 5.16E-30 | |  |
| 298 | 8.33E-27 | 2.07E-27 | 1.87E-32 | 3.84E-33 | | 2.13E-29 | 1.45E-29 | |  |
| 298.15 | 8.45E-27 | 2.11E-27 | 1.91E-32 | 3.93E-33 | | 2.17E-29 | 1.48E-29 | |  |
| 300 | 9.98E-27 | 2.58E-27 | 2.43E-32 | 5.21E-33 | | 2.71E-29 | 1.87E-29 | |  |
| 310 | 2.41E-26 | 7.35E-27 | 8.60E-32 | 2.27E-32 | | 8.66E-29 | 6.24E-29 | |  |
| 320 | 5.59E-26 | 1.97E-26 | 2.88E-31 | 9.03E-32 | | 2.59E-28 | 1.94E-28 | |  |
| 330 | 1.25E-25 | 4.97E-26 | 9.17E-31 | 3.31E-31 | | 7.28E-28 | 5.63E-28 | |  |
| 340 | 2.70E-25 | 1.19E-25 | 2.76E-30 | 1.12E-30 | | 1.94E-27 | 1.54E-27 | |  |
| 350 | 5.64E-25 | 2.71E-25 | 7.92E-30 | 3.56E-30 | | 4.88E-27 | 3.98E-27 | |  |
| 360 | 1.14E-24 | 5.91E-25 | 2.16E-29 | 1.06E-29 | | 1.17E-26 | 9.77E-27 | |  |
| 374 | 2.90E-24 | 1.64E-24 | 8.19E-29 | 4.44E-29 | | 3.73E-26 | 3.18E-26 | |  |
| 380 | 4.25E-24 | 2.49E-24 | 1.41E-28 | 7.95E-29 | | 5.97E-26 | 5.14E-26 | |  |
| 390 | 7.86E-24 | 4.85E-24 | 3.39E-28 | 2.02E-28 | | 1.27E-25 | 1.11E-25 | |  |
| 400 | 1.42E-23 | 9.13E-24 | 7.82E-28 | 4.89E-28 | | 2.61E-25 | 2.30E-25 | |  |
| 410 | 2.49E-23 | 1.67E-23 | 1.74E-27 | 1.14E-27 | | 5.18E-25 | 4.63E-25 | |  |
| 424 | 5.28E-23 | 3.72E-23 | 5.05E-27 | 3.47E-27 | | 1.29E-24 | 1.17E-24 | |  |
| 430 | 7.20E-23 | 5.16E-23 | 7.82E-27 | 5.49E-27 | | 1.87E-24 | 1.70E-24 | |  |
| 440 | 1.19E-22 | 8.73E-23 | 1.58E-26 | 1.14E-26 | | 3.41E-24 | 3.13E-24 | |  |
| 450 | 1.91E-22 | 1.45E-22 | 3.11E-26 | 2.31E-26 | | 6.05E-24 | 5.60E-24 | |  |
| 460 | 3.03E-22 | 2.34E-22 | 5.95E-26 | 4.53E-26 | | 1.05E-23 | 9.78E-24 | |  |
| 470 | 4.73E-22 | 3.73E-22 | 1.11E-25 | 8.63E-26 | | 1.79E-23 | 1.67E-23 | |  |
| 480 | 7.25E-22 | 5.82E-22 | 2.02E-25 | 1.60E-25 | | 2.97E-23 | 2.80E-23 | |  |
| 490 | 1.09E-21 | 8.92E-22 | 3.60E-25 | 2.91E-25 | | 4.86E-23 | 4.59E-23 | |  |
| 500 | 1.63E-21 | 1.35E-21 | 6.28E-25 | 5.15E-25 | | 7.78E-23 | 7.39E-23 | |  |
| 510 | 2.38E-21 | 2.00E-21 | 1.07E-24 | 8.94E-25 | | 1.23E-22 | 1.17E-22 | |  |
| 520 | 3.45E-21 | 2.93E-21 | 1.80E-24 | 1.52E-24 | | 1.90E-22 | 1.82E-22 | |  |
| 530 | 4.93E-21 | 4.24E-21 | 2.97E-24 | 2.53E-24 | | 2.90E-22 | 2.78E-22 | |  |
| 540 | 6.97E-21 | 6.05E-21 | 4.81E-24 | 4.15E-24 | | 4.36E-22 | 4.20E-22 | |  |
| 550 | 9.73E-21 | 8.53E-21 | 7.66E-24 | 6.67E-24 | | 6.47E-22 | 6.24E-22 | |  |
| 560 | 1.34E-20 | 1.19E-20 | 1.20E-23 | 1.06E-23 | | 9.48E-22 | 9.16E-22 | |  |
| 570 | 1.84E-20 | 1.64E-20 | 1.86E-23 | 1.65E-23 | | 1.37E-21 | 1.33E-21 | |  |
| 580 | 2.49E-20 | 2.24E-20 | 2.83E-23 | 2.53E-23 | | 1.96E-21 | 1.90E-21 | |  |
| 590 | 3.34E-20 | 3.02E-20 | 4.26E-23 | 3.84E-23 | | 2.77E-21 | 2.69E-21 | |  |
| 600 | 4.44E-20 | 4.04E-20 | 6.34E-23 | 5.74E-23 | | 3.87E-21 | 3.77E-21 | |  |
| 610 | 5.86E-20 | 5.36E-20 | 9.30E-23 | 8.48E-23 | | 5.36E-21 | 5.23E-21 | |  |
| 620 | 7.67E-20 | 7.06E-20 | 1.35E-22 | 1.24E-22 | | 7.34E-21 | 7.18E-21 | |  |
| 630 | 9.96E-20 | 9.21E-20 | 1.94E-22 | 1.79E-22 | | 9.97E-21 | 9.76E-21 | |  |
| 637 | 1.19E-19 | 1.10E-19 | 2.48E-22 | 2.29E-22 | | 1.23E-20 | 1.20E-20 | |  |
| 640 | 1.28E-19 | 1.19E-19 | 2.75E-22 | 2.55E-22 | | 1.34E-20 | 1.31E-20 | |  |
| 650 | 1.64E-19 | 1.53E-19 | 3.87E-22 | 3.60E-22 | | 1.79E-20 | 1.76E-20 | |  |
| 660 | 2.09E-19 | 1.96E-19 | 5.39E-22 | 5.04E-22 | | 2.37E-20 | 2.33E-20 | |  |
| 670 | 2.64E-19 | 2.48E-19 | 7.44E-22 | 6.98E-22 | | 3.11E-20 | 3.06E-20 | |  |
| 680 | 3.31E-19 | 3.12E-19 | 1.02E-21 | 9.58E-22 | | 4.06E-20 | 4.00E-20 | |  |
| 690 | 4.13E-19 | 3.91E-19 | 1.38E-21 | 1.30E-21 | | 5.26E-20 | 5.18E-20 | |  |
| 700 | 5.12E-19 | 4.86E-19 | 1.86E-21 | 1.76E-21 | | 6.76E-20 | 6.67E-20 | |  |
| 800 | 3.39E-18 | 3.28E-18 | 2.48E-20 | 2.40E-20 | | 6.08E-19 | 6.03E-19 | |  |
| 900 | 1.53E-17 | 1.50E-17 | 1.94E-19 | 1.90E-19 | | 3.49E-18 | 3.47E-18 | |  |
| 1000 | 5.29E-17 | 5.23E-17 | 1.04E-18 | 1.02E-18 | | 1.46E-17 | 1.45E-17 | |  |
| 1100 | 1.50E-16 | 1.48E-16 | 4.18E-18 | 4.14E-18 | | 4.80E-17 | 4.79E-17 | |  |
| 1200 | 3.63E-16 | 3.61E-16 | 1.37E-17 | 1.36E-17 | | 1.32E-16 | 1.32E-16 | |  |
| 1300 | 7.81E-16 | 7.77E-16 | 3.78E-17 | 3.77E-17 | | 3.18E-16 | 3.17E-16 | |  |
| 1400 | 1.53E-15 | 1.52E-15 | 9.19E-17 | 9.16E-17 | | 6.82E-16 | 6.81E-16 | |  |
| 1500 | 2.77E-15 | 2.76E-15 | 2.01E-16 | 2.00E-16 | | 1.34E-15 | 1.34E-15 | |  |
| 1600 | 4.71E-15 | 4.70E-15 | 4.03E-16 | 4.02E-16 | | 2.44E-15 | 2.44E-15 | |  |
| 1700 | 7.60E-15 | 7.59E-15 | 7.50E-16 | 7.49E-16 | | 4.19E-15 | 4.19E-15 | |  |
| 1800 | 1.17E-14 | 1.17E-14 | 1.32E-15 | 1.31E-15 | | 6.84E-15 | 6.83E-15 | |  |
| 1900 | 1.74E-14 | 1.74E-14 | 2.19E-15 | 2.19E-15 | | 1.07E-14 | 1.07E-14 | |  |
| 2000 | 2.50E-14 | 2.50E-14 | 3.49E-15 | 3.49E-15 | | 1.60E-14 | 1.60E-14 | |  |
| 2100 | 3.49E-14 | 3.49E-14 | 5.36E-15 | 5.35E-15 | | 2.33E-14 | 2.33E-14 | |  |
| 2200 | 4.76E-14 | 4.75E-14 | 7.95E-15 | 7.94E-15 | | 3.29E-14 | 3.29E-14 | |  |
| 2300 | 6.34E-14 | 6.34E-14 | 1.14E-14 | 1.14E-14 | | 4.53E-14 | 4.53E-14 | |  |
| 2400 | 8.29E-14 | 8.28E-14 | 1.61E-14 | 1.61E-14 | | 6.11E-14 | 6.11E-14 | |  |
| 2500 | 1.06E-13 | 1.06E-13 | 2.21E-14 | 2.20E-14 | | 8.08E-14 | 8.08E-14 | |  |
| 2600 | 1.35E-13 | 1.35E-13 | 2.96E-14 | 2.96E-14 | | 1.05E-13 | 1.05E-13 | |  |
| 2700 | 1.68E-13 | 1.68E-13 | 3.91E-14 | 3.91E-14 | | 1.34E-13 | 1.34E-13 | |  |
| 2800 | 2.07E-13 | 2.07E-13 | 5.08E-14 | 5.08E-14 | | 1.69E-13 | 1.69E-13 | |  |
| 2900 | 2.52E-13 | 2.52E-13 | 6.50E-14 | 6.50E-14 | | 2.10E-13 | 2.10E-13 | |  |
| 3000 | 3.05E-13 | 3.05E-13 | 8.20E-14 | 8.20E-14 | | 2.59E-13 | 2.58E-13 | |  |

**Table S17**. High pressure limit rate constants calculated by TST theory at different levels for path 3.

|  | CCSD(T)/ aTZ//MP2/aTZ | | MP2/aTZ | | M06-2X/maTZ | |
| --- | --- | --- | --- | --- | --- | --- |
| T/K | k^Eckart^ | k^Shavit^ | k^Eckart^ | k^Shavit^ | k^Eckart^ | k^Shavit^ |
| 230 | 5.68E-27 | 3.90E-66 | 4.79E-27 | 1.62E-66 | 9.13E-53 | 4.54E-68 |
| 232 | 5.77E-27 | 1.13E-65 | 4.87E-27 | 4.72E-66 | 1.18E-52 | 1.34E-67 |
| 240 | 6.18E-27 | 6.66E-64 | 5.21E-27 | 2.86E-64 | 3.32E-52 | 8.55E-66 |
| 250 | 6.74E-27 | 7.53E-62 | 5.68E-27 | 3.35E-62 | 1.21E-51 | 1.06E-63 |
| 260 | 7.38E-27 | 5.90E-60 | 6.22E-27 | 2.71E-60 | 4.40E-51 | 9.02E-62 |
| 270 | 8.10E-27 | 3.34E-58 | 6.82E-27 | 1.58E-58 | 1.61E-50 | 5.53E-60 |
| 280 | 8.92E-27 | 1.42E-56 | 7.50E-27 | 6.88E-57 | 5.89E-50 | 2.53E-58 |
| 290 | 9.84E-27 | 4.64E-55 | 8.27E-27 | 2.31E-55 | 2.16E-49 | 8.87E-57 |
| 298 | 1.07E-26 | 6.39E-54 | 8.95E-27 | 3.24E-54 | 6.11E-49 | 1.29E-55 |
| 298.15 | 1.07E-26 | 6.70E-54 | 8.96E-27 | 3.40E-54 | 6.23E-49 | 1.35E-55 |
| 300 | 1.09E-26 | 1.20E-53 | 9.13E-27 | 6.12E-54 | 7.93E-49 | 2.45E-55 |
| 310 | 1.21E-26 | 2.53E-52 | 1.01E-26 | 1.31E-52 | 2.92E-48 | 5.48E-54 |
| 320 | 1.34E-26 | 4.38E-51 | 1.12E-26 | 2.33E-51 | 1.07E-47 | 1.01E-52 |
| 330 | 1.49E-26 | 6.39E-50 | 1.25E-26 | 3.46E-50 | 3.96E-47 | 1.56E-51 |
| 340 | 1.66E-26 | 7.95E-49 | 1.39E-26 | 4.38E-49 | 1.46E-46 | 2.04E-50 |
| 350 | 1.85E-26 | 8.57E-48 | 1.55E-26 | 4.80E-48 | 5.40E-46 | 2.32E-49 |
| 360 | 2.07E-26 | 8.09E-47 | 1.73E-26 | 4.61E-47 | 1.99E-45 | 2.30E-48 |
| 374 | 2.42E-26 | 1.53E-45 | 2.02E-26 | 8.91E-46 | 1.24E-44 | 4.64E-47 |
| 380 | 2.59E-26 | 5.05E-45 | 2.17E-26 | 2.96E-45 | 2.71E-44 | 1.57E-46 |
| 390 | 2.91E-26 | 3.41E-44 | 2.43E-26 | 2.03E-44 | 9.96E-44 | 1.11E-45 |
| 400 | 3.27E-26 | 2.09E-43 | 2.73E-26 | 1.26E-43 | 3.64E-43 | 7.08E-45 |
| 410 | 3.68E-26 | 1.17E-42 | 3.07E-26 | 7.14E-43 | 1.31E-42 | 4.14E-44 |
| 424 | 4.35E-26 | 1.14E-41 | 3.62E-26 | 7.08E-42 | 7.74E-42 | 4.26E-43 |
| 430 | 4.67E-26 | 2.90E-41 | 3.89E-26 | 1.81E-41 | 1.64E-41 | 1.11E-42 |
| 440 | 5.27E-26 | 1.29E-40 | 4.39E-26 | 8.15E-41 | 5.61E-41 | 5.12E-42 |
| 450 | 5.95E-26 | 5.39E-40 | 4.96E-26 | 3.43E-40 | 1.88E-40 | 2.21E-41 |
| 460 | 6.73E-26 | 2.11E-39 | 5.61E-26 | 1.36E-39 | 6.12E-40 | 8.98E-41 |
| 470 | 7.61E-26 | 7.82E-39 | 6.34E-26 | 5.08E-39 | 1.94E-39 | 3.44E-40 |
| 480 | 8.62E-26 | 2.74E-38 | 7.18E-26 | 1.80E-38 | 5.95E-39 | 1.25E-39 |
| 490 | 9.77E-26 | 9.13E-38 | 8.13E-26 | 6.04E-38 | 1.77E-38 | 4.29E-39 |
| 500 | 1.11E-25 | 2.90E-37 | 9.22E-26 | 1.93E-37 | 5.12E-38 | 1.40E-38 |
| 510 | 1.26E-25 | 8.79E-37 | 1.05E-25 | 5.91E-37 | 1.43E-37 | 4.40E-38 |
| 520 | 1.43E-25 | 2.56E-36 | 1.19E-25 | 1.73E-36 | 3.90E-37 | 1.32E-37 |
| 530 | 1.62E-25 | 7.15E-36 | 1.35E-25 | 4.88E-36 | 1.03E-36 | 3.79E-37 |
| 540 | 1.84E-25 | 1.92E-35 | 1.53E-25 | 1.32E-35 | 2.64E-36 | 1.05E-36 |
| 550 | 2.10E-25 | 4.99E-35 | 1.74E-25 | 3.46E-35 | 6.57E-36 | 2.80E-36 |
| 560 | 2.39E-25 | 1.25E-34 | 1.99E-25 | 8.73E-35 | 1.59E-35 | 7.24E-36 |
| 570 | 2.72E-25 | 3.05E-34 | 2.26E-25 | 2.14E-34 | 3.76E-35 | 1.81E-35 |
| 580 | 3.10E-25 | 7.19E-34 | 2.58E-25 | 5.07E-34 | 8.66E-35 | 4.38E-35 |
| 590 | 3.53E-25 | 1.65E-33 | 2.93E-25 | 1.17E-33 | 1.94E-34 | 1.03E-34 |
| 600 | 4.02E-25 | 3.67E-33 | 3.35E-25 | 2.62E-33 | 4.26E-34 | 2.35E-34 |
| 610 | 4.59E-25 | 7.97E-33 | 3.82E-25 | 5.72E-33 | 9.14E-34 | 5.24E-34 |
| 620 | 5.23E-25 | 1.69E-32 | 4.36E-25 | 1.22E-32 | 1.92E-33 | 1.14E-33 |
| 630 | 5.97E-25 | 3.50E-32 | 4.97E-25 | 2.53E-32 | 3.93E-33 | 2.41E-33 |
| 637 | 6.55E-25 | 5.74E-32 | 5.45E-25 | 4.17E-32 | 6.43E-33 | 4.03E-33 |
| 640 | 6.82E-25 | 7.07E-32 | 5.68E-25 | 5.15E-32 | 7.91E-33 | 5.00E-33 |
| 650 | 7.79E-25 | 1.40E-31 | 6.48E-25 | 1.03E-31 | 1.56E-32 | 1.01E-32 |
| 660 | 8.90E-25 | 2.72E-31 | 7.41E-25 | 2.00E-31 | 3.02E-32 | 2.02E-32 |
| 670 | 1.02E-24 | 5.17E-31 | 8.47E-25 | 3.82E-31 | 5.75E-32 | 3.92E-32 |
| 680 | 1.16E-24 | 9.67E-31 | 9.68E-25 | 7.17E-31 | 1.08E-31 | 7.49E-32 |
| 690 | 1.33E-24 | 1.77E-30 | 1.11E-24 | 1.32E-30 | 1.98E-31 | 1.41E-31 |
| 700 | 1.52E-24 | 3.20E-30 | 1.27E-24 | 2.40E-30 | 3.58E-31 | 2.59E-31 |
| 800 | 5.86E-24 | 5.26E-28 | 4.90E-24 | 4.08E-28 | 6.31E-29 | 5.20E-29 |
| 900 | 2.29E-23 | 2.84E-26 | 1.92E-23 | 2.27E-26 | 3.79E-27 | 3.35E-27 |
| 1000 | 8.91E-23 | 7.07E-25 | 7.54E-23 | 5.77E-25 | 1.06E-25 | 9.73E-26 |
| 1100 | 3.42E-22 | 9.98E-24 | 2.91E-22 | 8.30E-24 | 1.67E-24 | 1.58E-24 |
| 1200 | 1.27E-21 | 9.21E-23 | 1.09E-21 | 7.78E-23 | 1.71E-23 | 1.64E-23 |
| 1300 | 4.43E-21 | 6.14E-22 | 3.83E-21 | 5.25E-22 | 1.26E-22 | 1.22E-22 |
| 1400 | 1.44E-20 | 3.16E-21 | 1.25E-20 | 2.73E-21 | 7.10E-22 | 6.95E-22 |
| 1500 | 4.33E-20 | 1.32E-20 | 3.80E-20 | 1.16E-20 | 3.23E-21 | 3.18E-21 |
| 1600 | 1.20E-19 | 4.69E-20 | 1.06E-19 | 4.13E-20 | 1.24E-20 | 1.22E-20 |
| 1700 | 3.07E-19 | 1.44E-19 | 2.74E-19 | 1.28E-19 | 4.08E-20 | 4.04E-20 |
| 1800 | 7.33E-19 | 3.96E-19 | 6.56E-19 | 3.54E-19 | 1.19E-19 | 1.18E-19 |
| 1900 | 1.64E-18 | 9.86E-19 | 1.47E-18 | 8.86E-19 | 3.15E-19 | 3.13E-19 |
| 2000 | 3.44E-18 | 2.26E-18 | 3.11E-18 | 2.04E-18 | 7.59E-19 | 7.55E-19 |
| 2100 | 6.83E-18 | 4.80E-18 | 6.21E-18 | 4.36E-18 | 1.70E-18 | 1.69E-18 |
| 2200 | 1.29E-17 | 9.60E-18 | 1.18E-17 | 8.76E-18 | 3.55E-18 | 3.54E-18 |
| 2300 | 2.34E-17 | 1.82E-17 | 2.15E-17 | 1.67E-17 | 7.00E-18 | 6.98E-18 |
| 2400 | 4.08E-17 | 3.28E-17 | 3.75E-17 | 3.02E-17 | 1.31E-17 | 1.31E-17 |
| 2500 | 6.85E-17 | 5.68E-17 | 6.31E-17 | 5.24E-17 | 2.35E-17 | 2.35E-17 |
| 2600 | 1.11E-16 | 9.47E-17 | 1.03E-16 | 8.76E-17 | 4.05E-17 | 4.04E-17 |
| 2700 | 1.76E-16 | 1.53E-16 | 1.63E-16 | 1.42E-16 | 6.72E-17 | 6.71E-17 |
| 2800 | 2.70E-16 | 2.39E-16 | 2.51E-16 | 2.22E-16 | 1.08E-16 | 1.08E-16 |
| 2900 | 4.04E-16 | 3.63E-16 | 3.77E-16 | 3.38E-16 | 1.68E-16 | 1.68E-16 |
| 3000 | 5.92E-16 | 5.39E-16 | 5.53E-16 | 5.03E-16 | 2.56E-16 | 2.56E-16 |

**Table S18**. High pressure limit rate constants calculated by TST theory at different levels for path 4.

|  | CCSD(T)/ aTZ//MP2/aTZ | | MP2/aTZ | | M06-2X/maTZ | |
| --- | --- | --- | --- | --- | --- | --- |
| T/K | k^Eckart^ | k^Shavit^ | k^Eckart^ | k^Shavit^ | k^Eckart^ | k^Shavit^ |
| 230 | 1.77E-56 | 6.38E-60 | 1.74E-52 | 1.44E-58 | 8.68E-58 | 7.88E-60 |
| 232 | 3.53E-56 | 1.50E-59 | 3.20E-52 | 3.52E-58 | 1.72E-57 | 1.78E-59 |
| 240 | 5.18E-55 | 3.98E-58 | 3.39E-51 | 1.11E-56 | 2.44E-56 | 4.15E-58 |
| 250 | 1.22E-53 | 1.83E-56 | 5.48E-50 | 6.19E-55 | 5.63E-55 | 1.63E-56 |
| 260 | 2.35E-52 | 6.46E-55 | 7.44E-49 | 2.56E-53 | 1.10E-53 | 5.09E-55 |
| 270 | 3.80E-51 | 1.79E-53 | 8.56E-48 | 8.09E-52 | 1.84E-52 | 1.26E-53 |
| 280 | 5.20E-50 | 3.98E-52 | 8.52E-47 | 2.00E-50 | 2.69E-51 | 2.56E-52 |
| 290 | 6.09E-49 | 7.27E-51 | 7.43E-46 | 3.94E-49 | 3.46E-50 | 4.39E-51 |
| 298 | 3.98E-48 | 6.52E-50 | 3.83E-45 | 3.70E-48 | 2.44E-49 | 3.76E-50 |
| 298.15 | 4.12E-48 | 6.78E-50 | 3.96E-45 | 3.85E-48 | 2.54E-49 | 3.92E-50 |
| 300 | 6.26E-48 | 1.11E-49 | 5.71E-45 | 6.35E-48 | 3.94E-49 | 6.36E-50 |
| 310 | 5.69E-47 | 1.44E-48 | 3.96E-44 | 8.51E-47 | 3.96E-48 | 7.91E-49 |
| 320 | 4.60E-46 | 1.60E-47 | 2.47E-43 | 9.66E-46 | 3.61E-47 | 8.61E-48 |
| 330 | 3.35E-45 | 1.55E-46 | 1.41E-42 | 9.42E-45 | 2.96E-46 | 8.24E-47 |
| 340 | 2.20E-44 | 1.33E-45 | 7.39E-42 | 8.00E-44 | 2.20E-45 | 7.02E-46 |
| 350 | 1.33E-43 | 1.01E-44 | 3.59E-41 | 5.99E-43 | 1.49E-44 | 5.33E-45 |
| 360 | 7.31E-43 | 6.90E-44 | 1.63E-40 | 4.00E-42 | 9.28E-44 | 3.68E-44 |
| 374 | 6.99E-42 | 8.61E-43 | 1.21E-39 | 4.78E-41 | 1.04E-42 | 4.66E-43 |
| 380 | 1.77E-41 | 2.40E-42 | 2.76E-39 | 1.31E-40 | 2.81E-42 | 1.32E-42 |
| 390 | 7.82E-41 | 1.24E-41 | 1.04E-38 | 6.52E-40 | 1.38E-41 | 6.93E-42 |
| 400 | 3.24E-40 | 5.95E-41 | 3.73E-38 | 2.99E-39 | 6.32E-41 | 3.38E-41 |
| 410 | 1.26E-39 | 2.64E-40 | 1.27E-37 | 1.27E-38 | 2.72E-40 | 1.54E-40 |
| 424 | 7.73E-39 | 1.90E-39 | 6.53E-37 | 8.58E-38 | 1.89E-39 | 1.14E-39 |
| 430 | 1.63E-38 | 4.26E-39 | 1.28E-36 | 1.87E-37 | 4.19E-39 | 2.60E-39 |
| 440 | 5.41E-38 | 1.56E-38 | 3.81E-36 | 6.53E-37 | 1.52E-38 | 9.75E-39 |
| 450 | 1.71E-37 | 5.41E-38 | 1.09E-35 | 2.15E-36 | 5.20E-38 | 3.47E-38 |
| 460 | 5.20E-37 | 1.78E-37 | 3.00E-35 | 6.74E-36 | 1.70E-37 | 1.17E-37 |
| 470 | 1.50E-36 | 5.57E-37 | 7.97E-35 | 2.01E-35 | 5.31E-37 | 3.76E-37 |
| 480 | 4.21E-36 | 1.67E-36 | 2.04E-34 | 5.71E-35 | 1.59E-36 | 1.15E-36 |
| 490 | 1.14E-35 | 4.77E-36 | 5.08E-34 | 1.56E-34 | 4.57E-36 | 3.39E-36 |
| 500 | 2.94E-35 | 1.31E-35 | 1.21E-33 | 4.07E-34 | 1.26E-35 | 9.58E-36 |
| 510 | 7.39E-35 | 3.47E-35 | 2.83E-33 | 1.03E-33 | 3.36E-35 | 2.60E-35 |
| 520 | 1.80E-34 | 8.86E-35 | 6.40E-33 | 2.49E-33 | 8.67E-35 | 6.81E-35 |
| 530 | 4.27E-34 | 2.19E-34 | 1.41E-32 | 5.86E-33 | 2.15E-34 | 1.72E-34 |
| 540 | 9.73E-34 | 5.22E-34 | 3.04E-32 | 1.33E-32 | 5.20E-34 | 4.22E-34 |
| 550 | 2.16E-33 | 1.21E-33 | 6.32E-32 | 2.94E-32 | 1.22E-33 | 1.00E-33 |
| 560 | 4.76E-33 | 2.73E-33 | 1.30E-31 | 6.32E-32 | 2.77E-33 | 2.31E-33 |
| 570 | 1.01E-32 | 5.97E-33 | 2.59E-31 | 1.32E-31 | 6.14E-33 | 5.18E-33 |
| 580 | 2.08E-32 | 1.28E-32 | 5.04E-31 | 2.69E-31 | 1.33E-32 | 1.13E-32 |
| 590 | 4.21E-32 | 2.66E-32 | 9.67E-31 | 5.36E-31 | 2.80E-32 | 2.41E-32 |
| 600 | 8.29E-32 | 5.41E-32 | 1.81E-30 | 1.04E-30 | 5.78E-32 | 5.02E-32 |
| 610 | 1.61E-31 | 1.08E-31 | 3.35E-30 | 1.98E-30 | 1.17E-31 | 1.02E-31 |
| 620 | 3.08E-31 | 2.10E-31 | 6.04E-30 | 3.70E-30 | 2.29E-31 | 2.02E-31 |
| 630 | 5.75E-31 | 4.01E-31 | 1.08E-29 | 6.77E-30 | 4.45E-31 | 3.95E-31 |
| 637 | 8.81E-31 | 6.23E-31 | 1.59E-29 | 1.02E-29 | 6.97E-31 | 6.23E-31 |
| 640 | 1.06E-30 | 7.50E-31 | 1.88E-29 | 1.22E-29 | 8.43E-31 | 7.53E-31 |
| 650 | 1.91E-30 | 1.38E-30 | 3.25E-29 | 2.14E-29 | 1.58E-30 | 1.42E-30 |
| 660 | 3.40E-30 | 2.50E-30 | 5.49E-29 | 3.72E-29 | 2.87E-30 | 2.60E-30 |
| 670 | 5.94E-30 | 4.44E-30 | 9.20E-29 | 6.34E-29 | 5.21E-30 | 4.72E-30 |
| 680 | 1.02E-29 | 7.76E-30 | 1.51E-28 | 1.07E-28 | 9.17E-30 | 8.39E-30 |
| 690 | 1.75E-29 | 1.34E-29 | 2.46E-28 | 1.76E-28 | 1.60E-29 | 1.47E-29 |
| 700 | 2.91E-29 | 2.27E-29 | 3.94E-28 | 2.88E-28 | 2.76E-29 | 2.54E-29 |
| 800 | 2.65E-27 | 2.27E-27 | 2.44E-26 | 2.02E-26 | 3.09E-27 | 2.94E-27 |
| 900 | 9.53E-26 | 8.64E-26 | 6.50E-25 | 5.72E-25 | 1.28E-25 | 1.24E-25 |
| 1000 | 1.74E-24 | 1.63E-24 | 9.20E-24 | 8.43E-24 | 2.65E-24 | 2.59E-24 |
| 1100 | 2.02E-23 | 1.92E-23 | 8.57E-23 | 8.07E-23 | 3.25E-23 | 3.21E-23 |
| 1200 | 1.61E-22 | 1.55E-22 | 5.77E-22 | 5.52E-22 | 2.72E-22 | 2.68E-22 |
| 1300 | 9.52E-22 | 9.25E-22 | 3.00E-21 | 2.91E-21 | 1.66E-21 | 1.65E-21 |
| 1400 | 4.46E-21 | 4.35E-21 | 1.27E-20 | 1.24E-20 | 8.02E-21 | 7.96E-21 |
| 1500 | 1.71E-20 | 1.69E-20 | 4.52E-20 | 4.44E-20 | 3.19E-20 | 3.17E-20 |
| 1600 | 5.69E-20 | 5.61E-20 | 1.41E-19 | 1.38E-19 | 1.08E-19 | 1.08E-19 |
| 1700 | 1.66E-19 | 1.63E-19 | 3.88E-19 | 3.81E-19 | 3.21E-19 | 3.19E-19 |
| 1800 | 4.32E-19 | 4.27E-19 | 9.56E-19 | 9.48E-19 | 8.51E-19 | 8.48E-19 |
| 1900 | 1.03E-18 | 1.02E-18 | 2.19E-18 | 2.16E-18 | 2.06E-18 | 2.05E-18 |
| 2000 | 2.27E-18 | 2.24E-18 | 4.63E-18 | 4.59E-18 | 4.59E-18 | 4.58E-18 |
| 2100 | 4.63E-18 | 4.60E-18 | 9.20E-18 | 9.11E-18 | 9.53E-18 | 9.52E-18 |
| 2200 | 8.99E-18 | 8.92E-18 | 1.72E-17 | 1.71E-17 | 1.87E-17 | 1.87E-17 |
| 2300 | 1.65E-17 | 1.64E-17 | 3.08E-17 | 3.06E-17 | 3.48E-17 | 3.48E-17 |
| 2400 | 2.90E-17 | 2.88E-17 | 5.27E-17 | 5.24E-17 | 6.19E-17 | 6.18E-17 |
| 2500 | 4.89E-17 | 4.86E-17 | 8.67E-17 | 8.63E-17 | 1.05E-16 | 1.05E-16 |
| 2600 | 7.91E-17 | 7.90E-17 | 1.38E-16 | 1.37E-16 | 1.72E-16 | 1.72E-16 |
| 2700 | 1.25E-16 | 1.24E-16 | 2.13E-16 | 2.12E-16 | 2.72E-16 | 2.72E-16 |
| 2800 | 1.91E-16 | 1.90E-16 | 3.19E-16 | 3.18E-16 | 4.19E-16 | 4.19E-16 |
| 2900 | 2.84E-16 | 2.83E-16 | 4.67E-16 | 4.65E-16 | 6.26E-16 | 6.25E-16 |
| 3000 | 4.12E-16 | 4.10E-16 | 6.67E-16 | 6.66E-16 | 9.16E-16 | 9.15E-16 |

**Table S19**. High pressure limit rate constants calculated by TST theory at different levels for path 6.

|  | CCSD(T)/ aTZ//MP2/aTZ | | MP2/aTZ | | M06-2X/maTZ | |
| --- | --- | --- | --- | --- | --- | --- |
| T/K | k^Eckart^ | k^Shavit^ | k^Eckart^ | k^Shavit^ | k^Eckart^ | k^Shavit^ |
| 230 | 5.55E-21 | 1.35E-31 | 5.97E-21 | 5.08E-39 | 6.83E-34 | 2.29E-34 |
| 232 | 5.60E-21 | 1.95E-31 | 6.02E-21 | 8.46E-39 | 9.94E-34 | 3.47E-34 |
| 240 | 5.83E-21 | 7.84E-31 | 6.26E-21 | 5.99E-38 | 4.26E-33 | 1.71E-33 |
| 250 | 6.12E-21 | 3.95E-30 | 6.57E-21 | 5.81E-37 | 2.36E-32 | 1.09E-32 |
| 260 | 6.44E-21 | 1.76E-29 | 6.91E-21 | 4.74E-36 | 1.17E-31 | 6.07E-32 |
| 270 | 6.78E-21 | 7.01E-29 | 7.26E-21 | 3.31E-35 | 5.21E-31 | 2.97E-31 |
| 280 | 7.14E-21 | 2.54E-28 | 7.65E-21 | 2.01E-34 | 2.12E-30 | 1.30E-30 |
| 290 | 7.53E-21 | 8.41E-28 | 8.06E-21 | 1.08E-33 | 7.88E-30 | 5.16E-30 |
| 298 | 7.86E-21 | 2.07E-27 | 8.40E-21 | 3.84E-33 | 2.13E-29 | 1.45E-29 |
| 298.15 | 7.87E-21 | 2.11E-27 | 8.41E-21 | 3.93E-33 | 2.17E-29 | 1.48E-29 |
| 300 | 7.94E-21 | 2.58E-27 | 8.49E-21 | 5.21E-33 | 2.71E-29 | 1.87E-29 |
| 310 | 8.39E-21 | 7.35E-27 | 8.96E-21 | 2.27E-32 | 8.66E-29 | 6.24E-29 |
| 320 | 8.86E-21 | 1.97E-26 | 9.45E-21 | 9.03E-32 | 2.59E-28 | 1.94E-28 |
| 330 | 9.36E-21 | 4.97E-26 | 9.98E-21 | 3.31E-31 | 7.28E-28 | 5.63E-28 |
| 340 | 9.9E-21 | 1.19E-25 | 1.05E-20 | 1.12E-30 | 1.94E-27 | 1.54E-27 |
| 350 | 1.05E-20 | 2.71E-25 | 1.11E-20 | 3.56E-30 | 4.88E-27 | 3.98E-27 |
| 360 | 1.11E-20 | 5.91E-25 | 1.18E-20 | 1.06E-29 | 1.17E-26 | 9.77E-27 |
| 374 | 1.2E-20 | 1.64E-24 | 1.28E-20 | 4.44E-29 | 3.73E-26 | 3.18E-26 |
| 380 | 1.24E-20 | 2.49E-24 | 1.32E-20 | 7.95E-29 | 5.97E-26 | 5.14E-26 |
| 390 | 1.32E-20 | 4.85E-24 | 1.40E-20 | 2.02E-28 | 1.27E-25 | 1.11E-25 |
| 400 | 1.40E-20 | 9.13E-24 | 1.48E-20 | 4.89E-28 | 2.61E-25 | 2.3E-25 |
| 410 | 1.48E-20 | 1.67E-23 | 1.57E-20 | 1.14E-27 | 5.18E-25 | 4.63E-25 |
| 424 | 1.61E-20 | 3.72E-23 | 1.70E-20 | 3.47E-27 | 1.29E-24 | 1.17E-24 |
| 430 | 1.67E-20 | 5.16E-23 | 1.76E-20 | 5.49E-27 | 1.87E-24 | 1.7E-24 |
| 440 | 1.78E-20 | 8.73E-23 | 1.87E-20 | 1.14E-26 | 3.41E-24 | 3.13E-24 |
| 450 | 1.89E-20 | 1.45E-22 | 1.98E-20 | 2.31E-26 | 6.05E-24 | 5.6E-24 |
| 460 | 2.02E-20 | 2.34E-22 | 2.10E-20 | 4.53E-26 | 1.05E-23 | 9.78E-24 |
| 470 | 2.15E-20 | 3.73E-22 | 2.23E-20 | 8.63E-26 | 1.79E-23 | 1.67E-23 |
| 480 | 2.31E-20 | 5.82E-22 | 2.36E-20 | 1.6E-25 | 2.97E-23 | 2.8E-23 |
| 490 | 2.49E-20 | 8.92E-22 | 2.51E-20 | 2.91E-25 | 4.86E-23 | 4.59E-23 |
| 500 | 2.69E-20 | 1.35E-21 | 2.66E-20 | 5.15E-25 | 7.78E-23 | 7.39E-23 |
| 510 | 2.92E-20 | 2.00E-21 | 2.83E-20 | 8.94E-25 | 1.23E-22 | 1.17E-22 |
| 520 | 3.19E-20 | 2.93E-21 | 3.00E-20 | 1.52E-24 | 1.9E-22 | 1.82E-22 |
| 530 | 3.52E-20 | 4.24E-21 | 3.19E-20 | 2.53E-24 | 2.9E-22 | 2.78E-22 |
| 540 | 3.91E-20 | 6.05E-21 | 3.39E-20 | 4.15E-24 | 4.36E-22 | 4.20E-22 |
| 550 | 4.39E-20 | 8.53E-21 | 3.60E-20 | 6.67E-24 | 6.47E-22 | 6.24E-22 |
| 560 | 4.97E-20 | 1.19E-20 | 3.82E-20 | 1.06E-23 | 9.48E-22 | 9.16E-22 |
| 570 | 5.70E-20 | 1.64E-20 | 4.06E-20 | 1.65E-23 | 1.37E-21 | 1.33E-21 |
| 580 | 6.59E-20 | 2.24E-20 | 4.31E-20 | 2.53E-23 | 1.96E-21 | 1.9E-21 |
| 590 | 7.70E-20 | 3.02E-20 | 4.58E-20 | 3.84E-23 | 2.77E-21 | 2.69E-21 |
| 600 | 9.08E-20 | 4.04E-20 | 4.87E-20 | 5.74E-23 | 3.87E-21 | 3.77E-21 |
| 610 | 1.08E-19 | 5.36E-20 | 5.18E-20 | 8.48E-23 | 5.36E-21 | 5.23E-21 |
| 620 | 1.29E-19 | 7.06E-20 | 5.51E-20 | 1.24E-22 | 7.34E-21 | 7.18E-21 |
| 630 | 1.55E-19 | 9.21E-20 | 5.86E-20 | 1.79E-22 | 9.97E-21 | 9.76E-21 |
| 637 | 1.77E-19 | 1.10E-19 | 6.12E-20 | 2.29E-22 | 1.23E-20 | 1.20E-20 |
| 640 | 1.88E-19 | 1.19E-19 | 6.24E-20 | 2.55E-22 | 1.34E-20 | 1.31E-20 |
| 650 | 2.27E-19 | 1.53E-19 | 6.64E-20 | 3.60E-22 | 1.79E-20 | 1.76E-20 |
| 660 | 2.76E-19 | 1.96E-19 | 7.07E-20 | 5.04E-22 | 2.37E-20 | 2.33E-20 |
| 670 | 3.35E-19 | 2.48E-19 | 7.53E-20 | 6.98E-22 | 3.11E-20 | 3.06E-20 |
| 680 | 4.07E-19 | 3.12E-19 | 8.03E-20 | 9.58E-22 | 4.06E-20 | 4.00E-20 |
| 690 | 4.94E-19 | 3.91E-19 | 8.56E-20 | 1.30E-21 | 5.26E-20 | 5.18E-20 |
| 700 | 5.98E-19 | 4.86E-19 | 9.14E-20 | 1.76E-21 | 6.76E-20 | 6.67E-20 |
| 800 | 3.55E-18 | 3.28E-18 | 1.9E-19 | 2.40E-20 | 6.08E-19 | 6.03E-19 |
| 900 | 1.56E-17 | 1.50E-17 | 4.97E-19 | 1.90E-19 | 3.49E-18 | 3.47E-18 |
| 1000 | 5.35E-17 | 5.23E-17 | 1.59E-18 | 1.02E-18 | 1.46E-17 | 1.45E-17 |
| 1100 | 1.51E-16 | 1.48E-16 | 5.19E-18 | 4.14E-18 | 4.8E-17 | 4.79E-17 |
| 1200 | 3.65E-16 | 3.61E-16 | 1.55E-17 | 1.36E-17 | 1.32E-16 | 1.32E-16 |
| 1300 | 7.84E-16 | 7.77E-16 | 4.11E-17 | 3.77E-17 | 3.18E-16 | 3.17E-16 |
| 1400 | 1.53E-15 | 1.52E-15 | 9.78E-17 | 9.17E-17 | 6.82E-16 | 6.81E-16 |
| 1500 | 2.78E-15 | 2.76E-15 | 2.11E-16 | 2.01E-16 | 1.34E-15 | 1.34E-15 |
| 1600 | 4.73E-15 | 4.70E-15 | 4.21E-16 | 4.02E-16 | 2.44E-15 | 2.44E-15 |
| 1700 | 7.63E-15 | 7.59E-15 | 7.82E-16 | 7.51E-16 | 4.2E-15 | 4.19E-15 |
| 1800 | 1.18E-14 | 1.17E-14 | 1.37E-15 | 1.32E-15 | 6.84E-15 | 6.84E-15 |
| 1900 | 1.75E-14 | 1.74E-14 | 2.28E-15 | 2.2E-15 | 1.07E-14 | 1.07E-14 |
| 2000 | 2.51E-14 | 2.50E-14 | 3.64E-15 | 3.52E-15 | 1.6E-14 | 1.60E-14 |
| 2100 | 3.51E-14 | 3.49E-14 | 5.6E-15 | 5.41E-15 | 2.33E-14 | 2.33E-14 |
| 2200 | 4.79E-14 | 4.76E-14 | 8.33E-15 | 8.05E-15 | 3.29E-14 | 3.29E-14 |
| 2300 | 6.4E-14 | 6.36E-14 | 1.2E-14 | 1.16E-14 | 4.54E-14 | 4.54E-14 |
| 2400 | 8.37E-14 | 8.32E-14 | 1.7E-14 | 1.64E-14 | 6.12E-14 | 6.12E-14 |
| 2500 | 1.08E-13 | 1.07E-13 | 2.34E-14 | 2.26E-14 | 8.09E-14 | 8.09E-14 |
| 2600 | 1.37E-13 | 1.36E-13 | 3.17E-14 | 3.06E-14 | 1.05E-13 | 1.05E-13 |
| 2700 | 1.71E-13 | 1.7E-13 | 4.21E-14 | 4.07E-14 | 1.34E-13 | 1.34E-13 |
| 2800 | 2.11E-13 | 2.09E-13 | 5.5E-14 | 5.31E-14 | 1.69E-13 | 1.69E-13 |
| 2900 | 2.58E-13 | 2.56E-13 | 7.08E-14 | 6.85E-14 | 2.11E-13 | 2.11E-13 |
| 3000 | 3.12E-13 | 3.10E-13 | 9.01E-14 | 8.71E-14 | 2.6E-13 | 2.60E-13 |

**Table S20**. High pressure limit rate constants calculated by TST theory at different levels for P2 adducts via path 3 + path 4 + path 6.

|  | CCSD(T)/ aTZ//MP2 aTZ | | MP2 aTZ | | M06-2X/maTZ | |
| --- | --- | --- | --- | --- | --- | --- |
| T/K | k^Eckart^ | k^Shavit^ | k^Eckart^ | k^Shavit^ | k^Eckart^ | k^Shavit^ |
| 230 | 2.59E-50 | 9.36E-54 | 1.27E-46 | 1.05E-52 | 2.97E-50 | 2.70E-52 |
| 232 | 4.93E-50 | 2.09E-53 | 2.08E-46 | 2.29E-52 | 5.60E-50 | 5.82E-52 |
| 240 | 5.90E-49 | 4.53E-52 | 1.40E-45 | 4.58E-51 | 6.52E-49 | 1.11E-50 |
| 250 | 1.07E-47 | 1.60E-50 | 1.31E-44 | 1.48E-49 | 1.17E-47 | 3.40E-49 |
| 260 | 1.57E-46 | 4.31E-49 | 1.06E-43 | 3.65E-48 | 1.73E-46 | 7.99E-48 |
| 270 | 1.93E-45 | 9.09E-48 | 7.52E-43 | 7.11E-47 | 2.18E-45 | 1.49E-46 |
| 280 | 2.01E-44 | 1.54E-46 | 4.78E-42 | 1.12E-45 | 2.36E-44 | 2.25E-45 |
| 290 | 1.80E-43 | 2.15E-45 | 2.75E-41 | 1.46E-44 | 2.23E-43 | 2.83E-44 |
| 298 | 9.50E-43 | 1.56E-44 | 1.04E-40 | 1.00E-43 | 1.23E-42 | 1.90E-43 |
| 298.15 | 9.79E-43 | 1.61E-44 | 1.07E-40 | 1.04E-43 | 1.27E-42 | 1.96E-43 |
| 300 | 1.42E-42 | 2.51E-44 | 1.44E-40 | 1.60E-43 | 1.86E-42 | 3.00E-43 |
| 310 | 9.94E-42 | 2.51E-43 | 7.00E-40 | 1.50E-42 | 1.37E-41 | 2.74E-42 |
| 320 | 6.24E-41 | 2.17E-42 | 3.15E-39 | 1.23E-41 | 9.14E-41 | 2.18E-41 |
| 330 | 3.55E-40 | 1.65E-41 | 1.33E-38 | 8.87E-41 | 5.50E-40 | 1.53E-40 |
| 340 | 1.84E-39 | 1.11E-40 | 5.25E-38 | 5.69E-40 | 3.01E-39 | 9.61E-40 |
| 350 | 8.81E-39 | 6.72E-40 | 1.97E-37 | 3.28E-39 | 1.52E-38 | 5.43E-39 |
| 360 | 3.90E-38 | 3.68E-39 | 7.00E-37 | 1.72E-38 | 7.04E-38 | 2.79E-38 |
| 374 | 2.78E-37 | 3.42E-38 | 3.82E-36 | 1.51E-37 | 5.34E-37 | 2.39E-37 |
| 380 | 6.21E-37 | 8.46E-38 | 7.69E-36 | 3.65E-37 | 1.22E-36 | 5.73E-37 |
| 390 | 2.26E-36 | 3.60E-37 | 2.39E-35 | 1.49E-36 | 4.60E-36 | 2.31E-36 |
| 400 | 7.75E-36 | 1.42E-36 | 7.11E-35 | 5.71E-36 | 1.63E-35 | 8.72E-36 |
| 410 | 2.52E-35 | 5.27E-36 | 2.04E-34 | 2.04E-35 | 5.46E-35 | 3.09E-35 |
| 424 | 1.21E-34 | 2.98E-35 | 8.40E-34 | 1.10E-34 | 2.72E-34 | 1.64E-34 |
| 430 | 2.31E-34 | 6.04E-35 | 1.51E-33 | 2.20E-34 | 5.25E-34 | 3.25E-34 |
| 440 | 6.53E-34 | 1.88E-34 | 3.89E-33 | 6.66E-34 | 1.52E-33 | 9.76E-34 |
| 450 | 1.77E-33 | 5.59E-34 | 9.73E-33 | 1.92E-33 | 4.19E-33 | 2.79E-33 |
| 460 | 4.63E-33 | 1.58E-33 | 2.36E-32 | 5.30E-33 | 1.11E-32 | 7.64E-33 |
| 470 | 1.16E-32 | 4.30E-33 | 5.55E-32 | 1.40E-32 | 2.83E-32 | 2.00E-32 |
| 480 | 2.83E-32 | 1.12E-32 | 1.27E-31 | 3.56E-32 | 6.97E-32 | 5.06E-32 |
| 490 | 6.67E-32 | 2.80E-32 | 2.83E-31 | 8.71E-32 | 1.66E-31 | 1.23E-31 |
| 500 | 1.52E-31 | 6.77E-32 | 6.13E-31 | 2.06E-31 | 3.81E-31 | 2.89E-31 |
| 510 | 3.37E-31 | 1.58E-31 | 1.30E-30 | 4.70E-31 | 8.51E-31 | 6.59E-31 |
| 520 | 7.28E-31 | 3.58E-31 | 2.68E-30 | 1.04E-30 | 1.85E-30 | 1.45E-30 |
| 530 | 1.53E-30 | 7.87E-31 | 5.41E-30 | 2.24E-30 | 3.89E-30 | 3.12E-30 |
| 540 | 3.13E-30 | 1.68E-30 | 1.07E-29 | 4.70E-30 | 8.01E-30 | 6.50E-30 |
| 550 | 6.26E-30 | 3.49E-30 | 2.06E-29 | 9.59E-30 | 1.61E-29 | 1.32E-29 |
| 560 | 1.23E-29 | 7.07E-30 | 3.91E-29 | 1.91E-29 | 3.15E-29 | 2.62E-29 |
| 570 | 2.35E-29 | 1.40E-29 | 7.26E-29 | 3.71E-29 | 6.03E-29 | 5.09E-29 |
| 580 | 4.40E-29 | 2.71E-29 | 1.32E-28 | 7.05E-29 | 1.13E-28 | 9.65E-29 |
| 590 | 8.09E-29 | 5.12E-29 | 2.37E-28 | 1.31E-28 | 2.08E-28 | 1.79E-28 |
| 600 | 1.46E-28 | 9.49E-29 | 4.17E-28 | 2.39E-28 | 3.76E-28 | 3.27E-28 |
| 610 | 2.59E-28 | 1.72E-28 | 7.23E-28 | 4.29E-28 | 6.67E-28 | 5.84E-28 |
| 620 | 4.52E-28 | 3.08E-28 | 1.23E-27 | 7.54E-28 | 1.16E-27 | 1.02E-27 |
| 630 | 7.76E-28 | 5.39E-28 | 2.07E-27 | 1.30E-27 | 1.99E-27 | 1.77E-27 |
| 637 | 1.12E-27 | 7.91E-28 | 2.95E-27 | 1.89E-27 | 2.87E-27 | 2.57E-27 |
| 640 | 1.31E-27 | 9.30E-28 | 3.43E-27 | 2.21E-27 | 3.36E-27 | 3.00E-27 |
| 650 | 2.18E-27 | 1.58E-27 | 5.61E-27 | 3.71E-27 | 5.58E-27 | 5.02E-27 |
| 660 | 3.59E-27 | 2.63E-27 | 9.04E-27 | 6.11E-27 | 9.13E-27 | 8.27E-27 |
| 670 | 5.81E-27 | 4.33E-27 | 1.44E-26 | 9.93E-27 | 1.48E-26 | 1.34E-26 |
| 680 | 9.28E-27 | 7.03E-27 | 2.26E-26 | 1.59E-26 | 2.35E-26 | 2.15E-26 |
| 690 | 1.47E-26 | 1.13E-26 | 3.51E-26 | 2.52E-26 | 3.70E-26 | 3.40E-26 |
| 700 | 2.29E-26 | 1.78E-26 | 5.39E-26 | 3.93E-26 | 5.76E-26 | 5.31E-26 |
| 800 | 1.11E-24 | 9.48E-25 | 2.30E-24 | 1.90E-24 | 2.69E-24 | 2.56E-24 |
| 900 | 2.41E-23 | 2.18E-23 | 4.58E-23 | 4.04E-23 | 5.65E-23 | 5.47E-23 |
| 1000 | 2.97E-22 | 2.77E-22 | 5.26E-22 | 4.83E-22 | 6.72E-22 | 6.57E-22 |
| 1100 | 2.40E-21 | 2.28E-21 | 4.02E-21 | 3.78E-21 | 5.25E-21 | 5.17E-21 |
| 1200 | 1.41E-20 | 1.36E-20 | 2.25E-20 | 2.15E-20 | 3.00E-20 | 2.96E-20 |
| 1300 | 6.42E-20 | 6.25E-20 | 9.92E-20 | 9.59E-20 | 1.33E-19 | 1.32E-19 |
| 1400 | 2.41E-19 | 2.36E-19 | 3.60E-19 | 3.51E-19 | 4.89E-19 | 4.85E-19 |
| 1500 | 7.69E-19 | 7.57E-19 | 1.12E-18 | 1.10E-18 | 1.53E-18 | 1.52E-18 |
| 1600 | 2.16E-18 | 2.13E-18 | 3.06E-18 | 3.01E-18 | 4.21E-18 | 4.18E-18 |
| 1700 | 5.41E-18 | 5.35E-18 | 7.52E-18 | 7.42E-18 | 1.04E-17 | 1.03E-17 |
| 1800 | 1.24E-17 | 1.23E-17 | 1.69E-17 | 1.67E-17 | 2.34E-17 | 2.33E-17 |
| 1900 | 2.62E-17 | 2.60E-17 | 3.52E-17 | 3.49E-17 | 4.89E-17 | 4.87E-17 |
| 2000 | 5.20E-17 | 5.16E-17 | 6.87E-17 | 6.81E-17 | 9.55E-17 | 9.53E-17 |
| 2100 | 9.71E-17 | 9.65E-17 | 1.27E-16 | 1.26E-16 | 1.76E-16 | 1.76E-16 |
| 2200 | 1.73E-16 | 1.72E-16 | 2.22E-16 | 2.21E-16 | 3.10E-16 | 3.09E-16 |
| 2300 | 2.93E-16 | 2.92E-16 | 3.74E-16 | 3.72E-16 | 5.22E-16 | 5.21E-16 |
| 2400 | 4.80E-16 | 4.78E-16 | 6.05E-16 | 6.02E-16 | 8.46E-16 | 8.44E-16 |
| 2500 | 7.58E-16 | 7.55E-16 | 9.47E-16 | 9.43E-16 | 1.32E-15 | 1.32E-15 |
| 2600 | 1.16E-15 | 1.16E-15 | 1.44E-15 | 1.43E-15 | 2.01E-15 | 2.01E-15 |
| 2700 | 1.73E-15 | 1.72E-15 | 2.13E-15 | 2.12E-15 | 2.97E-15 | 2.97E-15 |
| 2800 | 2.52E-15 | 2.51E-15 | 3.07E-15 | 3.06E-15 | 4.29E-15 | 4.29E-15 |
| 2900 | 3.58E-15 | 3.56E-15 | 4.33E-15 | 4.32E-15 | 6.06E-15 | 6.05E-15 |
| 3000 | 4.98E-15 | 4.97E-15 | 6.00E-15 | 5.98E-15 | 8.39E-15 | 8.38E-15 |

**Table S21**. High pressure limit rate constants calculated by TST theory at different levels for path 5.

|  | MP2/aTZ | | M06-2X/maTZ | CCSD(T) /6-31++G(2df, pd)// MP2/aTZ | CCSD(T)-full/6-311++G(df,2p)// MP2/aTZ |
| --- | --- | --- | --- | --- | --- |
| T/K | k^Eckart^ | k^Shavit^ | k | k | k |
| 230 | 1.42E-14 | 1.32E-14 | 1.04E-10 | 7.45E-11 | 7.45E-11 |
| 232 | 1.47E-14 | 1.38E-14 | 1.00E-10 | 7.22E-11 | 7.22E-11 |
| 240 | 1.67E-14 | 1.63E-14 | 8.59E-11 | 6.42E-11 | 6.41E-11 |
| 250 | 1.94E-14 | 1.98E-14 | 7.20E-11 | 5.61E-11 | 5.60E-11 |
| 260 | 2.25E-14 | 2.37E-14 | 6.14E-11 | 4.96E-11 | 4.96E-11 |
| 270 | 2.58E-14 | 2.81E-14 | 5.31E-11 | 4.44E-11 | 4.43E-11 |
| 280 | 2.95E-14 | 3.30E-14 | 4.65E-11 | 4.02E-11 | 4.01E-11 |
| 290 | 3.35E-14 | 3.83E-14 | 4.12E-11 | 3.66E-11 | 3.66E-11 |
| 298 | 3.70E-14 | 4.29E-14 | 3.77E-11 | 3.42E-11 | 3.42E-11 |
| 298.15 | 3.71E-14 | 4.30E-14 | 3.76E-11 | 3.42E-11 | 3.41E-11 |
| 300 | 3.79E-14 | 4.41E-14 | 3.69E-11 | 3.37E-11 | 3.36E-11 |
| 310 | 4.26E-14 | 5.04E-14 | 3.33E-11 | 3.12E-11 | 3.11E-11 |
| 320 | 4.78E-14 | 5.72E-14 | 3.03E-11 | 2.91E-11 | 2.90E-11 |
| 330 | 5.33E-14 | 6.45E-14 | 2.78E-11 | 2.73E-11 | 2.72E-11 |
| 340 | 5.92E-14 | 7.24E-14 | 2.57E-11 | 2.57E-11 | 2.57E-11 |
| 350 | 6.55E-14 | 8.08E-14 | 2.39E-11 | 2.44E-11 | 2.43E-11 |
| 360 | 7.23E-14 | 8.97E-14 | 2.23E-11 | 2.32E-11 | 2.32E-11 |
| 374 | 8.25E-14 | 1.03E-13 | 2.05E-11 | 2.18E-11 | 2.18E-11 |
| 380 | 8.72E-14 | 1.09E-13 | 1.98E-11 | 2.13E-11 | 2.13E-11 |
| 390 | 9.53E-14 | 1.20E-13 | 1.88E-11 | 2.05E-11 | 2.05E-11 |
| 400 | 1.04E-13 | 1.31E-13 | 1.79E-11 | 1.98E-11 | 1.98E-11 |
| 410 | 1.13E-13 | 1.43E-13 | 1.71E-11 | 1.92E-11 | 1.92E-11 |
| 424 | 1.27E-13 | 1.61E-13 | 1.61E-11 | 1.84E-11 | 1.84E-11 |
| 430 | 1.33E-13 | 1.68E-13 | 1.57E-11 | 1.82E-11 | 1.81E-11 |
| 440 | 1.44E-13 | 1.82E-13 | 1.52E-11 | 1.77E-11 | 1.77E-11 |
| 450 | 1.55E-13 | 1.96E-13 | 1.47E-11 | 1.73E-11 | 1.73E-11 |
| 460 | 1.67E-13 | 2.11E-13 | 1.42E-11 | 1.70E-11 | 1.69E-11 |
| 470 | 1.79E-13 | 2.27E-13 | 1.38E-11 | 1.67E-11 | 1.66E-11 |
| 480 | 1.92E-13 | 2.43E-13 | 1.34E-11 | 1.64E-11 | 1.64E-11 |
| 490 | 2.06E-13 | 2.60E-13 | 1.31E-11 | 1.62E-11 | 1.61E-11 |
| 500 | 2.20E-13 | 2.78E-13 | 1.28E-11 | 1.59E-11 | 1.59E-11 |
| 510 | 2.35E-13 | 2.96E-13 | 1.25E-11 | 1.57E-11 | 1.57E-11 |
| 520 | 2.50E-13 | 3.15E-13 | 1.23E-11 | 1.56E-11 | 1.55E-11 |
| 530 | 2.66E-13 | 3.35E-13 | 1.20E-11 | 1.54E-11 | 1.54E-11 |
| 540 | 2.83E-13 | 3.56E-13 | 1.18E-11 | 1.53E-11 | 1.53E-11 |
| 550 | 3.00E-13 | 3.77E-13 | 1.16E-11 | 1.52E-11 | 1.51E-11 |
| 560 | 3.18E-13 | 3.99E-13 | 1.15E-11 | 1.51E-11 | 1.50E-11 |
| 570 | 3.37E-13 | 4.22E-13 | 1.13E-11 | 1.50E-11 | 1.50E-11 |
| 580 | 3.56E-13 | 4.45E-13 | 1.12E-11 | 1.49E-11 | 1.49E-11 |
| 590 | 3.77E-13 | 4.69E-13 | 1.11E-11 | 1.49E-11 | 1.48E-11 |
| 600 | 3.97E-13 | 4.94E-13 | 1.10E-11 | 1.48E-11 | 1.48E-11 |
| 610 | 4.19E-13 | 5.20E-13 | 1.09E-11 | 1.48E-11 | 1.47E-11 |
| 620 | 4.41E-13 | 5.47E-13 | 1.08E-11 | 1.47E-11 | 1.47E-11 |
| 630 | 4.65E-13 | 5.75E-13 | 1.07E-11 | 1.47E-11 | 1.47E-11 |
| 637 | 4.81E-13 | 5.94E-13 | 1.07E-11 | 1.47E-11 | 1.47E-11 |
| 640 | 4.88E-13 | 6.03E-13 | 1.07E-11 | 1.47E-11 | 1.47E-11 |
| 650 | 5.13E-13 | 6.32E-13 | 1.06E-11 | 1.47E-11 | 1.47E-11 |
| 660 | 5.39E-13 | 6.62E-13 | 1.05E-11 | 1.47E-11 | 1.47E-11 |
| 670 | 5.65E-13 | 6.93E-13 | 1.05E-11 | 1.47E-11 | 1.47E-11 |
| 680 | 5.92E-13 | 7.25E-13 | 1.05E-11 | 1.48E-11 | 1.47E-11 |
| 690 | 6.20E-13 | 7.58E-13 | 1.04E-11 | 1.48E-11 | 1.48E-11 |
| 700 | 6.48E-13 | 7.91E-13 | 1.04E-11 | 1.48E-11 | 1.48E-11 |
| 800 | 9.84E-13 | 1.18E-12 | 1.05E-11 | 1.55E-11 | 1.54E-11 |
| 900 | 1.41E-12 | 1.67E-12 | 1.09E-11 | 1.65E-11 | 1.65E-11 |
| 1000 | 1.91E-12 | 2.22E-12 | 1.16E-11 | 1.79E-11 | 1.78E-11 |
| 1100 | 2.49E-12 | 2.86E-12 | 1.24E-11 | 1.95E-11 | 1.95E-11 |
| 1200 | 3.17E-12 | 3.60E-12 | 1.35E-11 | 2.13E-11 | 2.12E-11 |
| 1300 | 3.97E-12 | 4.47E-12 | 1.46E-11 | 2.34E-11 | 2.31E-11 |
| 1400 | 4.88E-12 | 5.46E-12 | 1.59E-11 | 2.57E-11 | 2.52E-11 |
| 1500 | 5.93E-12 | 6.58E-12 | 1.74E-11 | 2.81E-11 | 2.75E-11 |
| 1600 | 7.11E-12 | 7.84E-12 | 1.90E-11 | 3.08E-11 | 3.00E-11 |
| 1700 | 8.44E-12 | 9.26E-12 | 2.07E-11 | 3.37E-11 | 3.28E-11 |
| 1800 | 9.92E-12 | 1.08E-11 | 2.25E-11 | 3.67E-11 | 3.59E-11 |
| 1900 | 1.16E-11 | 1.26E-11 | 2.42E-11 | 4.00E-11 | 3.91E-11 |
| 2000 | 1.34E-11 | 1.45E-11 | 2.59E-11 | 4.35E-11 | 4.26E-11 |
| 2100 | 1.53E-11 | 1.65E-11 | 2.76E-11 | 4.71E-11 | 4.63E-11 |
| 2200 | 1.75E-11 | 1.88E-11 | 2.95E-11 | 5.10E-11 | 5.03E-11 |
| 2300 | 1.98E-11 | 2.12E-11 | 3.14E-11 | 5.51E-11 | 5.45E-11 |
| 2400 | 2.24E-11 | 2.39E-11 | 3.34E-11 | 5.94E-11 | 5.90E-11 |
| 2500 | 2.51E-11 | 2.67E-11 | 3.55E-11 | 6.39E-11 | 6.37E-11 |
| 2600 | 2.80E-11 | 2.97E-11 | 3.77E-11 | 6.86E-11 | 6.85E-11 |
| 2700 | 3.11E-11 | 3.30E-11 | 3.99E-11 | 7.35E-11 | 7.34E-11 |
| 2800 | 3.44E-11 | 3.64E-11 | 4.23E-11 | 7.86E-11 | 7.86E-11 |
| 2900 | 3.80E-11 | 4.01E-11 | 4.47E-11 | 8.40E-11 | 8.39E-11 |
| 3000 | 4.18E-11 | 4.40E-11 | 4.72E-11 | 8.95E-11 | 8.95E-11 |

**Table S22**. High pressure limit rate constants calculated by VTST theory at the MP2/aTZ, M06-2X/maTZ, CCSD(T)/6-31++G(2df,pd)//MP2/aTZ, and CCSD(T)-full/6-311++G(df,2p)// MP2/aTZ levels for path 1.

|  | CCSD(T)-full/6-311++G(2df,2p)//MP2/aTZ | CCSD(T)-full/6-311++G(3df,2p)//MP2/aTZ | CCSD(T)/TZ// MP2/aTZ | CCSD(T)/aTZ//MP2/aTZ |
| --- | --- | --- | --- | --- |
| T/K | k | k | k | k |
| 230 | 4.39E-11 | 6.69E-11 | 5.39E-11 | 1.41E-10 |
| 232 | 4.28E-11 | 6.49E-11 | 5.24E-11 | 1.36E-10 |
| 240 | 3.86E-11 | 5.79E-11 | 4.70E-11 | 1.18E-10 |
| 250 | 3.44E-11 | 5.07E-11 | 4.16E-11 | 1.01E-10 |
| 260 | 3.10E-11 | 4.51E-11 | 3.72E-11 | 8.70E-11 |
| 270 | 2.83E-11 | 4.05E-11 | 3.37E-11 | 7.63E-11 |
| 280 | 2.59E-11 | 3.67E-11 | 3.07E-11 | 6.76E-11 |
| 290 | 2.40E-11 | 3.36E-11 | 2.83E-11 | 6.06E-11 |
| 298 | 2.27E-11 | 3.14E-11 | 2.66E-11 | 5.59E-11 |
| 298.15 | 2.27E-11 | 3.14E-11 | 2.66E-11 | 5.58E-11 |
| 300 | 2.24E-11 | 3.09E-11 | 2.62E-11 | 5.48E-11 |
| 310 | 2.10E-11 | 2.87E-11 | 2.45E-11 | 5.00E-11 |
| 320 | 1.98E-11 | 2.69E-11 | 2.30E-11 | 4.59E-11 |
| 330 | 1.88E-11 | 2.52E-11 | 2.17E-11 | 4.25E-11 |
| 340 | 1.79E-11 | 2.39E-11 | 2.06E-11 | 3.95E-11 |
| 350 | 1.72E-11 | 2.27E-11 | 1.97E-11 | 3.70E-11 |
| 360 | 1.65E-11 | 2.16E-11 | 1.88E-11 | 3.48E-11 |
| 374 | 1.57E-11 | 2.04E-11 | 1.78E-11 | 3.22E-11 |
| 380 | 1.54E-11 | 1.99E-11 | 1.75E-11 | 3.13E-11 |
| 390 | 1.50E-11 | 1.92E-11 | 1.69E-11 | 2.98E-11 |
| 400 | 1.46E-11 | 1.86E-11 | 1.64E-11 | 2.85E-11 |
| 410 | 1.42E-11 | 1.80E-11 | 1.60E-11 | 2.74E-11 |
| 424 | 1.38E-11 | 1.73E-11 | 1.54E-11 | 2.60E-11 |
| 430 | 1.36E-11 | 1.71E-11 | 1.52E-11 | 2.55E-11 |
| 440 | 1.34E-11 | 1.67E-11 | 1.49E-11 | 2.47E-11 |
| 450 | 1.32E-11 | 1.63E-11 | 1.46E-11 | 2.40E-11 |
| 460 | 1.30E-11 | 1.60E-11 | 1.44E-11 | 2.33E-11 |
| 470 | 1.28E-11 | 1.58E-11 | 1.42E-11 | 2.27E-11 |
| 480 | 1.27E-11 | 1.55E-11 | 1.40E-11 | 2.22E-11 |
| 490 | 1.26E-11 | 1.53E-11 | 1.38E-11 | 2.18E-11 |
| 500 | 1.24E-11 | 1.51E-11 | 1.37E-11 | 2.13E-11 |
| 510 | 1.24E-11 | 1.49E-11 | 1.36E-11 | 2.10E-11 |
| 520 | 1.23E-11 | 1.48E-11 | 1.34E-11 | 2.06E-11 |
| 530 | 1.22E-11 | 1.47E-11 | 1.34E-11 | 2.03E-11 |
| 540 | 1.22E-11 | 1.45E-11 | 1.33E-11 | 2.00E-11 |
| 550 | 1.21E-11 | 1.45E-11 | 1.32E-11 | 1.98E-11 |
| 560 | 1.21E-11 | 1.44E-11 | 1.31E-11 | 1.96E-11 |
| 570 | 1.21E-11 | 1.43E-11 | 1.31E-11 | 1.94E-11 |
| 580 | 1.20E-11 | 1.42E-11 | 1.31E-11 | 1.92E-11 |
| 590 | 1.20E-11 | 1.42E-11 | 1.30E-11 | 1.90E-11 |
| 600 | 1.20E-11 | 1.42E-11 | 1.30E-11 | 1.89E-11 |
| 610 | 1.21E-11 | 1.41E-11 | 1.30E-11 | 1.88E-11 |
| 620 | 1.21E-11 | 1.41E-11 | 1.30E-11 | 1.87E-11 |
| 630 | 1.21E-11 | 1.41E-11 | 1.30E-11 | 1.86E-11 |
| 637 | 1.21E-11 | 1.41E-11 | 1.31E-11 | 1.85E-11 |
| 640 | 1.21E-11 | 1.41E-11 | 1.31E-11 | 1.85E-11 |
| 650 | 1.22E-11 | 1.41E-11 | 1.31E-11 | 1.84E-11 |
| 660 | 1.22E-11 | 1.41E-11 | 1.31E-11 | 1.84E-11 |
| 670 | 1.22E-11 | 1.42E-11 | 1.31E-11 | 1.83E-11 |
| 680 | 1.23E-11 | 1.42E-11 | 1.32E-11 | 1.83E-11 |
| 690 | 1.24E-11 | 1.42E-11 | 1.32E-11 | 1.83E-11 |
| 700 | 1.24E-11 | 1.43E-11 | 1.33E-11 | 1.83E-11 |
| 800 | 1.32E-11 | 1.49E-11 | 1.40E-11 | 1.86E-11 |
| 900 | 1.41E-11 | 1.59E-11 | 1.51E-11 | 1.94E-11 |
| 1000 | 1.51E-11 | 1.69E-11 | 1.61E-11 | 2.07E-11 |
| 1100 | 1.64E-11 | 1.82E-11 | 1.74E-11 | 2.22E-11 |
| 1200 | 1.80E-11 | 1.97E-11 | 1.90E-11 | 2.41E-11 |
| 1300 | 1.98E-11 | 2.15E-11 | 2.08E-11 | 2.62E-11 |
| 1400 | 2.18E-11 | 2.36E-11 | 2.28E-11 | 2.85E-11 |
| 1500 | 2.40E-11 | 2.59E-11 | 2.51E-11 | 3.10E-11 |
| 1600 | 2.65E-11 | 2.84E-11 | 2.76E-11 | 3.37E-11 |
| 1700 | 2.92E-11 | 3.12E-11 | 3.03E-11 | 3.67E-11 |
| 1800 | 3.21E-11 | 3.41E-11 | 3.33E-11 | 3.98E-11 |
| 1900 | 3.52E-11 | 3.73E-11 | 3.64E-11 | 4.32E-11 |
| 2000 | 3.85E-11 | 4.08E-11 | 3.98E-11 | 4.68E-11 |
| 2100 | 4.21E-11 | 4.44E-11 | 4.34E-11 | 5.05E-11 |
| 2200 | 4.59E-11 | 4.83E-11 | 4.73E-11 | 5.45E-11 |
| 2300 | 5.00E-11 | 5.24E-11 | 5.14E-11 | 5.87E-11 |
| 2400 | 5.42E-11 | 5.68E-11 | 5.57E-11 | 6.31E-11 |
| 2500 | 5.88E-11 | 6.14E-11 | 6.03E-11 | 6.77E-11 |
| 2600 | 6.35E-11 | 6.63E-11 | 6.51E-11 | 7.25E-11 |
| 2700 | 6.85E-11 | 7.14E-11 | 7.02E-11 | 7.76E-11 |
| 2800 | 7.38E-11 | 7.68E-11 | 7.55E-11 | 8.28E-11 |
| 2900 | 7.93E-11 | 8.24E-11 | 8.11E-11 | 8.83E-11 |
| 3000 | 8.51E-11 | 8.84E-11 | 8.70E-11 | 9.40E-11 |

**Table S23**. High pressure limit rate constants calculated by VTST theory at the CCSD(T)-full/6-311++G(2df,2p)//MP2/aTZ, CCSD(T)-full/6-311++G(3df,2p)//MP2/aTZ, CCSD(T)/TZ// MP2/aTZ, and CCSD(T)/aTZ//MP2/aTZ levels for path 1.

|  | MP2/aTZ | | M06-2X/maTZ | | CCSD(T)/6-31++G(2df, pd)//MP2/aTZ |
| --- | --- | --- | --- | --- | --- |
| T/K | k^Eckart^ | k^Shavit^ | k^Eckart^ | k^Shavit^ | k |
| 230 | 1.54E-14 | 9.74E-15 | 3.58E-13 | 3.69E-11 | 2.89E-11 |
| 232 | 1.57E-14 | 1.02E-14 | 3.61E-13 | 3.68E-11 | 2.80E-11 |
| 240 | 1.72E-14 | 1.20E-14 | 3.74E-13 | 3.65E-11 | 2.50E-11 |
| 250 | 1.93E-14 | 1.46E-14 | 3.92E-13 | 3.61E-11 | 2.20E-11 |
| 260 | 2.15E-14 | 1.74E-14 | 4.09E-13 | 3.59E-11 | 1.95E-11 |
| 270 | 2.38E-14 | 2.06E-14 | 4.28E-13 | 3.57E-11 | 1.75E-11 |
| 280 | 2.63E-14 | 2.40E-14 | 4.47E-13 | 3.56E-11 | 1.59E-11 |
| 290 | 2.89E-14 | 2.78E-14 | 4.67E-13 | 3.55E-11 | 1.46E-11 |
| 298 | 3.11E-14 | 3.10E-14 | 4.84E-13 | 3.55E-11 | 1.36E-11 |
| 298.15 | 3.12E-14 | 3.10E-14 | 4.84E-13 | 3.55E-11 | 1.36E-11 |
| 300 | 3.17E-14 | 3.18E-14 | 4.88E-13 | 3.54E-11 | 1.34E-11 |
| 310 | 3.47E-14 | 3.62E-14 | 5.09E-13 | 3.55E-11 | 1.25E-11 |
| 320 | 3.79E-14 | 4.08E-14 | 5.32E-13 | 3.55E-11 | 1.17E-11 |
| 330 | 4.12E-14 | 4.58E-14 | 5.55E-13 | 3.56E-11 | 1.10E-11 |
| 340 | 4.47E-14 | 5.11E-14 | 5.78E-13 | 3.57E-11 | 1.03E-11 |
| 350 | 4.84E-14 | 5.66E-14 | 6.03E-13 | 3.59E-11 | 9.82E-12 |
| 360 | 5.24E-14 | 6.25E-14 | 6.28E-13 | 3.60E-11 | 9.36E-12 |
| 374 | 5.82E-14 | 7.13E-14 | 6.65E-13 | 3.63E-11 | 8.81E-12 |
| 380 | 6.08E-14 | 7.52E-14 | 6.81E-13 | 3.65E-11 | 8.61E-12 |
| 390 | 6.53E-14 | 8.20E-14 | 7.09E-13 | 3.67E-11 | 8.29E-12 |
| 400 | 7.00E-14 | 8.91E-14 | 7.38E-13 | 3.70E-11 | 8.02E-12 |
| 410 | 7.50E-14 | 9.65E-14 | 7.67E-13 | 3.73E-11 | 7.77E-12 |
| 424 | 8.23E-14 | 1.07E-13 | 8.10E-13 | 3.77E-11 | 7.47E-12 |
| 430 | 8.56E-14 | 1.12E-13 | 8.29E-13 | 3.79E-11 | 7.35E-12 |
| 440 | 9.12E-14 | 1.21E-13 | 8.61E-13 | 3.82E-11 | 7.18E-12 |
| 450 | 9.71E-14 | 1.29E-13 | 8.94E-13 | 3.86E-11 | 7.02E-12 |
| 460 | 1.03E-13 | 1.38E-13 | 9.28E-13 | 3.90E-11 | 6.88E-12 |
| 470 | 1.09E-13 | 1.47E-13 | 9.62E-13 | 3.93E-11 | 6.75E-12 |
| 480 | 1.16E-13 | 1.57E-13 | 9.98E-13 | 3.97E-11 | 6.64E-12 |
| 490 | 1.23E-13 | 1.67E-13 | 1.03E-12 | 4.01E-11 | 6.54E-12 |
| 500 | 1.30E-13 | 1.77E-13 | 1.07E-12 | 4.05E-11 | 6.44E-12 |
| 510 | 1.37E-13 | 1.87E-13 | 1.11E-12 | 4.10E-11 | 6.36E-12 |
| 520 | 1.45E-13 | 1.98E-13 | 1.15E-12 | 4.14E-11 | 6.28E-12 |
| 530 | 1.53E-13 | 2.09E-13 | 1.19E-12 | 4.18E-11 | 6.21E-12 |
| 540 | 1.61E-13 | 2.20E-13 | 1.23E-12 | 4.23E-11 | 6.15E-12 |
| 550 | 1.69E-13 | 2.32E-13 | 1.27E-12 | 4.28E-11 | 6.09E-12 |
| 560 | 1.78E-13 | 2.44E-13 | 1.32E-12 | 4.32E-11 | 6.04E-12 |
| 570 | 1.86E-13 | 2.56E-13 | 1.36E-12 | 4.37E-11 | 5.98E-12 |
| 580 | 1.95E-13 | 2.69E-13 | 1.41E-12 | 4.42E-11 | 5.93E-12 |
| 590 | 2.05E-13 | 2.81E-13 | 1.45E-12 | 4.47E-11 | 5.87E-12 |
| 600 | 2.14E-13 | 2.94E-13 | 1.50E-12 | 4.52E-11 | 5.81E-12 |
| 610 | 2.23E-13 | 3.07E-13 | 1.55E-12 | 4.57E-11 | 5.75E-12 |
| 620 | 2.32E-13 | 3.19E-13 | 1.60E-12 | 4.63E-11 | 5.70E-12 |
| 630 | 2.41E-13 | 3.31E-13 | 1.65E-12 | 4.68E-11 | 5.65E-12 |
| 637 | 2.47E-13 | 3.39E-13 | 1.68E-12 | 4.72E-11 | 5.62E-12 |
| 640 | 2.50E-13 | 3.42E-13 | 1.70E-12 | 4.73E-11 | 5.61E-12 |
| 650 | 2.59E-13 | 3.55E-13 | 1.75E-12 | 4.79E-11 | 5.57E-12 |
| 660 | 2.68E-13 | 3.67E-13 | 1.80E-12 | 4.84E-11 | 5.54E-12 |
| 670 | 2.78E-13 | 3.80E-13 | 1.86E-12 | 4.90E-11 | 5.52E-12 |
| 680 | 2.88E-13 | 3.93E-13 | 1.91E-12 | 4.95E-11 | 5.49E-12 |
| 690 | 2.98E-13 | 4.06E-13 | 1.97E-12 | 5.01E-11 | 5.47E-12 |
| 700 | 3.08E-13 | 4.19E-13 | 2.03E-12 | 5.07E-11 | 5.46E-12 |
| 800 | 4.27E-13 | 5.70E-13 | 2.66E-12 | 5.68E-11 | 5.47E-12 |
| 900 | 5.75E-13 | 7.53E-13 | 3.41E-12 | 6.34E-11 | 5.69E-12 |
| 1000 | 7.55E-13 | 9.71E-13 | 4.29E-12 | 7.05E-11 | 6.06E-12 |
| 1100 | 9.72E-13 | 1.23E-12 | 5.29E-12 | 7.80E-11 | 6.56E-12 |
| 1200 | 1.23E-12 | 1.53E-12 | 6.43E-12 | 8.60E-11 | 7.15E-12 |
| 1300 | 1.53E-12 | 1.88E-12 | 7.72E-12 | 9.44E-11 | 7.84E-12 |
| 1400 | 1.87E-12 | 2.27E-12 | 9.16E-12 | 1.03E-10 | 8.62E-12 |
| 1500 | 2.27E-12 | 2.72E-12 | 1.07E-11 | 1.12E-10 | 9.49E-12 |
| 1600 | 2.71E-12 | 3.22E-12 | 1.25E-11 | 1.22E-10 | 1.04E-11 |
| 1700 | 3.22E-12 | 3.78E-12 | 1.44E-11 | 1.32E-10 | 1.15E-11 |
| 1800 | 3.78E-12 | 4.40E-12 | 1.65E-11 | 1.42E-10 | 1.26E-11 |
| 1900 | 4.40E-12 | 5.09E-12 | 1.88E-11 | 1.53E-10 | 1.38E-11 |
| 2000 | 5.08E-12 | 5.84E-12 | 2.12E-11 | 1.64E-10 | 1.51E-11 |
| 2100 | 5.83E-12 | 6.66E-12 | 2.39E-11 | 1.76E-10 | 1.65E-11 |
| 2200 | 6.64E-12 | 7.54E-12 | 2.67E-11 | 1.88E-10 | 1.80E-11 |
| 2300 | 7.53E-12 | 8.51E-12 | 2.97E-11 | 2.00E-10 | 1.96E-11 |
| 2400 | 8.49E-12 | 9.54E-12 | 3.29E-11 | 2.13E-10 | 2.12E-11 |
| 2500 | 9.52E-12 | 1.07E-11 | 3.63E-11 | 2.26E-10 | 2.30E-11 |
| 2600 | 1.06E-11 | 1.19E-11 | 4.00E-11 | 2.39E-10 | 2.49E-11 |
| 2700 | 1.18E-11 | 1.31E-11 | 4.38E-11 | 2.53E-10 | 2.68E-11 |
| 2800 | 1.31E-11 | 1.45E-11 | 4.79E-11 | 2.67E-10 | 2.89E-11 |
| 2900 | 1.44E-11 | 1.59E-11 | 5.21E-11 | 2.81E-10 | 3.10E-11 |
| 3000 | 1.59E-11 | 1.75E-11 | 5.66E-11 | 2.96E-10 | 3.33E-11 |

**Table S24**. High pressure limit rate constants calculated by VTST theory at the MP2/aTZ, M06-2X/maTZ, and CCSD(T)/6-31++G(2df, pd)//MP2/aTZ levels for path 2.

|  | CCSD(T)-full/6-311++G(df, 2p)//MP2/aTZ | CCSD(T)-full/6-311++G(2df,2p)//MP2/aTZ | CCSD(T)-full/6-311++G(3df,2p)//MP2/aTZ | CCSD(T)/TZ//MP2/aTZ | CCSD(T)/aTZ//MP2/aTZ |
| --- | --- | --- | --- | --- | --- |
| T/K | k | k | k | k | k |
| 230 | 2.65E-11 | 8.79E-12 | 1.26E-11 | 2.13E-11 | 3.32E-11 |
| 232 | 2.58E-11 | 8.62E-12 | 1.23E-11 | 2.08E-11 | 3.22E-11 |
| 240 | 2.30E-11 | 7.98E-12 | 1.12E-11 | 1.87E-11 | 2.86E-11 |
| 250 | 2.03E-11 | 7.33E-12 | 1.02E-11 | 1.66E-11 | 2.50E-11 |
| 260 | 1.81E-11 | 6.78E-12 | 9.31E-12 | 1.49E-11 | 2.21E-11 |
| 270 | 1.63E-11 | 6.32E-12 | 8.59E-12 | 1.35E-11 | 1.98E-11 |
| 280 | 1.48E-11 | 5.94E-12 | 7.98E-12 | 1.23E-11 | 1.79E-11 |
| 290 | 1.36E-11 | 5.61E-12 | 7.47E-12 | 1.13E-11 | 1.63E-11 |
| 298 | 1.28E-11 | 5.38E-12 | 7.12E-12 | 1.07E-11 | 1.52E-11 |
| 298.15 | 1.27E-11 | 5.38E-12 | 7.11E-12 | 1.07E-11 | 1.52E-11 |
| 300 | 1.26E-11 | 5.33E-12 | 7.03E-12 | 1.05E-11 | 1.50E-11 |
| 310 | 1.17E-11 | 5.09E-12 | 6.66E-12 | 9.84E-12 | 1.38E-11 |
| 320 | 1.09E-11 | 4.88E-12 | 6.34E-12 | 9.25E-12 | 1.29E-11 |
| 330 | 1.03E-11 | 4.70E-12 | 6.06E-12 | 8.73E-12 | 1.21E-11 |
| 340 | 9.75E-12 | 4.54E-12 | 5.82E-12 | 8.29E-12 | 1.14E-11 |
| 350 | 9.26E-12 | 4.41E-12 | 5.61E-12 | 7.90E-12 | 1.08E-11 |
| 360 | 8.84E-12 | 4.28E-12 | 5.42E-12 | 7.56E-12 | 1.03E-11 |
| 374 | 8.33E-12 | 4.14E-12 | 5.20E-12 | 7.15E-12 | 9.63E-12 |
| 380 | 8.14E-12 | 4.08E-12 | 5.11E-12 | 6.99E-12 | 9.39E-12 |
| 390 | 7.85E-12 | 4.00E-12 | 4.98E-12 | 6.75E-12 | 9.03E-12 |
| 400 | 7.60E-12 | 3.93E-12 | 4.87E-12 | 6.54E-12 | 8.71E-12 |
| 410 | 7.37E-12 | 3.86E-12 | 4.77E-12 | 6.35E-12 | 8.43E-12 |
| 424 | 7.09E-12 | 3.78E-12 | 4.65E-12 | 6.11E-12 | 8.08E-12 |
| 430 | 6.98E-12 | 3.75E-12 | 4.60E-12 | 6.02E-12 | 7.95E-12 |
| 440 | 6.81E-12 | 3.70E-12 | 4.53E-12 | 5.87E-12 | 7.75E-12 |
| 450 | 6.66E-12 | 3.65E-12 | 4.46E-12 | 5.73E-12 | 7.57E-12 |
| 460 | 6.53E-12 | 3.61E-12 | 4.41E-12 | 5.60E-12 | 7.41E-12 |
| 470 | 6.40E-12 | 3.57E-12 | 4.35E-12 | 5.46E-12 | 7.26E-12 |
| 480 | 6.29E-12 | 3.52E-12 | 4.30E-12 | 5.34E-12 | 7.13E-12 |
| 490 | 6.18E-12 | 3.47E-12 | 4.26E-12 | 5.22E-12 | 7.01E-12 |
| 500 | 6.07E-12 | 3.43E-12 | 4.22E-12 | 5.12E-12 | 6.91E-12 |
| 510 | 5.97E-12 | 3.39E-12 | 4.18E-12 | 5.02E-12 | 6.81E-12 |
| 520 | 5.85E-12 | 3.36E-12 | 4.13E-12 | 4.94E-12 | 6.73E-12 |
| 530 | 5.74E-12 | 3.34E-12 | 4.08E-12 | 4.87E-12 | 6.65E-12 |
| 540 | 5.65E-12 | 3.31E-12 | 4.04E-12 | 4.80E-12 | 6.58E-12 |
| 550 | 5.56E-12 | 3.30E-12 | 4.00E-12 | 4.74E-12 | 6.52E-12 |
| 560 | 5.48E-12 | 3.28E-12 | 3.97E-12 | 4.69E-12 | 6.46E-12 |
| 570 | 5.41E-12 | 3.27E-12 | 3.94E-12 | 4.64E-12 | 6.41E-12 |
| 580 | 5.35E-12 | 3.26E-12 | 3.92E-12 | 4.60E-12 | 6.37E-12 |
| 590 | 5.30E-12 | 3.25E-12 | 3.90E-12 | 4.56E-12 | 6.32E-12 |
| 600 | 5.25E-12 | 3.25E-12 | 3.88E-12 | 4.53E-12 | 6.28E-12 |
| 610 | 5.20E-12 | 3.24E-12 | 3.87E-12 | 4.50E-12 | 6.24E-12 |
| 620 | 5.16E-12 | 3.24E-12 | 3.85E-12 | 4.48E-12 | 6.20E-12 |
| 630 | 5.13E-12 | 3.25E-12 | 3.85E-12 | 4.46E-12 | 6.15E-12 |
| 637 | 5.11E-12 | 3.25E-12 | 3.84E-12 | 4.45E-12 | 6.11E-12 |
| 640 | 5.10E-12 | 3.25E-12 | 3.84E-12 | 4.44E-12 | 6.10E-12 |
| 650 | 5.07E-12 | 3.26E-12 | 3.84E-12 | 4.43E-12 | 6.05E-12 |
| 660 | 5.05E-12 | 3.27E-12 | 3.84E-12 | 4.42E-12 | 6.01E-12 |
| 670 | 5.04E-12 | 3.28E-12 | 3.84E-12 | 4.42E-12 | 5.97E-12 |
| 680 | 5.02E-12 | 3.29E-12 | 3.85E-12 | 4.41E-12 | 5.94E-12 |
| 690 | 5.01E-12 | 3.30E-12 | 3.85E-12 | 4.41E-12 | 5.91E-12 |
| 700 | 5.00E-12 | 3.32E-12 | 3.86E-12 | 4.41E-12 | 5.89E-12 |
| 800 | 5.07E-12 | 3.53E-12 | 4.04E-12 | 4.54E-12 | 5.84E-12 |
| 900 | 5.32E-12 | 3.86E-12 | 4.35E-12 | 4.82E-12 | 6.04E-12 |
| 1000 | 5.71E-12 | 4.28E-12 | 4.76E-12 | 5.23E-12 | 6.40E-12 |
| 1100 | 6.20E-12 | 4.77E-12 | 5.26E-12 | 5.73E-12 | 6.88E-12 |
| 1200 | 6.80E-12 | 5.35E-12 | 5.85E-12 | 6.32E-12 | 7.48E-12 |
| 1300 | 7.48E-12 | 6.00E-12 | 6.51E-12 | 6.99E-12 | 8.17E-12 |
| 1400 | 8.26E-12 | 6.72E-12 | 7.25E-12 | 7.75E-12 | 8.96E-12 |
| 1500 | 9.11E-12 | 7.52E-12 | 8.08E-12 | 8.59E-12 | 9.84E-12 |
| 1600 | 1.01E-11 | 8.40E-12 | 8.98E-12 | 9.52E-12 | 1.08E-11 |
| 1700 | 1.11E-11 | 9.35E-12 | 9.96E-12 | 1.05E-11 | 1.19E-11 |
| 1800 | 1.22E-11 | 1.04E-11 | 1.10E-11 | 1.16E-11 | 1.30E-11 |
| 1900 | 1.34E-11 | 1.15E-11 | 1.22E-11 | 1.28E-11 | 1.42E-11 |
| 2000 | 1.47E-11 | 1.27E-11 | 1.34E-11 | 1.40E-11 | 1.55E-11 |
| 2100 | 1.60E-11 | 1.40E-11 | 1.47E-11 | 1.54E-11 | 1.69E-11 |
| 2200 | 1.75E-11 | 1.54E-11 | 1.61E-11 | 1.68E-11 | 1.84E-11 |
| 2300 | 1.91E-11 | 1.68E-11 | 1.76E-11 | 1.83E-11 | 2.00E-11 |
| 2400 | 2.07E-11 | 1.84E-11 | 1.92E-11 | 2.00E-11 | 2.17E-11 |
| 2500 | 2.25E-11 | 2.00E-11 | 2.09E-11 | 2.17E-11 | 2.35E-11 |
| 2600 | 2.43E-11 | 2.17E-11 | 2.27E-11 | 2.35E-11 | 2.54E-11 |
| 2700 | 2.62E-11 | 2.36E-11 | 2.45E-11 | 2.54E-11 | 2.74E-11 |
| 2800 | 2.83E-11 | 2.55E-11 | 2.65E-11 | 2.74E-11 | 2.94E-11 |
| 2900 | 3.04E-11 | 2.75E-11 | 2.86E-11 | 2.95E-11 | 3.16E-11 |
| 3000 | 3.26E-11 | 2.97E-11 | 3.07E-11 | 3.17E-11 | 3.39E-11 |

**Table S25**. High pressure limit rate constants calculated by VTST theory at the CCSD(T)-full/6-311++G(df, 2p)//MP2/aTZ, CCSD(T)-full/6-311++G(2df, 2p)//MP2/aTZ, CCSD(T)-full/6-311 ++G(3df, 2p)//MP2/aTZ, CCSD(T)/TZ//MP2/aTZ , and CCSD(T)/aTZ//MP2/aTZ levels for path 2.

|  | MP2/aTZ | |  | M06-2X/maTZ | |  | CCSD(T)/aTZ//MP2/aTZ | |  |
| --- | --- | --- | --- | --- | --- | --- | --- | --- | --- |
| T/K | k^Eckart^ | k^Shavit^ | | k^Eckart^ | k^Shavit^ | | k^Eckart^ | k^Shavit^ | |
| 230 | 1.04E-36 | 5.08E-39 | | 7.70E-34 | 2.58E-34 | | 8.37E-30 | 1.35E-31 | |
| 232 | 1.41E-36 | 8.46E-39 | | 1.12E-33 | 3.90E-34 | | 1.04E-29 | 1.95E-31 | |
| 240 | 4.73E-36 | 5.99E-38 | | 4.77E-33 | 1.92E-33 | | 2.48E-29 | 7.84E-31 | |
| 250 | 2.13E-35 | 5.81E-37 | | 2.63E-32 | 1.22E-32 | | 7.21E-29 | 3.95E-30 | |
| 260 | 9.38E-35 | 4.74E-36 | | 1.30E-31 | 6.73E-32 | | 2.06E-28 | 1.76E-29 | |
| 270 | 4.02E-34 | 3.31E-35 | | 5.76E-31 | 3.28E-31 | | 5.71E-28 | 7.01E-29 | |
| 280 | 1.65E-33 | 2.01E-34 | | 2.33E-30 | 1.43E-30 | | 1.54E-27 | 2.54E-28 | |
| 290 | 6.50E-33 | 1.08E-33 | | 8.65E-30 | 5.66E-30 | | 3.99E-27 | 8.41E-28 | |
| 298 | 1.87E-32 | 3.84E-33 | | 2.33E-29 | 1.59E-29 | | 8.33E-27 | 2.07E-27 | |
| 298.15 | 1.91E-32 | 3.93E-33 | | 2.37E-29 | 1.62E-29 | | 8.45E-27 | 2.11E-27 | |
| 300 | 2.43E-32 | 5.21E-33 | | 2.95E-29 | 2.04E-29 | | 9.98E-27 | 2.58E-27 | |
| 310 | 8.60E-32 | 2.27E-32 | | 9.46E-29 | 6.81E-29 | | 2.41E-26 | 7.35E-27 | |
| 320 | 2.88E-31 | 9.03E-32 | | 2.83E-28 | 2.11E-28 | | 5.59E-26 | 1.97E-26 | |
| 330 | 9.17E-31 | 3.31E-31 | | 7.90E-28 | 6.11E-28 | | 1.25E-25 | 4.97E-26 | |
| 340 | 2.76E-30 | 1.12E-30 | | 2.09E-27 | 1.67E-27 | | 2.70E-25 | 1.19E-25 | |
| 350 | 7.92E-30 | 3.56E-30 | | 5.28E-27 | 4.30E-27 | | 5.64E-25 | 2.71E-25 | |
| 360 | 2.16E-29 | 1.06E-29 | | 1.27E-26 | 1.05E-26 | | 1.14E-24 | 5.90E-25 | |
| 374 | 8.19E-29 | 4.44E-29 | | 4.00E-26 | 3.42E-26 | | 2.90E-24 | 1.64E-24 | |
| 380 | 1.41E-28 | 7.95E-29 | | 6.42E-26 | 5.52E-26 | | 4.25E-24 | 2.49E-24 | |
| 390 | 3.39E-28 | 2.02E-28 | | 1.36E-25 | 1.19E-25 | | 7.86E-24 | 4.85E-24 | |
| 400 | 7.82E-28 | 4.89E-28 | | 2.79E-25 | 2.47E-25 | | 1.42E-23 | 9.13E-24 | |
| 410 | 1.74E-27 | 1.14E-27 | | 5.54E-25 | 4.94E-25 | | 2.49E-23 | 1.67E-23 | |
| 424 | 5.05E-27 | 3.47E-27 | | 1.37E-24 | 1.24E-24 | | 5.28E-23 | 3.72E-23 | |
| 430 | 7.82E-27 | 5.48E-27 | | 1.99E-24 | 1.81E-24 | | 7.20E-23 | 5.16E-23 | |
| 440 | 1.58E-26 | 1.14E-26 | | 3.62E-24 | 3.32E-24 | | 1.19E-22 | 8.73E-23 | |
| 450 | 3.11E-26 | 2.31E-26 | | 6.43E-24 | 5.94E-24 | | 1.91E-22 | 1.45E-22 | |
| 460 | 5.95E-26 | 4.53E-26 | | 1.12E-23 | 1.04E-23 | | 3.03E-22 | 2.34E-22 | |
| 470 | 1.11E-25 | 8.63E-26 | | 1.89E-23 | 1.77E-23 | | 4.73E-22 | 3.73E-22 | |
| 480 | 2.02E-25 | 1.60E-25 | | 3.14E-23 | 2.96E-23 | | 7.25E-22 | 5.82E-22 | |
| 490 | 3.60E-25 | 2.91E-25 | | 5.13E-23 | 4.85E-23 | | 1.09E-21 | 8.92E-22 | |
| 500 | 6.28E-25 | 5.15E-25 | | 8.21E-23 | 7.80E-23 | | 1.63E-21 | 1.35E-21 | |
| 510 | 1.07E-24 | 8.94E-25 | | 1.29E-22 | 1.23E-22 | | 2.38E-21 | 2.00E-21 | |
| 520 | 1.80E-24 | 1.52E-24 | | 2.01E-22 | 1.91E-22 | | 3.45E-21 | 2.93E-21 | |
| 530 | 2.97E-24 | 2.53E-24 | | 3.05E-22 | 2.93E-22 | | 4.93E-21 | 4.24E-21 | |
| 540 | 4.81E-24 | 4.15E-24 | | 4.59E-22 | 4.41E-22 | | 6.97E-21 | 6.05E-21 | |
| 550 | 7.66E-24 | 6.67E-24 | | 6.80E-22 | 6.56E-22 | | 9.73E-21 | 8.53E-21 | |
| 560 | 1.20E-23 | 1.06E-23 | | 9.94E-22 | 9.62E-22 | | 1.34E-20 | 1.19E-20 | |
| 570 | 1.86E-23 | 1.65E-23 | | 1.43E-21 | 1.39E-21 | | 1.84E-20 | 1.64E-20 | |
| 580 | 2.83E-23 | 2.53E-23 | | 2.06E-21 | 1.99E-21 | | 2.49E-20 | 2.24E-20 | |
| 590 | 4.26E-23 | 3.84E-23 | | 2.90E-21 | 2.82E-21 | | 3.34E-20 | 3.02E-20 | |
| 600 | 6.34E-23 | 5.74E-23 | | 4.05E-21 | 3.95E-21 | | 4.44E-20 | 4.04E-20 | |
| 610 | 9.30E-23 | 8.48E-23 | | 5.60E-21 | 5.46E-21 | | 5.86E-20 | 5.36E-20 | |
| 620 | 1.35E-22 | 1.24E-22 | | 7.67E-21 | 7.50E-21 | | 7.67E-20 | 7.06E-20 | |
| 630 | 1.94E-22 | 1.79E-22 | | 1.04E-20 | 1.02E-20 | | 9.96E-20 | 9.21E-20 | |
| 637 | 2.48E-22 | 2.29E-22 | | 1.28E-20 | 1.26E-20 | | 1.19E-19 | 1.10E-19 | |
| 640 | 2.75E-22 | 2.55E-22 | | 1.40E-20 | 1.37E-20 | | 1.28E-19 | 1.19E-19 | |
| 650 | 3.87E-22 | 3.60E-22 | | 1.86E-20 | 1.83E-20 | | 1.64E-19 | 1.53E-19 | |
| 660 | 5.39E-22 | 5.04E-22 | | 2.47E-20 | 2.43E-20 | | 2.09E-19 | 1.96E-19 | |
| 670 | 7.44E-22 | 6.98E-22 | | 3.24E-20 | 3.19E-20 | | 2.64E-19 | 2.48E-19 | |
| 680 | 1.02E-21 | 9.58E-22 | | 4.22E-20 | 4.16E-20 | | 3.31E-19 | 3.12E-19 | |
| 690 | 1.38E-21 | 1.30E-21 | | 5.47E-20 | 5.39E-20 | | 4.13E-19 | 3.91E-19 | |
| 700 | 1.86E-21 | 1.76E-21 | | 7.03E-20 | 6.93E-20 | | 5.12E-19 | 4.86E-19 | |
| 800 | 2.48E-20 | 2.40E-20 | | 6.30E-19 | 6.24E-19 | | 3.39E-18 | 3.28E-18 | |
| 900 | 1.94E-19 | 1.90E-19 | | 3.60E-18 | 3.58E-18 | | 1.53E-17 | 1.50E-17 | |
| 1000 | 1.04E-18 | 1.02E-18 | | 1.50E-17 | 1.49E-17 | | 3.66E-17 | 3.61E-17 | |
| 1100 | 2.99E-18 | 2.96E-18 | | 4.92E-17 | 4.91E-17 | | 5.06E-17 | 5.01E-17 | |
| 1200 | 4.96E-18 | 4.93E-18 | | 1.36E-16 | 1.35E-16 | | 6.64E-17 | 6.60E-17 | |
| 1300 | 7.65E-18 | 7.61E-18 | | 3.25E-16 | 3.24E-16 | | 8.38E-17 | 8.35E-17 | |
| 1400 | 1.11E-17 | 1.11E-17 | | 6.95E-16 | 6.95E-16 | | 1.03E-16 | 1.02E-16 | |
| 1500 | 1.54E-17 | 1.53E-17 | | 1.36E-15 | 1.36E-15 | | 1.22E-16 | 1.22E-16 | |
| 1600 | 2.04E-17 | 2.04E-17 | | 2.48E-15 | 2.48E-15 | | 1.43E-16 | 1.43E-16 | |
| 1700 | 2.63E-17 | 2.63E-17 | | 4.26E-15 | 4.26E-15 | | 1.64E-16 | 1.64E-16 | |
| 1800 | 3.30E-17 | 3.29E-17 | | 6.94E-15 | 6.94E-15 | | 1.86E-16 | 1.86E-16 | |
| 1900 | 4.04E-17 | 4.03E-17 | | 1.09E-14 | 1.08E-14 | | 2.08E-16 | 2.08E-16 | |
| 2000 | 4.85E-17 | 4.85E-17 | | 1.63E-14 | 1.62E-14 | | 2.30E-16 | 2.30E-16 | |
| 2100 | 5.73E-17 | 5.73E-17 | | 2.36E-14 | 2.36E-14 | | 2.53E-16 | 2.52E-16 | |
| 2200 | 6.67E-17 | 6.67E-17 | | 3.33E-14 | 3.33E-14 | | 2.75E-16 | 2.75E-16 | |
| 2300 | 7.67E-17 | 7.67E-17 | | 4.59E-14 | 4.59E-14 | | 2.97E-16 | 2.97E-16 | |
| 2400 | 8.73E-17 | 8.73E-17 | | 6.18E-14 | 6.18E-14 | | 3.20E-16 | 3.19E-16 | |
| 2500 | 9.83E-17 | 9.83E-17 | | 8.17E-14 | 8.16E-14 | | 3.42E-16 | 3.42E-16 | |
| 2600 | 1.10E-16 | 1.10E-16 | | 1.06E-13 | 1.06E-13 | | 3.64E-16 | 3.64E-16 | |
| 2700 | 1.22E-16 | 1.22E-16 | | 1.36E-13 | 1.35E-13 | | 3.85E-16 | 3.85E-16 | |
| 2800 | 1.34E-16 | 1.34E-16 | | 1.71E-13 | 1.71E-13 | | 4.07E-16 | 4.07E-16 | |
| 2900 | 1.46E-16 | 1.46E-16 | | 2.12E-13 | 2.12E-13 | | 4.28E-16 | 4.28E-16 | |
| 3000 | 1.59E-16 | 1.59E-16 | | 2.61E-13 | 2.61E-13 | | 4.49E-16 | 4.49E-16 | |

**Table S26**. High pressure limit rate constants calculated by VTST theory at the MP2/aTZ, M06-2X/maTZ, and CCSD(T)/aTZ//MP2/aTZ levels for path 3.

|  | MP2/aTZ | |  | M06-2X/maTZ | |  | CCSD(T)/aTZ//MP2/aTZ | |  |
| --- | --- | --- | --- | --- | --- | --- | --- | --- | --- |
| T/K | k^Eckart^ | k^Shavit^ | | k^Eckart^ | k^Shavit^ | | k^Eckart^ | k^Shavit^ | |
| 230 | 4.79E-27 | 1.62E-66 | | 9.13E-53 | 4.54E-68 | | 1.70E-26 | 1.17E-65 | |
| 232 | 4.87E-27 | 4.72E-66 | | 1.18E-52 | 1.34E-67 | | 1.74E-26 | 3.41E-65 | |
| 240 | 5.21E-27 | 2.86E-64 | | 3.32E-52 | 8.55E-66 | | 1.89E-26 | 2.04E-63 | |
| 250 | 5.68E-27 | 3.35E-62 | | 1.21E-51 | 1.06E-63 | | 2.10E-26 | 2.34E-61 | |
| 260 | 6.22E-27 | 2.71E-60 | | 4.40E-51 | 9.02E-62 | | 2.33E-26 | 1.87E-59 | |
| 270 | 6.82E-27 | 1.58E-58 | | 1.61E-50 | 5.53E-60 | | 2.60E-26 | 1.07E-57 | |
| 280 | 7.50E-27 | 6.88E-57 | | 5.89E-50 | 2.53E-58 | | 2.91E-26 | 4.62E-56 | |
| 290 | 8.27E-27 | 2.31E-55 | | 2.16E-49 | 8.87E-57 | | 3.25E-26 | 1.53E-54 | |
| 298 | 8.95E-27 | 3.24E-54 | | 6.11E-49 | 1.29E-55 | | 3.56E-26 | 2.13E-53 | |
| 298.15 | 8.96E-27 | 3.39E-54 | | 6.23E-49 | 1.35E-55 | | 3.56E-26 | 2.24E-53 | |
| 300 | 9.13E-27 | 6.12E-54 | | 7.93E-49 | 2.45E-55 | | 3.64E-26 | 4.03E-53 | |
| 310 | 1.01E-26 | 1.31E-52 | | 2.92E-48 | 5.48E-54 | | 4.08E-26 | 8.55E-52 | |
| 320 | 1.12E-26 | 2.32E-51 | | 1.07E-47 | 1.01E-52 | | 4.58E-26 | 1.50E-50 | |
| 330 | 1.25E-26 | 3.46E-50 | | 3.96E-47 | 1.56E-51 | | 5.15E-26 | 2.21E-49 | |
| 340 | 1.39E-26 | 4.38E-49 | | 1.46E-46 | 2.04E-50 | | 5.80E-26 | 2.78E-48 | |
| 350 | 1.55E-26 | 4.80E-48 | | 5.40E-46 | 2.32E-49 | | 6.54E-26 | 3.03E-47 | |
| 360 | 1.73E-26 | 4.61E-47 | | 1.99E-45 | 2.30E-48 | | 7.37E-26 | 2.88E-46 | |
| 374 | 2.02E-26 | 8.90E-46 | | 1.24E-44 | 4.64E-47 | | 8.74E-26 | 5.53E-45 | |
| 380 | 2.17E-26 | 2.96E-45 | | 2.71E-44 | 1.57E-46 | | 9.41E-26 | 1.83E-44 | |
| 390 | 2.43E-26 | 2.03E-44 | | 9.96E-44 | 1.11E-45 | | 1.06E-25 | 1.25E-43 | |
| 400 | 2.73E-26 | 1.26E-43 | | 3.63E-43 | 7.08E-45 | | 1.21E-25 | 7.69E-43 | |
| 410 | 3.07E-26 | 7.14E-43 | | 1.31E-42 | 4.14E-44 | | 1.37E-25 | 4.35E-42 | |
| 424 | 3.62E-26 | 7.08E-42 | | 7.74E-42 | 4.26E-43 | | 1.63E-25 | 4.28E-41 | |
| 430 | 3.89E-26 | 1.81E-41 | | 1.64E-41 | 1.11E-42 | | 1.76E-25 | 1.09E-40 | |
| 440 | 4.39E-26 | 8.14E-41 | | 5.61E-41 | 5.11E-42 | | 2.00E-25 | 4.89E-40 | |
| 450 | 4.96E-26 | 3.43E-40 | | 1.88E-40 | 2.21E-41 | | 2.27E-25 | 2.05E-39 | |
| 460 | 5.60E-26 | 1.36E-39 | | 6.12E-40 | 8.98E-41 | | 2.58E-25 | 8.11E-39 | |
| 470 | 6.34E-26 | 5.08E-39 | | 1.94E-39 | 3.44E-40 | | 2.94E-25 | 3.02E-38 | |
| 480 | 7.18E-26 | 1.80E-38 | | 5.95E-39 | 1.25E-39 | | 3.35E-25 | 1.06E-37 | |
| 490 | 8.13E-26 | 6.04E-38 | | 1.77E-38 | 4.29E-39 | | 3.81E-25 | 3.56E-37 | |
| 500 | 9.22E-26 | 1.93E-37 | | 5.12E-38 | 1.40E-38 | | 4.35E-25 | 1.14E-36 | |
| 510 | 1.05E-25 | 5.91E-37 | | 1.43E-37 | 4.40E-38 | | 4.96E-25 | 3.47E-36 | |
| 520 | 1.19E-25 | 1.73E-36 | | 3.90E-37 | 1.32E-37 | | 5.65E-25 | 1.01E-35 | |
| 530 | 1.35E-25 | 4.88E-36 | | 1.03E-36 | 3.79E-37 | | 6.45E-25 | 2.84E-35 | |
| 540 | 1.53E-25 | 1.32E-35 | | 2.64E-36 | 1.05E-36 | | 7.37E-25 | 7.69E-35 | |
| 550 | 1.74E-25 | 3.45E-35 | | 6.57E-36 | 2.80E-36 | | 8.42E-25 | 2.00E-34 | |
| 560 | 1.99E-25 | 8.73E-35 | | 1.59E-35 | 7.24E-36 | | 9.62E-25 | 5.05E-34 | |
| 570 | 2.26E-25 | 2.14E-34 | | 3.76E-35 | 1.81E-35 | | 1.10E-24 | 1.23E-33 | |
| 580 | 2.58E-25 | 5.07E-34 | | 8.65E-35 | 4.37E-35 | | 1.26E-24 | 2.92E-33 | |
| 590 | 2.93E-25 | 1.17E-33 | | 1.94E-34 | 1.03E-34 | | 1.44E-24 | 6.71E-33 | |
| 600 | 3.35E-25 | 2.62E-33 | | 4.26E-34 | 2.35E-34 | | 1.65E-24 | 1.50E-32 | |
| 610 | 3.82E-25 | 5.72E-33 | | 9.13E-34 | 5.24E-34 | | 1.88E-24 | 3.26E-32 | |
| 620 | 4.35E-25 | 1.22E-32 | | 1.91E-33 | 1.14E-33 | | 2.12E-24 | 6.84E-32 | |
| 630 | 4.97E-25 | 2.53E-32 | | 3.93E-33 | 2.41E-33 | | 2.39E-24 | 1.40E-31 | |
| 637 | 5.45E-25 | 4.17E-32 | | 6.42E-33 | 4.02E-33 | | 2.60E-24 | 2.28E-31 | |
| 640 | 5.68E-25 | 5.15E-32 | | 7.90E-33 | 4.99E-33 | | 2.70E-24 | 2.80E-31 | |
| 650 | 6.48E-25 | 1.03E-31 | | 1.56E-32 | 1.01E-32 | | 3.05E-24 | 5.48E-31 | |
| 660 | 7.41E-25 | 2.00E-31 | | 3.02E-32 | 2.01E-32 | | 3.44E-24 | 1.05E-30 | |
| 670 | 8.47E-25 | 3.82E-31 | | 5.73E-32 | 3.91E-32 | | 3.89E-24 | 1.98E-30 | |
| 680 | 9.68E-25 | 7.17E-31 | | 1.07E-31 | 7.47E-32 | | 4.40E-24 | 3.66E-30 | |
| 690 | 1.11E-24 | 1.32E-30 | | 1.97E-31 | 1.40E-31 | | 4.97E-24 | 6.64E-30 | |
| 700 | 1.27E-24 | 2.40E-30 | | 3.56E-31 | 2.58E-31 | | 5.62E-24 | 1.18E-29 | |
| 800 | 4.90E-24 | 4.08E-28 | | 6.26E-29 | 5.16E-29 | | 1.97E-23 | 1.76E-27 | |
| 900 | 1.92E-23 | 2.27E-26 | | 3.75E-27 | 3.31E-27 | | 7.06E-23 | 8.78E-26 | |
| 1000 | 7.54E-23 | 5.77E-25 | | 1.04E-25 | 9.58E-26 | | 2.58E-22 | 2.04E-24 | |
| 1100 | 2.91E-22 | 8.30E-24 | | 1.64E-24 | 1.55E-24 | | 9.35E-22 | 2.73E-23 | |
| 1200 | 1.09E-21 | 7.78E-23 | | 1.68E-23 | 1.61E-23 | | 3.30E-21 | 2.40E-22 | |
| 1300 | 3.83E-21 | 5.25E-22 | | 1.23E-22 | 1.19E-22 | | 1.11E-20 | 1.54E-21 | |
| 1400 | 1.25E-20 | 2.73E-21 | | 6.92E-22 | 6.77E-22 | | 3.49E-20 | 7.66E-21 | |
| 1500 | 3.80E-20 | 1.16E-20 | | 3.14E-21 | 3.09E-21 | | 1.02E-19 | 3.12E-20 | |
| 1600 | 1.06E-19 | 4.13E-20 | | 1.20E-20 | 1.18E-20 | | 2.76E-19 | 1.08E-19 | |
| 1700 | 2.74E-19 | 1.28E-19 | | 3.95E-20 | 3.91E-20 | | 6.91E-19 | 3.25E-19 | |
| 1800 | 6.56E-19 | 3.54E-19 | | 1.15E-19 | 1.15E-19 | | 1.62E-18 | 8.74E-19 | |
| 1900 | 1.47E-18 | 8.86E-19 | | 3.04E-19 | 3.02E-19 | | 3.54E-18 | 2.14E-18 | |
| 2000 | 3.11E-18 | 2.04E-18 | | 7.33E-19 | 7.29E-19 | | 7.33E-18 | 4.81E-18 | |
| 2100 | 6.21E-18 | 4.36E-18 | | 1.64E-18 | 1.63E-18 | | 1.44E-17 | 1.01E-17 | |
| 2200 | 1.18E-17 | 8.76E-18 | | 3.42E-18 | 3.40E-18 | | 2.69E-17 | 2.00E-17 | |
| 2300 | 2.15E-17 | 1.67E-17 | | 6.73E-18 | 6.71E-18 | | 4.81E-17 | 3.74E-17 | |
| 2400 | 3.75E-17 | 3.02E-17 | | 1.26E-17 | 1.26E-17 | | 8.29E-17 | 6.68E-17 | |
| 2500 | 6.31E-17 | 5.24E-17 | | 2.26E-17 | 2.25E-17 | | 1.38E-16 | 1.14E-16 | |
| 2600 | 1.03E-16 | 8.76E-17 | | 3.88E-17 | 3.87E-17 | | 2.22E-16 | 1.89E-16 | |
| 2700 | 1.63E-16 | 1.42E-16 | | 6.44E-17 | 6.42E-17 | | 3.48E-16 | 3.02E-16 | |
| 2800 | 2.51E-16 | 2.22E-16 | | 1.03E-16 | 1.03E-16 | | 5.30E-16 | 4.69E-16 | |
| 2900 | 3.77E-16 | 3.38E-16 | | 1.61E-16 | 1.61E-16 | | 7.89E-16 | 7.09E-16 | |
| 3000 | 5.53E-16 | 5.03E-16 | | 2.45E-16 | 2.44E-16 | | 1.15E-15 | 1.05E-15 | |

**Table S27**. High pressure limit rate constants calculated by VTST theory at the MP2/aTZ, M06-2X/maTZ, and CCSD(T)/aTZ//MP2/aTZ levels for path 4.

|  | MP2/aTZ | |  | M06-2X/maTZ | |  | CCSD(T)/aTZ//MP2/aTZ | |  |
| --- | --- | --- | --- | --- | --- | --- | --- | --- | --- |
| T/K | k^Eckart^ | k^Shavit^ | | k^Eckart^ | k^Shavit^ | | k^Eckart^ | k^Shavit^ | |
| 230 | 1.61E-52 | 1.32E-58 | | 3.51E-58 | 3.19E-60 | | 1.76E-56 | 6.38E-60 | |
| 232 | 2.96E-52 | 3.25E-58 | | 7.02E-58 | 7.30E-60 | | 3.53E-56 | 1.50E-59 | |
| 240 | 3.13E-51 | 1.03E-56 | | 1.03E-56 | 1.75E-58 | | 5.18E-55 | 3.98E-58 | |
| 250 | 5.07E-50 | 5.73E-55 | | 2.45E-55 | 7.12E-57 | | 1.22E-53 | 1.83E-56 | |
| 260 | 6.88E-49 | 2.38E-53 | | 4.97E-54 | 2.30E-55 | | 2.35E-52 | 6.46E-55 | |
| 270 | 7.96E-48 | 7.52E-52 | | 8.54E-53 | 5.83E-54 | | 3.80E-51 | 1.79E-53 | |
| 280 | 7.95E-47 | 1.85E-50 | | 1.29E-51 | 1.22E-52 | | 5.17E-50 | 3.98E-52 | |
| 290 | 6.94E-46 | 3.67E-49 | | 1.69E-50 | 2.14E-51 | | 6.09E-49 | 7.28E-51 | |
| 298 | 3.59E-45 | 3.45E-48 | | 1.21E-49 | 1.87E-50 | | 3.98E-48 | 6.53E-50 | |
| 298.15 | 3.7E-45 | 3.60E-48 | | 1.26E-49 | 1.95E-50 | | 4.12E-48 | 6.78E-50 | |
| 300 | 5.36E-45 | 5.95E-48 | | 1.96E-49 | 3.18E-50 | | 6.26E-48 | 1.11E-49 | |
| 310 | 3.71E-44 | 7.98E-47 | | 2.03E-48 | 4.04E-49 | | 5.69E-47 | 1.44E-48 | |
| 320 | 2.32E-43 | 9.03E-46 | | 1.88E-47 | 4.50E-48 | | 4.60E-46 | 1.60E-47 | |
| 330 | 1.32E-42 | 8.83E-45 | | 1.57E-46 | 4.38E-47 | | 3.35E-45 | 1.56E-46 | |
| 340 | 6.94E-42 | 7.51E-44 | | 1.19E-45 | 3.80E-46 | | 2.20E-44 | 1.33E-45 | |
| 350 | 3.38E-41 | 5.62E-43 | | 8.18E-45 | 2.93E-45 | | 1.33E-43 | 1.01E-44 | |
| 360 | 1.53E-40 | 3.76E-42 | | 5.19E-44 | 2.06E-44 | | 7.31E-43 | 6.90E-44 | |
| 374 | 1.13E-39 | 4.50E-41 | | 5.92E-43 | 2.65E-43 | | 6.99E-42 | 8.60E-43 | |
| 380 | 2.59E-39 | 1.23E-40 | | 1.62E-42 | 7.57E-43 | | 1.77E-41 | 2.41E-42 | |
| 390 | 9.77E-39 | 6.11E-40 | | 8.03E-42 | 4.05E-42 | | 7.82E-41 | 1.25E-41 | |
| 400 | 3.5E-38 | 2.81E-39 | | 3.73E-41 | 1.99E-41 | | 3.24E-40 | 5.93E-41 | |
| 410 | 1.19E-37 | 1.19E-38 | | 1.62E-40 | 9.16E-41 | | 1.26E-39 | 2.64E-40 | |
| 424 | 6.12E-37 | 8.08E-38 | | 1.15E-39 | 6.92E-40 | | 7.73E-39 | 1.90E-39 | |
| 430 | 1.21E-36 | 1.76E-37 | | 2.56E-39 | 1.58E-39 | | 1.63E-38 | 4.26E-39 | |
| 440 | 3.58E-36 | 6.13E-37 | | 9.33E-39 | 6.01E-39 | | 5.41E-38 | 1.56E-38 | |
| 450 | 1.02E-35 | 2.02E-36 | | 3.23E-38 | 2.15E-38 | | 1.71E-37 | 5.41E-38 | |
| 460 | 2.81E-35 | 6.32E-36 | | 1.07E-37 | 7.34E-38 | | 5.20E-37 | 1.77E-37 | |
| 470 | 7.48E-35 | 1.88E-35 | | 3.36E-37 | 2.38E-37 | | 1.50E-36 | 5.58E-37 | |
| 480 | 1.92E-34 | 5.36E-35 | | 1.01E-36 | 7.37E-37 | | 4.21E-36 | 1.67E-36 | |
| 490 | 4.75E-34 | 1.46E-34 | | 2.92E-36 | 2.18E-36 | | 1.14E-35 | 4.77E-36 | |
| 500 | 1.13E-33 | 3.81E-34 | | 8.17E-36 | 6.19E-36 | | 2.94E-35 | 1.31E-35 | |
| 510 | 2.63E-33 | 9.58E-34 | | 2.19E-35 | 1.69E-35 | | 7.39E-35 | 3.47E-35 | |
| 520 | 6E-33 | 2.33E-33 | | 5.67E-35 | 4.47E-35 | | 1.80E-34 | 8.85E-35 | |
| 530 | 1.32E-32 | 5.48E-33 | | 1.42E-34 | 1.14E-34 | | 4.27E-34 | 2.20E-34 | |
| 540 | 2.83E-32 | 1.25E-32 | | 3.45E-34 | 2.80E-34 | | 9.73E-34 | 5.22E-34 | |
| 550 | 5.89E-32 | 2.75E-32 | | 8.11E-34 | 6.70E-34 | | 2.16E-33 | 1.21E-33 | |
| 560 | 1.21E-31 | 5.91E-32 | | 1.87E-33 | 1.56E-33 | | 4.76E-33 | 2.73E-33 | |
| 570 | 2.41E-31 | 1.23E-31 | | 4.16E-33 | 3.50E-33 | | 1.01E-32 | 5.99E-33 | |
| 580 | 4.7E-31 | 2.51E-31 | | 9.03E-33 | 7.69E-33 | | 2.08E-32 | 1.28E-32 | |
| 590 | 9.02E-31 | 4.98E-31 | | 1.91E-32 | 1.66E-32 | | 4.21E-32 | 2.66E-32 | |
| 600 | 1.69E-30 | 9.70E-31 | | 3.96E-32 | 3.44E-32 | | 8.29E-32 | 5.39E-32 | |
| 610 | 3.11E-30 | 1.85E-30 | | 8.04E-32 | 7.04E-32 | | 1.61E-31 | 1.07E-31 | |
| 620 | 5.65E-30 | 3.44E-30 | | 1.59E-31 | 1.40E-31 | | 3.08E-31 | 2.10E-31 | |
| 630 | 9.97E-30 | 6.28E-30 | | 3.09E-31 | 2.75E-31 | | 5.75E-31 | 3.99E-31 | |
| 637 | 1.48E-29 | 9.45E-30 | | 4.88E-31 | 4.34E-31 | | 8.81E-31 | 6.23E-31 | |
| 640 | 1.74E-29 | 1.12E-29 | | 5.89E-31 | 5.27E-31 | | 1.06E-30 | 7.52E-31 | |
| 650 | 3.01E-29 | 1.99E-29 | | 1.11E-30 | 9.97E-31 | | 1.91E-30 | 1.38E-30 | |
| 660 | 5.09E-29 | 3.44E-29 | | 2.02E-30 | 1.83E-30 | | 3.40E-30 | 2.49E-30 | |
| 670 | 8.49E-29 | 5.87E-29 | | 3.66E-30 | 3.34E-30 | | 5.94E-30 | 4.43E-30 | |
| 680 | 1.4E-28 | 9.84E-29 | | 6.51E-30 | 5.97E-30 | | 1.02E-29 | 7.76E-30 | |
| 690 | 2.27E-28 | 1.63E-28 | | 1.14E-29 | 1.05E-29 | | 1.75E-29 | 1.34E-29 | |
| 700 | 3.64E-28 | 2.66E-28 | | 1.97E-29 | 1.82E-29 | | 2.91E-29 | 2.27E-29 | |
| 800 | 2.24E-26 | 1.85E-26 | | 2.27E-27 | 2.16E-27 | | 2.65E-27 | 2.27E-27 | |
| 900 | 5.9E-25 | 5.21E-25 | | 9.60E-26 | 9.31E-26 | | 9.53E-26 | 8.62E-26 | |
| 1000 | 8.31E-24 | 7.61E-24 | | 2.02E-24 | 1.97E-24 | | 1.74E-24 | 1.63E-24 | |
| 1100 | 7.67E-23 | 7.23E-23 | | 2.50E-23 | 2.47E-23 | | 2.02E-23 | 1.92E-23 | |
| 1200 | 5.16E-22 | 4.92E-22 | | 2.10E-22 | 2.08E-22 | | 1.61E-22 | 1.55E-22 | |
| 1300 | 2.65E-21 | 2.57E-21 | | 1.30E-21 | 1.29E-21 | | 9.52E-22 | 9.26E-22 | |
| 1400 | 1.12E-20 | 1.09E-20 | | 6.30E-21 | 6.25E-21 | | 4.46E-21 | 4.37E-21 | |
| 1500 | 3.95E-20 | 3.87E-20 | | 2.52E-20 | 2.50E-20 | | 1.71E-20 | 1.69E-20 | |
| 1600 | 1.23E-19 | 1.21E-19 | | 8.56E-20 | 8.50E-20 | | 5.66E-20 | 5.59E-20 | |
| 1700 | 3.36E-19 | 3.32E-19 | | 2.54E-19 | 2.53E-19 | | 1.64E-19 | 1.62E-19 | |
| 1800 | 8.26E-19 | 8.15E-19 | | 6.77E-19 | 6.77E-19 | | 4.25E-19 | 4.21E-19 | |
| 1900 | 1.88E-18 | 1.86E-18 | | 1.64E-18 | 1.63E-18 | | 1.01E-18 | 9.98E-19 | |
| 2000 | 3.97E-18 | 3.93E-18 | | 3.67E-18 | 3.66E-18 | | 2.21E-18 | 2.19E-18 | |
| 2100 | 7.82E-18 | 7.75E-18 | | 7.63E-18 | 7.63E-18 | | 4.49E-18 | 4.46E-18 | |
| 2200 | 1.46E-17 | 1.46E-17 | | 1.50E-17 | 1.50E-17 | | 8.62E-18 | 8.57E-18 | |
| 2300 | 2.61E-17 | 2.60E-17 | | 2.79E-17 | 2.78E-17 | | 1.58E-17 | 1.57E-17 | |
| 2400 | 4.46E-17 | 4.44E-17 | | 4.96E-17 | 4.95E-17 | | 2.76E-17 | 2.74E-17 | |
| 2500 | 7.32E-17 | 7.29E-17 | | 8.43E-17 | 8.43E-17 | | 4.62E-17 | 4.60E-17 | |
| 2600 | 1.16E-16 | 1.16E-16 | | 1.38E-16 | 1.38E-16 | | 7.43E-17 | 7.43E-17 | |
| 2700 | 1.79E-16 | 1.78E-16 | | 2.18E-16 | 2.18E-16 | | 1.17E-16 | 1.16E-16 | |
| 2800 | 2.67E-16 | 2.66E-16 | | 3.36E-16 | 3.36E-16 | | 1.77E-16 | 1.77E-16 | |
| 2900 | 3.91E-16 | 3.90E-16 | | 5.02E-16 | 5.01E-16 | | 2.62E-16 | 2.62E-16 | |
| 3000 | 5.56E-16 | 5.53E-16 | | 7.34E-16 | 7.33E-16 | | 3.81E-16 | 3.79E-16 | |

**Table S28**. High pressure limit rate constants calculated by VTST theory at the MP2/aTZ, M06-2X/maTZ, and CCSD(T)/aTZ//MP2/aTZ levels for path 6.

|  | MP2/aTZ | | M06-2X/maTZ | | CCSD(T)/aTZ//MP2/aTZ | |
| --- | --- | --- | --- | --- | --- | --- |
| T/K | k^Eckart^ | k^Shavit^ | k^Eckart^ | k^Shavit^ | k^Eckart^ | k^Shavit^ |
| 230 | 4.79E-27 | 5.08E-39 | 7.7E-34 | 2.58E-34 | 1.7E-26 | 1.35E-31 |
| 232 | 4.87E-27 | 8.46E-39 | 1.12E-33 | 3.9E-34 | 1.74E-26 | 1.95E-31 |
| 240 | 5.21E-27 | 5.99E-38 | 4.77E-33 | 1.92E-33 | 1.89E-26 | 7.84E-31 |
| 250 | 5.68E-27 | 5.81E-37 | 2.63E-32 | 1.22E-32 | 2.11E-26 | 3.95E-30 |
| 260 | 6.22E-27 | 4.74E-36 | 1.3E-31 | 6.73E-32 | 2.35E-26 | 1.76E-29 |
| 270 | 6.82E-27 | 3.31E-35 | 5.76E-31 | 3.28E-31 | 2.66E-26 | 7.01E-29 |
| 280 | 7.5E-27 | 2.01E-34 | 2.33E-30 | 1.43E-30 | 3.06E-26 | 2.54E-28 |
| 290 | 8.27E-27 | 1.08E-33 | 8.65E-30 | 5.66E-30 | 3.65E-26 | 8.41E-28 |
| 298 | 8.95E-27 | 3.84E-33 | 2.33E-29 | 1.59E-29 | 4.39E-26 | 2.07E-27 |
| 298.15 | 8.96E-27 | 3.93E-33 | 2.37E-29 | 1.62E-29 | 4.41E-26 | 2.11E-27 |
| 300 | 9.13E-27 | 5.21E-33 | 2.95E-29 | 2.04E-29 | 4.64E-26 | 2.58E-27 |
| 310 | 1.01E-26 | 2.27E-32 | 9.46E-29 | 6.81E-29 | 6.49E-26 | 7.35E-27 |
| 320 | 1.12E-26 | 9.03E-32 | 2.83E-28 | 2.11E-28 | 1.02E-25 | 1.97E-26 |
| 330 | 1.25E-26 | 3.31E-31 | 7.9E-28 | 6.11E-28 | 1.77E-25 | 4.97E-26 |
| 340 | 1.39E-26 | 1.12E-30 | 2.09E-27 | 1.67E-27 | 3.28E-25 | 1.19E-25 |
| 350 | 1.55E-26 | 3.56E-30 | 5.28E-27 | 4.3E-27 | 6.29E-25 | 2.71E-25 |
| 360 | 1.73E-26 | 1.06E-29 | 1.27E-26 | 1.05E-26 | 1.21E-24 | 5.9E-25 |
| 374 | 2.03E-26 | 4.44E-29 | 4E-26 | 3.42E-26 | 2.99E-24 | 1.64E-24 |
| 380 | 2.18E-26 | 7.95E-29 | 6.42E-26 | 5.52E-26 | 4.34E-24 | 2.49E-24 |
| 390 | 2.46E-26 | 2.02E-28 | 1.36E-25 | 1.19E-25 | 7.97E-24 | 4.85E-24 |
| 400 | 2.81E-26 | 4.89E-28 | 2.79E-25 | 2.47E-25 | 1.43E-23 | 9.13E-24 |
| 410 | 3.24E-26 | 1.14E-27 | 5.54E-25 | 4.94E-25 | 2.5E-23 | 1.67E-23 |
| 424 | 4.13E-26 | 3.47E-27 | 1.37E-24 | 1.24E-24 | 5.3E-23 | 3.72E-23 |
| 430 | 4.67E-26 | 5.48E-27 | 1.99E-24 | 1.81E-24 | 7.22E-23 | 5.16E-23 |
| 440 | 5.97E-26 | 1.14E-26 | 3.62E-24 | 3.32E-24 | 1.19E-22 | 8.73E-23 |
| 450 | 8.07E-26 | 2.31E-26 | 6.43E-24 | 5.94E-24 | 1.91E-22 | 1.45E-22 |
| 460 | 1.16E-25 | 4.53E-26 | 1.12E-23 | 1.04E-23 | 3.03E-22 | 2.34E-22 |
| 470 | 1.74E-25 | 8.63E-26 | 1.89E-23 | 1.77E-23 | 4.73E-22 | 3.73E-22 |
| 480 | 2.74E-25 | 1.6E-25 | 3.14E-23 | 2.96E-23 | 7.25E-22 | 5.82E-22 |
| 490 | 4.41E-25 | 2.91E-25 | 5.13E-23 | 4.85E-23 | 1.09E-21 | 8.92E-22 |
| 500 | 7.2E-25 | 5.15E-25 | 8.21E-23 | 7.8E-23 | 1.63E-21 | 1.35E-21 |
| 510 | 1.18E-24 | 8.94E-25 | 1.29E-22 | 1.23E-22 | 2.38E-21 | 2E-21 |
| 520 | 1.92E-24 | 1.52E-24 | 2.01E-22 | 1.91E-22 | 3.45E-21 | 2.93E-21 |
| 530 | 3.11E-24 | 2.53E-24 | 3.05E-22 | 2.93E-22 | 4.93E-21 | 4.24E-21 |
| 540 | 4.96E-24 | 4.15E-24 | 4.59E-22 | 4.41E-22 | 6.97E-21 | 6.05E-21 |
| 550 | 7.83E-24 | 6.67E-24 | 6.8E-22 | 6.56E-22 | 9.73E-21 | 8.53E-21 |
| 560 | 1.22E-23 | 1.06E-23 | 9.94E-22 | 9.62E-22 | 1.34E-20 | 1.19E-20 |
| 570 | 1.88E-23 | 1.65E-23 | 1.43E-21 | 1.39E-21 | 1.84E-20 | 1.64E-20 |
| 580 | 2.86E-23 | 2.53E-23 | 2.06E-21 | 1.99E-21 | 2.49E-20 | 2.24E-20 |
| 590 | 4.29E-23 | 3.84E-23 | 2.9E-21 | 2.82E-21 | 3.34E-20 | 3.02E-20 |
| 600 | 6.37E-23 | 5.74E-23 | 4.05E-21 | 3.95E-21 | 4.44E-20 | 4.04E-20 |
| 610 | 9.34E-23 | 8.48E-23 | 5.6E-21 | 5.46E-21 | 5.86E-20 | 5.36E-20 |
| 620 | 1.35E-22 | 1.24E-22 | 7.67E-21 | 7.5E-21 | 7.67E-20 | 7.06E-20 |
| 630 | 1.94E-22 | 1.79E-22 | 1.04E-20 | 1.02E-20 | 9.96E-20 | 9.21E-20 |
| 637 | 2.49E-22 | 2.29E-22 | 1.28E-20 | 1.26E-20 | 1.19E-19 | 1.1E-19 |
| 640 | 2.76E-22 | 2.55E-22 | 1.4E-20 | 1.37E-20 | 1.28E-19 | 1.19E-19 |
| 650 | 3.88E-22 | 3.6E-22 | 1.86E-20 | 1.83E-20 | 1.64E-19 | 1.53E-19 |
| 660 | 5.4E-22 | 5.04E-22 | 2.47E-20 | 2.43E-20 | 2.09E-19 | 1.96E-19 |
| 670 | 7.45E-22 | 6.98E-22 | 3.24E-20 | 3.19E-20 | 2.64E-19 | 2.48E-19 |
| 680 | 1.02E-21 | 9.58E-22 | 4.22E-20 | 4.16E-20 | 3.31E-19 | 3.12E-19 |
| 690 | 1.38E-21 | 1.3E-21 | 5.47E-20 | 5.39E-20 | 4.13E-19 | 3.91E-19 |
| 700 | 1.86E-21 | 1.76E-21 | 7.03E-20 | 6.93E-20 | 5.12E-19 | 4.86E-19 |
| 800 | 2.48E-20 | 2.4E-20 | 6.3E-19 | 6.24E-19 | 3.39E-18 | 3.28E-18 |
| 900 | 1.94E-19 | 1.9E-19 | 3.6E-18 | 3.58E-18 | 1.53E-17 | 1.5E-17 |
| 1000 | 1.04E-18 | 1.02E-18 | 1.5E-17 | 1.49E-17 | 3.66E-17 | 3.61E-17 |
| 1100 | 2.99E-18 | 2.96E-18 | 4.92E-17 | 4.91E-17 | 5.06E-17 | 5.01E-17 |
| 1200 | 4.96E-18 | 4.93E-18 | 1.36E-16 | 1.35E-16 | 6.64E-17 | 6.6E-17 |
| 1300 | 7.66E-18 | 7.61E-18 | 3.25E-16 | 3.24E-16 | 8.38E-17 | 8.35E-17 |
| 1400 | 1.11E-17 | 1.11E-17 | 6.95E-16 | 6.95E-16 | 1.03E-16 | 1.02E-16 |
| 1500 | 1.55E-17 | 1.54E-17 | 1.36E-15 | 1.36E-15 | 1.22E-16 | 1.22E-16 |
| 1600 | 2.06E-17 | 2.06E-17 | 2.48E-15 | 2.48E-15 | 1.43E-16 | 1.43E-16 |
| 1700 | 2.69E-17 | 2.68E-17 | 4.26E-15 | 4.26E-15 | 1.65E-16 | 1.64E-16 |
| 1800 | 3.45E-17 | 3.41E-17 | 6.94E-15 | 6.94E-15 | 1.88E-16 | 1.87E-16 |
| 1900 | 4.38E-17 | 4.3E-17 | 1.09E-14 | 1.08E-14 | 2.13E-16 | 2.11E-16 |
| 2000 | 5.56E-17 | 5.45E-17 | 1.63E-14 | 1.62E-14 | 2.4E-16 | 2.37E-16 |
| 2100 | 7.13E-17 | 6.94E-17 | 2.36E-14 | 2.36E-14 | 2.72E-16 | 2.67E-16 |
| 2200 | 9.31E-17 | 9.01E-17 | 3.33E-14 | 3.33E-14 | 3.11E-16 | 3.04E-16 |
| 2300 | 1.24E-16 | 1.19E-16 | 4.59E-14 | 4.59E-14 | 3.61E-16 | 3.5E-16 |
| 2400 | 1.69E-16 | 1.62E-16 | 6.19E-14 | 6.19E-14 | 4.31E-16 | 4.13E-16 |
| 2500 | 2.35E-16 | 2.24E-16 | 8.18E-14 | 8.17E-14 | 5.26E-16 | 5.02E-16 |
| 2600 | 3.29E-16 | 3.14E-16 | 1.06E-13 | 1.06E-13 | 6.6E-16 | 6.27E-16 |
| 2700 | 4.64E-16 | 4.42E-16 | 1.36E-13 | 1.35E-13 | 8.5E-16 | 8.03E-16 |
| 2800 | 6.52E-16 | 6.22E-16 | 1.71E-13 | 1.71E-13 | 1.11E-15 | 1.05E-15 |
| 2900 | 9.14E-16 | 8.74E-16 | 2.13E-13 | 2.13E-13 | 1.48E-15 | 1.4E-15 |
| 3000 | 1.27E-15 | 1.22E-15 | 2.62E-13 | 2.62E-13 | 1.98E-15 | 1.88E-15 |

**Table S29**. High pressure limit rate constants calculated by VTST theory at the MP2/aTZ, M06-2X/maTZ, and CCSD(T)/aTZ//MP2/aTZ levels for path 3 + path 4 + path 6.

|  | MP2/aTZ | |  | M06-2X/maTZ | |  | CCSD(T)/aTZ//MP2/aTZ | |  |
| --- | --- | --- | --- | --- | --- | --- | --- | --- | --- |
| T/K | k^Eckart^ | k^Shavit^ | | k^Eckart^ | k^Shavit^ | | k^Eckart^ | k^Shavit^ | |
| 230 | 1.17E-46 | 9.64E-53 | | 1.20E-50 | 1.09E-52 | | 2.58E-50 | 9.36E-54 | |
| 232 | 1.92E-46 | 2.11E-52 | | 2.29E-50 | 2.38E-52 | | 4.93E-50 | 2.09E-53 | |
| 240 | 1.29E-45 | 4.23E-51 | | 2.75E-49 | 4.68E-51 | | 5.90E-49 | 4.53E-52 | |
| 250 | 1.21E-44 | 1.37E-49 | | 5.09E-48 | 1.48E-49 | | 1.07E-47 | 1.60E-50 | |
| 260 | 9.80E-44 | 3.39E-48 | | 7.80E-47 | 3.60E-48 | | 1.57E-46 | 4.31E-49 | |
| 270 | 7.00E-43 | 6.61E-47 | | 1.01E-45 | 6.90E-47 | | 1.93E-45 | 9.09E-48 | |
| 280 | 4.46E-42 | 1.04E-45 | | 1.13E-44 | 1.07E-45 | | 2.00E-44 | 1.54E-46 | |
| 290 | 2.57E-41 | 1.36E-44 | | 1.09E-43 | 1.38E-44 | | 1.80E-43 | 2.15E-45 | |
| 298 | 9.75E-41 | 9.38E-44 | | 6.11E-43 | 9.44E-44 | | 9.50E-43 | 1.56E-44 | |
| 298.15 | 1.00E-40 | 9.72E-44 | | 6.30E-43 | 9.77E-44 | | 9.79E-43 | 1.61E-44 | |
| 300 | 1.35E-40 | 1.50E-43 | | 9.28E-43 | 1.50E-43 | | 1.42E-42 | 2.51E-44 | |
| 310 | 6.55E-40 | 1.41E-42 | | 7.03E-42 | 1.40E-42 | | 9.94E-42 | 2.51E-43 | |
| 320 | 2.95E-39 | 1.15E-41 | | 4.77E-41 | 1.14E-41 | | 6.24E-41 | 2.17E-42 | |
| 330 | 1.24E-38 | 8.31E-41 | | 2.92E-40 | 8.14E-41 | | 3.55E-40 | 1.65E-41 | |
| 340 | 4.93E-38 | 5.34E-40 | | 1.63E-39 | 5.20E-40 | | 1.84E-39 | 1.11E-40 | |
| 350 | 1.85E-37 | 3.08E-39 | | 8.34E-39 | 2.99E-39 | | 8.81E-39 | 6.72E-40 | |
| 360 | 6.57E-37 | 1.62E-38 | | 3.94E-38 | 1.56E-38 | | 3.90E-38 | 3.68E-39 | |
| 374 | 3.58E-36 | 1.42E-37 | | 3.04E-37 | 1.36E-37 | | 2.78E-37 | 3.42E-38 | |
| 380 | 7.22E-36 | 3.43E-37 | | 7.02E-37 | 3.29E-37 | | 6.21E-37 | 8.46E-38 | |
| 390 | 2.24E-35 | 1.40E-36 | | 2.68E-36 | 1.35E-36 | | 2.26E-36 | 3.60E-37 | |
| 400 | 6.68E-35 | 5.36E-36 | | 9.62E-36 | 5.14E-36 | | 7.75E-36 | 1.42E-36 | |
| 410 | 1.92E-34 | 1.92E-35 | | 3.26E-35 | 1.84E-35 | | 2.52E-35 | 5.27E-36 | |
| 424 | 7.88E-34 | 1.04E-34 | | 1.65E-34 | 9.94E-35 | | 1.21E-34 | 2.98E-35 | |
| 430 | 1.42E-33 | 2.07E-34 | | 3.20E-34 | 1.98E-34 | | 2.31E-34 | 6.04E-35 | |
| 440 | 3.65E-33 | 6.25E-34 | | 9.33E-34 | 6.01E-34 | | 6.53E-34 | 1.88E-34 | |
| 450 | 9.13E-33 | 1.80E-33 | | 2.60E-33 | 1.73E-33 | | 1.77E-33 | 5.59E-34 | |
| 460 | 2.21E-32 | 4.97E-33 | | 6.97E-33 | 4.79E-33 | | 4.63E-33 | 1.58E-33 | |
| 470 | 5.21E-32 | 1.31E-32 | | 1.79E-32 | 1.27E-32 | | 1.16E-32 | 4.30E-33 | |
| 480 | 1.19E-31 | 3.33E-32 | | 4.44E-32 | 3.23E-32 | | 2.83E-32 | 1.12E-32 | |
| 490 | 2.65E-31 | 8.16E-32 | | 1.06E-31 | 7.91E-32 | | 6.67E-32 | 2.80E-32 | |
| 500 | 5.74E-31 | 1.93E-31 | | 2.47E-31 | 1.87E-31 | | 1.52E-31 | 6.77E-32 | |
| 510 | 1.21E-30 | 4.40E-31 | | 5.55E-31 | 4.29E-31 | | 3.37E-31 | 1.58E-31 | |
| 520 | 2.51E-30 | 9.75E-31 | | 1.21E-30 | 9.54E-31 | | 7.28E-31 | 3.58E-31 | |
| 530 | 5.05E-30 | 2.10E-30 | | 2.57E-30 | 2.06E-30 | | 1.53E-30 | 7.87E-31 | |
| 540 | 9.97E-30 | 4.39E-30 | | 5.32E-30 | 4.32E-30 | | 3.13E-30 | 1.68E-30 | |
| 550 | 1.92E-29 | 8.95E-30 | | 1.07E-29 | 8.84E-30 | | 6.26E-30 | 3.49E-30 | |
| 560 | 3.64E-29 | 1.78E-29 | | 2.12E-29 | 1.77E-29 | | 1.23E-29 | 7.07E-30 | |
| 570 | 6.76E-29 | 3.46E-29 | | 4.08E-29 | 3.44E-29 | | 2.35E-29 | 1.40E-29 | |
| 580 | 1.23E-28 | 6.57E-29 | | 7.70E-29 | 6.56E-29 | | 4.40E-29 | 2.71E-29 | |
| 590 | 2.21E-28 | 1.22E-28 | | 1.42E-28 | 1.23E-28 | | 8.09E-29 | 5.12E-29 | |
| 600 | 3.88E-28 | 2.23E-28 | | 2.58E-28 | 2.24E-28 | | 1.46E-28 | 9.49E-29 | |
| 610 | 6.72E-28 | 3.99E-28 | | 4.60E-28 | 4.03E-28 | | 2.59E-28 | 1.72E-28 | |
| 620 | 1.15E-27 | 7.00E-28 | | 8.05E-28 | 7.10E-28 | | 4.52E-28 | 3.08E-28 | |
| 630 | 1.92E-27 | 1.21E-27 | | 1.38E-27 | 1.23E-27 | | 7.76E-28 | 5.39E-28 | |
| 637 | 2.74E-27 | 1.75E-27 | | 2.01E-27 | 1.79E-27 | | 1.12E-27 | 7.91E-28 | |
| 640 | 3.18E-27 | 2.05E-27 | | 2.35E-27 | 2.10E-27 | | 1.31E-27 | 9.30E-28 | |
| 650 | 5.20E-27 | 3.43E-27 | | 3.91E-27 | 3.52E-27 | | 2.18E-27 | 1.58E-27 | |
| 660 | 8.37E-27 | 5.66E-27 | | 6.43E-27 | 5.82E-27 | | 3.59E-27 | 2.63E-27 | |
| 670 | 1.33E-26 | 9.19E-27 | | 1.04E-26 | 9.49E-27 | | 5.81E-27 | 4.33E-27 | |
| 680 | 2.09E-26 | 1.47E-26 | | 1.67E-26 | 1.53E-26 | | 9.28E-27 | 7.03E-27 | |
| 690 | 3.24E-26 | 2.33E-26 | | 2.63E-26 | 2.42E-26 | | 1.47E-26 | 1.13E-26 | |
| 700 | 4.98E-26 | 3.63E-26 | | 4.11E-26 | 3.80E-26 | | 2.29E-26 | 1.78E-26 | |
| 800 | 2.11E-24 | 1.74E-24 | | 1.98E-24 | 1.88E-24 | | 1.11E-24 | 9.48E-25 | |
| 900 | 4.16E-23 | 3.67E-23 | | 4.23E-23 | 4.10E-23 | | 2.41E-23 | 2.18E-23 | |
| 1000 | 4.75E-22 | 4.35E-22 | | 5.11E-22 | 5.00E-22 | | 2.97E-22 | 2.77E-22 | |
| 1100 | 3.60E-21 | 3.39E-21 | | 4.04E-21 | 3.98E-21 | | 2.40E-21 | 2.28E-21 | |
| 1200 | 2.01E-20 | 1.92E-20 | | 2.32E-20 | 2.30E-20 | | 1.41E-20 | 1.36E-20 | |
| 1300 | 8.78E-20 | 8.49E-20 | | 1.04E-19 | 1.03E-19 | | 6.42E-20 | 6.25E-20 | |
| 1400 | 3.17E-19 | 3.09E-19 | | 3.84E-19 | 3.81E-19 | | 2.41E-19 | 2.36E-19 | |
| 1500 | 9.80E-19 | 9.60E-19 | | 1.21E-18 | 1.20E-18 | | 7.69E-19 | 7.57E-19 | |
| 1600 | 2.67E-18 | 2.62E-18 | | 3.33E-18 | 3.31E-18 | | 2.15E-18 | 2.12E-18 | |
| 1700 | 6.52E-18 | 6.44E-18 | | 8.24E-18 | 8.21E-18 | | 5.37E-18 | 5.31E-18 | |
| 1800 | 1.46E-17 | 1.44E-17 | | 1.86E-17 | 1.86E-17 | | 1.22E-17 | 1.21E-17 | |
| 1900 | 3.03E-17 | 3.00E-17 | | 3.90E-17 | 3.88E-17 | | 2.57E-17 | 2.55E-17 | |
| 2000 | 5.89E-17 | 5.84E-17 | | 7.63E-17 | 7.61E-17 | | 5.07E-17 | 5.03E-17 | |
| 2100 | 1.08E-16 | 1.07E-16 | | 1.41E-16 | 1.41E-16 | | 9.41E-17 | 9.35E-17 | |
| 2200 | 1.89E-16 | 1.88E-16 | | 2.48E-16 | 2.48E-16 | | 1.66E-16 | 1.65E-16 | |
| 2300 | 3.17E-16 | 3.16E-16 | | 4.18E-16 | 4.17E-16 | | 2.81E-16 | 2.79E-16 | |
| 2400 | 5.12E-16 | 5.10E-16 | | 6.77E-16 | 6.76E-16 | | 4.57E-16 | 4.54E-16 | |
| 2500 | 7.99E-16 | 7.96E-16 | | 1.06E-15 | 1.06E-15 | | 7.17E-16 | 7.14E-16 | |
| 2600 | 1.21E-15 | 1.21E-15 | | 1.61E-15 | 1.61E-15 | | 1.09E-15 | 1.09E-15 | |
| 2700 | 1.79E-15 | 1.78E-15 | | 2.38E-15 | 2.38E-15 | | 1.62E-15 | 1.61E-15 | |
| 2800 | 2.57E-15 | 2.56E-15 | | 3.44E-15 | 3.44E-15 | | 2.34E-15 | 2.34E-15 | |
| 2900 | 3.62E-15 | 3.61E-15 | | 4.86E-15 | 4.85E-15 | | 3.31E-15 | 3.30E-15 | |
| 3000 | 5.00E-15 | 4.98E-15 | | 6.72E-15 | 6.71E-15 | | 4.60E-15 | 4.58E-15 | |

**Table S30**. High pressure limit rate constants calculated by VTST theory at the MP2/aTZ, M06-2X/maTZ, and CCSD(T)/aTZ//MP2/aTZ levels for path 5.

| T/K | P/bar | k (cm^3^molecule^−1^ s ^−1^) | T/K | P/bar | k (cm^3^molecule^−1^ s ^−1^) |
| --- | --- | --- | --- | --- | --- |
|  |  |  | 200 | 1.00$E$-07 | 7E-16 |
|  |  |  | 210 | 1.00$E$-07 | 3.65E-16 |
|  |  |  | 220 | 1.00$E$-07 | 2.02E-16 |
|  |  |  | 230 | 1.00$E$-07 | 1.17E-16 |
|  |  |  | 240 | 1.00$E$-07 | 7.16E-17 |
|  |  |  | 250 | 1.00$E$-07 | 3.96E-17 |
|  |  |  | 260 | 1.00$E$-07 | 2.59E-17 |
|  |  |  | 270 | 1.00$E$-07 | 1.75E-17 |
|  |  |  | 280 | 1.00$E$-07 | 1.22E-17 |
|  |  |  | 290 | 1.00$E$-07 | 8.67E-18 |
| 298 | 0.00$E$+00 | 7.87E-32 | 298 | 1.00$E$-07 | 6.73E-18 |
|  |  |  | 298.15 | 1.00$E$-07 | 6.7E-18 |
|  |  |  | 300 | 1.00$E$-07 | 6.33E-18 |
|  |  |  | 310 | 1.00$E$-07 | 4.72E-18 |
|  |  |  | 320 | 1.00$E$-07 | 3.59E-18 |
|  |  |  | 330 | 1.00$E$-07 | 2.77E-18 |
|  |  |  | 340 | 1.00$E$-07 | 2.18E-18 |
|  |  |  | 350 | 1.00$E$-07 | 1.74E-18 |
|  |  |  | 360 | 1.00$E$-07 | 1.4E-18 |
|  |  |  | 370 | 1.00$E$-07 | 7.65E-19 |
|  |  |  | 380 | 1.00$E$-07 | 6.33E-19 |
|  |  |  | 390 | 1.00$E$-07 | 5.29E-19 |
| 400 | 0.00$E$+00 | 2.8E-32 | 400 | 1.00$E$-07 | 4.47E-19 |
|  |  |  | 410 | 1.00$E$-07 | 3.81E-19 |
|  |  |  | 420 | 1.00$E$-07 | 3.27E-19 |
|  |  |  | 430 | 1.00$E$-07 | 2.83E-19 |
|  |  |  | 440 | 1.00$E$-07 | 2.47E-19 |
|  |  |  | 450 | 1.00$E$-07 | 2.17E-19 |
|  |  |  | 460 | 1.00$E$-07 | 1.91E-19 |
|  |  |  | 470 | 1.00$E$-07 | 1.7E-19 |
|  |  |  | 480 | 1.00$E$-07 | 1.52E-19 |
|  |  |  | 490 | 1.00$E$-07 | 1.36E-19 |
|  |  |  | 500 | 1.00$E$-07 | 1.19E-19 |
|  |  |  | 510 | 1.00$E$-07 | 1.08E-19 |
|  |  |  | 520 | 1.00$E$-07 | 9.84E-20 |
|  |  |  | 530 | 1.00$E$-07 | 9.01E-20 |
|  |  |  | 540 | 1.00$E$-07 | 8.29E-20 |
|  |  |  | 550 | 1.00$E$-07 | 7.65E-20 |
|  |  |  | 560 | 1.00$E$-07 | 7.08E-20 |
|  |  |  | 570 | 1.00$E$-07 | 6.58E-20 |
|  |  |  | 580 | 1.00$E$-07 | 6.12E-20 |
|  |  |  | 590 | 1.00$E$-07 | 5.72E-20 |
| 600 | 0.00$E$+00 | 1.09E-32 | 600 | 1.00$E$-07 | 5.36E-20 |
|  |  |  | 610 | 1.00$E$-07 | 5.03E-20 |
|  |  |  | 620 | 1.00$E$-07 | 5.4E-20 |
|  |  |  | 630 | 1.00$E$-07 | 5.13E-20 |
|  |  |  | 640 | 1.00$E$-07 | 4.88E-20 |
|  |  |  | 650 | 1.00$E$-07 | 4.65E-20 |
|  |  |  | 660 | 1.00$E$-07 | 4.45E-20 |
|  |  |  | 670 | 1.00$E$-07 | 4.26E-20 |
|  |  |  | 680 | 1.00$E$-07 | 4.08E-20 |
|  |  |  | 690 | 1.00$E$-07 | 3.92E-20 |
|  |  |  | 700 | 1.00$E$-07 | 3.77E-20 |
|  |  |  | 710 | 1.00$E$-07 | 3.63E-20 |
|  |  |  | 720 | 1.00$E$-07 | 3.51E-20 |
|  |  |  | 730 | 1.00$E$-07 | 3.39E-20 |
|  |  |  | 740 | 1.00$E$-07 | 2.98E-20 |
|  |  |  | 750 | 1.00$E$-07 | 2.89E-20 |
|  |  |  | 760 | 1.00$E$-07 | 2.81E-20 |
|  |  |  | 770 | 1.00$E$-07 | 2.73E-20 |
|  |  |  | 780 | 1.00$E$-07 | 2.65E-20 |
|  |  |  | 790 | 1.00$E$-07 | 2.59E-20 |
|  |  |  | 800 | 1.00$E$-07 | 2.52E-20 |
| T/K | P/bar | k (cm^3^molecule^−1^ s ^−1^) | T/K | P/bar | k (cm^3^molecule^−1^ s ^−1^) |
| 200 | 1.00$E$-06 | 3.6E-15 | 200 | 1.00$E$-05 | 1.79E-14 |
| 210 | 1.00$E$-06 | 1.93E-15 | 210 | 1.00$E$-05 | 9.97E-15 |
| 220 | 1.00$E$-06 | 1.1E-15 | 220 | 1.00$E$-05 | 5.86E-15 |
| 230 | 1.00$E$-06 | 6.58E-16 | 230 | 1.00$E$-05 | 3.61E-15 |
| 240 | 1.00$E$-06 | 4.11E-16 | 240 | 1.00$E$-05 | 2.31E-15 |
| 250 | 1.00$E$-06 | 2.46E-16 | 250 | 1.00$E$-05 | 1.46E-15 |
| 260 | 1.00$E$-06 | 1.64E-16 | 260 | 1.00$E$-05 | 9.99E-16 |
| 270 | 1.00$E$-06 | 1.13E-16 | 270 | 1.00$E$-05 | 7.02E-16 |
| 280 | 1.00$E$-06 | 7.99E-17 | 280 | 1.00$E$-05 | 5.06E-16 |
| 290 | 1.00$E$-06 | 5.8E-17 | 290 | 1.00$E$-05 | 3.74E-16 |
| 298 | 1.00$E$-06 | 4.55E-17 | 298 | 1.00$E$-05 | 2.98E-16 |
| 298.15 | 1.00$E$-06 | 4.53E-17 | 298.15 | 1.00$E$-05 | 2.96E-16 |
| 300 | 1.00$E$-06 | 4.3E-17 | 300 | 1.00$E$-05 | 2.82E-16 |
| 310 | 1.00$E$-06 | 3.25E-17 | 310 | 1.00$E$-05 | 2.16E-16 |
| 320 | 1.00$E$-06 | 2.5E-17 | 320 | 1.00$E$-05 | 1.69E-16 |
| 330 | 1.00$E$-06 | 1.96E-17 | 330 | 1.00$E$-05 | 1.34E-16 |
| 340 | 1.00$E$-06 | 1.56E-17 | 340 | 1.00$E$-05 | 1.08E-16 |
| 350 | 1.00$E$-06 | 1.26E-17 | 350 | 1.00$E$-05 | 8.81E-17 |
| 360 | 1.00$E$-06 | 1.03E-17 | 360 | 1.00$E$-05 | 7.27E-17 |
| 370 | 1.00$E$-06 | 6E-18 | 370 | 1.00$E$-05 | 4.63E-17 |
| 380 | 1.00$E$-06 | 5E-18 | 380 | 1.00$E$-05 | 3.89E-17 |
| 390 | 1.00$E$-06 | 4.2E-18 | 390 | 1.00$E$-05 | 3.3E-17 |
| 400 | 1.00$E$-06 | 3.57E-18 | 400 | 1.00$E$-05 | 2.83E-17 |
| 410 | 1.00$E$-06 | 3.06E-18 | 410 | 1.00$E$-05 | 2.44E-17 |
| 420 | 1.00$E$-06 | 2.64E-18 | 420 | 1.00$E$-05 | 2.12E-17 |
| 430 | 1.00$E$-06 | 2.3E-18 | 430 | 1.00$E$-05 | 1.86E-17 |
| 440 | 1.00$E$-06 | 2.02E-18 | 440 | 1.00$E$-05 | 1.64E-17 |
| 450 | 1.00$E$-06 | 1.78E-18 | 450 | 1.00$E$-05 | 1.45E-17 |
| 460 | 1.00E-06 | 1.58E-18 | 460 | 1.00$E$-05 | 1.3E-17 |
| 470 | 1.00$E$-06 | 1.41E-18 | 470 | 1.00$E$-05 | 1.16E-17 |
| 480 | 1.00$E$-06 | 1.26E-18 | 480 | 1.00$E$-05 | 1.05E-17 |
| 490 | 1.00$E$-06 | 1.14E-18 | 490 | 1.00$E$-05 | 9.5E-18 |
| 500 | 1.00$E$-06 | 8.46E-19 | 500 | 1.00$E$-05 | 6.9E-18 |
| 510 | 1.00$E$-06 | 7.71E-19 | 510 | 1.00$E$-05 | 6.31E-18 |
| 520 | 1.00$E$-06 | 7.05E-19 | 520 | 1.00$E$-05 | 5.79E-18 |
| 530 | 1.00$E$-06 | 6.48E-19 | 530 | 1.00$E$-05 | 5.34E-18 |
| 540 | 1.00$E$-06 | 5.97E-19 | 540 | 1.00$E$-05 | 4.94E-18 |
| 550 | 1.00$E$-06 | 5.52E-19 | 550 | 1.00$E$-05 | 4.58E-18 |
| 560 | 1.00$E$-06 | 5.12E-19 | 560 | 1.00$E$-05 | 4.27E-18 |
| 570 | 1.00$E$-06 | 4.77E-19 | 570 | 1.00$E$-05 | 3.98E-18 |
| 580 | 1.00$E$-06 | 4.45E-19 | 580 | 1.00$E$-05 | 3.73E-18 |
| 590 | 1.00$E$-06 | 4.17E-19 | 590 | 1.00$E$-05 | 3.5E-18 |
| 600 | 1.00$E$-06 | 3.91E-19 | 600 | 1.00$E$-05 | 3.29E-18 |
| 610 | 1.00$E$-06 | 3.68E-19 | 610 | 1.00$E$-05 | 3.11E-18 |
| 620 | 1.00$E$-06 | 4.41E-19 | 620 | 1.00$E$-05 | 3.51E-18 |
| 630 | 1.00$E$-06 | 4.2E-19 | 630 | 1.00$E$-05 | 3.35E-18 |
| 640 | 1.00$E$-06 | 4E-19 | 640 | 1.00$E$-05 | 3.2E-18 |
| 650 | 1.00$E$-06 | 3.83E-19 | 650 | 1.00$E$-05 | 3.07E-18 |
| 660 | 1.00$E$-06 | 3.66E-19 | 660 | 1.00$E$-05 | 2.94E-18 |
| 670 | 1.00$E$-06 | 3.52E-19 | 670 | 1.00$E$-05 | 2.83E-18 |
| 680 | 1.00$E$-06 | 3.38E-19 | 680 | 1.00$E$-05 | 2.72E-18 |
| 690 | 1.00$E$-06 | 3.25E-19 | 690 | 1.00$E$-05 | 2.62E-18 |
| 700 | 1.00$E$-06 | 3.13E-19 | 700 | 1.00$E$-05 | 2.53E-18 |
| 710 | 1.00$E$-06 | 3.02E-19 | 710 | 1.00$E$-05 | 2.44E-18 |
| 720 | 1.00$E$-06 | 2.92E-19 | 720 | 1.00$E$-05 | 2.36E-18 |
| 730 | 1.00$E$-06 | 2.83E-19 | 730 | 1.00$E$-05 | 2.29E-18 |
| 740 | 1.00$E$-06 | 2.54E-19 | 740 | 1.00$E$-05 | 2.14E-18 |
| 750 | 1.00$E$-06 | 2.47E-19 | 750 | 1.00$E$-05 | 2.09E-18 |
| 760 | 1.00$E$-06 | 2.4E-19 | 760 | 1.00$E$-05 | 2.03E-18 |
| 770 | 1.00$E$-06 | 2.34E-19 | 770 | 1.00$E$-05 | 1.99E-18 |
| 780 | 1.00$E$-06 | 2.28E-19 | 780 | 1.00$E$-05 | 1.94E-18 |
| 790 | 1.00$E$-06 | 2.23E-19 | 790 | 1.00$E$-05 | 1.9E-18 |
| 800 | 1.00$E$-06 | 2.18E-19 | 800 | 1.00$E$-05 | 1.86E-18 |
| T/K | P/bar | k (cm^3^molecule^−1^ s ^−1^) | T/K | P/bar | k (cm^3^molecule^−1^ s ^−1^) |
| 200 | 1.00$E$-04 | 8.54E-14 | 200 | 1.00$E$-03 | 3.94E-13 |
| 210 | 1.00$E$-04 | 4.97E-14 | 210 | 1.00$E$-03 | 2.38E-13 |
| 220 | 1.00$E$-04 | 3.03E-14 | 220 | 1.00$E$-03 | 1.52E-13 |
| 230 | 1.00$E$-04 | 1.93E-14 | 230 | 1.00$E$-03 | 1E-13 |
| 240 | 1.00$E$-04 | 1.27E-14 | 240 | 1.00$E$-03 | 6.8E-14 |
| 250 | 1.00$E$-04 | 8.41E-15 | 250 | 1.00$E$-03 | 4.69E-14 |
| 260 | 1.00$E$-04 | 5.89E-15 | 260 | 1.00$E$-03 | 3.38E-14 |
| 270 | 1.00$E$-04 | 4.24E-15 | 270 | 1.00$E$-03 | 2.49E-14 |
| 280 | 1.00$E$-04 | 3.12E-15 | 280 | 1.00$E$-03 | 1.87E-14 |
| 290 | 1.00$E$-04 | 2.35E-15 | 290 | 1.00$E$-03 | 1.44E-14 |
| 298 | 1.00$E$-04 | 1.89E-15 | 298 | 1.00$E$-03 | 1.18E-14 |
| 298.15 | 1.00$E$-04 | 1.89E-15 | 298.15 | 1.00$E$-03 | 1.18E-14 |
| 300 | 1.00$E$-04 | 1.8E-15 | 300 | 1.00$E$-03 | 1.12E-14 |
| 310 | 1.00$E$-04 | 1.4E-15 | 310 | 1.00$E$-03 | 8.93E-15 |
| 320 | 1.00$E$-04 | 1.11E-15 | 320 | 1.00$E$-03 | 7.19E-15 |
| 330 | 1.00$E$-04 | 8.95E-16 | 330 | 1.00$E$-03 | 5.88E-15 |
| 340 | 1.00$E$-04 | 7.3E-16 | 340 | 1.00$E$-03 | 4.86E-15 |
| 350 | 1.00$E$-04 | 6.03E-16 | 350 | 1.00$E$-03 | 4.05E-15 |
| 360 | 1.00$E$-04 | 5.03E-16 | 360 | 1.00$E$-03 | 3.42E-15 |
| 370 | 1.00$E$-04 | 3.49E-16 | 370 | 1.00$E$-03 | 2.54E-15 |
| 380 | 1.00$E$-04 | 2.96E-16 | 380 | 1.00$E$-03 | 2.18E-15 |
| 390 | 1.00$E$-04 | 2.53E-16 | 390 | 1.00$E$-03 | 1.89E-15 |
| 400 | 1.00$E$-04 | 2.19E-16 | 400 | 1.00$E$-03 | 1.65E-15 |
| 410 | 1.00$E$-04 | 1.9E-16 | 410 | 1.00$E$-03 | 1.45E-15 |
| 420 | 1.00$E$-04 | 1.67E-16 | 420 | 1.00$E$-03 | 1.28E-15 |
| 430 | 1.00$E$-04 | 1.47E-16 | 430 | 1.00$E$-03 | 1.14E-15 |
| 440 | 1.00$E$-04 | 1.31E-16 | 440 | 1.00$E$-03 | 1.02E-15 |
| 450 | 1.00$E$-04 | 1.17E-16 | 450 | 1.00$E$-03 | 9.15E-16 |
| 460 | 1.00$E$-04 | 1.05E-16 | 460 | 1.00$E$-03 | 8.29E-16 |
| 470 | 1.00$E$-04 | 9.47E-17 | 470 | 1.00$E$-03 | 7.54E-16 |
| 480 | 1.00$E$-04 | 8.59E-17 | 480 | 1.00$E$-03 | 6.89E-16 |
| 490 | 1.00$E$-04 | 7.83E-17 | 490 | 1.00$E$-03 | 6.31E-16 |
| 500 | 1.00$E$-04 | 5.8E-17 | 500 | 1.00$E$-03 | 4.8E-16 |
| 510 | 1.00$E$-04 | 5.32E-17 | 510 | 1.00$E$-03 | 4.43E-16 |
| 520 | 1.00$E$-04 | 4.9E-17 | 520 | 1.00$E$-03 | 4.1E-16 |
| 530 | 1.00$E$-04 | 4.54E-17 | 530 | 1.00$E$-03 | 3.81E-16 |
| 540 | 1.00$E$-04 | 4.21E-17 | 540 | 1.00$E$-03 | 3.55E-16 |
| 550 | 1.00$E$-04 | 3.92E-17 | 550 | 1.00$E$-03 | 3.32E-16 |
| 560 | 1.00$E$-04 | 3.66E-17 | 560 | 1.00$E$-03 | 3.11E-16 |
| 570 | 1.00$E$-04 | 3.43E-17 | 570 | 1.00$E$-03 | 2.92E-16 |
| 580 | 1.00$E$-04 | 3.22E-17 | 580 | 1.00$E$-03 | 2.76E-16 |
| 590 | 1.00$E$-04 | 3.03E-17 | 590 | 1.00$E$-03 | 2.61E-16 |
| 600 | 1.00$E$-04 | 2.86E-17 | 600 | 1.00$E$-03 | 2.47E-16 |
| 610 | 1.00$E$-04 | 2.71E-17 | 610 | 1.00$E$-03 | 2.34E-16 |
| 620 | 1.00$E$-04 | 2.64E-17 | 620 | 1.00$E$-03 | 1.87E-16 |
| 630 | 1.00$E$-04 | 2.52E-17 | 630 | 1.00$E$-03 | 1.79E-16 |
| 640 | 1.00$E$-04 | 2.41E-17 | 640 | 1.00$E$-03 | 1.71E-16 |
| 650 | 1.00$E$-04 | 2.31E-17 | 650 | 1.00$E$-03 | 1.64E-16 |
| 660 | 1.00$E$-04 | 2.22E-17 | 660 | 1.00$E$-03 | 1.58E-16 |
| 670 | 1.00$E$-04 | 2.13E-17 | 670 | 1.00$E$-03 | 1.52E-16 |
| 680 | 1.00$E$-04 | 2.05E-17 | 680 | 1.00$E$-03 | 1.46E-16 |
| 690 | 1.00$E$-04 | 1.98E-17 | 690 | 1.00$E$-03 | 1.41E-16 |
| 700 | 1.00$E$-04 | 1.91E-17 | 700 | 1.00$E$-03 | 1.36E-16 |
| 710 | 1.00$E$-04 | 1.85E-17 | 710 | 1.00$E$-03 | 1.32E-16 |
| 720 | 1.00$E$-04 | 1.79E-17 | 720 | 1.00$E$-03 | 1.28E-16 |
| 730 | 1.00$E$-04 | 1.73E-17 | 730 | 1.00$E$-03 | 1.24E-16 |
| 740 | 1.00$E$-04 | 1.79E-17 | 740 | 1.00$E$-03 | 1.48E-16 |
| 750 | 1.00$E$-04 | 1.75E-17 | 750 | 1.00$E$-03 | 1.44E-16 |
| 760 | 1.00$E$-04 | 1.71E-17 | 760 | 1.00$E$-03 | 1.41E-16 |
| 770 | 1.00$E$-04 | 1.67E-17 | 770 | 1.00$E$-03 | 1.38E-16 |
| 780 | 1.00$E$-04 | 1.63E-17 | 780 | 1.00$E$-03 | 1.35E-16 |
| 790 | 1.00$E$-04 | 1.6E-17 | 790 | 1.00$E$-03 | 1.32E-16 |
| 800 | 1.00$E$-04 | 1.57E-17 | 800 | 1.00$E$-03 | 1.3E-16 |
| T/K | P/bar | k (cm^3^molecule^−1^ s ^−1^ | T/K | P/bar | k (cm^3^molecule^−1^ s ^−1^ |
| 200 | 1.00$E$-02 | 1.74E-12 | 200 | 1.00$E$-01 | 7.51E-12 |
| 210 | 1.00$E$-02 | 1.11E-12 | 210 | 1.00$E$-01 | 4.96E-12 |
| 220 | 1.00$E$-02 | 7.33E-13 | 220 | 1.00$E$-01 | 3.43E-12 |
| 230 | 1.00$E$-02 | 5.02E-13 | 230 | 1.00$E$-01 | 2.44E-12 |
| 240 | 1.00$E$-02 | 3.54E-13 | 240 | 1.00$E$-01 | 1.78E-12 |
| 250 | 1.00$E$-02 | 2.54E-13 | 250 | 1.00$E$-01 | 1.33E-12 |
| 260 | 1.00$E$-02 | 1.88E-13 | 260 | 1.00$E$-01 | 1.02E-12 |
| 270 | 1.00$E$-02 | 1.43E-13 | 270 | 1.00$E$-01 | 7.92E-13 |
| 280 | 1.00$E$-02 | 1.1E-13 | 280 | 1.00$E$-01 | 6.26E-13 |
| 290 | 1.00$E$-02 | 8.63E-14 | 290 | 1.00$E$-01 | 5.04E-13 |
| 298 | 1.00$E$-02 | 7.2E-14 | 298 | 1.00$E$-01 | 4.27E-13 |
| 298.15 | 1.00$E$-02 | 7.17E-14 | 298.15 | 1.00$E$-01 | 4.27E-13 |
| 300 | 1.00$E$-02 | 6.88E-14 | 300 | 1.00$E$-01 | 4.11E-13 |
| 310 | 1.00$E$-02 | 5.57E-14 | 310 | 1.00$E$-01 | 3.39E-13 |
| 320 | 1.00$E$-02 | 4.56E-14 | 320 | 1.00$E$-01 | 2.84E-13 |
| 330 | 1.00$E$-02 | 3.79E-14 | 330 | 1.00$E$-01 | 2.4E-13 |
| 340 | 1.00$E$-02 | 3.18E-14 | 340 | 1.00$E$-01 | 2.04E-13 |
| 350 | 1.00$E$-02 | 2.69E-14 | 350 | 1.00$E$-01 | 1.75E-13 |
| 360 | 1.00$E$-02 | 2.3E-14 | 360 | 1.00$E$-01 | 1.52E-13 |
| 370 | 1.00$E$-02 | 1.8E-14 | 370 | 1.00$E$-01 | 1.24E-13 |
| 380 | 1.00$E$-02 | 1.57E-14 | 380 | 1.00$E$-01 | 1.09E-13 |
| 390 | 1.00$E$-02 | 1.37E-14 | 390 | 1.00$E$-01 | 9.67E-14 |
| 400 | 1.00$E$-02 | 1.21E-14 | 400 | 1.00$E$-01 | 8.61E-14 |
| 410 | 1.00$E$-02 | 1.07E-14 | 410 | 1.00$E$-01 | 7.71E-14 |
| 420 | 1.00$E$-02 | 9.54E-15 | 420 | 1.00$E$-01 | 6.95E-14 |
| 430 | 1.00$E$-02 | 8.57E-15 | 430 | 1.00$E$-01 | 6.29E-14 |
| 440 | 1.00$E$-02 | 7.73E-15 | 440 | 1.00$E$-01 | 5.72E-14 |
| 450 | 1.00$E$-02 | 7.01E-15 | 450 | 1.00$E$-01 | 5.23E-14 |
| 460 | 1.00$E$-02 | 6.39E-15 | 460 | 1.00$E$-01 | 4.81E-14 |
| 470 | 1.00$E$-02 | 5.85E-15 | 470 | 1.00$E$-01 | 4.44E-14 |
| 480 | 1.00$E$-02 | 5.38E-15 | 480 | 1.00$E$-01 | 4.11E-14 |
| 490 | 1.00$E$-02 | 4.97E-15 | 490 | 1.00$E$-01 | 3.81E-14 |
| 500 | 1.00$E$-02 | 3.94E-15 | 500 | 1.00$E$-01 | 3.16E-14 |
| 510 | 1.00$E$-02 | 3.65E-15 | 510 | 1.00$E$-01 | 2.95E-14 |
| 520 | 1.00$E$-02 | 3.4E-15 | 520 | 1.00$E$-01 | 2.76E-14 |
| 530 | 1.00$E$-02 | 3.18E-15 | 530 | 1.00$E$-01 | 2.59E-14 |
| 540 | 1.00$E$-02 | 2.98E-15 | 540 | 1.00$E$-01 | 2.44E-14 |
| 550 | 1.00$E$-02 | 2.8E-15 | 550 | 1.00$E$-01 | 2.31E-14 |
| 560 | 1.00$E$-02 | 2.63E-15 | 560 | 1.00$E$-01 | 2.19E-14 |
| 570 | 1.00$E$-02 | 2.49E-15 | 570 | 1.00$E$-01 | 2.07E-14 |
| 580 | 1.00$E$-02 | 2.35E-15 | 580 | 1.00$E$-01 | 1.97E-14 |
| 590 | 1.00$E$-02 | 2.23E-15 | 590 | 1.00$E$-01 | 1.88E-14 |
| 600 | 1.00$E$-02 | 2.12E-15 | 600 | 1.00$E$-01 | 1.8E-14 |
| 610 | 1.00$E$-02 | 2.02E-15 | 610 | 1.00$E$-01 | 1.72E-14 |
| 620 | 1.00$E$-02 | 1.49E-15 | 620 | 1.00$E$-01 | 1.27E-14 |
| 630 | 1.00$E$-02 | 1.43E-15 | 630 | 1.00$E$-01 | 1.22E-14 |
| 640 | 1.00$E$-02 | 1.37E-15 | 640 | 1.00$E$-01 | 1.17E-14 |
| 650 | 1.00$E$-02 | 1.32E-15 | 650 | 1.00$E$-01 | 1.13E-14 |
| 660 | 1.00$E$-02 | 1.27E-15 | 660 | 1.00$E$-01 | 1.09E-14 |
| 670 | 1.00$E$-02 | 1.22E-15 | 670 | 1.00$E$-01 | 1.06E-14 |
| 680 | 1.00$E$-02 | 1.18E-15 | 680 | 1.00$E$-01 | 1.02E-14 |
| 690 | 1.00$E$-02 | 1.14E-15 | 690 | 1.00$E$-01 | 9.9E-15 |
| 700 | 1.00$E$-02 | 1.11E-15 | 700 | 1.00$E$-01 | 9.61E-15 |
| 710 | 1.00$E$-02 | 1.07E-15 | 710 | 1.00$E$-01 | 9.34E-15 |
| 720 | 1.00$E$-02 | 1.04E-15 | 720 | 1.00$E$-01 | 9.09E-15 |
| 730 | 1.00$E$-02 | 1.01E-15 | 730 | 1.00$E$-01 | 8.83E-15 |
| 740 | 1.00$E$-02 | 1.18E-15 | 740 | 1.00$E$-01 | 8.65E-15 |
| 750 | 1.00$E$-02 | 1.15E-15 | 750 | 1.00$E$-01 | 8.46E-15 |
| 760 | 1.00$E$-02 | 1.13E-15 | 760 | 1.00$E$-01 | 8.29E-15 |
| 770 | 1.00$E$-02 | 1.1E-15 | 770 | 1.00$E$-01 | 8.1E-15 |
| 780 | 1.00$E$-02 | 1.08E-15 | 780 | 1.00$E$-01 | 7.97E-15 |
| 790 | 1.00$E$-02 | 1.06E-15 | 790 | 1.00$E$-01 | 7.78E-15 |
| 800 | 1.00$E$-02 | 1.04E-15 | 800 | 1.00$E$-01 | 7.66E-15 |
| T/K | P/bar | k (cm^3^molecule^−1^ s ^−1^) | T/K | P/bar | k (cm^3^molecule^−1^ s ^−1^) |
| 200 | 1.00$E$+00 | 3.1E-11 | 200 | 1.00$E$+01 | 1.13E-10 |
| 210 | 1.00$E$+00 | 2.14E-11 | 210 | 1.00$E$+01 | 8.03E-11 |
| 220 | 1.00$E$+00 | 1.53E-11 | 220 | 1.00$E$+01 | 5.94E-11 |
| 230 | 1.00$E$+00 | 1.13E-11 | 230 | 1.00$E$+01 | 4.53E-11 |
| 240 | 1.00$E$+00 | 8.56E-12 | 240 | 1.00$E$+01 | 3.55E-11 |
| 250 | 1.00$E$+00 | 6.61E-12 | 250 | 1.00$E$+01 | 2.83E-11 |
| 260 | 1.00$E$+00 | 5.22E-12 | 260 | 1.00$E$+01 | 2.31E-11 |
| 270 | 1.00$E$+00 | 4.18E-12 | 270 | 1.00$E$+01 | 1.91E-11 |
| 280 | 1.00$E$+00 | 3.41E-12 | 280 | 1.00$E$+01 | 1.6E-11 |
| 290 | 1.00$E$+00 | 2.81E-12 | 290 | 1.00$E$+01 | 1.35E-11 |
| 298 | 1.00$E$+00 | 2.43E-12 | 298 | 1.00$E$+01 | 1.2E-11 |
| 298.15 | 1.00$E$+00 | 2.42E-12 | 298.15 | 1.00$E$+01 | 1.19E-11 |
| 300 | 1.00$E$+00 | 2.35E-12 | 300 | 1.00$E$+01 | 1.16E-11 |
| 310 | 1.00$E$+00 | 1.98E-12 | 310 | 1.00$E$+01 | 1E-11 |
| 320 | 1.00$E$+00 | 1.69E-12 | 320 | 1.00$E$+01 | 8.76E-12 |
| 330 | 1.00$E$+00 | 1.45E-12 | 330 | 1.00$E$+01 | 7.7E-12 |
| 340 | 1.00$E$+00 | 1.26E-12 | 340 | 1.00$E$+01 | 6.8E-12 |
| 350 | 1.00$E$+00 | 1.1E-12 | 350 | 1.00$E$+01 | 6.06E-12 |
| 360 | 1.00$E$+00 | 9.69E-13 | 360 | 1.00$E$+01 | 5.43E-12 |
| 370 | 1.00$E$+00 | 8.19E-13 | 370 | 1.00$E$+01 | 4.75E-12 |
| 380 | 1.00$E$+00 | 7.29E-13 | 380 | 1.00$E$+01 | 4.3E-12 |
| 390 | 1.00$E$+00 | 6.54E-13 | 390 | 1.00$E$+01 | 3.91E-12 |
| 400 | 1.00$E$+00 | 5.89E-13 | 400 | 1.00$E$+01 | 3.57E-12 |
| 410 | 1.00$E$+00 | 5.33E-13 | 410 | 1.00$E$+01 | 3.27E-12 |
| 420 | 1.00$E$+00 | 4.86E-13 | 420 | 1.00$E$+01 | 3.02E-12 |
| 430 | 1.00$E$+00 | 4.44E-13 | 430 | 1.00$E$+01 | 2.79E-12 |
| 440 | 1.00$E$+00 | 4.08E-13 | 440 | 1.00$E$+01 | 2.59E-12 |
| 450 | 1.00$E$+00 | 3.76E-13 | 450 | 1.00$E$+01 | 2.41E-12 |
| 460 | 1.00$E$+00 | 3.48E-13 | 460 | 1.00$E$+01 | 2.26E-12 |
| 470 | 1.00$E$+00 | 3.24E-13 | 470 | 1.00$E$+01 | 2.12E-12 |
| 480 | 1.00$E$+00 | 3.02E-13 | 480 | 1.00$E$+01 | 1.99E-12 |
| 490 | 1.00$E$+00 | 2.83E-13 | 490 | 1.00$E$+01 | 1.88E-12 |
| 500 | 1.00$E$+00 | 2.43E-13 | 500 | 1.00$E$+01 | 1.68E-12 |
| 510 | 1.00$E$+00 | 2.29E-13 | 510 | 1.00$E$+01 | 1.59E-12 |
| 520 | 1.00$E$+00 | 2.15E-13 | 520 | 1.00$E$+01 | 1.51E-12 |
| 530 | 1.00$E$+00 | 2.04E-13 | 530 | 1.00$E$+01 | 1.44E-12 |
| 540 | 1.00$E$+00 | 1.93E-13 | 540 | 1.00$E$+01 | 1.37E-12 |
| 550 | 1.00$E$+00 | 1.83E-13 | 550 | 1.00$E$+01 | 1.31E-12 |
| 560 | 1.00$E$+00 | 1.74E-13 | 560 | 1.00$E$+01 | 1.25E-12 |
| 570 | 1.00$E$+00 | 1.66E-13 | 570 | 1.00$E$+01 | 1.2E-12 |
| 580 | 1.00$E$+00 | 1.59E-13 | 580 | 1.00$E$+01 | 1.16E-12 |
| 590 | 1.00$E$+00 | 1.52E-13 | 590 | 1.00$E$+01 | 1.12E-12 |
| 600 | 1.00$E$+00 | 1.46E-13 | 600 | 1.00$E$+01 | 1.08E-12 |
| 610 | 1.00$E$+00 | 1.41E-13 | 610 | 1.00$E$+01 | 1.04E-12 |
| 620 | 1.00$E$+00 | 1.07E-13 | 620 | 1.00$E$+01 | 8.27E-13 |
| 630 | 1.00$E$+00 | 1.03E-13 | 630 | 1.00$E$+01 | 8E-13 |
| 640 | 1.00$E$+00 | 9.93E-14 | 640 | 1.00$E$+01 | 7.75E-13 |
| 650 | 1.00$E$+00 | 9.59E-14 | 650 | 1.00$E$+01 | 7.52E-13 |
| 660 | 1.00$E$+00 | 9.29E-14 | 660 | 1.00$E$+01 | 7.3E-13 |
| 670 | 1.00$E$+00 | 8.99E-14 | 670 | 1.00$E$+01 | 7.1E-13 |
| 680 | 1.00$E$+00 | 8.73E-14 | 680 | 1.00$E$+01 | 6.92E-13 |
| 690 | 1.00$E$+00 | 8.48E-14 | 690 | 1.00$E$+01 | 6.74E-13 |
| 700 | 1.00$E$+00 | 8.25E-14 | 700 | 1.00$E$+01 | 6.58E-13 |
| 710 | 1.00$E$+00 | 8.03E-14 | 710 | 1.00$E$+01 | 6.43E-13 |
| 720 | 1.00$E$+00 | 7.83E-14 | 720 | 1.00$E$+01 | 6.29E-13 |
| 730 | 1.00$E$+00 | 7.64E-14 | 730 | 1.00$E$+01 | 6.15E-13 |
| 740 | 1.00$E$+00 | 6.48E-14 | 740 | 1.00$E$+01 | 5.16E-13 |
| 750 | 1.00$E$+00 | 6.35E-14 | 750 | 1.00$E$+01 | 5.07E-13 |
| 760 | 1.00$E$+00 | 6.22E-14 | 760 | 1.00$E$+01 | 4.98E-13 |
| 770 | 1.00$E$+00 | 6.1E-14 | 770 | 1.00$E$+01 | 4.89E-13 |
| 780 | 1.00$E$+00 | 5.99E-14 | 780 | 1.00$E$+01 | 4.81E-13 |
| 790 | 1.00$E$+00 | 5.88E-14 | 790 | 1.00$E$+01 | 4.74E-13 |
| 800 | 1.00$E$+00 | 5.78E-14 | 800 | 1.00$E$+01 | 4.66E-13 |
| T/K | P/bar | k (cm^3^molecule^−1^ s ^−1^) | T/K | P/bar | k (cm^3^molecule^−1^ s ^−1^) |
| 200 | 1.00$E$+02 | 2.89E-10 | 200 | 1.00$E$+03 | 4.48E-10 |
| 210 | 1.00$E$+02 | 2.13E-10 | 210 | 1.00$E$+03 | 3.39E-10 |
| 220 | 1.00$E$+02 | 1.62E-10 | 220 | 1.00$E$+03 | 2.66E-10 |
| 230 | 1.00$E$+02 | 1.28E-10 | 230 | 1.00$E$+03 | 2.15E-10 |
| 240 | 1.00$E$+02 | 1.03E-10 | 240 | 1.00$E$+03 | 1.78E-10 |
| 250 | 1.00$E$+02 | 8.5E-11 | 250 | 1.00$E$+03 | 1.51E-10 |
| 260 | 1.00$E$+02 | 7.14E-11 | 260 | 1.00$E$+03 | 1.3E-10 |
| 270 | 1.00$E$+02 | 6.1E-11 | 270 | 1.00$E$+03 | 1.14E-10 |
| 280 | 1.00$E$+02 | 5.27E-11 | 280 | 1.00$E$+03 | 1.02E-10 |
| 290 | 1.00$E$+02 | 4.61E-11 | 290 | 1.00$E$+03 | 9.14E-11 |
| 298 | 1.00$E$+02 | 4.17E-11 | 298 | 1.00$E$+03 | 8.47E-11 |
| 298.15 | 1.00$E$+02 | 4.16E-11 | 298.15 | 1.00$E$+03 | 8.45E-11 |
| 300 | 1.00$E$+02 | 4.07E-11 | 300 | 1.00$E$+03 | 8.31E-11 |
| 310 | 1.00$E$+02 | 3.62E-11 | 310 | 1.00$E$+03 | 7.62E-11 |
| 320 | 1.00$E$+02 | 3.25E-11 | 320 | 1.00$E$+03 | 7.03E-11 |
| 330 | 1.00$E$+02 | 2.93E-11 | 330 | 1.00$E$+03 | 6.53E-11 |
| 340 | 1.00$E$+02 | 2.66E-11 | 340 | 1.00$E$+03 | 6.09E-11 |
| 350 | 1.00$E$+02 | 2.43E-11 | 350 | 1.00$E$+03 | 5.72E-11 |
| 360 | 1.00$E$+02 | 2.22E-11 | 360 | 1.00$E$+03 | 5.39E-11 |
| 370 | 1.00$E$+02 | 2.02E-11 | 370 | 1.00$E$+03 | 5.08E-11 |
| 380 | 1.00$E$+02 | 1.87E-11 | 380 | 1.00$E$+03 | 4.81E-11 |
| 390 | 1.00$E$+02 | 1.73E-11 | 390 | 1.00$E$+03 | 4.58E-11 |
| 400 | 1.00$E$+02 | 1.61E-11 | 400 | 1.00$E$+03 | 4.36E-11 |
| 410 | 1.00$E$+02 | 1.51E-11 | 410 | 1.00$E$+03 | 4.18E-11 |
| 420 | 1.00$E$+02 | 1.41E-11 | 420 | 1.00$E$+03 | 4E-11 |
| 430 | 1.00$E$+02 | 1.33E-11 | 430 | 1.00$E$+03 | 3.85E-11 |
| 440 | 1.00$E$+02 | 1.25E-11 | 440 | 1.00$E$+03 | 3.7E-11 |
| 450 | 1.00$E$+02 | 1.18E-11 | 450 | 1.00$E$+03 | 3.57E-11 |
| 460 | 1.00$E$+02 | 1.12E-11 | 460 | 1.00$E$+03 | 3.44E-11 |
| 470 | 1.00$E$+02 | 1.06E-11 | 470 | 1.00$E$+03 | 3.33E-11 |
| 480 | 1.00$E$+02 | 1.01E-11 | 480 | 1.00$E$+03 | 3.23E-11 |
| 490 | 1.00$E$+02 | 9.64E-12 | 490 | 1.00$E$+03 | 3.13E-11 |
| 500 | 1.00$E$+02 | 8.95E-12 | 500 | 1.00$E$+03 | 3.01E-11 |
| 510 | 1.00$E$+02 | 8.58E-12 | 510 | 1.00$E$+03 | 2.93E-11 |
| 520 | 1.00$E$+02 | 8.22E-12 | 520 | 1.00$E$+03 | 2.85E-11 |
| 530 | 1.00$E$+02 | 7.9E-12 | 530 | 1.00$E$+03 | 2.78E-11 |
| 540 | 1.00$E$+02 | 7.61E-12 | 540 | 1.00$E$+03 | 2.71E-11 |
| 550 | 1.00$E$+02 | 7.34E-12 | 550 | 1.00$E$+03 | 2.65E-11 |
| 560 | 1.00$E$+02 | 7.09E-12 | 560 | 1.00$E$+03 | 2.59E-11 |
| 570 | 1.00$E$+02 | 6.86E-12 | 570 | 1.00$E$+03 | 2.54E-11 |
| 580 | 1.00$E$+02 | 6.65E-12 | 580 | 1.00$E$+03 | 2.49E-11 |
| 590 | 1.00$E$+02 | 6.46E-12 | 590 | 1.00$E$+03 | 2.44E-11 |
| 600 | 1.00$E$+02 | 6.28E-12 | 600 | 1.00$E$+03 | 2.4E-11 |
| 610 | 1.00$E$+02 | 6.11E-12 | 610 | 1.00$E$+03 | 2.35E-11 |
| 620 | 1.00$E$+02 | 5.27E-12 | 620 | 1.00$E$+03 | 2.22E-11 |
| 630 | 1.00$E$+02 | 5.13E-12 | 630 | 1.00$E$+03 | 2.18E-11 |
| 640 | 1.00$E$+02 | 5.01E-12 | 640 | 1.00$E$+03 | 2.15E-11 |
| 650 | 1.00$E$+02 | 4.89E-12 | 650 | 1.00$E$+03 | 2.12E-11 |
| 660 | 1.00$E$+02 | 4.78E-12 | 660 | 1.00$E$+03 | 2.09E-11 |
| 670 | 1.00$E$+02 | 4.68E-12 | 670 | 1.00$E$+03 | 2.06E-11 |
| 680 | 1.00$E$+02 | 4.58E-12 | 680 | 1.00$E$+03 | 2.04E-11 |
| 690 | 1.00$E$+02 | 4.49E-12 | 690 | 1.00$E$+03 | 2.02E-11 |
| 700 | 1.00$E$+02 | 4.41E-12 | 700 | 1.00$E$+03 | 1.99E-11 |
| 710 | 1.00$E$+02 | 4.33E-12 | 710 | 1.00$E$+03 | 1.97E-11 |
| 720 | 1.00$E$+02 | 4.26E-12 | 720 | 1.00$E$+03 | 1.95E-11 |
| 730 | 1.00$E$+02 | 4.19E-12 | 730 | 1.00$E$+03 | 1.94E-11 |
| 740 | 1.00$E$+02 | 3.61E-12 | 740 | 1.00$E$+03 | 1.79E-11 |
| 750 | 1.00$E$+02 | 3.56E-12 | 750 | 1.00$E$+03 | 1.78E-11 |
| 760 | 1.00$E$+02 | 3.51E-12 | 760 | 1.00$E$+03 | 1.76E-11 |
| 770 | 1.00$E$+02 | 3.46E-12 | 770 | 1.00$E$+03 | 1.75E-11 |
| 780 | 1.00$E$+02 | 3.41E-12 | 780 | 1.00$E$+03 | 1.74E-11 |
| 790 | 1.00$E$+02 | 3.37E-12 | 790 | 1.00$E$+03 | 1.73E-11 |
| 800 | 1.00$E$+02 | 3.33E-12 | 800 | 1.00$E$+03 | 1.72E-11 |
| T/K | P/bar | k (cm^3^molecule^−1^ s ^−1^) | T/K | P/bar | k (cm^3^molecule^−1^ s ^−1^) |
| 200 | 1.00$E$+04 | 4.95E-10 | 200 | 1.00$E$+05 | 5.01E-10 |
| 210 | 1.00$E$+04 | 3.79E-10 | 210 | 1.00$E$+05 | 3.84E-10 |
| 220 | 1.00$E$+04 | 3.01E-10 | 220 | 1.00$E$+05 | 3.05E-10 |
| 230 | 1.00$E$+04 | 2.46E-10 | 230 | 1.00$E$+05 | 2.49E-10 |
| 240 | 1.00$E$+04 | 2.06E-10 | 240 | 1.00$E$+05 | 2.1E-10 |
| 250 | 1.00$E$+04 | 1.77E-10 | 250 | 1.00$E$+05 | 1.81E-10 |
| 260 | 1.00$E$+04 | 1.55E-10 | 260 | 1.00$E$+05 | 1.58E-10 |
| 270 | 1.00$E$+04 | 1.37E-10 | 270 | 1.00$E$+05 | 1.41E-10 |
| 280 | 1.00$E$+04 | 1.24E-10 | 280 | 1.00$E$+05 | 1.28E-10 |
| 290 | 1.00$E$+04 | 1.13E-10 | 290 | 1.00$E$+05 | 1.17E-10 |
| 298 | 1.00$E$+04 | 1.06E-10 | 298 | 1.00$E$+05 | 1.1E-10 |
| 298.15 | 1.00$E$+04 | 1.06E-10 | 298.15 | 1.00$E$+05 | 1.09E-10 |
| 300 | 1.00$E$+04 | 1.04E-10 | 300 | 1.00$E$+05 | 1.08E-10 |
| 310 | 1.00$E$+04 | 9.7E-11 | 310 | 1.00$E$+05 | 1.01E-10 |
| 320 | 1.00$E$+04 | 9.09E-11 | 320 | 1.00$E$+05 | 9.48E-11 |
| 330 | 1.00$E$+04 | 8.58E-11 | 330 | 1.00$E$+05 | 8.98E-11 |
| 340 | 1.00$E$+04 | 8.15E-11 | 340 | 1.00$E$+05 | 8.56E-11 |
| 350 | 1.00$E$+04 | 7.77E-11 | 350 | 1.00$E$+05 | 8.21E-11 |
| 360 | 1.00$E$+04 | 7.46E-11 | 360 | 1.00$E$+05 | 7.9E-11 |
| 370 | 1.00$E$+04 | 7.18E-11 | 370 | 1.00$E$+05 | 7.64E-11 |
| 380 | 1.00$E$+04 | 6.94E-11 | 380 | 1.00$E$+05 | 7.42E-11 |
| 390 | 1.00$E$+04 | 6.73E-11 | 390 | 1.00$E$+05 | 7.23E-11 |
| 400 | 1.00$E$+04 | 6.54E-11 | 400 | 1.00$E$+05 | 7.07E-11 |
| 410 | 1.00$E$+04 | 6.37E-11 | 410 | 1.00$E$+05 | 6.93E-11 |
| 420 | 1.00$E$+04 | 6.22E-11 | 420 | 1.00$E$+05 | 6.81E-11 |
| 430 | 1.00$E$+04 | 6.09E-11 | 430 | 1.00$E$+05 | 6.7E-11 |
| 440 | 1.00$E$+04 | 5.97E-11 | 440 | 1.00$E$+05 | 6.61E-11 |
| 450 | 1.00$E$+04 | 5.87E-11 | 450 | 1.00$E$+05 | 6.53E-11 |
| 460 | 1.00$E$+04 | 5.77E-11 | 460 | 1.00$E$+05 | 6.47E-11 |
| 470 | 1.00$E$+04 | 5.67E-11 | 470 | 1.00$E$+05 | 6.41E-11 |
| 480 | 1.00$E$+04 | 5.6E-11 | 480 | 1.00$E$+05 | 6.36E-11 |
| 490 | 1.00$E$+04 | 5.52E-11 | 490 | 1.00$E$+05 | 6.32E-11 |
| 500 | 1.00$E$+04 | 5.45E-11 | 500 | 1.00$E$+05 | 6.29E-11 |
| 510 | 1.00$E$+04 | 5.38E-11 | 510 | 1.00$E$+05 | 6.26E-11 |
| 520 | 1.00$E$+04 | 5.33E-11 | 520 | 1.00$E$+05 | 6.24E-11 |
| 530 | 1.00$E$+04 | 5.27E-11 | 530 | 1.00$E$+05 | 6.22E-11 |
| 540 | 1.00$E$+04 | 5.23E-11 | 540 | 1.00$E$+05 | 6.22E-11 |
| 550 | 1.00$E$+04 | 5.18E-11 | 550 | 1.00$E$+05 | 6.2E-11 |
| 560 | 1.00$E$+04 | 5.15E-11 | 560 | 1.00$E$+05 | 6.2E-11 |
| 570 | 1.00$E$+04 | 5.11E-11 | 570 | 1.00$E$+05 | 6.2E-11 |
| 580 | 1.00$E$+04 | 5.07E-11 | 580 | 1.00$E$+05 | 6.21E-11 |
| 590 | 1.00$E$+04 | 5.04E-11 | 590 | 1.00$E$+05 | 6.22E-11 |
| 600 | 1.00$E$+04 | 5.02E-11 | 600 | 1.00$E$+05 | 6.22E-11 |
| 610 | 1.00$E$+04 | 4.99E-11 | 610 | 1.00$E$+05 | 6.24E-11 |
| 620 | 1.00$E$+04 | 4.94E-11 | 620 | 1.00$E$+05 | 6.26E-11 |
| 630 | 1.00$E$+04 | 4.91E-11 | 630 | 1.00$E$+05 | 6.28E-11 |
| 640 | 1.00$E$+04 | 4.89E-11 | 640 | 1.00$E$+05 | 6.3E-11 |
| 650 | 1.00$E$+04 | 4.87E-11 | 650 | 1.00$E$+05 | 6.32E-11 |
| 660 | 1.00$E$+04 | 4.86E-11 | 660 | 1.00$E$+05 | 6.34E-11 |
| 670 | 1.00$E$+04 | 4.85E-11 | 670 | 1.00$E$+05 | 6.37E-11 |
| 680 | 1.00$E$+04 | 4.84E-11 | 680 | 1.00$E$+05 | 6.4E-11 |
| 690 | 1.00$E$+04 | 4.82E-11 | 690 | 1.00$E$+05 | 6.42E-11 |
| 700 | 1.00$E$+04 | 4.82E-11 | 700 | 1.00$E$+05 | 6.46E-11 |
| 710 | 1.00$E$+04 | 4.81E-11 | 710 | 1.00$E$+05 | 6.5E-11 |
| 720 | 1.00$E$+04 | 4.81E-11 | 720 | 1.00$E$+05 | 6.52E-11 |
| 730 | 1.00$E$+04 | 4.8E-11 | 730 | 1.00$E$+05 | 6.56E-11 |
| 740 | 1.00$E$+04 | 4.74E-11 | 740 | 1.00$E$+05 | 6.6E-11 |
| 750 | 1.00$E$+04 | 4.74E-11 | 750 | 1.00$E$+05 | 6.63E-11 |
| 760 | 1.00$E$+04 | 4.73E-11 | 760 | 1.00$E$+05 | 6.67E-11 |
| 770 | 1.00$E$+04 | 4.74E-11 | 770 | 1.00$E$+05 | 6.71E-11 |
| 780 | 1.00$E$+04 | 4.74E-11 | 780 | 1.00$E$+05 | 6.75E-11 |
| 790 | 1.00$E$+04 | 4.75E-11 | 790 | 1.00$E$+05 | 6.79E-11 |
| 800 | 1.00$E$+04 | 4.75E-11 | 800 | 1.00$E$+05 | 6.84E-11 |
| T/K | P/bar | k (cm^3^molecule^−1^ s ^−1^) | T/K | P/bar | k (cm^3^molecule^−1^ s ^−1^) |
| 200 | 1.00$E$+06 | 5.01E-10 | 200 | 1.00$E$+07 | 5.01E-10 |
| 210 | 1.00$E$+06 | 3.84E-10 | 210 | 1.00$E$+07 | 3.84E-10 |
| 220 | 1.00$E$+06 | 3.05E-10 | 220 | 1.00$E$+07 | 3.05E-10 |
| 230 | 1.00$E$+06 | 2.5E-10 | 230 | 1.00$E$+07 | 2.51E-10 |
| 240 | 1.00$E$+06 | 2.1E-10 | 240 | 1.00$E$+07 | 2.1E-10 |
| 250 | 1.00$E$+06 | 1.81E-10 | 250 | 1.00$E$+07 | 1.81E-10 |
| 260 | 1.00$E$+06 | 1.59E-10 | 260 | 1.00$E$+07 | 1.59E-10 |
| 270 | 1.00$E$+06 | 1.42E-10 | 270 | 1.00$E$+07 | 1.42E-10 |
| 280 | 1.00$E$+06 | 1.28E-10 | 280 | 1.00$E$+07 | 1.28E-10 |
| 290 | 1.00$E$+06 | 1.17E-10 | 290 | 1.00$E$+07 | 1.17E-10 |
| 298 | 1.00$E$+06 | 1.1E-10 | 298 | 1.00$E$+07 | 1.1E-10 |
| 298.15 | 1.00$E$+06 | 1.1E-10 | 298.15 | 1.00$E$+07 | 1.1E-10 |
| 300 | 1.00$E$+06 | 1.08E-10 | 300 | 1.00$E$+07 | 1.08E-10 |
| 310 | 1.00$E$+06 | 1.01E-10 | 310 | 1.00$E$+07 | 1.01E-10 |
| 320 | 1.00$E$+06 | 9.52E-11 | 320 | 1.00$E$+07 | 9.53E-11 |
| 330 | 1.00$E$+06 | 9.03E-11 | 330 | 1.00$E$+07 | 9.03E-11 |
| 340 | 1.00$E$+06 | 8.6E-11 | 340 | 1.00$E$+07 | 8.62E-11 |
| 350 | 1.00$E$+06 | 8.26E-11 | 350 | 1.00$E$+07 | 8.25E-11 |
| 360 | 1.00$E$+06 | 7.96E-11 | 360 | 1.00$E$+07 | 7.95E-11 |
| 370 | 1.00$E$+06 | 7.7E-11 | 370 | 1.00$E$+07 | 7.68E-11 |
| 380 | 1.00$E$+06 | 7.48E-11 | 380 | 1.00$E$+07 | 7.48E-11 |
| 390 | 1.00$E$+06 | 7.3E-11 | 390 | 1.00$E$+07 | 7.3E-11 |
| 400 | 1.00$E$+06 | 7.14E-11 | 400 | 1.00$E$+07 | 7.14E-11 |
| 410 | 1.00$E$+06 | 7.00E-11 | 410 | 1.00$E$+07 | 7.00E-11 |
| 420 | 1.00$E$+06 | 6.88E-11 | 420 | 1.00$E$+07 | 6.89E-11 |
| 430 | 1.00$E$+06 | 6.77E-11 | 430 | 1.00$E$+07 | 6.79E-11 |
| 440 | 1.00$E$+06 | 6.69E-11 | 440 | 1.00$E$+07 | 6.69E-11 |
| 450 | 1.00$E$+06 | 6.62E-11 | 450 | 1.00$E$+07 | 6.62E-11 |
| 460 | 1.00$E$+06 | 6.56E-11 | 460 | 1.00$E$+07 | 6.56E-11 |
| 470 | 1.00$E$+06 | 6.5E-11 | 470 | 1.00$E$+07 | 6.52E-11 |
| 480 | 1.00$E$+06 | 6.46E-11 | 480 | 1.00$E$+07 | 6.48E-11 |
| 490 | 1.00$E$+06 | 6.43E-11 | 490 | 1.00$E$+07 | 6.43E-11 |
| 500 | 1.00$E$+06 | 6.4E-11 | 500 | 1.00$E$+07 | 6.41E-11 |
| 510 | 1.00$E$+06 | 6.38E-11 | 510 | 1.00$E$+07 | 6.4E-11 |
| 520 | 1.00$E$+06 | 6.36E-11 | 520 | 1.00$E$+07 | 6.38E-11 |
| 530 | 1.00$E$+06 | 6.35E-11 | 530 | 1.00$E$+07 | 6.37E-11 |
| 540 | 1.00$E$+06 | 6.36E-11 | 540 | 1.00$E$+07 | 6.37E-11 |
| 550 | 1.00$E$+06 | 6.36E-11 | 550 | 1.00$E$+07 | 6.37E-11 |
| 560 | 1.00$E$+06 | 6.37E-11 | 560 | 1.00$E$+07 | 6.38E-11 |
| 570 | 1.00$E$+06 | 6.38E-11 | 570 | 1.00$E$+07 | 6.39E-11 |
| 580 | 1.00$E$+06 | 6.39E-11 | 580 | 1.00$E$+07 | 6.4E-11 |
| 590 | 1.00$E$+06 | 6.41E-11 | 590 | 1.00$E$+07 | 6.43E-11 |
| 600 | 1.00$E$+06 | 6.42E-11 | 600 | 1.00$E$+07 | 6.45E-11 |
| 610 | 1.00$E$+06 | 6.45E-11 | 610 | 1.00$E$+07 | 6.47E-11 |
| 620 | 1.00$E$+06 | 6.48E-11 | 620 | 1.00$E$+07 | 6.49E-11 |
| 630 | 1.00$E$+06 | 6.51E-11 | 630 | 1.00$E$+07 | 6.53E-11 |
| 640 | 1.00$E$+06 | 6.54E-11 | 640 | 1.00$E$+07 | 6.55E-11 |
| 650 | 1.00$E$+06 | 6.57E-11 | 650 | 1.00$E$+07 | 6.6E-11 |
| 660 | 1.00$E$+06 | 6.6E-11 | 660 | 1.00$E$+07 | 6.63E-11 |
| 670 | 1.00$E$+06 | 6.64E-11 | 670 | 1.00$E$+07 | 6.67E-11 |
| 680 | 1.00$E$+06 | 6.68E-11 | 680 | 1.00$E$+07 | 6.72E-11 |
| 690 | 1.00$E$+06 | 6.73E-11 | 690 | 1.00$E$+07 | 6.75E-11 |
| 700 | 1.00$E$+06 | 6.77E-11 | 700 | 1.00$E$+07 | 6.8E-11 |
| 710 | 1.00$E$+06 | 6.81E-11 | 710 | 1.00$E$+07 | 6.86E-11 |
| 720 | 1.00$E$+06 | 6.86E-11 | 720 | 1.00$E$+07 | 6.9E-11 |
| 730 | 1.00$E$+06 | 6.91E-11 | 730 | 1.00$E$+07 | 6.94E-11 |
| 740 | 1.00$E$+06 | 6.96E-11 | 740 | 1.00$E$+07 | 6.99E-11 |
| 750 | 1.00$E$+06 | 7.02E-11 | 750 | 1.00$E$+07 | 7.05E-11 |
| 760 | 1.00$E$+06 | 7.06E-11 | 760 | 1.00$E$+07 | 7.11E-11 |
| 770 | 1.00$E$+06 | 7.12E-11 | 770 | 1.00$E$+07 | 7.15E-11 |
| 780 | 1.00$E$+06 | 7.18E-11 | 780 | 1.00$E$+07 | 7.23E-11 |
| 790 | 1.00$E$+06 | 7.24E-11 | 790 | 1.00$E$+07 | 7.27E-11 |
| 800 | 1.00$E$+06 | 7.29E-11 | 800 | 1.00$E$+07 | 7.34E-11 |

**Table S31**. Pressure dependent rate constants calculated at the M06-2X/maTZ for P1 adducts via path 1 + path 2.

| Species | Methods | Cartesian coordinates |
| --- | --- | --- |
| N_2_H_4_ | MP2/aTZ | N 0.71022200 -0.07620800 0.10027700  N -0.71022200 -0.07624200 -0.10025200  H -1.13694500 -0.30436600 0.78692800  H -1.04907700 0.83800400 -0.37777400  H 1.13695200 -0.30462500 -0.78682500  H 1.04906900 0.83813200 0.37749800 |
|  | MP2/maTZ | N 0.70919000 -0.07530300 0.10072500  N -0.70919000 -0.07530200 -0.10072500  H -1.13643600 -0.31199200 0.78271800  H -1.05052200 0.83910900 -0.37074400  H 1.13643500 -0.31198700 -0.78272000  H 1.05052200 0.83910700 0.37074900 |
|  | MP2/6-311++g(3df,3pd) | N 0.70820000 -0.07605500 0.09987400  N -0.70820000 -0.07597300 -0.09993600  H -1.13577100 -0.30460200 0.78616700  H -1.04843600 0.83653300 -0.37905900  H 1.13576900 -0.30396100 -0.78641600  H 1.04843900 0.83622300 0.37974200 |
|  | M06-2X/aTZ | N -0.70563700 -0.07380300 -0.09844400  N 0.70563700 -0.07379800 0.09844700  H 1.14207700 -0.31731600 -0.77937100  H 1.05379800 0.83390900 0.38320500  H -1.14207600 -0.31727800 0.77938700  H -1.05379800 0.83389100 -0.38324400 |
|  | M06-2X /maTZ | N 0.70509300 -0.07335300 0.09810200  N -0.70509300 -0.07335300 -0.09810100  H -1.14181200 -0.32017000 0.77852000  H -1.05456500 0.83364300 -0.38378800  H 1.14181200 -0.32017100 -0.77852000  H 1.05456500 0.83364300 0.38378800 |
|  | M06-2X/6-311++g(3df,3pd) | N -0.70795900 -0.07674600 -0.09897600  N 0.70795800 -0.07485500 0.10041200  H 1.13697400 -0.31254500 -0.78243200  H 1.04931000 0.83930500 0.37217800  H -1.13697000 -0.29765000 0.78821600  H -1.04931100 0.83209800 -0.38801000 |
|  | M06-HF/aTZ | N -0.70183400 -0.07014000 -0.09502400  N 0.70183400 -0.07014000 0.09502400  H 1.14579500 -0.33643800 -0.76928300  H 1.05651400 0.82742000 0.39242700  H -1.14579500 -0.33644100 0.76928200  H -1.05651400 0.82742100 -0.39242400 |
|  | M06-HF/maTZ | N 0.70189200 -0.06975900 0.09506700  N -0.70189200 -0.06976000 -0.09506700  H -1.14701000 -0.33997600 0.76774400  H -1.05824000 0.82829400 -0.39049200  H 1.14701000 -0.33997800 -0.76774300  H 1.05824100 0.82829500 0.39048900 |
|  | M06-HF/6-311++g(3df,3pd) | N 0.70278300 -0.07131900 0.09598800  N -0.70278300 -0.07132900 -0.09598000  H -1.14473200 -0.32992900 0.77175800  H -1.05262900 0.82921500 -0.39072600  H 1.14473300 -0.33001100 -0.77172200  H 1.05262800 0.82925700 0.39064100 |
|  | B3LYP/aTZ | N 0.70957900 -0.07369200 0.09810600  N -0.70957900 -0.07369000 -0.09810800  H -1.14531800 -0.31871100 0.78065000  H -1.06002500 0.83454300 -0.38375100  H 1.14531900 -0.31869200 -0.78065800  H 1.06002400 0.83453400 0.38377000 |
|  | B3LYP/maTZ | N 0.70943800 -0.07318000 0.09787700  N -0.70943800 -0.07318000 -0.09787700  H -1.14578800 -0.32228800 0.77926700  H -1.06128700 0.83454900 -0.38332000  H 1.14578800 -0.32228800 -0.77926700  H 1.06128700 0.83454900 0.38332100 |
|  | B3LYP/6-311++g(3df,3pd) | N -0.70943900 -0.07412900 -0.09834600  N 0.70943800 -0.07390600 0.09851000  H 1.14499700 -0.31786800 -0.78109900  H 1.05896600 0.83554100 0.38242600  H -1.14498900 -0.31610600 0.78181100  H -1.05897000 0.83468000 -0.38428200 |
|  | B3LYP/6-311g(d,p) | N 0.71069600 -0.07474900 -0.10063200  N -0.71069200 -0.07635000 0.09947400  H -1.05907700 0.83669700 0.38410900  H -1.14106900 -0.30460400 -0.78910800  H 1.14111300 -0.31696400 0.78420600  H 1.05900600 0.84256500 -0.37109900 |
| OH | MP2/aTZ | O 0.00000000 0.00000000 0.10771900  H 0.00000000 0.00000000 -0.86175200 |
|  | MP2/maTZ | O 0.00000000 0.00000000 0.10761600  H 0.00000000 0.00000000 -0.86092700 |
|  | MP2/6-311++g(3df,3pd) | O 0.00000000 0.00000000 0.10736500  H 0.00000000 0.00000000 -0.85892000 |
|  | M06-2X/aTZ | O 0.00000000 0.00000000 0.10799100  H 0.00000000 0.00000000 -0.86393200 |
|  | M06-2X /maTZ | O 0.00000000 0.00000000 0.10799100  H 0.00000000 0.00000000 -0.86393100 |
|  | M06-2X /6-311++g(3df,3pd) | O 0.00000000 0.00000000 0.10785300  H 0.00000000 0.00000000 -0.86282000 |
|  | M06-HF/aTZ | O 0.00000000 0.00000000 0.10791600  H 0.00000000 0.00000000 -0.86332500 |
|  | M06-HF /maTZ | O 0.00000000 0.00000000 0.10791600  H 0.00000000 0.00000000 -0.86332500 |
|  | M06-HF /6-311++g(3df,3pd) | O 0.00000000 0.00000000 0.10777800  H 0.00000000 0.00000000 -0.86222500 |
|  | B3LYP/aTZ | O 0.00000000 0.00000000 0.10833400  H 0.00000000 0.00000000 -0.86666800 |
|  | B3LYP/maTZ | O 0.00000000 0.00000000 0.10832500  H 0.00000000 0.00000000 -0.86660100 |
|  | B3LYP /6-311++g(3df,3pd) | O 0.00000000 0.00000000 0.10824400  H 0.00000000 0.00000000 -0.86595000 |
|  | B3LYP/6-311g(d,p) | O 0.00000000 0.00000000 0.10835700  H 0.00000000 0.00000000 -0.86685200 |
| N_2_H_3_ | MP2/aTZ | N 0.73421500 -0.15089200 0.02090100  N -0.59049500 0.02388400 -0.06568000  H -1.13045900 -0.79676300 0.14859100  H -1.01624100 0.90380500 0.18497700  H 1.14065700 0.78201700 -0.02012000 |
|  | MP2/maTZ | N 0.73426600 -0.15082100 0.02124600  N -0.59046000 0.02382700 -0.06652500  H -1.12929500 -0.79600100 0.15012200  H -1.01527800 0.90218700 0.18784700  H 1.13792700 0.78276800 -0.02101500 |
|  | MP2/6-311++g(3df,3pd) | N 0.73351800 -0.15076300 0.02066100  N -0.58969600 0.02384200 -0.06481000 H -1.13084100 -0.79662100 0.14654600  H -1.01636500 0.90414100 0.18231300  H 1.14045800 0.78092800 -0.01981500 |
|  | M06-2X/aTZ | N 0.73423800 -0.15131600 0.02154700  N -0.59084400 0.02436600 -0.06608200  H -1.13382200 -0.79628800 0.14739900  H -1.01740800 0.90433400 0.18731200  H 1.14746700 0.78060600 -0.02296600 |
|  | M06-2X /maTZ | N 0.73440100 -0.15129500 0.02159200  N -0.59071800 0.02434500 -0.06575800  H -1.13366000 -0.79696700 0.14599700  H -1.01866500 0.90414900 0.18680800  H 1.14654400 0.78146500 -0.02364100 |
|  | M06-2X/6-311++g(3df,3pd) | N 0.73402300 -0.15133500 0.02207300  N -0.59073600 0.02442400 -0.06798800  H -1.13303700 -0.79557500 0.15193600  H -1.01705200 0.90312000 0.19259800  H 1.14708200 0.78083600 -0.02312400 |
|  | M06-HF/aTZ | N 0.72939400 -0.15153100 0.01999700  N -0.58747200 0.02466200 -0.06066800  H -1.13621500 -0.79483100 0.13513800  H -1.00864800 0.91003700 0.17140300  H 1.15141500 0.77288000 -0.02184800 |
|  | M06-HF/maTZ | N 0.72999400 -0.15150100 0.01914900  N -0.58723700 0.02440200 -0.05855800  H -1.13813800 -0.79520700 0.13109100  H -1.01158800 0.91069600 0.16462200  H 1.15042600 0.77420600 -0.01984800 |
|  | M06-HF/6-311++g(3df,3pd) | N 0.73010700 -0.15124800 0.02016800  N -0.58831200 0.02455000 -0.06256200  H -1.13527700 -0.79385700 0.14112100  H -1.00880400 0.90845800 0.17577100  H 1.15151700 0.77228400 -0.02013200 |
|  | B3LYP/aTZ | N 0.73791400 -0.15103900 0.02255900  N -0.59476200 0.02444500 -0.06987300  H -1.13347400 -0.79649200 0.15646200  H -1.02179900 0.90174500 0.19843000  H 1.15321200 0.78090500 -0.02369800 |
|  | B3LYP/maTZ | N 0.73816100 -0.15101200 0.02281200  N -0.59484100 0.02445400 -0.07015300  H -1.13289200 -0.79697000 0.15632400  H -1.02263800 0.90114400 0.19982300  H 1.15228900 0.78173400 -0.02476600 |
|  | B3LYP/6-311++g(3df,3pd) | N 0.73789700 -0.15105300 0.02276700  N -0.59468400 0.02443400 -0.07055600  H -1.13332800 -0.79634700 0.15801400  H -1.02187300 0.90141800 0.20035700  H 1.15270400 0.78126000 -0.02384600 |
|  |  | N 0.74189100 -0.15080500 0.02581200  N -0.59696100 0.02465300 -0.07577900  H -1.12779100 -0.80090000 0.16276000  H -1.03022200 0.89370900 0.22127700  H 1.14350400 0.79025200 -0.03426500 |
| H_2_O | MP2/aTZ | O 0.00000000 0.00000000 0.11822800  H 0.00000000 0.75817200 -0.47291300  H 0.00000000 -0.75817200 -0.47291300 |
|  | MP2/maTZ | O 0.00000000 0.00000000 0.11822800  H 0.00000000 0.75817200 -0.47291300  H 0.00000000 -0.75817200 -0.47291300 |
|  | MP2/6-311++g(3df,3pd) | O 0.00000000 0.00000000 0.11815700  H 0.00000000 0.75525900 -0.47262900  H 0.00000000 -0.75525900 -0.47262900 |
|  | M06-2X/aTZ | O 0.00000000 0.00000000 0.11632500  H 0.00000000 -0.76291900 -0.46530000  H 0.00000000 0.76291900 -0.46530000 |
|  | M06-2X /maTZ | O 0.00000000 0.00000000 0.11645300  H 0.00000000 0.76231700 -0.46581300  H 0.00000000 -0.76231700 -0.46581300 |
|  | M06-2X/6-311++g(3df,3pd) | O 0.00000000 0.11666600 0.00000000  H 0.76095500 -0.46656900 0.00000000  H -0.76095500 -0.46675500 0.00000000 |
|  | M06-HF/aTZ | O 0.00000000 0.00000000 0.11384100  H 0.00000000 0.76879500 -0.45536300  H 0.00000000 -0.76879500 -0.45536300 |
|  | M06-HF /maTZ | O 0.00000000 0.00000000 0.11417800  H 0.00000000 0.76782700 -0.45671300  H 0.00000000 -0.76782700 -0.45671300 |
|  | M06-HF/6-311++g(3df,3pd) | O 0.00000000 0.00000000 0.11432000  H 0.00000000 0.76733800 -0.45727900  H 0.00000000 -0.76733800 -0.45727900 |
|  | B3LYP/aTZ | O 0.00000000 0.11699800 0.00000000  H 0.76351000 -0.46799100 0.00000000  H -0.76351000 -0.46799100 0.00000000 |
|  | B3LYP/maTZ | O 0.00000000 0.00000000 0.11704600  H 0.00000000 0.76328200 -0.46818600  H 0.00000000 -0.76328200 -0.46818600 |
|  | B3LYP/6-311++g(3df,3pd) | O 0.00000000 0.00000000 0.11697400  H 0.00000000 0.76274500 -0.46789500  H 0.00000000 -0.76274500 -0.46789500 |
|  | B3LYP/6-311g(d,p) | O 0.00000000 0.00000000 0.11864800  H 0.00000000 0.75705100 -0.47459200  H 0.00000000 -0.75705100 -0.47459200 |
| H_2_NOH | MP2/aTZ | O 0.72390800 0.00000900 -0.13966200  H 1.10694300 -0.00004700 0.74314500  H -1.03672200 -0.80930500 -0.35125900  N -0.68925100 -0.00000800 0.15379800  H -1.03672900 0.80933500 -0.35118000 |
|  | MP2/maTZ | O 0.72331400 -0.00013200 -0.13965800  H 1.10534400 0.00060300 0.74239400  H -1.03830200 -0.80765100 -0.34997500  N -0.68795200 0.00009700 0.15368300  H -1.03788800 0.80742500 -0.35094000 |
|  | MP2/6-311++g(3df,3pd) | O 0.72058800 0.00018700 -0.13955900  H 1.10624700 -0.00095400 0.73884800  H -1.03857800 -0.80840400 -0.34800400  N -0.68479300 -0.00015700 0.15315100  H -1.03882300 0.80895900 -0.34642900 |
|  | M06-2X/aTZ | O -0.71455900 0.00002800 -0.13976300  H -1.12176600 -0.00014200 0.72902600 H 1.04219200 0.81136900 -0.33784400  N 0.67912700 -0.00002300 0.15214200  H 1.04215100 -0.81129600 -0.33807100 |
|  | M06-2X /maTZ | O 0.71444200 0.00017000 -0.13981100  H 1.12172100 -0.00083500 0.72893100  H -1.04265300 -0.81108000 -0.33839900  N -0.67880700 -0.00013200 0.15214700  H -1.04294900 0.81148100 -0.33707500 |
|  | M06-2X/6-311++g(3df,3pd) | O 0.71278100 0.00062200 -0.13981100  H 1.12209500 -0.00320500 0.72716000  H -1.04190600 -0.81003900 -0.33991500  N -0.67713600 -0.00051900 0.15228200  H -1.04247800 0.81190300 -0.33472700 |
|  | M06-HF/aTZ | O 0.70950500 -0.00006000 -0.13872500  H 1.12989000 0.00026900 0.71898600  H -1.04444600 -0.81625400 -0.32155200  N -0.67388700 0.00004100 0.14776000  H -1.04426900 0.81617500 -0.32195400 |
|  | M06-HF/maTZ | O 0.70956900 -0.00008700 -0.13884000  H 1.12892800 0.00041300 0.71950900  H -1.04572800 -0.81592600 -0.32121600  N -0.67346000 0.00006200 0.14775200  H -1.04552700 0.81577400 -0.32183800 |
|  | M06-HF/6-311++g(3df,3pd) | O 0.70817800 -0.00016900 -0.13878400  H 1.12920300 0.00084300 0.71839000  H -1.04376500 -0.81532700 -0.32078100  N -0.67248000 0.00012700 0.14781800  H -1.04350400 0.81495000 -0.32206400 |
|  | B3LYP/aTZ | O 0.72483200 0.00000200 -0.13954100  H 1.12308600 -0.00001500 0.73630000  H -1.04495800 -0.81199400 -0.34473000  N -0.69026200 -0.00000200 0.15278000  H -1.04495000 0.81200800 -0.34470400 |
|  | B3LYP/maTZ | O 0.72483200 0.00000200 -0.13954100  H 1.12308600 -0.00001500 0.73630000  H -1.04495800 -0.81199400 -0.34473000  N -0.69026200 -0.00000200 0.15278000  H -1.04495000 0.81200800 -0.34470400 |
|  | B3LYP/6-311++g(3df,3pd) | O 0.72304100 0.00000900 -0.13950700  H 1.12243900 -0.00009100 0.73454100  H -1.04470400 -0.81214000 -0.34384200  N -0.68821400 -0.00001600 0.15271800  H -1.04456400 0.81227500 -0.34366800 |
|  | B3LYP/6-311g(d,p) | O -0.72575700 0.00009300 -0.13983300  H -1.11357800 -0.00047200 0.74004000  H 1.04498500 0.81047700 -0.35039600  N 0.68997100 -0.00007800 0.15431300  H 1.04485300 -0.81020600 -0.35117600 |
| NH_2_ | MP2/aTZ | N 0.00000000 0.14118700 0.00000000  H 0.80052300 -0.49415600 0.00000000  H -0.80052300 -0.49415600 0.00000000 |
|  | MP2/maTZ | N 0.00000000 0.00000000 0.14143500  H 0.00000000 0.79933700 -0.49502100  H 0.00000000 -0.79933700 -0.49502100 |
|  | MP2/6-311++g(3df,3pd) | N 0.00000000 0.14109200 0.00000000  H 0.80033700 -0.49378000 0.00000000  H -0.80033700 -0.49386600 0.00000000 |
|  | M06-2X/aTZ | N 0.00000000 0.14088000 0.00000000  H 0.80458900 -0.49308100 0.00000000  H -0.80458900 -0.49308100 0.00000000 |
|  | M06-2X /maTZ | N 0.00000000 0.00000000 0.14129000  H 0.00000000 0.80374000 -0.49451600  H 0.00000000 -0.80374000 -0.49451600 |
|  | M06-2X/6-311++g(3df,3pd) | N 0.00000000 0.14117100 0.00000000  H 0.80390300 -0.49407000 0.00000000  H -0.80390300 -0.49412900 0.00000000 |
|  | M06-HF/aTZ | N 0.00000000 0.00000000 0.13825800  H 0.00000000 0.80902300 -0.48390400  H 0.00000000 -0.80902300 -0.48390400 |
|  | M06-HF/maTZ | N 0.00000000 0.00000000 0.13868900  H 0.00000000 0.80811500 -0.48541300  H 0.00000000 -0.80811500 -0.48541300 |
|  | M06-HF/6-311++g(3df,3pd) | N 0.00000000 0.00000000 0.13840200  H 0.00000000 0.80826500 -0.48440900  H 0.00000000 -0.80826500 -0.48440900 |
|  | B3LYP/aTZ | N 0.00000000 0.14159200 0.00000000  H 0.80549300 -0.49557100 0.00000000  H -0.80549300 -0.49557100 0.00000000 |
|  | B3LYP/maTZ | N 0.00000000 0.00000000 0.14159200  H 0.00000000 0.80549300 -0.49557100  H 0.00000000 -0.80549300 -0.49557100 |
|  | B3LYP/6-311++g(3df,3pd) | N 0.00000000 0.14163900 0.00000000  H 0.80544200 -0.49572400 0.00000000  H -0.80544200 -0.49574700 0.00000000 |
|  | B3LYP/6-311g(d,p) | N 0.00000000 0.00000000 0.14395300  H 0.00000000 0.80202800 -0.50383600  H 0.00000000 -0.80202800 -0.50383600 |
| H_2_NNHOH | MP2/aTZ | N 0.00375300 0.56664700 0.15430400  N 1.18923400 -0.15326800 -0.02163200  H 1.33348200 -0.71847100 0.80442000  H 1.11696200 -0.77558100 -0.82333300  H -0.03839600 1.21144600 -0.62724800  O -1.14628900 -0.28748800 -0.12632700  H -1.59264900 -0.31114000 0.72807100 |
|  | MP2/maTZ | N 0.00272000 0.56505100 0.15379600  N 1.18911200 -0.15205900 -0.02150900  H 1.33517500 -0.71630000 0.80385400  H 1.11942700 -0.77564400 -0.82152700  H -0.03878300 1.20960000 -0.62703500  O -1.14588600 -0.28754600 -0.12609200  H -1.59155300 -0.30823100 0.72743900 |
|  | MP2/6-311++g(3df,3pd) | N -0.00029500 0.56323800 0.15434500  N 1.18608800 -0.15102300 -0.02191900  H 1.33475600 -0.71588900 0.80302300  H 1.11644100 -0.77302900 -0.82347100  H -0.04006800 1.21618800 -0.61891100  O -1.14064300 -0.28675600 -0.12651700  H -1.58653500 -0.31872300 0.72451200 |
|  | M06-2X/aTZ | N -0.00651900 0.55725300 0.15340800  N 1.18374700 -0.14805500 -0.02339200  H 1.35170100 -0.70176700 0.80593800  H 1.11957100 -0.77454000 -0.82213800  H -0.04774700 1.23275000 -0.60043600  O -1.13373500 -0.28539300 -0.12612400  H -1.59424000 -0.33768700 0.71551900 |
|  | M06-2X/maTZ | N -0.00666900 0.55682700 0.15268200  N 1.18352100 -0.14785800 -0.02366000  H 1.35536000 -0.69746700 0.80764000  H 1.11941700 -0.77749000 -0.82010600  H -0.04797300 1.23274200 -0.60113500  O -1.13363900 -0.28564600 -0.12562200  H -1.59565800 -0.33540100 0.71542700 |
|  | M06-2X/6-311++g(3df,3pd) | N -0.00872800 0.55604700 0.15374200  N 1.18287000 -0.14670000 -0.02333400  H 1.34887400 -0.70379700 0.80443800  H 1.11792100 -0.77323100 -0.82232500  H -0.04932300 1.23511200 -0.59709300  O -1.13052400 -0.28533700 -0.12640100  H -1.59228100 -0.34082200 0.71333600 |
|  | M06-HF/aTZ | N -0.01289400 0.55001900 0.14957900  N 1.17836600 -0.14621800 -0.02645600  H 1.37726800 -0.67043700 0.81252500  H 1.11366800 -0.78060700 -0.81502800  H -0.05727900 1.25011600 -0.57814700  O -1.12532200 -0.28368100 -0.12383300  H -1.58939100 -0.35623000 0.70945600 |
|  | M06-HF/maTZ | N -0.01336000 0.54985000 0.14809800  N 1.17854100 -0.14570700 -0.02611500  H 1.37922800 -0.66679400 0.81456800  H 1.11513300 -0.78395400 -0.81203700  H -0.05712100 1.24955200 -0.58039100  O -1.12493500 -0.28514200 -0.12305000  H -1.59403400 -0.34666800 0.70838200 |
|  | M06-HF/6-311++g(3df,3pd) | N -0.01487900 0.55190800 0.14985700  N 1.17714700 -0.14603500 -0.02596700  H 1.36804400 -0.67603800 0.81078400  H 1.10794800 -0.77930800 -0.81497300  H -0.05816800 1.25077800 -0.57755600  O -1.12076300 -0.28487200 -0.12410700  H -1.58759000 -0.35756400 0.70737000 |
|  | B3LYP/aTZ | N 0.00941700 0.56174200 0.15360900  N 1.19186900 -0.15423400 -0.02058400  H 1.36130300 -0.71422600 0.80437100  H 1.15381300 -0.76482000 -0.83403400  H -0.03829300 1.22195500 -0.61390600  O -1.15911500 -0.28508600 -0.12628700  H -1.61290900 -0.31477400 0.72268400 |
|  | B3LYP/maTZ | N 0.00945800 0.56161000 0.15354800  N 1.19166000 -0.15423700 -0.02092000  H 1.36341600 -0.71218300 0.80497300  H 1.15297200 -0.76644700 -0.83327500  H -0.03836500 1.22171600 -0.61422200  O -1.15925400 -0.28482600 -0.12616300  H -1.61182000 -0.31609200 0.72342800 |
|  | B3LYP/6-311++g(3df,3pd) | N 0.00684400 0.56099600 0.15387500  N 1.19045100 -0.15322000 -0.02126800  H 1.36073300 -0.71211000 0.80458900  H 1.14848500 -0.76667700 -0.83270600  H -0.04039800 1.22408800 -0.61127700  O -1.15522000 -0.28477600 -0.12635700  H -1.60812700 -0.32152800 0.72200600 |
| MCr1a | MP2/aTZ | N -1.17587400 -0.64350300 -0.12274400  N -0.68000000 0.69715200 0.00685200  H -1.09757700 1.24741000 -0.73169500  H -0.94313100 1.11627300 0.89238000  H -0.46568000 -1.26093800 0.25053600  H -2.02172900 -0.77624100 0.41862000  O 2.04413600 -0.12731500 0.00648200  H 1.16614200 0.31647400 -0.07044900 |
|  | MP2/maTZ | N 1.18272200 -0.64157200 -0.12262500  N 0.68243900 0.69564000 0.00740400  H 0.94879900 1.11816800 0.88971400  H 1.09624000 1.24639300 -0.73193100  H 2.03103300 -0.77172200 0.41397800  O -2.05417800 -0.12792200 0.00665000  H 0.47847100 -1.26201600 0.25384700  H -1.17724900 0.31407900 -0.07226700 |
|  | MP2/6-311++g(3df,3pd) | N -1.19097300 -0.63472200 -0.12273700  N -0.67247600 0.69265300 0.00910900  H -1.06323800 1.24894900 -0.73888300  H -0.93848800 1.12200600 0.88834600  H -0.50692700 -1.26560200 0.27437400  H -2.05576800 -0.74691800 0.39198600  O 2.05489800 -0.13181600 0.00515800  H 1.16938000 0.29057900 -0.06169000 |
|  | M06-2X/aTZ | N 1.09983900 -0.65124100 0.11514800  N 0.67577600 0.69634500 -0.00869200  H 1.06646400 1.23604500 0.74944000  H 0.93604400 1.11565500 -0.89156200  H 1.95301700 -0.83545300 -0.39190400  O -1.95794500 -0.11640700 -0.00683700  H 0.36159300 -1.25288600 -0.22238900  H -1.08286500 0.35216400 0.06592200 |
|  | M06-2X /maTZ | N -1.08833800 0.67129400 -0.11938800  N -0.72151700 -0.70095900 0.00644500  H -1.18191300 -1.22066200 -0.72848900  H -0.99567800 -1.09891700 0.89800500  H -0.31450700 1.23075300 0.21789800  O 1.99209300 0.09894400 0.00881500  H -1.91272300 0.89412800 0.42432100  H 1.13705500 -0.38919800 -0.09166100 |
|  | M06-2X /6-311++g(3df,3pd) | N -1.07626200 -0.67473700 -0.12044100  N -0.72544300 0.70087900 0.00659400  H -1.00387400 1.09415900 0.89896800  H -1.19221800 1.21630200 -0.72750400  H -1.89978900 -0.90478200 0.42231100  O 1.98438500 -0.09479800 0.00830300  H -0.29841200 -1.22378700 0.22591700  H 1.13115200 0.39349600 -0.08918400 |
|  | M06-HF/aTZ | N 1.09914200 -0.65151600 -0.11512400  N 0.67605600 0.69641300 0.00873600  H 0.93743200 1.11574600 0.89125300  H 1.06635300 1.23562300 -0.74990500  H 1.95348000 -0.83559500 0.39000200  O -1.95784000 -0.11617600 0.00659300  H 0.36141200 -1.25271200 0.22431200  H -1.08234600 0.35206700 -0.06369200 |
|  | M06-HF/maTZ | N 1.14912700 -0.63412600 -0.11226300  N 0.66153600 0.69308600 0.00029500  H 0.85094100 1.10844700 0.90304100  H 1.07974000 1.25778100 -0.72415300  H 1.95248900 -0.80060500 0.47628700  O -1.98213600 -0.12986700 0.01038300  H 0.41149700 -1.28038100 0.13157000  H -1.11221300 0.34097500 -0.08603100 |
|  | M06-HF /6-311++g(3df,3pd) | - |
|  | B3LYP/aTZ | N 1.33535000 -0.57206300 -0.11997400  N 0.61292400 0.66072000 0.00927600  H 0.80664500 1.13592200 0.88554600  H 0.89572900 1.26988700 -0.74770100  H 2.21371700 -0.54906100 0.38542500  O -2.13778800 -0.17018000 0.00518800  H 0.76242600 -1.31091000 0.26697000  H -1.21412500 0.19500700 -0.05685700 |
|  | B3LYP/maTZ | N 1.31992400 -0.57998500 -0.11947700  N 0.62124700 0.66592500 0.00920600  H 0.82159100 1.13778000 0.88597000  H 0.91546100 1.26975300 -0.74773200  H 2.19772500 -0.57562900 0.38741200  O -2.13029500 -0.16673400 0.00568800  H 0.73109200 -1.30906300 0.26212000  H -1.21170700 0.20944500 -0.06137600 |
|  | B3LYP /6-311++g(3df,3pd) | N -1.31999300 -0.57900400 -0.12019100  N -0.61988500 0.66495300 0.00948500  H -0.82145000 1.13634200 0.88625900  H -0.91121500 1.26956800 -0.74825000  H -2.19927000 -0.57122800 0.38457500  O 2.12944200 -0.16661700 0.00516600  H -0.73445200 -1.30778000 0.26782100  H 1.20999400 0.20439400 -0.05678700 |
|  | B3LYP/6-311g(d,p) | N -1.05944000 -0.68899400 -0.12062300  N -0.73403100 0.70583800 0.00063200  H -0.97866300 1.09047300 0.91103400  H -1.25470600 1.21437800 -0.70509900  H -1.82219400 -0.94970100 0.49765800  O 1.96312900 -0.08906500 0.01224200  H -0.22856500 -1.20283700 0.15666000  H 1.13339100 0.44229700 -0.11825300 |
| MCr1b | MP2/aTZ | N -1.17587400 -0.64350300 -0.12274400  N -0.68000000 0.69715200 0.00685200  H -1.09757700 1.24741000 -0.73169500  H -0.94313100 1.11627300 0.89238000  H -0.46568000 -1.26093800 0.25053600  H -2.02172900 -0.77624100 0.41862000  O 2.04413600 -0.12731500 0.00648200  H 1.16614200 0.31647400 -0.07044900 |
|  | MP2/maTZ | N -1.18320200 -0.64138400 -0.12258800  N -0.68224900 0.69557900 0.00725800  H -1.09677300 1.24675300 -0.73136100  H -0.94740200 1.11792000 0.89002900  H -0.47872200 -1.26223100 0.25278000  H -2.03088300 -0.77140900 0.41503500  O 2.05431600 -0.12804200 0.00668300  H 1.17741400 0.31393700 -0.07263100 |
|  | MP2/6-311++g(3df,3pd) | N 1.19700300 -0.63200800 -0.12274600  N 0.66907600 0.69157100 0.00925900  H 0.93271700 1.12307600 0.88815100  H 1.05508500 1.25051100 -0.73923900  H 2.06295500 -0.73783100 0.39137500  O -2.05756800 -0.13381400 0.00526800  H 0.51785000 -1.26778000 0.27489900  H -1.17061600 0.28559300 -0.06292700 |
|  | M06-2X/aTZ | N -1.08658300 -0.67171600 -0.11966500  N -0.72038600 0.70115400 0.00591600  H -1.18471700 1.22100000 -0.72625100  H -0.99175700 1.09706300 0.89897000  H -0.31129500 -1.22957800 0.21699200  O 1.98899400 -0.09861900 0.00858400  H -1.90840000 -0.89446800 0.42777300  H 1.13300000 0.38887600 -0.08991000 |
|  | M06-2X /maTZ | N -1.08834100 0.67136700 0.11942300  N -0.72170100 -0.70097100 -0.00648800  H -0.99620300 -1.09891400 -0.89795200  H -1.18209800 -1.22058400 0.72851100  H -1.91290700 0.89412800 -0.42401700  O 1.99236900 0.09887700 -0.00882600  H -0.31463900 1.23069000 -0.21838700  H 1.13719700 -0.38910200 0.09191000 |
|  | M06-2X /6-311++g(3df,3pd) | N 1.07434800 -0.67555100 -0.12038900  N 0.72677500 0.70092600 0.00651000  H 1.00516200 1.09311100 0.89940700  H 1.19636500 1.21528900 -0.72657000  H 1.89657500 -0.90773600 0.42330500  O -1.98354700 -0.09404600 0.00851800  H 0.29443800 -1.22252000 0.22446700  H -1.13202200 0.39659800 -0.09160100 |
|  | M06-HF/aTZ | N -1.09992600 -0.65123500 -0.11507200  N -0.67579600 0.69635100 0.00848700  H -0.93464300 1.11531700 0.89192600  H -1.06761800 1.23628900 -0.74884900  H -1.95307800 -0.83514100 0.39212600  O 1.95806600 -0.11636300 0.00668100  H -0.36170000 -1.25305200 0.22221500  H 1.08256800 0.35168500 -0.06477900 |
|  | M06-HF /maTZ | N -1.11886200 -0.64632200 -0.11492900  N -0.67512300 0.69515500 0.00852300  H -0.93021200 1.11908600 0.89080200  H -1.05937300 1.23993000 -0.74919800  H -1.97403900 -0.81864800 0.39344300  O 1.97636700 -0.12070900 0.00701100  H -0.38989800 -1.26063900 0.22022300  H 1.10047900 0.34411200 -0.06651800 |
|  | M06-HF /6-311++g(3df,3pd) |  |
|  | B3LYP/aTZ | N 1.33535000 -0.57206300 -0.11997400  N 0.61292400 0.66072000 0.00927600  H 0.80664500 1.13592200 0.88554600  H 0.89572900 1.26988700 -0.74770100  H 2.21371700 -0.54906100 0.38542500  O -2.13778800 -0.17018000 0.00518800  H 0.76242600 -1.31091000 0.26697000  H -1.21412500 0.19500700 -0.05685700 |
|  | B3LYP/maTZ | N -1.32051600 -0.57967700 -0.11948000  N -0.62115400 0.66573200 0.00926400  H -0.82073800 1.13760500 0.88616700  H -0.91483800 1.26986600 -0.74760600  H -2.19856200 -0.57475400 0.38701000  O 2.13078600 -0.16679400 0.00575600  H -0.73237100 -1.30914100 0.26245400  H 1.21191600 0.20839500 -0.06255700 |
|  | B3LYP /6-311++g(3df,3pd) | N 1.31478500 -0.58136600 -0.12017600  N 0.62156300 0.66651600 0.00954100  H 0.82527000 1.13679300 0.88642900  H 0.91645500 1.26955500 -0.74801600  H 2.19465700 -0.57823900 0.38364500  O -2.12615400 -0.16556400 0.00516300  H 0.72554500 -1.30675300 0.26862300  H -1.20713300 0.20710000 -0.05754000 |
|  | B3LYP/6-311g(d,p) | N 1.05966000 -0.68903000 -0.12056200  N 0.73445600 0.70583300 0.00044400  H 0.97762100 1.09017700 0.91134200  H 1.25635800 1.21437200 -0.70434700  H 1.82172900 -0.95000400 0.49844300  O -1.96362000 -0.08898400 0.01218800  H 0.22844300 -1.20276700 0.15586800  H -1.13399800 0.44247000 -0.11798300 |
| MCr2 | MP2/aTZ | N -1.14365500 -0.32667100 -0.11636400  N 0.02070100 0.47749100 0.03423400  H 0.02095000 1.20574600 -0.68561600  H 0.12645200 0.88629800 1.10059700  H -1.19278900 -0.84531600 0.76683800  O 1.16014700 -0.29282400 -0.15990500  H -1.92062800 0.33878300 -0.08607300  H 1.54551600 -0.29865500 0.75841100 |
|  | MP2/maTZ | N -1.21151900 0.01070400 0.15565200  N -0.02345100 -0.06071500 -0.57653600  H -0.07833800 -0.93094600 -1.09284000  H 0.77265300 3.24723500 -0.14280200  H -1.22164600 -0.68584700 0.89624100  O 1.09929600 -0.33320700 0.31182900  H -1.25658200 0.92450200 0.58460000  H 1.63433600 0.46079300 0.20635900 |
|  | MP2/6-311++g(3df,3pd) | N -1.14177100 -0.32368100 -0.11628100  N 0.02230000 0.47752600 0.02990300  H 0.02262400 1.20077900 -0.69409000  H 0.12789700 0.89665500 1.09326300  H -1.17284000 -0.86428400 0.75289100  O 1.15346400 -0.29596200 -0.15618800  H -1.91856500 0.33889100 -0.05140600  H 1.54946300 -0.28125900 0.75348600 |
|  | M06-2X/aTZ | N -1.14301300 -0.32430500 -0.11566200  N 0.02367800 0.47086700 0.03330200  H 0.01799000 1.20468500 -0.68405000  H 0.10118800 0.91652800 1.07462700  H -1.18719600 -0.86674400 0.75247000  O 1.15622800 -0.29130200 -0.15489900  H -1.91781500 0.34401800 -0.06928600  H 1.57134900 -0.29401000 0.74194900 |
|  | M06-2X /maTZ | N -1.14857400 -0.31770300 -0.11292500  N 0.02523800 0.47164600 0.00883300  H 0.02945100 1.18351300 -0.72717800  H 0.11745900 0.97314400 1.06145100  H -1.12887000 -0.91131500 0.71997700  O 1.14615600 -0.30710300 -0.13996600  H -1.91198800 0.34636200 0.05077400  H 1.58805700 -0.21248300 0.74334900 |
|  | M06-2X /6-311++g(3df,3pd) | N 0.02590500 0.47614800 0.02004800  H 0.02896300 1.19680500 -0.70523400  H 0.11477400 0.92108600 1.09555700  H -1.12235000 -0.92714900 0.69995200  O 1.14722900 -0.29455600 -0.14898800  H -1.91555000 0.33634000 0.05426300  H 1.54875500 -0.29176800 0.75793000 |
|  | M06-HF/aTZ | N -1.14264500 -0.31603800 -0.11368900  N 0.02289500 0.46718500 0.03049000  H 0.02480900 1.20862900 -0.67899400  H 0.08653800 0.92423500 1.04243600  H -1.16026700 -0.91717200 0.71071800  O 1.14994500 -0.29482000 -0.14788900  H -1.92118000 0.34026100 -0.02416400  H 1.60879400 -0.25542300 0.71551300 |
|  | M06-HF /maTZ | N -1.14596800 -0.31246500 -0.11419500  N 0.02384700 0.46612800 0.01812300  H 0.03125700 1.20171500 -0.69547100  H 0.08968300 0.94633300 1.03401500  H -1.13229500 -0.93807500 0.69142000  O 1.14646000 -0.30148200 -0.14015900  H -1.91546200 0.34239300 0.04294700  H 1.60999100 -0.21615100 0.72086100 |
|  | M06-HF /6-311++g(3df,3pd) | N -1.14199600 -0.31513000 -0.11540900  N 0.02441200 0.46973400 0.02645700  H 0.02775200 1.20590900 -0.68639900  H 0.08541700 0.93155900 1.03784800  H -1.14074400 -0.92894500 0.69939400  O 1.14575900 -0.29727600 -0.14476700  H -1.91865000 0.33717800 0.01199000  H 1.60324400 -0.24971600 0.71796700 |
|  | B3LYP/aTZ | N -1.14760100 -0.33956100 0.01357100  N 0.01428000 0.45605400 -0.02390900  H 0.03159600 1.07353500 -0.84503700  H 0.08769800 1.00129800 0.95662200  H -1.63385200 -0.10008100 0.88611500  O 1.20318000 -0.29516600 -0.11714600  H -1.71260800 -0.13573700 -0.80410900  H 1.53497800 -0.29314000 0.81594200 |
|  | B3LYP/maTZ | - |
|  | B3LYP /6-311++g(3df,3pd) | N -1.14613000 -0.33735100 0.02551700  N 0.01584500 0.45373600 -0.03973000  H 0.03328600 1.04947800 -0.87541100  H 0.09158100 1.03003200 0.93619600  H -1.63670300 -0.05964600 0.88469100  O 1.19858700 -0.30162300 -0.10778200  H -1.70768700 -0.17192500 -0.80260400  H 1.54282800 -0.24965300 0.81888100 |
| MCp1 | MP2/aTZ | N 0.88088500 0.72296700 0.00790700  N 1.07599000 -0.59339200 0.07552500  H 0.22735100 -1.13723600 0.01851700  H 1.92876700 -1.01585200 -0.25703600  H 1.79306700 1.15931000 -0.09538300  O -1.77500300 -0.08171500 -0.10000500  H -1.05136700 0.56847500 -0.03759400  H -2.39592200 0.17198900 0.58751800 |
|  | MP2/maTZ | N 0.87927700 0.72354600 0.01054800  N 1.07587400 -0.59261500 0.07826300  H 0.22588200 -1.13411300 0.03362400  H 1.91764600 -1.01436600 -0.28041700  H 1.78971300 1.15678400 -0.11417500  O -1.76785900 -0.08764800 -0.09557500  H -1.05303900 0.57107400 -0.03679200  H -2.42338900 0.20528800 0.54068700 |
|  | MP2/6-311++g(3df,3pd) | N 0.87972100 0.72248500 0.00731300  N 1.07596900 -0.59265600 0.07534400  H 0.22805800 -1.13713600 0.01852600  H 1.92918400 -1.01435300 -0.25674400  H 1.79064400 1.15953900 -0.09421200  O -1.77410300 -0.08200300 -0.09946800  H -1.05270400 0.56657200 -0.03715700  H -2.39218700 0.17259300 0.58673400 |
|  | M06-2X/aTZ | N 0.89974100 0.72236900 0.01094600  N 1.05735200 -0.60085200 0.07828100  H 0.19007800 -1.11784800 0.02031700  H 1.88914500 -1.04560600 -0.28050000  H 1.82258200 1.13711000 -0.10129700  O -1.76739600 -0.07174200 -0.10017900  H -1.06459700 0.59708800 -0.04754200  H -2.39769400 0.15258000 0.58586600 |
|  | M06-2X /maTZ | N 0.89859300 0.72301900 0.01121400  N 1.05668400 -0.60015200 0.07913400  H 0.18877200 -1.11729300 0.02924700  H 1.88465900 -1.04590600 -0.28780700  H 1.82076600 1.13759100 -0.10890600  O -1.75950500 -0.07726600 -0.09447500  H -1.06678100 0.60174700 -0.03917300  H -2.43831700 0.18191900 0.53000000 |
|  | M06-2X /6-311++g(3df,3pd) | N 0.90388800 0.72154400 0.01085100  N 1.05108100 -0.60287900 0.07935500  H 0.17888800 -1.11149200 0.01290600  H 1.87820100 -1.05316500 -0.28489100  H 1.83082400 1.12867500 -0.09646300  O -1.76679000 -0.06655300 -0.10300900  H -1.06902100 0.60611500 -0.05360600  H -2.36935000 0.13163300 0.61468700 |
|  | M06-HF/aTZ | N 0.86431900 0.71658600 0.00812300  N 1.05405600 -0.59332800 0.07278600  H 0.20381100 -1.13492700 0.00000200  H 1.91424100 -1.01025800 -0.24425300  H 1.76917700 1.16866900 -0.07676200  O -1.74441600 -0.07243900 -0.10051000  H -1.01309500 0.56678000 -0.05537400  H -2.34743100 0.12644000 0.61410300 |
|  | M06-HF /maTZ | N 0.85397500 0.71755200 0.00800500  N 1.06834300 -0.58832900 0.07144100  H 0.23005400 -1.14964900 0.00771800  H 1.93819000 -0.99145500 -0.23713500  H 1.74933400 1.18706900 -0.08345200  O -1.74697200 -0.08326400 -0.09595200  H -1.01544300 0.55674800 -0.05117700  H -2.38258100 0.15883400 0.57553900 |
|  | M06-HF /6-311++g(3df,3pd) | N 0.86451000 0.71621600 0.00758500  N 1.04311700 -0.59647700 0.07185000  H 0.18959600 -1.13025600 -0.01781900  H 1.90279300 -1.01866500 -0.23881700  H 1.77255600 1.16120200 -0.06322300  O -1.73934700 -0.06407000 -0.10441100  H -1.01183400 0.57917500 -0.06293600  H -2.29171900 0.08292800 0.66204200 |
|  | B3LYP/aTZ | N 0.86538400 0.72571600 0.01323100  N 1.13407300 -0.58714200 0.08302000  H 0.30983900 -1.16980900 0.02830900  H 1.98157500 -0.96404800 -0.31873300  H 1.74770500 1.21772600 -0.11794600  O -1.81612200 -0.09240600 -0.10345800  H -1.07744500 0.54130700 -0.03526900  H -2.42889800 0.14405300 0.59754900 |
|  | B3LYP/maTZ | N 0.86682400 0.72609600 0.01384700  N 1.12939300 -0.58809900 0.08365600  H 0.30152400 -1.16625000 0.03167900  H 1.97283700 -0.96954500 -0.32285700  H 1.75130100 1.21350700 -0.12160200  O -1.81043100 -0.09353800 -0.10162200  H -1.07868600 0.54859700 -0.03899500  H -2.43705000 0.15601900 0.58223100 |
|  | B3LYP /6-311++g(3df,3pd) | N 0.86705600 0.72542700 0.01243600  N 1.13014700 -0.58834400 0.08284800  H 0.30337300 -1.16746300 0.02155400  H 1.97868100 -0.96816000 -0.31497900  H 1.75224100 1.21425600 -0.11127700  O -1.81607200 -0.08889100 -0.10499000  H -1.07673700 0.54292800 -0.03961800  H -2.40940700 0.12998100 0.61725300 |
|  | B3LYP/6-311g(d,p) | N 0.90350600 0.72513000 0.01259600  N 1.04061500 -0.61367100 0.08088400  H 0.15080200 -1.08931100 -0.03182900  H 1.85254500 -1.07231400 -0.31739500  H 1.84073700 1.12365900 -0.06742700  H -1.06741800 0.61376000 -0.14192700  O -1.78167900 -0.04902700 -0.10975500  H -2.13207900 0.03621000 0.78225400 |
| MCp2 | MP2/aTZ | N -0.88188800 0.72410600 0.00391700  N -1.06879900 -0.59418200 -0.03638100  H -0.22621600 -1.13467200 0.09167100  H -1.94755900 -1.01255700 0.22496400  H 1.05352000 0.57105400 -0.05938800  H -1.80040400 1.15942100 0.00923200  O 1.76933700 -0.09104600 -0.07387800  H 2.42077400 0.23565700 0.55179300 |
|  | MP2/maTZ | N -0.88037800 0.72451000 0.00415300  N -1.07155400 -0.59276400 -0.03711400  H -0.23201300 -1.13569200 0.09383300  H -1.95050300 -1.00777100 0.22640300  H 1.05560500 0.56725200 -0.06256100  H -1.79792700 1.16063700 0.00653100  O 1.76877500 -0.09574800 -0.06909000  H 2.43816200 0.25933400 0.51924000 |
|  | MP2/6-311++g(3df,3pd) | N -0.88323400 0.72359500 0.00410100  N -1.06996400 -0.59325200 -0.03372000  H -0.22903900 -1.13631300 0.09082700  H -1.95205800 -1.01311900 0.21235800  H 1.05678500 0.56693200 -0.06018100  H -1.80039400 1.15929800 0.00672800  O 1.77298900 -0.09002600 -0.07540400  H 2.41317400 0.23101300 0.56082800 |
|  | M06-2X/aTZ | N -0.90120000 0.72381400 0.00193800  N -1.05333400 -0.60013600 -0.04012200  H -0.19296800 -1.11893700 0.07124600  H -1.91271000 -1.04200500 0.24901700  H 1.06847800 0.59558500 -0.06540100  H -1.82979200 1.13980900 0.02919600  O 1.76280500 -0.08380500 -0.06781000  H 2.44629200 0.23023800 0.52570600 |
|  | M06-2X /maTZ | N 0.90021700 0.72412400 -0.00228800  N 1.05254100 -0.59981800 0.04090600  H 0.19188800 -1.11861800 -0.07072000  H 1.91111000 -1.04224000 -0.25081400  H -1.06915200 0.59782200 0.06615500  H 1.82900200 1.14038400 -0.02768100  O -1.75883400 -0.08634600 0.06380300  H -2.46148600 0.24327200 -0.49768900 |
|  | M06-2X /6-311++g(3df,3pd) | N -0.90340100 0.72259300 0.00222900  N -1.05055300 -0.60182300 -0.04202900  H -0.18613900 -1.11572900 0.06482500  H -1.90420400 -1.04731200 0.26016000  H 1.07057800 0.59836800 -0.08082300  H -1.83358100 1.13456900 0.03764000  O 1.76875100 -0.07561400 -0.07669000  H 2.38101800 0.18962100 0.61032200 |
|  | M06-HF/aTZ | N -0.86398600 0.71772300 0.00380400  N -1.05425900 -0.59219500 -0.03443500  H -0.21093000 -1.13860900 0.06779900  H -1.93469400 -1.00422900 0.22581400  H 1.01455500 0.55867500 -0.08128700  H -1.77046100 1.17352400 0.02041400  O 1.74591000 -0.08193400 -0.07750100  H 2.36195900 0.18741200 0.60168700 |
|  | M06-HF /maTZ | N -0.85412300 0.71910900 0.00230300  N -1.06797100 -0.58713000 -0.03255200  H -0.23726400 -1.15216100 0.07296700  H -1.95860900 -0.98411200 0.21667500  H 1.01779800 0.55149600 -0.06308600  H -1.75204600 1.19196600 0.01338400  O 1.74398800 -0.09586800 -0.06684300  H 2.43287100 0.23591100 0.50654600 |
|  | M06-HF /6-311++g(3df,3pd) | N -0.86331100 0.71786900 0.00503300  N -1.04536300 -0.59424900 -0.03588900  H -0.19902400 -1.13512200 0.07031500  H -1.92253400 -1.01143100 0.22644600  H 1.01282300 0.56927500 -0.08247500  H -1.77112500 1.16784300 0.02146400  O 1.73754000 -0.07841400 -0.07982900  H 2.34025600 0.17140600 0.61888000 |
|  | B3LYP/aTZ | N -0.86463800 0.72617800 -0.00239200  N -1.13447300 -0.58667800 -0.04507300  H -0.32087300 -1.17399100 0.07350500  H -2.01543600 -0.95086700 0.28979400  H 1.08261900 0.53638000 -0.06596500  H -1.75184600 1.22535000 0.03483200  O 1.82019800 -0.10206200 -0.07437600  H 2.43773300 0.20312800 0.59509900 |
|  | B3LYP/maTZ | N -0.86457700 0.72665800 -0.00185200  N -1.13181500 -0.58682900 -0.04532600  H -0.31677500 -1.17207300 0.07504800  H -2.01209600 -0.95295200 0.28983900  H 1.08240800 0.53934400 -0.06679900  H -1.75319700 1.22402800 0.03137800  O 1.81492900 -0.10488000 -0.07070800  H 2.45497100 0.22189300 0.56644100 |
|  | B3LYP /6-311++g(3df,3pd) | N -0.86704900 0.72607200 -0.00099400  N -1.13004600 -0.58798800 -0.04574100  H -0.31276700 -1.17040800 0.07526700  H -2.00955900 -0.95698300 0.28900600  H 1.08100400 0.53697500 -0.06989700  H -1.75692200 1.22080900 0.03231100  O 1.81959500 -0.09895600 -0.07639700  H 2.42114600 0.19466100 0.61164200 |
|  | B3LYP/6-311g(d,p) | N -0.89965400 0.72624000 0.00439000  N -1.04759100 -0.61040800 -0.05914700  H -0.16084200 -1.09755600 0.01720500  H -1.86200500 -1.06809900 0.33394100  H 1.07306400 0.59644300 -0.18401600  H -1.83437700 1.13340500 0.06858100  O 1.78510800 -0.06139700 -0.08124500  H 2.13401600 0.11615800 0.79755100 |
| MCp3a | MP2/aTZ | N -2.41800100 -0.08477700 0.00005200  N 0.52481500 0.02533800 0.00015800  H 0.24840000 -0.52958500 0.80498000  H 0.24794800 -0.52931200 -0.80469600  H -3.36673800 0.29419400 0.00021100  O 1.96551000 -0.05467100 -0.00025900  H -1.81791100 0.74403000 0.00024900  H 2.21651900 0.87410900 -0.00014200 |
|  | MP2/maTZ | N -2.43505600 -0.08447500 0.00009400  N 0.53441200 0.02233600 0.00005100  H 0.25908600 -0.53346200 0.80351200  H 0.25882400 -0.53332100 -0.80341800  H -3.38223700 0.29686500 0.00023800  O 1.97326100 -0.05207200 -0.00018100  H -1.83652600 0.74452100 0.00023600  H 2.21927600 0.87694500 -0.00013800 |
|  | MP2/6-311++g(3df,3pd) | N -2.41662600 -0.08410000 0.00008900  N 0.52867400 0.02314200 0.00010700  H 0.24763400 -0.52914000 0.80422600  H 0.24726000 -0.52893100 -0.80402400  H -3.36475800 0.29442100 0.00016500  O 1.96117700 -0.05322400 -0.00023400  H -1.81655600 0.74375600 0.00028700  H 2.21266400 0.87239000 -0.00015500 |
|  | M06-2X/aTZ | N -2.35855200 -0.08896400 0.00031600  N 0.51089700 0.04942700 -0.00011200  H 0.19943300 -0.48551900 0.80543100  H 0.19936200 -0.48552600 -0.80562500  H -3.32876400 0.23684800 0.00000100  O 1.92854200 -0.07425200 -0.00016600  H -1.79789600 0.76953700 0.00027600  H 2.23311100 0.83543700 -0.00018200 |
|  | M06-2X /maTZ | N 2.35707400 -0.08624900 -0.00030600  N -0.50946800 0.04081500 0.00010200  H -0.20240600 -0.49698200 -0.80541500  H -0.20233200 -0.49697400 0.80559500  H 3.32595700 0.24382700 -0.00001000  O -1.92803800 -0.06832000 0.00016800  H 1.79267300 0.77001200 -0.00027200  H -2.22283100 0.84471300 0.00018500 |
|  | M06-2X /6-311++g(3df,3pd) | N -2.35127200 -0.09016800 0.00035000  N 0.51138200 0.05652600 -0.00004500  H 0.19390600 -0.47603100 0.80503400  H 0.19367300 -0.47597800 -0.80506600  H -3.32864200 0.21411900 -0.00032600  O 1.92422500 -0.07936700 -0.00025600  H -1.81185100 0.78203700 0.00056700  H 2.23834300 0.82628400 -0.00030000 |
|  | M06-HF/aTZ | N 2.38324800 -0.08166400 0.00048800  N -0.52568200 0.02640400 -0.00118400  H -0.21834500 -0.49768600 -0.81178800  H -0.21593700 -0.49596900 0.80959600  H 3.34511900 0.25666100 0.00200500  O -1.93454400 -0.05973600 0.00097500  H 1.79530500 0.75394600 -0.00314800  H -2.23275200 0.84776300 0.00040200 |
|  | M06-HF /maTZ | N 2.39726400 -0.07626600 0.00003600  N -0.53311700 0.01084300 -0.00019000  H -0.23321400 -0.51851100 -0.81051200  H -0.23296100 -0.51770200 0.81056700  H 3.36362600 0.25032700 0.00046700  O -1.94332900 -0.04876100 0.00017000  H 1.82236700 0.76883600 -0.00055300  H -2.22221100 0.86510100 -0.00025400 |
|  | M06-HF /6-311++g(3df,3pd) | N 2.36912500 -0.08295300 0.00002800  N -0.52275500 0.03483400 -0.00040600  H -0.20825900 -0.48575300 -0.80984400  H -0.20752800 -0.48441400 0.80959600  H 3.34068700 0.22625400 0.00144000  O -1.92790200 -0.06618300 0.00037600  H 1.81010700 0.77249300 -0.00125400  H -2.23638000 0.83771300 -0.00029600 |
|  | B3LYP/aTZ | N 2.60765800 0.01681300 -0.00081200  N -0.66785800 -0.44244100 0.00059300  H -0.76003300 -1.04615400 -0.81196000  H -0.76188100 -1.04689500 0.81237900  H 2.95229300 0.98380900 0.00399500  O -1.87949000 0.34052700 -0.00046000  H 1.58246300 0.11790700 0.00062400  H -1.55551700 1.24651300 0.00017700 |
|  | B3LYP/maTZ | N 2.60765800 0.01681300 -0.00081200  N -0.66785800 -0.44244100 0.00059300  H -0.76003300 -1.04615400 -0.81196000  H -0.76188100 -1.04689500 0.81237900  H 2.95229300 0.98380900 0.00399500  O -1.87949000 0.34052700 -0.00046000  H 1.58246300 0.11790700 0.00062400  H -1.55551700 1.24651300 0.00017700 |
|  | B3LYP/6-311++g(3df,3pd) | N 2.60158800 0.01720500 -0.00090100  N -0.66759600 -0.44365200 0.00065800  H -0.75999200 -1.04726100 -0.81212300  H -0.76197200 -1.04784300 0.81277800  H 2.93764900 0.98729600 0.00451300  O -1.87276100 0.34210300 -0.00052100  H 1.57539500 0.10952700 0.00051500  H -1.54693400 1.24658700 0.00018100 |
|  | B3LYP/6-311g(d,p) | N 2.58804200 0.01838100 0.00038700  N -0.64413200 -0.42216500 -0.00039300  H -0.71680800 -1.03372100 -0.81180900  H -0.71498800 -1.03234000 0.81222400  H 2.88558800 1.00515200 -0.00172200  O -1.88015800 0.32021300 0.00035200  H 1.55665700 0.09268100 -0.00064700  H -1.57654700 1.23301300 -0.00082300 |
| MCp3b | MP2/aTZ | O -0.92266000 0.72347500 0.10093700  H -1.46885200 1.09536700 -0.59904900  N 1.97305800 -0.12405300 -0.01875700  H -1.44555600 -1.03278100 0.77264400  H 1.34859000 0.67830500 0.10168200  H 2.91289900 0.26675400 0.06004200  N -1.09179300 -0.69845300 -0.11833800  H -0.13465300 -1.03790500 -0.18315400 |
|  | MP2/maTZ | O -0.92722000 0.72142600 0.09589700  H -1.51057900 1.08564400 -0.57576700  N 1.97471600 -0.11935100 -0.02017900  H -1.40418300 -1.03771400 0.79008800  H 1.36527000 0.69125400 0.11418200  H 2.91973500 0.25713500 0.05914400  N -1.09148000 -0.70025400 -0.11418700  H -0.13513900 -1.03048900 -0.21425600 |
|  | MP2/6-311++g(3df,3pd) | O -0.92714600 0.71925700 0.10081600  H -1.46840400 1.09029600 -0.59910100  N 1.97338200 -0.12195100 -0.01962800  H -1.44038000 -1.03687400 0.77031600  H 1.34812600 0.67790600 0.10701800  H 2.91221800 0.26858300 0.06300200  N -1.08802700 -0.69516000 -0.11769500  H -0.13188500 -1.03419300 -0.18649700 |
|  | M06-2X/aTZ | O -0.91015000 0.71394900 0.09441900  H -1.46694500 1.10607800 -0.58137600  N 1.95112000 -0.12053500 -0.02797700  H -1.40481000 -1.03576000 0.78778600  H 1.32137700 0.66983100 0.14915900  H 2.89275200 0.26115000 0.08868800  N -1.08330200 -0.68970500 -0.11058400  H -0.13590100 -1.04121200 -0.22968200 |
|  | M06-2X /maTZ | O -0.90787900 0.71366700 0.09373900  H -1.46966900 1.10701100 -0.57738800  N 1.94712400 -0.12019100 -0.02579800  H -1.40442100 -1.03537900 0.78814300  H 1.33416000 0.68312500 0.15238600  H 2.89727700 0.24509300 0.07441900  N -1.08386100 -0.68932200 -0.11072100  H -0.13715700 -1.04259700 -0.23184000 |
|  | M06-2X /6-311++g(3df,3pd) | O -0.90481900 0.71272900 0.09529500  H -1.44984100 1.10919100 -0.58642100  N 1.94757400 -0.12307400 -0.02899500  H -1.40804400 -1.03242000 0.78697200  H 1.30835400 0.66019000 0.14724600  H 2.88387600 0.26910900 0.09683900  N -1.08417500 -0.68594100 -0.11053800  H -0.13959000 -1.04479600 -0.23025900 |
|  | M06-HF/aTZ | O -0.92241900 0.70644100 0.09340400  H -1.47963400 1.10060800 -0.57514000  N 1.94888600 -0.10735900 -0.03810300  H -1.38142400 -1.04858400 0.78952800  H 1.32585000 0.68324900 0.14109500  H 2.89237200 0.22536100 0.15483000  N -1.07026900 -0.68976800 -0.10415400  H -0.12812900 -1.03227400 -0.26174500 |
|  | M06-HF /maTZ | O -0.92209300 0.70667400 0.09205900  H -1.49014100 1.10414400 -0.56551600  N 1.95551000 -0.10896900 -0.03368700  H -1.38789200 -1.04639400 0.79118300  H 1.36116400 0.70404700 0.14251900  H 2.91270000 0.20143700 0.12865300  N -1.08080100 -0.68800400 -0.10426900  H -0.14205300 -1.03781500 -0.26761800 |
|  | M06-HF/6-311++g(3df,3pd) | O -0.89975400 0.70696500 0.08974900  H -1.45354500 1.10841100 -0.57683100  N 1.93281500 -0.11145800 -0.04713100  H -1.34586700 -1.03727700 0.81000400  H 1.27592600 0.64164400 0.17172400  H 2.85750200 0.24349400 0.18877800  N -1.07407100 -0.68416700 -0.09746200  H -0.14719100 -1.04261300 -0.29951800 |
|  | B3LYP/aTZ | O -0.94443900 0.72377100 0.09785900  H -1.49736700 1.10612200 -0.58995700  N 2.01096600 -0.12021500 -0.02656800  H -1.45048700 -1.04085400 0.78183100  H 1.40054300 0.69356300 0.11830300  H 2.96213700 0.24250200 0.09476500  N -1.11137800 -0.70112700 -0.11382800  H -0.15643400 -1.04210300 -0.20504100 |
|  | B3LYP/maTZ | O -0.94571300 0.72287200 0.09659600  H -1.51066000 1.10099700 -0.58376300  N 2.00371100 -0.11666200 -0.02636500  H -1.43401500 -1.04480800 0.78591000  H 1.40782800 0.70739500 0.12226100  H 2.96170200 0.22968100 0.09054500  N -1.10522700 -0.70316000 -0.11295800  H -0.14853400 -1.03748900 -0.21246200 |
|  | B3LYP /6-311++g(3df,3pd) | O -0.93686600 0.72290000 0.09940200  H -1.47353300 1.11092800 -0.59698800  N 2.00642000 -0.12430900 -0.02619300  H -1.46031500 -1.03636900 0.77748800  H 1.37582800 0.67533900 0.11290200  H 2.94828600 0.26112900 0.09813400  N -1.11158700 -0.69681200 -0.11462700  H -0.15917300 -1.04638800 -0.20101200 |
| MCP4 | MP2/aTZ | N -0.01232300 -0.01660100 0.57476900  N -1.20251100 -0.06485100 -0.15712100  H -1.30222000 0.81621900 -0.64366700  H -1.16834300 -0.80816200 -0.85108700  H -0.01215600 -0.85463900 1.14581800  O 1.12535000 -0.27591800 -0.30166100  H 0.37446100 3.06699500 0.09414000  H 1.60929600 0.55710300 -0.25544700 |
|  | MP2/maTZ | N -0.01819100 -0.07693600 0.57710300  N -1.20597300 -0.02634100 -0.15712600  H -1.28028300 0.89612000 -0.56282800  H -1.19108500 -0.70374700 -0.91518900  H -0.04536500 -0.96136800 1.07098800  O 1.11545000 -0.28911800 -0.31395400  H 0.53880500 3.28566100 0.16504300  H 1.62347400 0.51921900 -0.18622300 |
|  | MP2/6-311++g(3df,3pd) | N -0.00921200 -0.07109000 0.57514700  N -1.19770200 -0.05867500 -0.15743800  H -1.30301700 0.86164700 -0.56235400  H -1.15989500 -0.73524000 -0.91584000  H -0.00928300 -0.95130900 1.07621000  O 1.12303400 -0.24536200 -0.31291800  H 0.33363800 3.11634200 0.17863700  H 1.60268700 0.57981800 -0.19726700 |
|  | M06-2X/aTZ | N -0.00339300 -0.13387200 0.56120100  N -1.19378700 -0.03044600 -0.15831900  H -1.32946900 0.94126400 -0.40766100  H -1.14931300 -0.57929600 -1.01357300  H 0.00930800 -1.06590900 0.95783400  O 1.12071200 -0.16067600 -0.32739300  H 0.29631700 2.48279700 0.39239700  H 1.58772600 0.65677700 -0.13001900 |
|  | M06-2X /maTZ | N -0.00437200 -0.14738500 0.55902200  N -1.19421800 -0.02564700 -0.15772900  H -1.33340600 0.95210100 -0.37969600  H -1.14998000 -0.55134100 -1.02748300  H 0.00607000 -1.08830400 0.93471500  O 1.11977200 -0.15619300 -0.32970600  H 0.31458000 2.49836500 0.40739600  H 1.59468700 0.64995300 -0.10633400 |
|  | M06-2X /6-311++g(3df,3pd) | N 0.19467700 -0.06921300 0.57157100  N -0.47612400 -1.04631100 -0.16389100  H -1.34802700 -0.65293100 -0.49305000  H 0.07195000 -1.32949300 -0.97305600  H 0.95944000 -0.54387400 1.03687500  O 0.87540400 0.83391700 -0.30365000  H -5.16956300 1.98405900 0.14053300  H 0.45310000 1.67957100 -0.13586000 |
|  | M06-HF/aTZ | - |
|  | M06-HF /maTZ | - |
|  | M06-HF /6-311++g(3df,3pd) | - |
|  | B3LYP/aTZ | N -0.00426500 0.36589900 0.37011500  N -1.20965700 -0.14997300 -0.09259200  H -1.35897100 0.16881800 -1.04125300  H -1.21799600 -1.16725600 -0.07519900  H 0.03077500 0.20519200 1.37009100  O 1.13451000 -0.40357300 -0.11098700  H 0.36935000 2.28408200 -0.63202000  H 1.59821900 0.22627300 -0.67638600 |
|  | B3LYP/maTZ | N -0.00469400 0.35443100 0.38244100  N -1.21130200 -0.14341700 -0.09775900  H -1.35948000 0.20438300 -1.03621200  H -1.22416800 -1.16092700 -0.11160900  H 0.02688600 0.15565800 1.37580600  O 1.13103700 -0.41091100 -0.12012300  H 0.41783600 2.37884400 -0.59687000  H 1.60260600 0.23222900 -0.66290300 |
|  | B3LYP /6-311++g(3df,3pd) | N -0.00214200 0.36354500 0.37321200  N -1.20828800 -0.14878400 -0.09253500  H -1.35609500 0.17656900 -1.03955000  H -1.21245400 -1.16655300 -0.08508800  H 0.03213000 0.19797500 1.37253000  O 1.13093200 -0.40201800 -0.11256400  H 0.36944200 2.27788000 -0.63278700  H 1.59253300 0.22694600 -0.67933200 |
| TS2 | MP2/aTZ | N -0.52994300 0.72852800 -0.13005100  N -1.32231200 -0.42176800 0.11458000  H -1.61793100 -0.77371900 -0.78598200  H -0.77618000 -1.15045000 0.56463600  H -0.44084400 1.22154000 0.75073200  O 1.65823900 -0.27990300 -0.06566900  H 0.45436700 0.45969300 -0.44889400  H 2.08046100 0.33483800 0.55315100 |
|  | MP2/maTZ | N -0.52941600 0.72684700 -0.12908100  N -1.32763400 -0.41729700 0.11173200  H -1.62029800 -0.77039600 -0.78821200  H -0.79254300 -1.14853200 0.56905700  H -0.44145700 1.21820600 0.75163900  O 1.66018500 -0.28653400 -0.05681600  H 0.45492500 0.45045400 -0.43807600  H 2.11723700 0.37569200 0.48156000 |
|  | MP2/6-311++g(3df,3pd) | N -0.52973900 0.72390600 -0.13098300  N -1.32773400 -0.41792600 0.11368000  H -1.62360900 -0.77291700 -0.78496400  H -0.79161100 -1.14795000 0.57206900  H -0.44566900 1.22444700 0.74525900  O 1.66630200 -0.27840300 -0.06610800  H 0.45304000 0.45031200 -0.44138100  H 2.07974400 0.33146800 0.55900500 |
|  | M06-2X/aTZ | N -0.59290800 0.74855200 -0.09547000  N -1.23865500 -0.48220600 0.12916300  H -1.68663200 -0.75786300 -0.73398400  H -0.56297200 -1.19870900 0.37914600  H -0.40966900 1.19349300 0.79279200  O 1.63440000 -0.26277200 -0.06826900  H 0.27739900 0.66585100 -0.64219100  H 2.12760900 0.33497500 0.51453400 |
|  | M06-2X /maTZ | N -0.59604600 0.75038500 -0.09250100  N -1.23221300 -0.48526300 0.12754300  H -1.68561000 -0.75839900 -0.73354100  H -0.55154100 -1.19949400 0.37129300  H -0.41268200 1.19257100 0.79704100  O 1.62712800 -0.26553500 -0.06162200  H 0.27107900 0.67701600 -0.64477500  H 2.15954600 0.35673600 0.45765900 |
|  | M06-2X /6-311++g(3df,3pd) | N -0.59252500 0.74875000 -0.09612200  N -1.23458200 -0.48217400 0.12800100  H -1.66605900 -0.76745100 -0.74081400  H -0.55812100 -1.19308000 0.39312000  H -0.41292000 1.19381800 0.79334400  O 1.62692600 -0.26235100 -0.06751300  H 0.28374600 0.66432100 -0.63603200  H 2.12769300 0.33517100 0.50733100 |
|  | M06-HF/aTZ | N 0.53488800 0.72666900 0.11014700  N 1.26844000 -0.44587800 -0.12217400  H 1.70870700 -0.72337500 0.74170100  H 0.67217700 -1.19785900 -0.44563100  H 0.43184300 1.24177100 -0.75113300  O -1.63805900 -0.25564300 0.08662100  H -0.39320400 0.55945200 0.56053200  H -1.93834500 0.19962800 -0.71425500 |
|  | M06-HF/maTZ | N 0.53579700 0.72712000 0.11257400  N 1.27370100 -0.44191500 -0.12279700  H 1.70933300 -0.72574100 0.74142700  H 0.68434100 -1.19322300 -0.46101400  H 0.43345300 1.24325300 -0.74825100  O -1.63502900 -0.26986200 0.07518700  H -0.39449500 0.55003700 0.55602800  H -2.01888200 0.28813700 -0.61812700 |
|  | M06-HF /6-311++g(3df,3pd) | - |
|  | B3LYP/aTZ | N 0.46877400 0.55061100 0.09049500  N 1.59152600 -0.29060400 -0.00762700  H 1.70610600 -0.82458600 0.84246500  H 1.57110100 -0.91212700 -0.80764200  H 0.66657500 1.44763500 -0.33566700  H -0.35756200 0.15659600 -0.38998600  O -1.99110200 -0.20879400 -0.09305000  H -2.07949700 -0.01722100 0.85516000 |
|  | B3LYP/maTZ | N 0.46793400 0.55042300 0.08949200  N 1.58969200 -0.29027800 -0.00747800  H 1.70932800 -0.81929200 0.84496700  H 1.57101200 -0.91420300 -0.80577200  H 0.66391800 1.44682300 -0.33884000  H -0.36087000 0.15469200 -0.38681100  O -1.98816900 -0.20970300 -0.09265900  H -2.08142200 -0.01140800 0.85363200 |
|  | B3LYP /6-311++g(3df,3pd) | N 0.46620700 0.54879000 0.09040800  N 1.58931800 -0.28931500 -0.00856900  H 1.70855800 -0.82117600 0.84262700  H 1.56733200 -0.91284600 -0.80725400  H 0.66244200 1.45026200 -0.32741200  H -0.35942600 0.15586600 -0.39206800  O -1.98638700 -0.20696600 -0.09322700  H -2.07648700 -0.03270100 0.85705000 |
|  | B3LYP/6-311g(d,p) | N 0.44028300 0.51449400 0.09984600  N 1.62353100 -0.25388900 -0.00008300  H 1.69089400 -0.87063400 0.80009400  H 1.68181400 -0.80519800 -0.85269100  H 0.59666000 1.41828800 -0.33402500  H -0.34832900 0.07688100 -0.41268200  O -2.01182400 -0.20555600 -0.09425000  H -1.97314700 0.00087400 0.85496000 |
| TS3 | MP2/aTZ | N 0.48518900 0.75743300 -0.09527800  N 1.12553500 -0.51729900 -0.02517500  H 1.92694100 -0.48623600 -0.64188500  H 1.45989000 -0.70556100 0.91141500  H -0.55698300 0.58108900 -0.34770100  H 0.45683100 1.17415200 0.83009100  O -1.67440300 -0.15734800 0.00939900  H -1.16652000 -0.98559700 0.01605100 |
|  | MP2/maTZ | N 0.48527700 0.75381300 -0.09681600  N 1.13191500 -0.51554500 -0.02360600  H 1.92922000 -0.48355400 -0.64407600  H 1.47348000 -0.69742100 0.91075000  H -0.55581800 0.57331700 -0.34329600  H 0.45849000 1.17526700 0.82541600  O -1.68032800 -0.15580100 0.01039200  H -1.18309100 -0.98907700 0.01102600 |
|  | MP2/6-311++g(3df,3pd) | N 1.15836300 -0.34890600 -0.00852100  N 0.09113800 0.54755200 -0.04351700  H 0.77823200 1.85794100 0.47005500  H 0.01513300 0.88580600 -0.99786100  H 1.30701700 -0.62987800 0.95293100  O -1.26476000 -0.29404400 -0.03418900  H 0.94569200 -1.16804400 -0.56835600  H -1.67449900 0.01600500 0.78100600 |
|  | M06-2X/aTZ | N 0.53140500 0.75571000 -0.07907100  N 1.13015400 -0.52722100 -0.03207500  H 1.96190100 -0.51198500 -0.60806900  H 1.40133500 -0.77326100 0.91056000  H -0.44654100 0.66549500 -0.44138600  H 0.46933300 1.16126500 0.84657400  O -1.72460500 -0.14564400 0.01083700  H -1.22009400 -0.97579000 -0.01635100 |
|  | M06-2X /maTZ | N 0.51320200 0.69392600 -0.08473300  N 1.30028200 -0.47046400 -0.03739600  H 2.23354800 -0.24968900 -0.35672400  H 1.34096400 -0.87833000 0.88664200  H -0.40047400 0.47277200 -0.52964700  H 0.34769600 1.08751200 0.83390800  O -1.75321700 -0.29654100 -0.05834900  H -2.19039100 0.37582600 0.48752200 |
|  | M06-2X /6-311++g(3df,3pd) | N 0.53148700 0.75498400 -0.08018500  N 1.12895800 -0.52741900 -0.03207100  H 1.96234800 -0.51191900 -0.60626400  H 1.40036000 -0.77289100 0.91108600  H -0.44986900 0.66249300 -0.43480400  H 0.47550600 1.16422700 0.84448500  O -1.72363900 -0.14487100 0.01107400  H -1.22234700 -0.97588800 -0.01731000 |
|  | M06-HF/aTZ | N 0.50815800 0.75698300 -0.08912600  N 1.09329200 -0.52412600 -0.02689200  H 1.90855900 -0.54755000 -0.62175600  H 1.35478400 -0.78172500 0.91352800  H -0.51252100 0.65843700 -0.37156400  H 0.53001900 1.21850000 0.81032100  O -1.67125600 -0.15291200 0.01285700  H -1.12094000 -0.95436700 -0.02126200 |
|  | M06-HF/maTZ | N 0.51030400 0.75721400 -0.09256700  N 1.09265500 -0.52583000 -0.02221400  H 1.88472700 -0.56521000 -0.64681400  H 1.39171600 -0.75965600 0.91312200  H -0.51277400 0.65661200 -0.36583600  H 0.53797500 1.22402900 0.80403200  O -1.67479200 -0.15260500 0.01363500  H -1.12402600 -0.95462500 -0.01011700 |
|  | M06-HF/6-311++g(3df,3pd) | N 0.49478200 0.75224100 -0.08177100  N 1.11159500 -0.51442100 -0.03518400  H 1.96455000 -0.48793700 -0.57369000  H 1.32262600 -0.80239400 0.90911800  H -0.51243700 0.64117100 -0.40385200  H 0.47655800 1.19103900 0.82824300  O -1.66782800 -0.15386100 0.01267400  H -1.15331400 -0.97573000 -0.04253100 |
|  | B3LYP/aTZ | N 0.52167400 0.72124900 -0.05053400  N 1.26334500 -0.48152700 -0.07577900  H 2.24702500 -0.25453200 -0.00134200  H 0.99941900 -1.09829900 0.68418700  H -0.28610700 0.66395600 -0.67575600  H 0.24661500 1.02842500 0.87508700  O -1.68893600 -0.29633200 -0.05751700  H -2.19059600 0.35305100 0.46214700 |
|  | B3LYP/maTZ | N 0.52232300 0.72144100 -0.04984700  N 1.26292100 -0.48113900 -0.07530700  H 2.24763400 -0.25583600 -0.01143400  H 1.00313200 -1.09765800 0.68616000  H -0.28691100 0.66235200 -0.67365700  H 0.24776800 1.02856800 0.87609800  O -1.68879500 -0.29750700 -0.05581200  H -2.19797200 0.36051600 0.44541100 |
|  | B3LYP /6-311++g(3df,3pd) | N 0.52061300 0.72095200 -0.05045500  N 1.26138000 -0.48104600 -0.07544000  H 2.24613800 -0.25384800 -0.01261700  H 1.00459400 -1.09319900 0.69089200  H -0.29084300 0.66006400 -0.67237300  H 0.24733400 1.02754500 0.87616700  O -1.68618800 -0.29690000 -0.05682200  H -2.19166300 0.35529900 0.45377100 |
|  | B3LYP/6-311g(d,p) | N 0.51309500 0.72090900 -0.05883200  N 1.26979000 -0.47805700 -0.07399100  H 2.25253700 -0.23066100 -0.03021200  H 1.03875700 -1.07189600 0.71704900  H -0.33061200 0.60401500 -0.64056200  H 0.24608000 1.02371200 0.87480500  O -1.68914900 -0.29957300 -0.05570800  H -2.17376700 0.37144400 0.45435100 |
| TS1 | MP2/aTZ | N 1.64859500 0.03052100 -0.00003100  N -0.05092300 -0.22418400 0.00018800  H -0.05975400 -0.82994300 -0.81181800  H -0.05975200 -0.82887100 0.81299600  H 1.78040900 0.64340900 -0.80279200  O -1.62256100 0.08485700 -0.00019300  H 1.78045400 0.64456100 0.80184100  H -1.64457700 1.04763500 0.00021700 |
|  | MP2/maTZ | N -1.64758000 0.02991400 -0.00012100  N 0.05091400 -0.22240300 0.00127500  H 0.05798400 -0.82348700 0.81575600  H 0.05802400 -0.83159600 -0.80714200  H -1.78056800 0.64706100 0.79807800  O 1.62234200 0.08398600 -0.00126700  H -1.78080000 0.63789000 -0.80529800  H 1.64328600 1.04566500 0.00066600 |
|  | MP2/6-311++g(3df,3pd) | N 1.64392200 0.03041800 -0.00004600  N -0.05132000 -0.22402400 0.00029900  H -0.05925000 -0.83002800 -0.81098700  H -0.05926100 -0.82822000 0.81293300  H 1.77741000 0.64256600 -0.80233200  O -1.61807700 0.08516300 -0.00030700  H 1.77746000 0.64447300 0.80077300  H -1.63995200 1.04514500 0.00029400 |
|  | M06-2X/aTZ | N -1.73167800 0.02234100 -0.00001700  N 0.05798300 -0.20909400 -0.00002000  H 0.06169900 -0.81835200 0.80999300  H 0.06169300 -0.81807800 -0.81023800  H -1.83405300 0.64367900 0.80219800  O 1.69421100 0.07714800 -0.00002000  H -1.83398600 0.64413800 -0.80188500  H 1.70682700 1.03870200 0.00035100 |
|  | M06-2X/maTZ | N -1.73151900 -0.02240100 -0.00019900  N 0.05807000 0.20913300 -0.00051200  H 0.06141300 0.81711100 -0.81177400  H 0.06122700 0.82083500 0.80796600  H -1.83373000 -0.64740400 -0.79981100  O 1.69413200 -0.07723400 0.00037400  H -1.83428200 -0.64086500 0.80442500  H 1.70646200 -1.03893300 0.00117600 |
|  | M06-2X\/6-311++g(3df,3pd) | N -1.72847900 0.02202400 -0.00004200  N 0.05741400 -0.20886800 -0.00001200  H 0.06200100 -0.81875200 0.80968900  H 0.06200300 -0.81851400 -0.80989100  H -1.82972500 0.64485500 0.80132600  O 1.69100400 0.07712400 -0.00000900  H -1.82961100 0.64542000 -0.80098300  H 1.70476300 1.03790400 0.00031600 |
|  | M06-HF/aTZ | N -1.70865000 -0.02411100 0.00004200  N 0.05573600 0.20774500 -0.00011600  H 0.05742400 0.80608000 -0.81660000  H 0.05742900 0.80707400 0.81563700  H -1.81602200 -0.63262900 -0.80725200  O 1.67384400 -0.07503400 0.00012500  H -1.81596700 -0.63202000 0.80780200  H 1.69678500 -1.03366700 -0.00007000 |
|  | M06-HF/maTZ | N -1.70847400 -0.02419000 -0.00008800  N 0.05589400 0.20826000 0.00007200  H 0.05637900 0.80916300 -0.81502500  H 0.05641100 0.80949700 0.81492700  H -1.81509200 -0.63448500 -0.80667300  O 1.67397800 -0.07554700 -0.00011100  H -1.81538200 -0.63372900 0.80702600  H 1.69392000 -1.03455700 0.00074000 |
|  | M06-HF/6-311++g(3df,3pd) | N -1.70719100 -0.02428200 0.00004600  N 0.05616000 0.20923400 0.00011800  H 0.05860400 0.80932600 -0.81448200  H 0.05865200 0.80825300 0.81550500  H -1.81096800 -0.63407800 -0.80665900  O 1.67117400 -0.07598900 -0.00007900  H -1.81077900 -0.63570800 0.80554400  H 1.69231600 -1.03453800 -0.00042700 |
|  | B3LYP/aTZ | N -1.77387400 0.02322100 0.00000000  N 0.06509900 -0.21300800 0.00001800  H 0.06775200 -0.82132800 0.81051600  H 0.06775300 -0.82139800 -0.81042600  H -1.87372700 0.64478500 0.80338300  O 1.73003300 0.07975600 -0.00001700  H -1.87372800 0.64465900 -0.80348100  H 1.73310700 1.04374900 0.00001600 |
|  | B3LYP/maTZ | N -1.77391600 0.02311000 -0.00000200  N 0.06508000 -0.21264300 -0.00000600  H 0.06712700 -0.82127400 0.81042400  H 0.06712400 -0.82119100 -0.81049600  H -1.87422200 0.64453400 0.80361100  O 1.73033200 0.07955700 0.00000500  H -1.87419500 0.64461400 -0.80355500  H 1.73336700 1.04359500 0.00002300 |
|  | B3LYP/6-311++g(3df,3pd) | N -1.77216100 0.02282100 -0.00009200  N 0.06462200 -0.21228400 -0.00018900  H 0.06789400 -0.82144600 0.80982400  H 0.06793900 -0.82007900 -0.81124300  H -1.87176900 0.64363000 0.80413800  O 1.72841400 0.07941800 0.00013600  H -1.87142800 0.64622800 -0.80233000  H 1.73282500 1.04255800 0.00049000 |
|  | B3LYP/6-311g(d,p) | N 1.77754700 0.02082300 -0.00000100  N -0.06214100 -0.21019700 0.00001100  H -0.06531000 -0.82473800 -0.80854500  H -0.06531000 -0.82465400 0.80863200  H 1.86984300 0.65090700 -0.80173100  O -1.73658300 0.07875600 -0.00001100  H 1.86984400 0.65099800 0.80165800  H -1.72424400 1.04305700 0.00000200 |
| TS1b | MP2/aTZ | N -1.32994659 -0.30424367 -0.11084286  N 0.14766859 0.51845367 0.02987386  H 0.20927059 1.22614267 -0.70763614  H 0.30691559 0.93983367 1.08463286  H -1.41760759 -0.79478167 0.78533814  O 1.20398159 -0.36382733 -0.15772714  H -2.03793659 0.43445833 -0.08842186  H 1.59913559 -0.38577233 0.75617586 |
|  | MP2/maTZ | - |
|  | MP2/6-311++g(3df,3pd) | N -1.25648219 -0.40263445 -0.13068642  N 0.13701119 0.55647945 0.04430842  H 0.13733519 1.27973245 -0.67968458  H 0.24260819 0.97560845 1.10766842  H -1.28755119 -0.94323745 0.73848558  O 1.26817519 -0.21700855 -0.14178258  H -2.03327619 0.25993755 -0.06581142  H 1.66417419 -0.20230555 0.76789142 |
|  | M06-2X/aTZ | N -1.34135700 -0.29853400 -0.11030100  N 0.15735100 0.51238100 0.02935100  H 0.21740700 1.22597800 -0.70569200  H 0.29499500 0.97169800 1.05845700  H -1.42915200 -0.81316600 0.77134000  O 1.20244300 -0.36710100 -0.15309100  H -2.04231600 0.44730200 -0.07144200  H 1.62756300 -0.39192900 0.73871900 |
|  | M06-2X /maTZ | N -1.26845307 -0.39831763 -0.12535989  N 0.14511707 0.55226063 0.02126789  H 0.14933007 1.26412763 -0.71474311  H 0.23733807 1.05375863 1.07388589  H -1.24874907 -0.99192963 0.70754211  O 1.26603507 -0.22648837 -0.12753111  H -2.03186707 0.26574737 0.03833911  H 1.70793607 -0.13186837 0.75578389 |
|  | M06-2X /6-311++g(3df,3pd) | N -1.35227221 -0.28311875 -0.11658570  N 0.16552321 0.51633475 0.01634270  H 0.23823021 1.21630175 -0.72537830  H 0.31822621 0.97185075 1.08018970  H -1.38612721 -0.87504775 0.71850630  O 1.19206721 -0.37764325 -0.14777930  H -2.04343521 0.45444225 0.05300530  H 1.60411721 -0.39982025 0.75413770 |
|  | M06-HF/aTZ | N -1.26649366 -0.39926218 -0.12900925  N 0.14674366 0.55040918 0.04581025  H 0.14865766 1.29185318 -0.66367375  H 0.21038666 1.00745918 1.05775625  H -1.28411566 -1.00039618 0.69539775  O 1.27379366 -0.21159582 -0.13256875  H -2.04502866 0.25703682 -0.03948425  H 1.73264266 -0.17219882 0.73083325 |
|  | M06-HF /maTZ | N -1.27029013 -0.39521001 -0.12825709  N 0.14816913 0.54887301 0.03218509  H 0.15557913 1.28446001 -0.68140891  H 0.21400513 1.02907801 1.04807709  H -1.25661713 -1.02082001 0.67735791  O 1.27078213 -0.21873699 -0.12609691  H -2.03978413 0.25964799 0.02888491  H 1.73431313 -0.13340599 0.73492309 |
|  | M06-HF/6-311++g(3df,3pd) | N -1.26581741 -0.39844817 -0.13046895  N 0.14823341 0.55305217 0.04151695  H 0.15157341 1.28922717 -0.67133905  H 0.20923841 1.01487717 1.05290795  H -1.26456541 -1.01226317 0.68433405  O 1.26958041 -0.21395783 -0.12970705  H -2.04247141 0.25385983 -0.00306995  H 1.72706541 -0.16639783 0.73302695 |
|  | B3LYP/aTZ | N -1.32350600 -0.32489700 0.00639500  N 0.12903600 0.49614000 -0.01629900  H 0.20296000 1.12522500 -0.82538600  H 0.24493500 1.01442000 0.97469800  H -1.79105600 -0.06092700 0.88208800  O 1.24923000 -0.35283200 -0.12079800  H -1.86536700 -0.05774600 -0.80871000  H 1.57598000 -0.39702000 0.81302600 |
|  | B3LYP/maTZ | - |
|  | B3LYP /6-311++g(3df,3pd) | N -1.25760383 -0.41324377 0.03177646  N 0.12731883 0.52962877 -0.04598946  H 0.14475983 1.12537077 -0.88167046  H 0.20305483 1.10592477 0.92993654  H -1.74817683 -0.13553877 0.89095046  O 1.31006083 -0.22573023 -0.11404146  H -1.81916083 -0.24781777 -0.79634454  H 1.65430183 -0.17376023 0.81262154 |
| TS1c | MP2/aTZ | N -1.24800900 -0.28111600 -0.09268900  N 0.10474600 0.58198000 0.00723000  H 0.13825300 1.07139400 -0.89332600  H 0.63713800 1.30188400 0.76786400  H -1.08720800 -0.96076600 0.65089800  O 1.05409200 -0.47638100 -0.07797100  H -1.92884400 0.37896000 0.28796300  H 1.81076300 -0.08646600 0.40857800 |
|  | MP2/maTZ | N 0.10161900 0.57806700 0.00905900  H 0.13590800 1.07198300 -0.88807100  H 0.64036100 1.30813400 0.75795000  H -1.08994700 -0.96385700 0.64527500  O 1.05126200 -0.47749700 -0.07426800  H -1.92646400 0.37734800 0.28398600  H 1.81518800 -0.06935500 0.38280100 |
|  | MP2/6-311++g(3df,3pd) | N -1.25846000 -0.27594000 -0.10769700  N 0.13750500 0.60245000 0.02184000  H 0.21679800 1.11991500 -0.85994400  H 0.57878500 1.25357300 0.79269900  H -1.13509100 -0.95451300 0.64138400  O 1.03217400 -0.49977700 -0.05194500  H -1.94675900 0.38888400 0.23937000  H 1.87555700 -0.09521600 0.20305000 |
|  | M06-2X/aTZ | N 1.26961600 -0.26061100 0.08652700  N -0.12292300 0.57830600 -0.01054600  H -0.13250900 1.02992800 0.90466700  H -0.80291400 1.39745700 -0.69543500  H 1.11372900 -0.96592300 -0.63046200  O -1.04105100 -0.49047600 0.07850400  H 1.92984300 0.39984300 -0.31984200  H -1.80659200 -0.16136000 -0.41882700 |
|  | M06-2X /maTZ | N -1.27293600 -0.25637100 -0.08596800  N 0.12611200 0.57981900 0.01491400  H 0.12865700 1.02258400 -0.90450900  H 0.85350600 1.42136400 0.66631800  H -1.12252300 -0.96319900 0.63093300  O 1.03671300 -0.49689100 -0.07890000  H -1.93134400 0.40589500 0.32047900  H 1.80576500 -0.17565500 0.41536100 |
|  | M06-2X/6-311++g(3df,3pd) | N 1.26927300 -0.26021700 0.08678600  N -0.12468100 0.57717800 -0.01098000  H -0.13252800 1.02979900 0.90362800  H -0.80447400 1.39481300 -0.69385400  H 1.11505900 -0.96281600 -0.63358100  O -1.03968900 -0.48940900 0.08002200  H 1.92775100 0.40231500 -0.31972100  H -1.80044600 -0.16756300 -0.42728600 |
|  | M06-HF/aTZ | N -1.28099800 -0.26388600 -0.10375200  N 0.13782300 0.57657900 -0.00059300  H 0.20164300 1.10686900 -0.87291100  H 0.47063900 1.22785700 0.82022500  H -1.07867100 -1.03739600 0.52178100  O 1.05836700 -0.46840100 -0.05566600  H -1.92295300 0.35996400 0.37577100  H 1.86463400 -0.09894100 0.33087500 |
|  | M06-HF /maTZ | N -1.27093800 -0.26192000 -0.10115000  N 0.12851800 0.57137500 -0.00154700  H 0.18983300 1.09912500 -0.87577400  H 0.49323600 1.24607000 0.80871600  H -1.07870800 -1.04377200 0.51717500  O 1.05677100 -0.46727300 -0.05599600  H -1.91709700 0.35653100 0.37898400  H 1.85550800 -0.08595400 0.33774800 |
|  | M06-HF/6-311++g(3df,3pd) | N -1.35122300 -0.12401400 -0.01237400  N 0.15610600 0.57640400 -0.03081900  H 0.23262500 1.06796200 -0.92468100  H 0.45071600 1.25367700 0.75444300  H -1.18712400 -0.93326100 -0.60449900  O 1.04337700 -0.49629200 -0.01580000  H -1.36488100 -0.46798900 0.94257200  H 1.88746700 -0.11678400 0.26091200 |
|  | B3LYP/aTZ | N -1.25903500 -0.28785500 -0.09206500  N 0.09998600 0.55744100 -0.00557400  H 0.13460700 1.09017100 -0.88153800  H 0.49797600 1.25321800 0.83965500  H -1.10490600 -0.98050700 0.63776800  O 1.09590200 -0.43720600 -0.09564200  H -1.93654100 0.37459900 0.28722400  H 1.75499400 -0.12693800 0.56550100 |
|  | B3LYP/maTZ | N -1.27914300 -0.26749000 -0.07849500  N 0.11492000 0.57020800 0.00779400  H 0.11363800 1.01039900 -0.91256100  H 0.86069400 1.46989700 0.68624100  H -1.15950400 -0.92508000 0.68951400  O 1.06297700 -0.49011400 -0.08912100  H -1.94958500 0.42297700 0.25860100  H 1.78050200 -0.17631100 0.48608000 |
|  | B3LYP/6-311++g(3df,3pd) | N -1.27260200 -0.27105900 -0.07972700  N 0.11161600 0.56766300 0.00164700  H 0.11714100 1.02133200 -0.91265900  H 0.79409600 1.42725300 0.72627500  H -1.14029500 -0.93739900 0.67869900  O 1.06592200 -0.47922500 -0.08993200  H -1.94350500 0.41182600 0.27226000  H 1.77208300 -0.16544400 0.50144400 |
| TS4 | MP2/aTZ | N 1.16182100 -0.32699300 -0.04155400  N 0.05664300 0.56335300 -0.02879600  H 0.64003100 1.63760400 0.52922000  H -0.02431400 0.94344600 -0.96912800  H 1.37097700 -0.53185700 0.92978200  O -1.21997900 -0.29872900 -0.04415800  H 0.83143500 -1.18290700 -0.47692800  H -1.58754400 -0.13097800 0.83277000 |
|  | MP2/maTZ | N 1.15785600 -0.33376200 -0.04461100  N 0.05901600 0.56563100 -0.02128900  H 0.67756300 1.64809600 0.50508200  H -0.02445500 0.94531900 -0.96037800  H 1.37177900 -0.54229100 0.92351500  O -1.22185600 -0.29373300 -0.04570900  H 0.81580500 -1.18640000 -0.47536200  H -1.58394300 -0.13794000 0.83411700 |
|  | MP2/6-311++g(3df,3pd) | N 1.15910200 -0.32570500 -0.04047900  N 0.05656100 0.56317000 -0.02899400  H 0.63561000 1.63234500 0.52744500  H -0.02319000 0.94409700 -0.96790700  H 1.36598400 -0.53436100 0.92995400  O -1.21566500 -0.30064900 -0.04338700  H 0.83061000 -1.17985200 -0.47940800  H -1.59334000 -0.11929200 0.82332100 |
|  | M06-2X/aTZ | N 1.14894900 -0.35358300 -0.02397600  N 0.08515000 0.56291200 -0.02763300  H 0.80932300 1.75171100 0.45875200  H 0.00971000 0.91939000 -0.97639200  H 1.31255900 -0.61843400 0.94002000  O -1.24417100 -0.29322500 -0.03657400  H 0.85924200 -1.17929700 -0.53734400  H -1.67616100 0.00713300 0.76881100 |
|  | M06-2X /maTZ | N 1.14706500 -0.35719700 -0.02570900  N 0.08655600 0.56383800 -0.02418200  H 0.82765200 1.75465900 0.44971700  H 0.01043400 0.92229600 -0.97234900  H 1.31520900 -0.62213500 0.93740300  O -1.24598400 -0.28977400 -0.03794600  H 0.85128500 -1.18249000 -0.53663100  H -1.67205400 -0.00062300 0.77466500 |
|  | M06-2X /6-311++g(3df,3pd) | N 1.14757400 -0.35359000 -0.02508000  N 0.08438800 0.56415400 -0.02661100  H 0.80814200 1.74516400 0.46064000  H 0.00917500 0.92297100 -0.97451800  H 1.30795800 -0.62080600 0.93917000  O -1.24085700 -0.29327100 -0.03698100  H 0.85147900 -1.17920900 -0.53569700  H -1.67362700 0.00410500 0.76809100 |
|  | M06-HF/aTZ | N 1.13921900 -0.35379800 -0.03722200  N 0.07786100 0.56339400 -0.02884600  H 0.80506300 1.68717800 0.47497800  H -0.00761000 0.94694200 -0.96428100  H 1.38077900 -0.54890000 0.92562100  O -1.23177300 -0.29055300 -0.03405300  H 0.81601300 -1.20372500 -0.48282100  H -1.65961900 -0.02424100 0.78140400 |
|  | M06-HF /maTZ | N 1.13932200 -0.35430600 -0.03249900  N 0.07938200 0.56462300 -0.02422600  H 0.82155500 1.70517100 0.45053900  H -0.00244200 0.94558500 -0.96118900  H 1.35200200 -0.58009100 0.93039500  O -1.22982200 -0.29594900 -0.03296700  H 0.82580900 -1.19259400 -0.50688400  H -1.68927500 0.01729800 0.74795300 |
|  | M06-HF/6-311++g(3df,3pd) | N 1.13901000 -0.35196500 -0.03414900  N 0.07650700 0.56611700 -0.02961300  H 0.79910300 1.69145700 0.47327100  H -0.00602500 0.94510700 -0.96557400  H 1.35637200 -0.56530200 0.93038200  O -1.22440200 -0.29805300 -0.03157400  H 0.81790600 -1.19618100 -0.49173100  H -1.68076000 0.01027300 0.75257800 |
|  | B3LYP/aTZ | N 1.16040200 -0.34831800 -0.00722000  N 0.09212700 0.54632500 -0.04462000  H 0.77453600 1.86513200 0.46897800  H 0.01592700 0.88227100 -0.99972300  H 1.30981200 -0.62879100 0.95391300  O -1.26824300 -0.29462300 -0.03397200  H 0.95498600 -1.16716300 -0.56965600  H -1.67702400 0.01948000 0.78114700 |
|  | B3LYP/maTZ | N 1.15829000 -0.35323000 -0.00894800  N 0.09486900 0.54774900 -0.03940500  H 0.79711600 1.87111500 0.45634000  H 0.01757500 0.88606400 -0.99362900  H 1.31017000 -0.63780200 0.95044600  O -1.27137500 -0.29013300 -0.03618200  H 0.94645500 -1.16922300 -0.57338800  H -1.67243500 0.00928100 0.78816000 |
|  | B3LYP/6-311++g(3df,3pd) | N 1.15836300 -0.34890600 -0.00852100  N 0.09113800 0.54755200 -0.04351700  H 0.77823200 1.85794100 0.47005500  H 0.01513300 0.88580600 -0.99786100  H 1.30701700 -0.62987800 0.95293100  O -1.26476000 -0.29404400 -0.03418900  H 0.94569200 -1.16804400 -0.56835600  H -1.67449900 0.01600500 0.78100600 |

**Table S32**. Optimized Cartesian coordinates, X, Y, and Z (Å) other paths at different levels for N_2_H_4_ + OH reaction.

| R 🡪 P1(N_2_H_3_+H_2_O) | | | | | | | | | |
| --- | --- | --- | --- | --- | --- | --- | --- | --- | --- |
| T/K | ${\Delta E}_{T}^{0}$ | ${\Delta H}_{T}^{0}$ | ${\Delta G}_{T}^{0}$ | ${T\Delta S}_{T}^{0}$ | T/K | ${\Delta E}_{T}^{0}$ | ${\Delta H}_{T}^{0}$ | ${\Delta G}_{T}^{0}$ | ${T\Delta S}_{T}^{0}$ |
| 200 | -37.27 | -37.27 | -38.05 | 0.78 | 500 | -37.17 | -37.17 | -39.31 | 2.14 |
| 250 | -37.23 | -37.23 | -38.25 | 1.02 | 550 | -37.18 | -37.18 | -39.52 | 2.35 |
| 298 | -37.20 | -37.20 | -38.03 | 0.84 | 600 | -37.19 | -37.19 | -39.74 | 2.54 |
| 350 | -37.18 | -37.18 | -38.67 | 1.49 | 700 | -37.24 | -37.24 | -40.16 | 2.92 |
| 400 | -37.17 | -37.17 | -38.88 | 1.71 | 900 | -37.37 | -37.37 | -40.98 | 3.61 |
| 450 | -37.16 | -37.16 | -39.10 | 1.93 | 1200 | -37.63 | -37.63 | -42.14 | 4.51 |
| R 🡪 P2(NH_2_OH+NH_2_) | | | | | | | | | |
| T/K | ${\Delta E}_{T}^{0}$ | ${\Delta H}_{T}^{0}$ | ${\Delta G}_{T}^{0}$ | ${T\Delta S}_{T}^{0}$ | T/K | ${\Delta E}_{T}^{0}$ | ${\Delta H}_{T}^{0}$ | ${\Delta G}_{T}^{0}$ | ${T\Delta S}_{T}^{0}$ |
| 200 | 1.15 | 1.15 | 0.23 | 0.92 | 500 | 1.24 | 1.24 | -1.24 | 2.48 |
| 250 | 1.19 | 1.19 | -0.01 | 1.20 | 550 | 1.23 | 1.23 | -1.49 | 2.72 |
| 298 | 1.23 | 1.23 | -0.22 | 1.46 | 600 | 1.21 | 1.21 | -1.73 | 2.95 |
| 350 | 1.24 | 1.24 | -0.50 | 1.73 | 700 | 5.27 | 5.27 | 1.98 | 3.29 |
| 400 | 1.24 | 1.24 | -0.74 | 1.99 | 900 | 1.07 | 1.07 | -3.18 | 4.25 |
| 450 | 1.25 | 1.25 | -0.99 | 2.24 | 1200 | 4.98 | 4.98 | -0.30 | 5.28 |
| R 🡪 P3(NH_2_NHOH+H) | | | | | | | | | |
| T/K | ${\Delta E}_{T}^{0}$ | ${\Delta H}_{T}^{0}$ | ${\Delta G}_{T}^{0}$ | ${T\Delta S}_{T}^{0}$ | T/K | ${\Delta E}_{T}^{0}$ | ${\Delta H}_{T}^{0}$ | ${\Delta G}_{T}^{0}$ | ${T\Delta S}_{T}^{0}$ |
| 200 | 23.16 | 23.16 | 24.99 | -1.83 | 500 | 23.66 | 23.66 | 27.55 | -3.89 |
| 250 | 23.19 | 23.19 | 25.44 | -2.26 | 550 | 23.79 | 23.79 | 27.93 | -4.14 |
| 298 | 23.24 | 23.24 | 25.87 | -2.63 | 600 | 28.20 | 28.20 | 32.71 | -4.51 |
| 350 | 23.32 | 23.32 | 26.32 | -3.00 | 700 | 28.51 | 28.51 | 33.44 | -4.93 |
| 400 | 23.42 | 23.42 | 26.74 | -3.32 | 900 | 29.17 | 29.17 | 34.76 | -5.59 |
| 450 | 23.53 | 23.53 | 27.16 | -3.62 | 1200 | 30.17 | 30.17 | 36.46 | -6.29 |
| T/K | ${\Delta E}_{T}^{0}$ | ${\Delta H}_{T}^{0}$ | ${\Delta G}_{T}^{0}$ | ${T\Delta S}_{T}^{0}$ | T/K | ${\Delta E}_{T}^{0}$ | ${\Delta H}_{T}^{0}$ | ${\Delta G}_{T}^{0}$ | ${T\Delta S}_{T}^{0}$ |

**Table S33.** The thermodynamic parameters (kcal/mol) of P1 – P3 adducts in the N_2_H_4_ + OH reaction at the MP2/aTZ Level.

| R 🡪 P1(N_2_H_3_+H_2_O) | | | | | | | | | |
| --- | --- | --- | --- | --- | --- | --- | --- | --- | --- |
| T/K | ${\Delta E}_{T}^{0}$ | ${\Delta H}_{T}^{0}$ | ${\Delta G}_{T}^{0}$ | ${T\Delta S}_{T}^{0}$ | T/K | ${\Delta E}_{T}^{0}$ | ${\Delta H}_{T}^{0}$ | ${\Delta G}_{T}^{0}$ | ${T\Delta S}_{T}^{0}$ |
| 200 | -36.37 | -36.37 | -37.17 | 0.80 | 500 | -36.24 | -36.24 | -38.47 | 2.23 |
| 250 | -36.33 | -36.33 | -37.37 | 1.04 | 550 | -36.24 | -36.25 | -38.69 | 2.45 |
| 300 | -36.30 | -36.30 | -37.59 | 1.29 | 600 | -36.26 | -36.26 | -38.92 | 2.66 |
| 350 | -36.27 | -36.27 | -37.80 | 1.54 | 700 | -36.29 | -36.29 | -39.36 | 3.06 |
| 400 | -36.25 | -36.25 | -38.03 | 1.77 | 900 | -36.41 | -36.41 | -40.22 | 3.80 |
| 450 | -36.24 | -36.24 | -38.25 | 2.01 | 1200 | -36.67 | -36.67 | -41.45 | 4.78 |
| R 🡪 P2(NH_2_OH+NH_2_) | | | | | | | | |  |
| T/K | ${\Delta E}_{T}^{0}$ | ${\Delta H}_{T}^{0}$ | ${\Delta G}_{T}^{0}$ | ${T\Delta S}_{T}^{0}$ | T/K | ${\Delta E}_{T}^{0}$ | ${\Delta H}_{T}^{0}$ | ${\Delta G}_{T}^{0}$ | ${T\Delta S}_{T}^{0}$ |
| 200 | 2.95 | 2.95 | 2.04 | 0.90 | 500 | 2.99 | 2.99 | 0.63 | 2.36 |
| 250 | 2.98 | 2.98 | 1.81 | 1.6 | 550 | 2.97 | 2.97 | 0.39 | 2.58 |
| 300 | 3.00 | 3.00 | 1.58 | 1.42 | 600 | 2.95 | 2.95 | 0.16 | 2.79 |
| 350 | 3.01 | 3.01 | 1.34 | 1.67 | 700 | 2.89 | 2.89 | 38.22 | 3.20 |
| 400 | 3.01 | 3.01 | 1.10 | 1.91 | 900 | 2.77 | 2.77 | -1.20 | 3.97 |
| 450 | 3.00 | 3.00 | 0.87 | 2.14 | 1200 | 2.55 | 2.55 | -2.49 | 5.04 |
| R 🡪 P3(NH_2_NHOH+H) | | | | | | | | | |
| T/K | ${\Delta E}_{T}^{0}$ | ${\Delta H}_{T}^{0}$ | ${\Delta G}_{T}^{0}$ | ${T\Delta S}_{T}^{0}$ | T/K | ${\Delta E}_{T}^{0}$ | ${\Delta H}_{T}^{0}$ | ${\Delta G}_{T}^{0}$ | ${T\Delta S}_{T}^{0}$ |
| 200 | 27.80 | 27.80 | 29.68 | -1.88 | 500 | 28.21 | 28.21 | 32.38 | -4.17 |
| 250 | 27.81 | 27.81 | 30.15 | -2.34 | 550 | 28.33 | 28.33 | 32.79 | -4.45 |
| 300 | 27.85 | 27.85 | 30.61 | -2.76 | 600 | 28.47 | 28.47 | 33.18 | -4.71 |
| 350 | 27.91 | 27.91 | 31.07 | -3.16 | 700 | 28.76 | 28.76 | 33.95 | -5.19 |
| 400 | 27.99 | 27.99 | 31.51 | -3.52 | 900 | 29.38 | 29.38 | 35.35 | -5.97 |
| 450 | 28.09 | 28.09 | 31.95 | -3.85 | 1200 | 30.33 | 30.33 | 37.20 | -6.86 |

**Table S34.** The thermodynamic parameters (kcal/mol) of P1-P3 adducts of N_2_H_4_+ OH reaction on the doublet potential energy surface at the UM06-2X/aug-cc-pVQZ level.

| R 🡪 P1(N_2_H_3_+H_2_O) | | | | | | | | | |
| --- | --- | --- | --- | --- | --- | --- | --- | --- | --- |
| T/K | ${\Delta E}_{T}^{0}$ | ${\Delta H}_{T}^{0}$ | ${\Delta G}_{T}^{0}$ | ${T\Delta S}_{T}^{0}$ | T/K | ${\Delta E}_{T}^{0}$ | ${\Delta H}_{T}^{0}$ | ${\Delta G}_{T}^{0}$ | ${T\Delta S}_{T}^{0}$ |
| 200 | -36.58 | -36.58 | -37.36 | 0.78 | 500 | -36.48 | -36.48 | -38.62 | 2.14 |
| 250 | -36.54 | -36.54 | -37.56 | 1.02 | 550 | -36.49 | -36.49 | -38.83 | 2.35 |
| 300 | -36.51 | -36.51 | -37.77 | 1.26 | 600 | -36.50 | -36.50 | -39.05 | 2.55 |
| 350 | -36.49 | -36.49 | -37.98 | 1.49 | 700 | -36.55 | -36.55 | -39.47 | 2.92 |
| 400 | -36.48 | -36.48 | -38.19 | 1.71 | 900 | -36.68 | -36.68 | -40.29 | 3.61 |
| 450 | -36.47 | -36.47 | -38.40 | 1.93 | 1200 | -36.94 | -36.94 | -41.45 | 4.51 |
| R 🡪 P2(NH_2_OH+NH_2_) | | | | | | | | |  |
| T/K | ${\Delta E}_{T}^{0}$ | ${\Delta H}_{T}^{0}$ | ${\Delta G}_{T}^{0}$ | ${T\Delta S}_{T}^{0}$ | T/K | ${\Delta E}_{T}^{0}$ | ${\Delta H}_{T}^{0}$ | ${\Delta G}_{T}^{0}$ | ${T\Delta S}_{T}^{0}$ |
| 200 | 1.82 | 1.82 | 0.90 | 0.92 | 500 | 1.91 | 1.91 | -0.57 | 2.48 |
| 250 | 1.86 | 1.86 | 0.67 | 1.20 | 550 | 1.90 | 1.90 | -0.81 | 2.72 |
| 300 | 1.89 | 1.89 | 0.42 | 1.47 | 600 | 1.89 | 1.89 | -1.06 | 2.95 |
| 350 | 1.91 | 1.91 | 0.18 | 1.73 | 700 | 1.68 | 1.69 | -1.61 | 3.29 |
| 400 | 1.92 | 1.92 | -0.07 | 1.99 | 900 | 1.74 | 1.74 | -2.51 | 4.25 |
| 450 | 1.92 | 1.92 | -0.32 | 2.24 | 1200 | 1.39 | 1.39 | -3.89 | 5.28 |
| R 🡪 P3(NH_2_NHOH+H) | | | | | | | | | |
| T/K | ${\Delta E}_{T}^{0}$ | ${\Delta H}_{T}^{0}$ | ${\Delta G}_{T}^{0}$ | ${T\Delta S}_{T}^{0}$ | T/K | ${\Delta E}_{T}^{0}$ | ${\Delta H}_{T}^{0}$ | ${\Delta G}_{T}^{0}$ | ${T\Delta S}_{T}^{0}$ |
| 200 | 28.85 | 28.85 | 30.68 | -1.83 | 500 | 29.35 | 29.35 | 33.24 | -3.89 |
| 250 | 28.88 | 28.88 | 31.13 | -2.26 | 550 | 29.49 | 29.49 | 33.63 | -4.14 |
| 300 | 28.93 | 28.93 | 31.58 | -2.65 | 600 | 29.63 | 29.63 | 34.00 | -4.36 |
| 350 | 27.97 | 29.01 | 32.01 | -3.00 | 700 | 29.71 | 29.71 | 34.63 | -4.93 |
| 400 | 29.11 | 29.11 | 32.43 | -3.32 | 900 | 30.37 | 30.37 | 35.96 | -5.59 |
| 450 | 29.22 | 29.22 | 32.85 | -3.62 | 1200 | 31.37 | 31.37 | 37.66 | -6.29 |

**Table S35.** The thermodynamic parameters (kcal/mol) of P1-P3 adducts of N_2_H_4_+ OH reaction on the doublet potential energy surface at the CCSD(T)/CBS (energy) + UMP2/aug-cc-pVTZ (corrections) level.

| CCSD(T)/aug-cc-pVQZ | | | CCSD(T)/aug-cc-pVTZ | |
| --- | --- | --- | --- | --- |
| Species | Largest amplitude | T1 Diagnostic | Largest amplitude | T1 Diagnostic |
| MCr1a | 0.010 | 0.010 | 0.011 | 0.010 |
| MCr1b | 0.010 | 0.010 | 0. 011 | 0.010 |
| MCr2 | 0.0583 | 0.019 | 0.061 | 0.019 |
| MCp1 | 0.041 | 0.017 | 0.045 | 0.018 |
| MCp2 | 0.043 | 0.017 | 0.037 | 0.018 |
| MCp3a | 0.018 | 0.011 | 0.020 | 0.011 |
| MCp3b | 0.011 | 0.011 | 0.011 | 0.011 |
| MCp4 | 0.009 | 0.012 | 0.0153 | 0.012 |
| TS1 | 0.049 | 0.028 | 0.048 | 0.028 |
| TS1b | 0.469 | 0.079 | 0.523 | 0.084 |
| TS1c | 0.054 | 0.025 | 0.052 | 0.024 |
| TS2 | 0.171 | 0.037 | 0.164 | 0.037 |
| TS3 | 0.198 | 0.036 | 0.199 | 0.036 |
| TS4 | 0.0273 | 0.023 | 0.027 | 0.023 |

**Table S36.** T1 diagnostics and Largest amplitudes calculated at the CCSD(T)/aQZ and CCSD(T)/aTZ levels.
